# Discovery of Daraxonrasib (RMC-6236), a Potent and Orally Bioavailable RAS(ON) Multi-selective, Noncovalent Tri-complex Inhibitor for the Treatment of Patients with Multiple RAS-Addicted Cancers

**DOI:** 10.1021/acs.jmedchem.4c02314

**Published:** 2025-03-08

**Authors:** James Cregg, Anne V. Edwards, Stephanie Chang, Bianca J. Lee, John E. Knox, Aidan C. A. Tomlinson, Abby Marquez, Yang Liu, Rebecca Freilich, Naing Aay, Yingyun Wang, Lingyan Jiang, Jingjing Jiang, Zhican Wang, Michael Flagella, David Wildes, Jacqueline A.M. Smith, Mallika Singh, Zhengping Wang, Adrian L. Gill, Elena S. Koltun

**Affiliations:** 475714 Revolution Medicines, Inc., Redwood City, California 94063, United States

## Abstract

Oncogenic RAS mutations are among the most common in human cancers. To target the active, GTP-bound state of RAS­(ON) directly, we employed an innovative tri-complex inhibitor (TCI) modality. Formation of a complex with an intracellular chaperone protein CypA, an inhibitor, and a target protein RAS blocks effector binding, inhibiting downstream RAS signaling and tumor cell proliferation. Herein, we describe the structure-guided SAR journey that led to the discovery of daraxonrasib (RMC-6236), a noncovalent, potent tri-complex inhibitor of multiple RAS mutant and wild-type (WT) variants. This orally bioavailable bRo5 macrocyclic molecule occupies a unique composite binding pocket comprising CypA and SWI/SWII regions of RAS­(ON). To achieve broad-spectrum RAS isoform activity, we deployed an SAR campaign that focused on interactions with residues conserved between mutants and WT RAS isoforms. Concurrent optimization of potency and drug-like properties led to the discovery of daraxonrasib (RMC-6236), currently in clinical evaluation in RAS mutant advanced solid tumors (NCT05379985; NCT06040541; NCT06162221; NCT06445062; NCT06128551).

## Graphical abstract

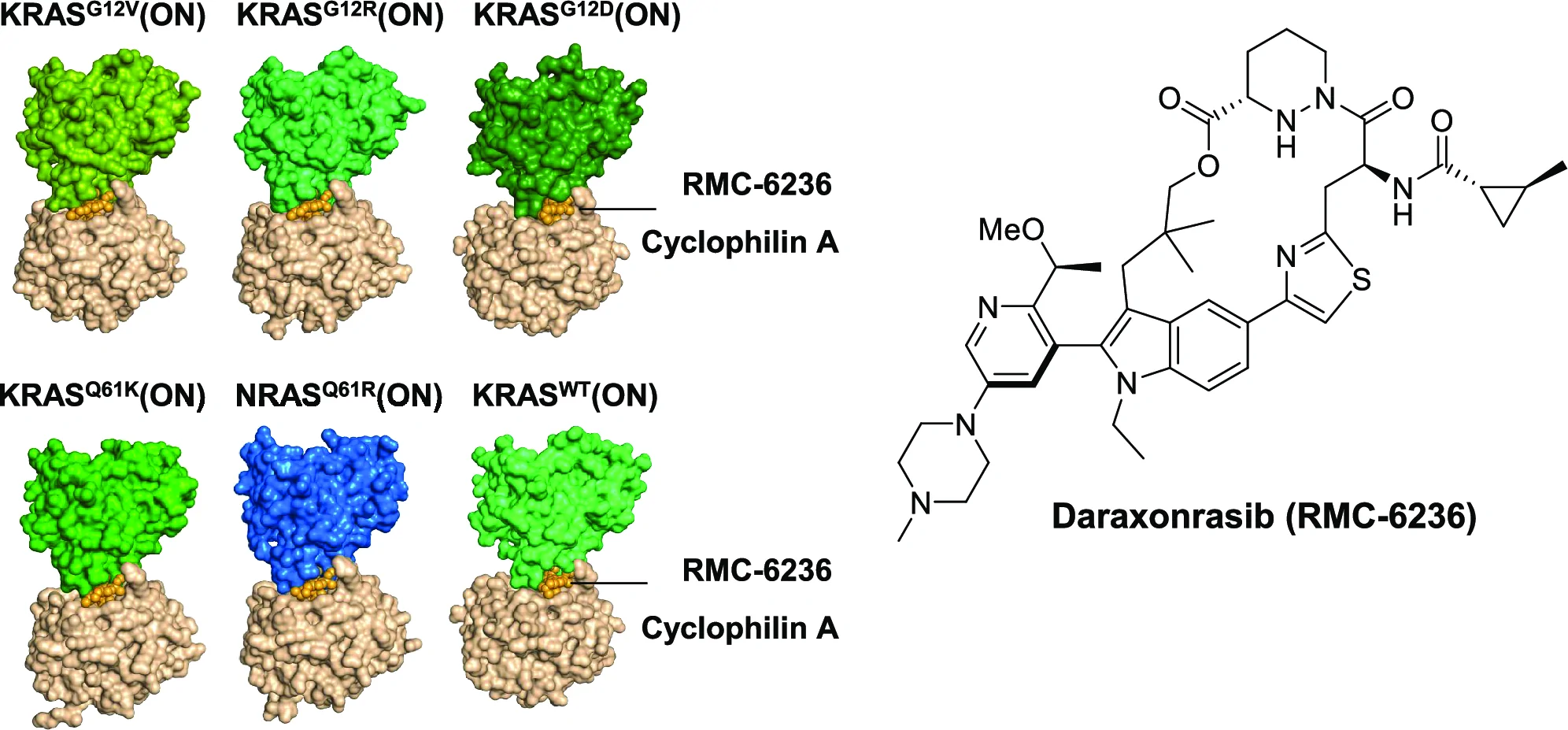

## Introduction

RAS (KRAS, NRAS, and HRAS) is one of the most frequently mutated oncogenes, with driver mutations occurring predominantly at glycine 12 (primarily D, V, C, R, and S), glycine 13 (primarily C and D) and glutamine 61 (primarily R and H).^1–3^ The scope of unmet medical need for patients with tumors driven by mutations in RAS remains very large, accounting for more than 200,000 new cases per year in the US alone^4^ with significant representation in three life-threatening tumor types, lung, colorectal, and pancreatic cancer (Figure 1), and with multiple mutants broadly distributed across multiple tumor types (Figure 1).^1,5–8^ These alterations in RAS disrupt intrinsic and GTPase activating protein (GAP) stimulated GTP hydrolysis and/or accelerate GDP to GTP nucleotide exchange, resulting in a shift in the population toward the active, oncogenic GTP-bound, RAS­(ON) state and activation of downstream effectors and signaling pathways ultimately leading to uncontrolled cell proliferation and survival.^9^ Recent clinical successes with KRAS^G12C^ mutant selective inhibitors such as sotorasib and adagrasib, which target the inactive GDP-bound, KRAS­(OFF) form, have demonstrated therapeutic value in targeting KRAS^G12C^ in KRAS^G12C^ mutant non-small cell lung cancer (NSCLC) and colorectal cancers (CRC).^5,6,10–13^ KRAS^G12C^ mutations represent a small percentage of RAS-driven cancers, leaving the majority of RAS cancer patients without a targeted therapeutic option (Figure 1). This includes patients who had an inadequate response post-treatment with first generation KRAS^G12C^(OFF) inhibitors or have developed resistance through numerous recurring mechanisms of escape to these inhibitors, including secondary RAS mutations and KRAS^G12C^ switch II binding pocket mutations.^14–18^ One approach to address this unmet medical need is through an inhibitor with broad-spectrum activity against multiple RAS mutants as well as the RAS wild type (RAS WT).

**Figure 1:**
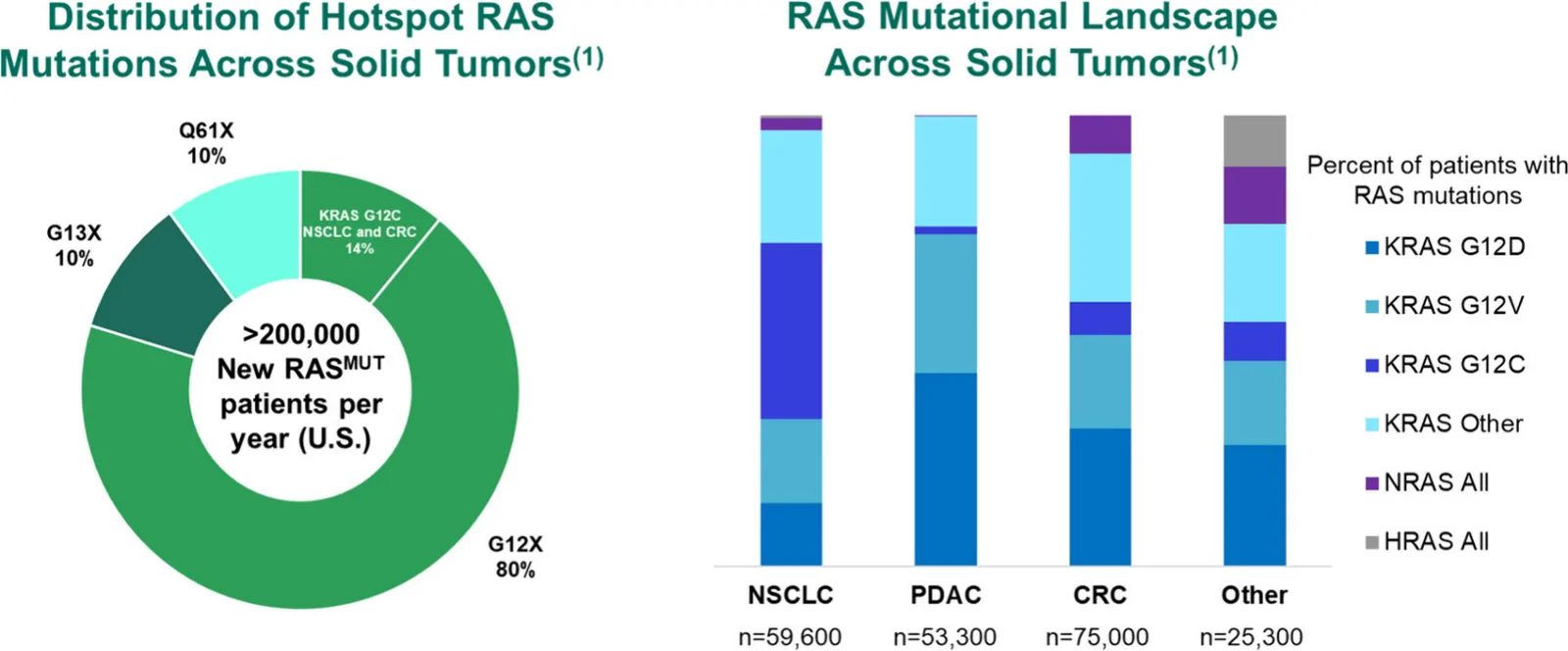
Heterogeneity of RAS mutations across solid tumors. (1) Percentages estimated based on frequencies of KRAS, NRAS, and HRAS mutations in codons G12X, G13X, and Q61X across solid tumors from Foundation Medicine Insights March 2022 and total incidence from ACS Cancer Facts and Figures 2023. NSCLC (non-small cell lung cancer); CRC (colorectal cancer); PDAC (pancreatic ductal adenocarcinoma) ().^19–22^

Efforts to develop pan-KRAS inhibitors have recently gained prominence.^23^ Several novel pan-KRAS inhibitors have emerged, including BI-3706674, LUNA18, LY4066434, and PF-07934040.^24–27^ These inhibitors were developed akin to irreversible KRAS^G12C^ inhibitors that bind to a shallow pocket located between switch I (SWI) and switch II (SWII) of the protein and preferentially bind the inactive state of KRAS (i.e., KRAS­(OFF)).^28–31^ Efforts at Revolution Medicines have focused instead on the active state of RAS (i.e., RAS­(ON)). We have recently described RMC-4998 and elironrasib (RMC-6291), two covalent inhibitors designed to target the active state of RAS^G12C^,^32^ and RMC-7977 a noncovalent, reversible RAS inhibitor with broad spectrum activity for the active state of both mutant and wild-type (WT) KRAS, NRAS, and HRAS variants.^33^ This report describes efforts that led to discovery and development of the investigational agent daraxonrasib (RMC-6236), a potent, orally bioavailable, RAS­(ON) multi-selective noncovalent, tri-complex inhibitor of both, mutant and WT KRAS, HRAS, and NRAS isoforms.^18^ These beyond rule of five (bRo5) macrocyclic inhibitors of the active (ON) form of RAS were derived from sanglifehrin A, a natural product that binds to cyclophilin A (CypA) with high affinity (Figure 2).^32,34–37,27,29^

**Figure 2:**
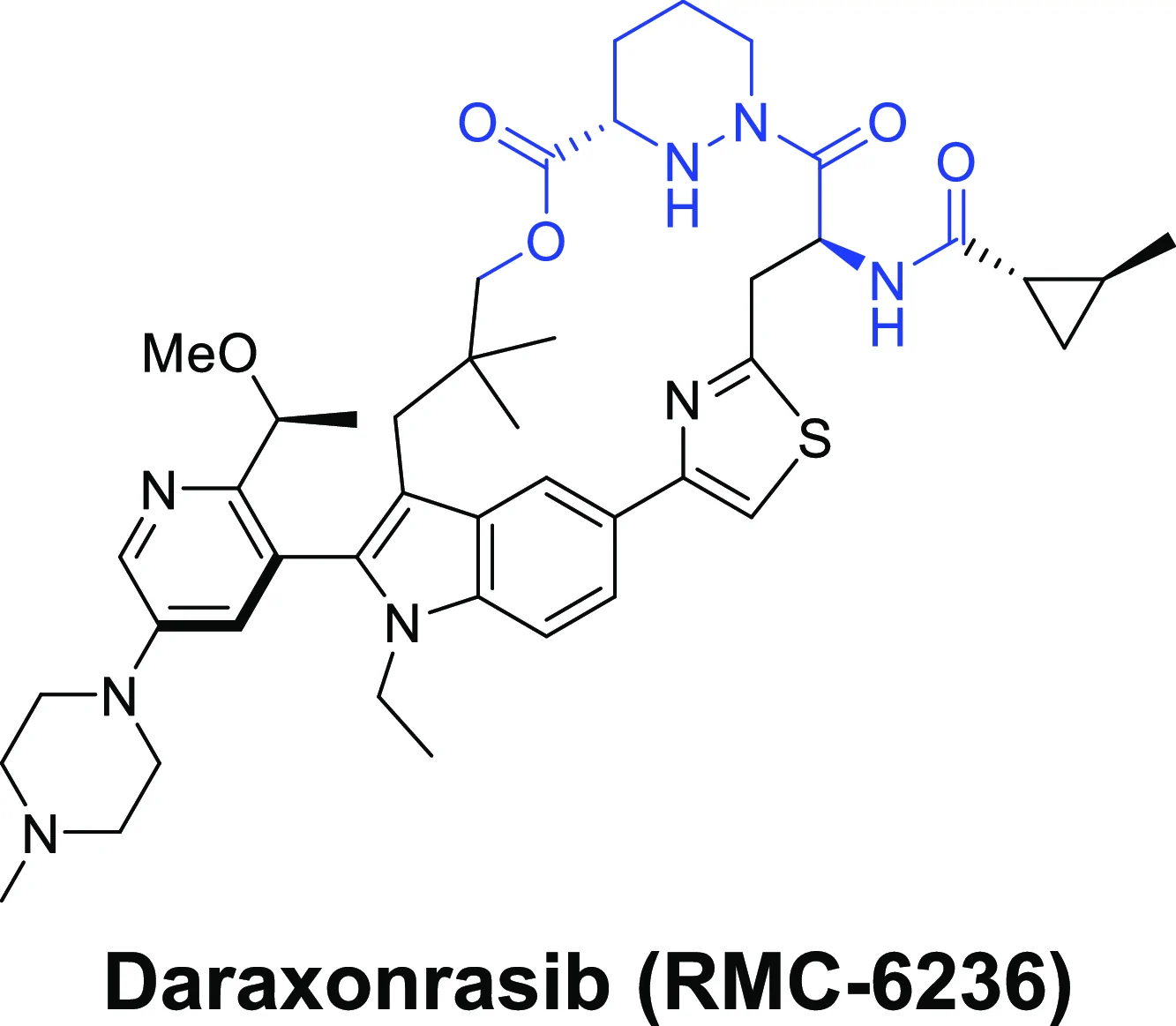
A minimal CypA binding motif (blue) was maintained from the natural product sanglifehrin A () and the remainder of the molecule was designed to optimize binding affinity to both tri-complex partners (RAS and CypA) alongside drug-like properties, including solubility and oral bioavailability, through multiparameter optimization.^18,32,33^

Historically, it has been challenging to directly target the GTP-bound form of RAS due to the absence of druggable ligand binding sites.^5,6^ In our approach, as previously described,^32^ when the small molecule inhibitor enters a cell (daraxonrasib (RMC-6236), Figure 3), it forms a binary complex with the chaperone protein CypA and remodels its surface to create a neomorphic interface with high affinity for the active state of RAS. This binary complex in turn engages the target protein RAS, which results in the compound occupying a new composite binding pocket on the otherwise featureless surface of the protein. Formation of the tri-complex leads to rapid disruption of the downstream activity of the target proteins as effector proteins (e.g., RAF, as shown in Figure 3) are sterically blocked from binding RAS by the bulky chaperone CypA. It has also been demonstrated that in the absence of the inhibitor, CypA has no affinity for binding to RAS, and conversely, an inhibitor of this sort has no measurable affinity for RAS in the absence of chaperone protein CypA.^32,33^

**Figure 3:**
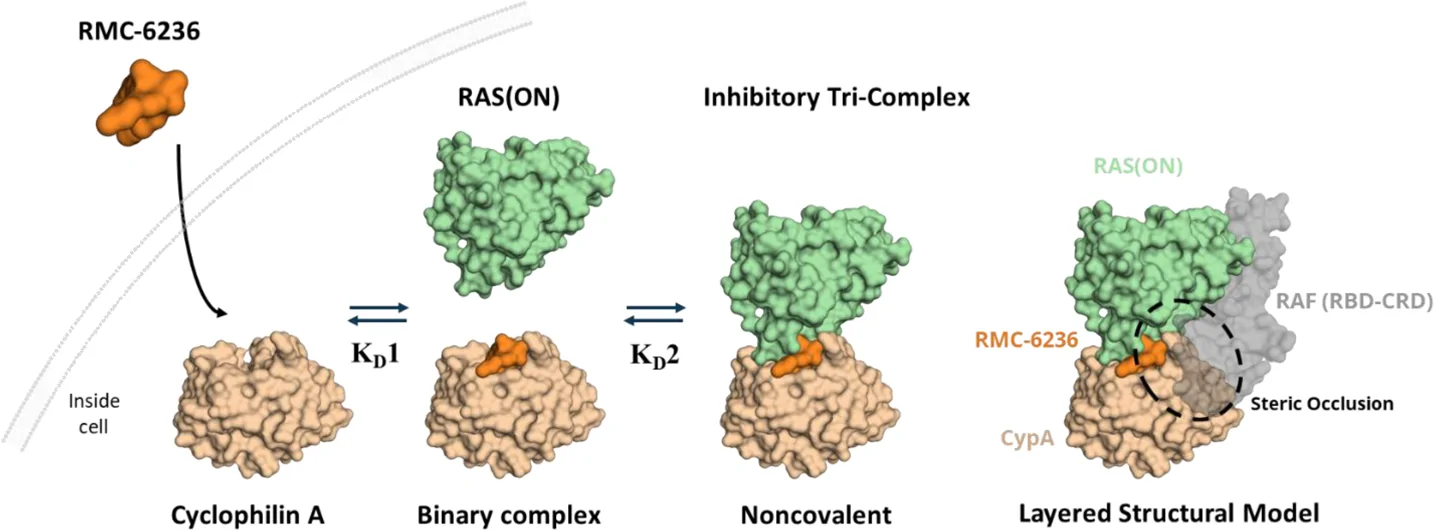
Tri-complex platform enables the selective targeting of oncogenic RAS­(ON) proteins.

## Results and Discussion

As previously reported, reversible RAS inhibitor compound **1** was identified in the course of the SAR exploration that led to the discovery of the covalent RAS­(ON) G12C-selective inhibitor elironrasib (RMC-6291).^32,37^ In this investigation, compound **1** was designed to probe the effect of a noncovalent and reversible tri-complex inhibitor. We have subsequently reported the discovery and comprehensive characterization of a preclinical RAS­(ON) multi-selective noncovalent tool compound, RMC-7977.^33^

Compound **1** is a beyond rule of bRo5 macrocyclic compound that contains a CypA-binding motif (K_D1_ = 862 nM) and forms a tri-complex with multiple mutants including KRAS^G12V^ (K_D2_ = 5827 nM). The CypA:compound **1** binary complex exhibited weak binding affinity (K_D2_ assessed by surface plasmon resonance (SPR) assay) for KRAS^G12V^ and this was sufficient to result in disruption of the interaction between KRAS^G12V^ and the RAS binding domain (RBD) of BRAF (TR-FRET IC_50_ = 4400 nM), demonstrating that it is possible to modulate the interaction of RAS with one of its effectors with a reversible tri-complex inhibitor. In addition, this acetamide derivative displayed weak inhibition of the RAS-BRAF RBD interaction for multiple oncogenic mutants and wild-type KRAS, NRAS, and HRAS proteins (Figure 4). We hypothesized that further optimization of binding interactions within the SWI and SWII areas conserved among most RAS mutants and RAS WT would result in potent compounds with broad spectrum RAS­(ON) activity. Therefore, compound **1** was selected as a starting point for the development of a reversible RAS­(ON) multi-selective inhibitor series. The concurrent multiparameter SAR optimization with focus on potency and drug-like properties was in good alignment with previous experiences reported for bRo5 macrocycles.^38,39^

**Figure 4:**
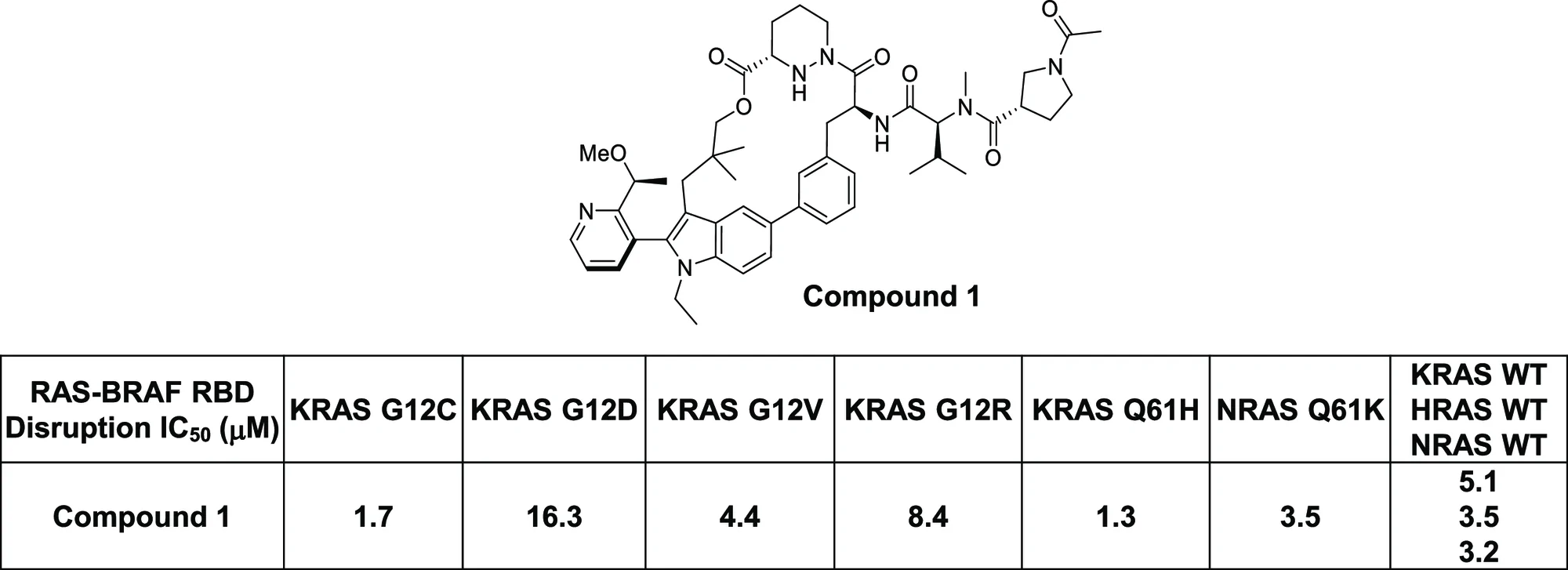
Compound **1** displayed weak inhibition of the RAS-BRAF RBD interaction for multiple oncogenic mutants and wild-type KRAS, NRAS, and HRAS proteins and demonstrated that noncovalent binding is sufficient for formation of a tri-complex and disruption of effector binding for multiple RAS mutants.

We focused the initial SAR exploration on potency through optimization of binding affinity to both CypA and RAS (K_D1_ and K_D2_ assessed by SPR assay), while probing the opportunities to enhance drug-like properties and ultimately bring oral bioavailability to a sufficient level for *in vivo* exploration. A biochemical assay based on concentration-dependent disruption of interaction between RAS G12V, G12C, G12D, and KRAS WT variants and the RAS binding domain (RBD) of BRAF kinase detected by time-resolved Foerster resonance energy transfer (TR-FRET) was employed to assess the potency of our compounds. Cell-based CellTiter-Glo proliferation assays in Capan-1 cells harboring a KRAS^G12V^ mutation, AsPC-1 cells harboring a KRAS^G12D^ mutation, and H358 cells harboring a KRAS^G12C^ mutation were employed to assess the cellular activity. Compound **1** occupies a composite binding pocket comprising CypA and SWI/SWII regions of RAS. The interactions of compound **1** with RAS are primarily with residues that are conserved between mutants and WT. We initiated structure-enabled exploration on the right-hand side (RHS) of the molecule. The RHS side chain interacted primarily with SWI and P-loop regions of RAS, and we probed if modifications reducing peptidic character could be tolerated for binding to both CypA and RAS. Through these efforts, we identified compound **2**, an analog for which the CypA:compound binary complex affinity (K_D2_) for the active state of multiple RAS mutants was comparable, including KRAS^G12V^ GMPPNP-bound K_D2_ = 4933 nM, KRAS^G12D^ GMPPNP-bound K_D2_ = 10,726 nM, and KRAS^G12C^ GMPPNP-bound K_D2_ = 2823 nM, and sufficiently disrupted effector binding of multiple RAS mutants (RAS-BRAF RBD disruption assay), which translated to improved cellular activity in KRAS G12V, 12C, and 12D mutant cells lines (Capan-1 KRAS^G12V^ CTG EC_50_ 2D, 1143 nM; AsPC-1 KRAS^G12D^ CTG EC_50_ 2D, 4410 nM; H358 KRAS^G12C^ CTG EC_50_ 2D, 1530 nM) (Table 1).

**Table 1:**
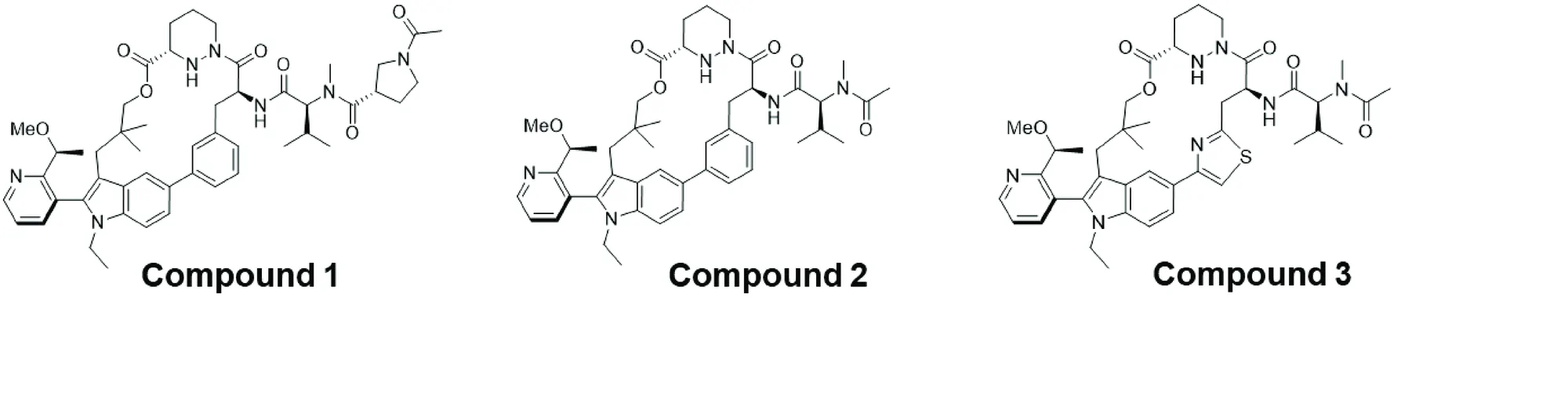

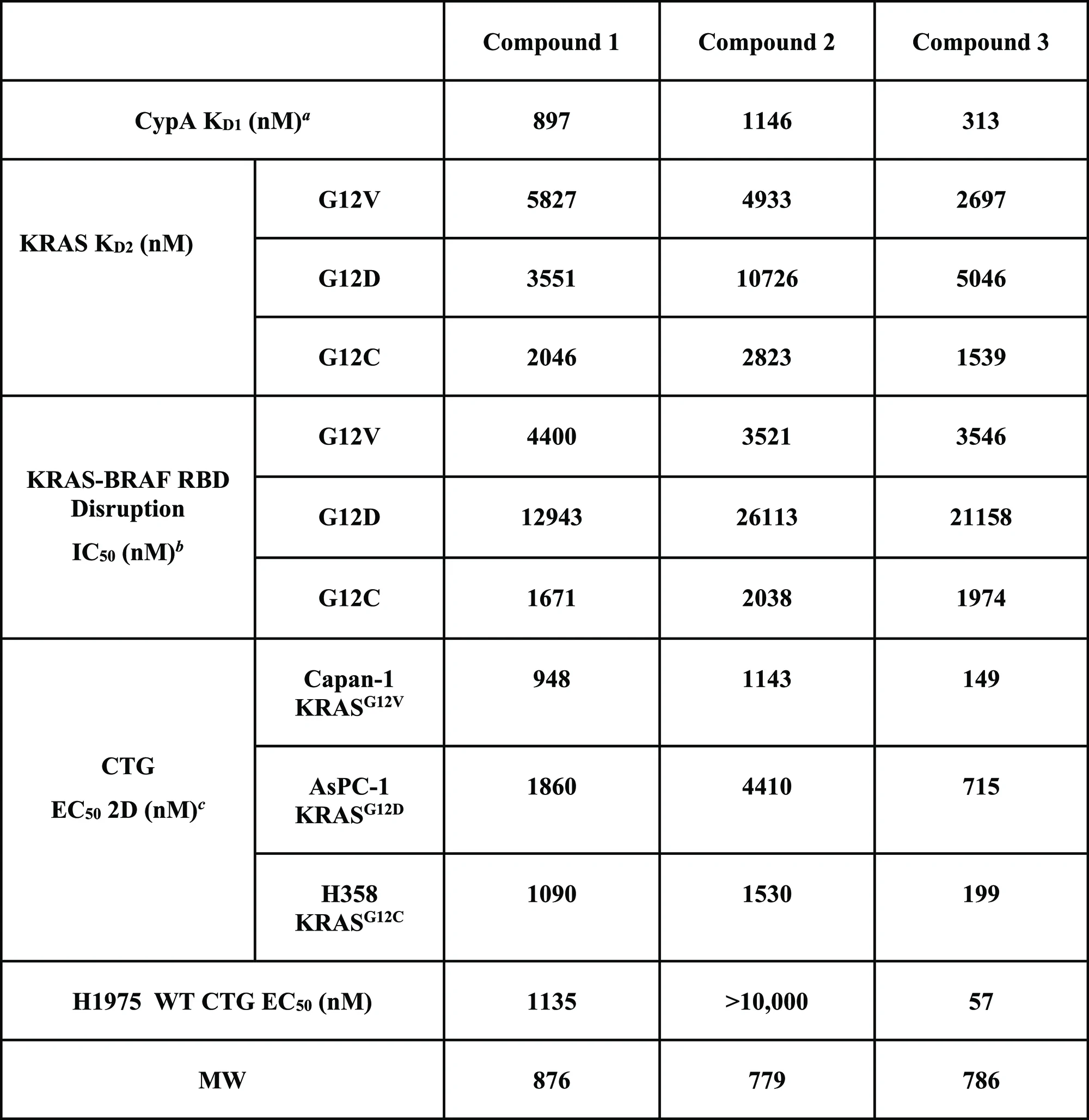
Initial SAR

a CypA binding affinity assessed by surface plasmon resonance (SPR).

b KRAS^G12X^ GMPPNP-bound BRAF RBD disruption assay, 3 h incubation.

c Viability assessed at 120 h by CellTiter-Glo luminescence assay (Promega) in Capan-1/NCI-H358 cells.

Next, we turned our attention to optimizations of the interaction of the central ring of the molecule with CypA and RAS. Multiple replacements of the phenyl moiety in the macrocycle were evaluated (Table 1), including aromatic and sp3 rich moieties (). Introduction of a thiazole moiety resulted in significant improvement in CypA binding affinity (compound **3** (K_D1_ = 313 nM), and comparable binary complex affinity for KRAS^G12V^ GMPPNP-bound (K_D2_ = 2697 nM), leading to significant improvement in cellular potency (Capan-1 KRAS^G12V^ CTG EC_50_ 2D, 149 nM). The nitrogen of the thiazole moiety in compound **3** forms a favorable through-water interaction with CypA at residue H126 (Figure 5C). The thiazole core also induces movement of the R55 residue in CypA, resulting in a shorter 3.0 Å hydrogen bond with the carbonyl of the valine backbone compared to a 3.2 Å hydrogen bond with the piperazic ester carbonyl in phenyl-containing compound **2** (Figure 5D), ultimately resulting in improvement in CypA binding affinity (K_D1_).

**Figure 5:**
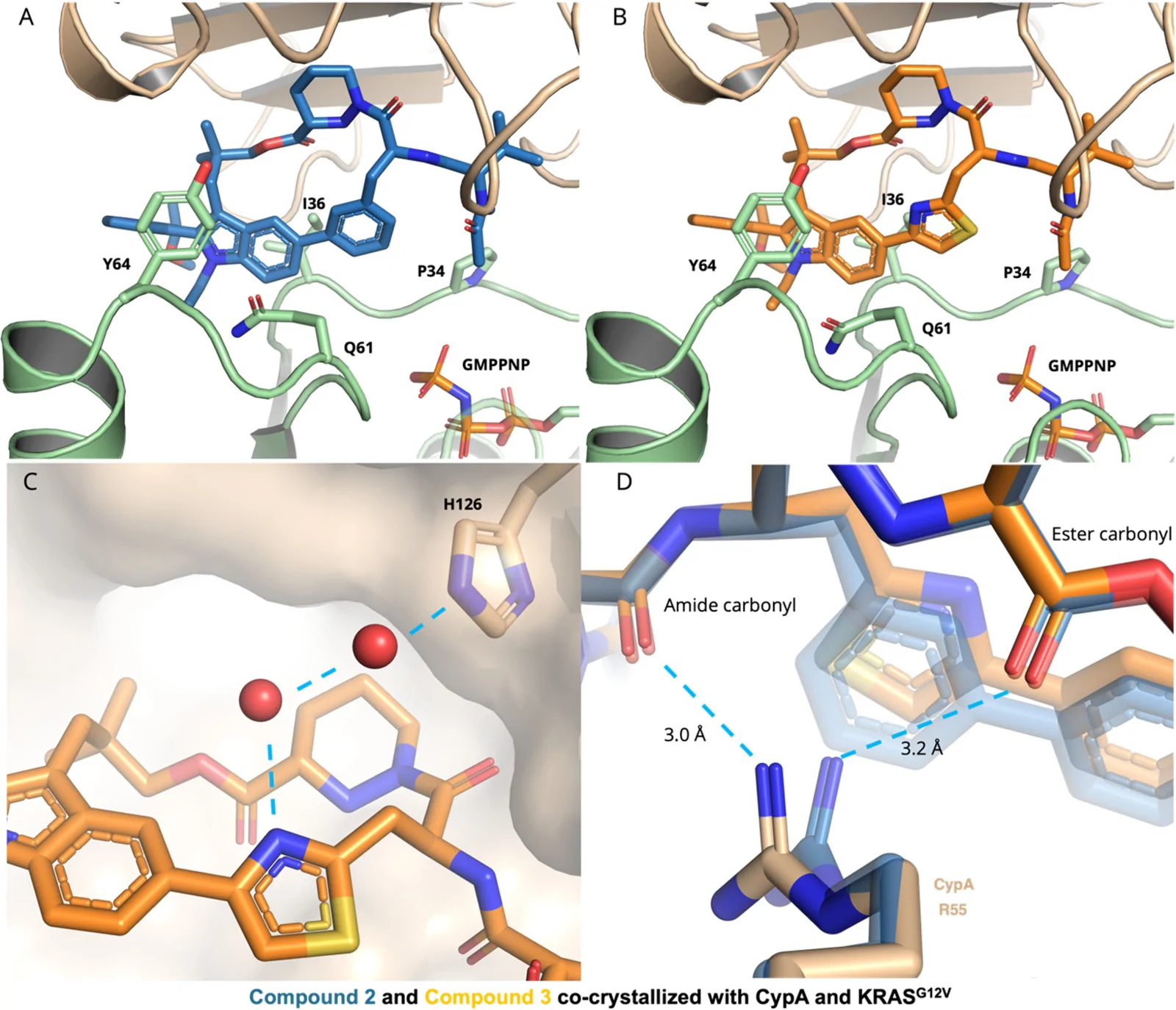
Compound **2** (blue, PDB ID 9BG4) and compound **3** (orange, PDB ID 9BG1) cocrystallized with CypA (wheat) and KRAS^G12V^ (green in A and B, hidden for clarity in C and D). A) and B) Compound **2** and compound **3**, respectively, have a similar binding mode between SWI and SWII. Both compounds make hydrophobic contacts with P34, I36, Q61, and Y64. C) The nitrogen in the thiazole core of compound **3** forms a through-water hydrogen bond to CypA H126. D) R55 of CypA forms a hydrogen bond with the ester carbonyl oxygen of compound **2**. In complex with compound **3** that contains a thiazole moiety, CypA R55 moves toward the amide carbonyl. This move enables the formation of a tighter hydrogen bond, leading to an improvement in K_D1_.

As demonstrated by data in Table 1, the ability of the tri-complex to drive disruption of effector binding for multiple RAS variants is in good correlation with binding affinity to corresponding mutants (K_D2_). Enabled by this observation, we selected the RAS-BRAF RBD disruption assay (IC_50_ values) as the primary biochemical potency assessment to guide the SAR exploration.

Compounds **1**–**3** potently inhibited cell proliferation in multiple cell lines bearing mutant or wild-type RAS while reducing the MW and number of rotatable bonds (Table 1). Encouraged by these observations, we turned our attention to optimization of drug-like properties. Compound **3** was profiled in *in vitro* ADME screening assays and based on the apparent permeability (P_*app*_, A-B), mouse liver microsome stability, solubility data, and oral bioavailability in mouse (Caco-2: 0.2 nm/s; MLM T_1/2_ 6 min; Solubility pH 7.4: 6 μM; m%F = 0.4%) data, we hypothesized that oral bioavailability for this lead compound was limited by a combination of first-pass metabolism, poor absorption, and low aqueous solubility. We first focused on addressing metabolic stability while continuing the exploration of the RHS of the molecule as the primary focus in further rounds of SAR optimization (Table 2). Introduction of the dimethylamino urea side chain in compound **4** was encouraging as it demonstrated that RHS modification can lead to improved aqueous solubility, although the observed mouse liver microsome stability for this analog was still on the lower end. Continued exploration of RHS series (Table 2) and introduction of a carbamate moiety in compound **5** or a cyclic azetidine urea moiety in compound **7** demonstrated improvement in liver microsome stability, although it resulted in lower aqueous solubility. Introduction of a methoxyazetidine urea containing side chain yielded analogue **6** with improved potency and oral bioavailability. Based on X-Ray crystallography data, we concluded that the urea moiety in compound **6** exhibited shape complementarity with RAS Y32 and P34, where formation of these additional van der Waals contacts likely contributed to the >10-fold potency boost observed for both WT and mutant RAS in the RAS-BRAF RBD disruption assay (Figure 6). The observed oral bioavailability (>10%) in rodent for the methoxy azetidine urea derivative compound **6** was likely due to the combination of an improved liver microsome stability and enhanced solubility, despite relatively low apparent permeability (P_*app*, A‑B_) when tested in a Caco-2 system. However, the measurement of apparent permeability *in vitro* could be confounded by the low recovery of compounds in the conventional Caco-2 assay, which is likely due to high nonspecific binding and/or retention of compound in cells, as a function of binding to CypA. Therefore, we applied a P_*app*, A‑B_ cutoff of 2.0 nm/s rather than apparent permeability (P_*app*, A‑B_) values to rank order molecules and advance compounds for *in vivo* rodent PK evaluation.

**Figure 6:**
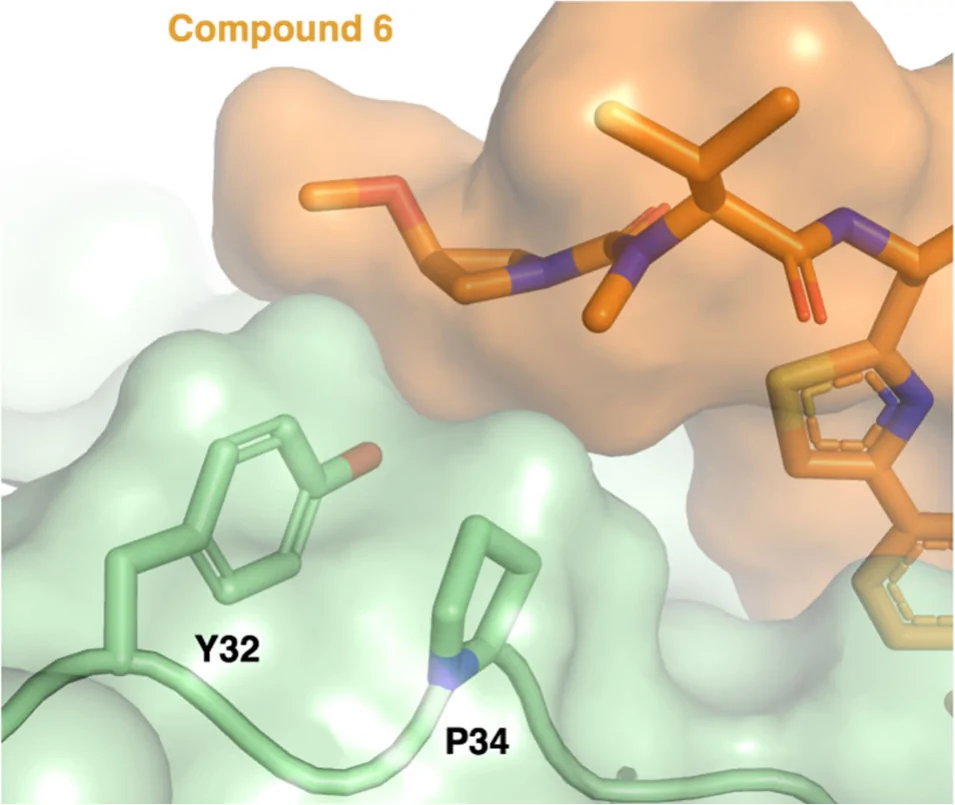
Compound **6** in a tri-complex with GMPPNP bound KRAS^G12V^(ON). The urea moiety (compound **6**) forms a large hydrophobic interface with SWI of RAS at the P34 and Y32 sidechains (PDB ID 9BG7).

**Table 2:**
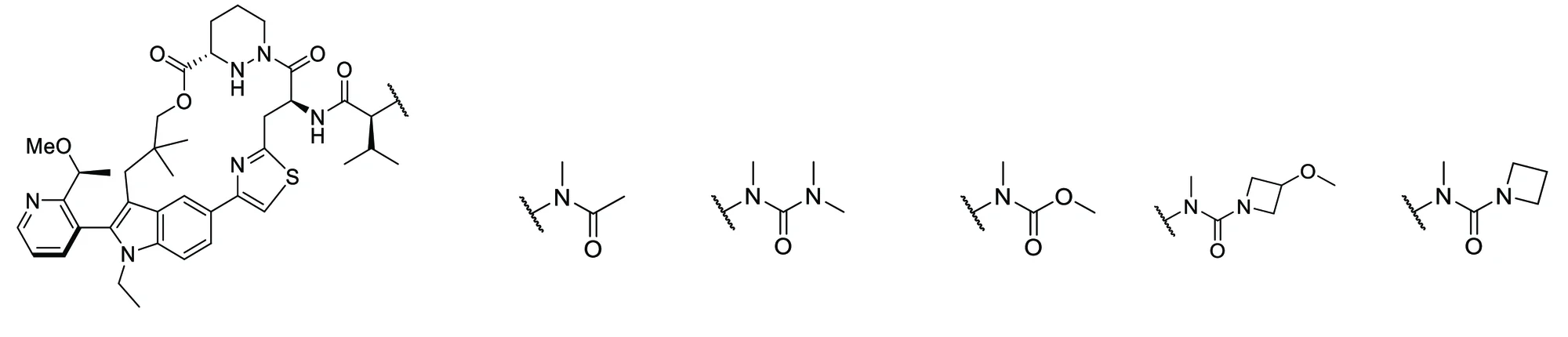

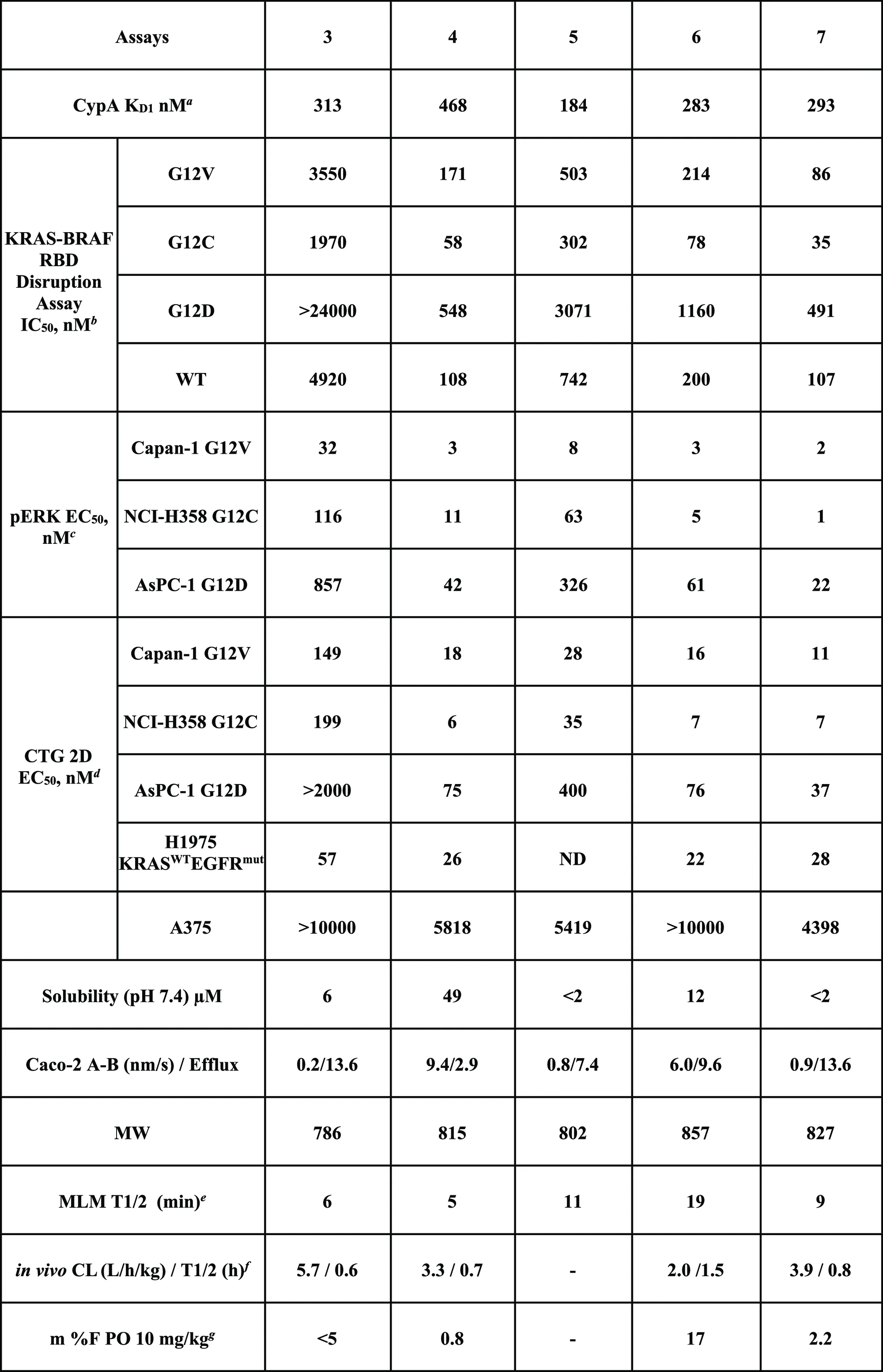
Urea Linker Improves Potency and PK

a CypA binding affinity assessed by surface plasmon resonance (SPR).

b KRAS^G12X or WT^ GMPPNP-bound-BRAF RBD disruption assay, 3 h incubation.

c pERK1/2 immunoassay (AlphaLISA), 4 h incubation.

d Viability assessed at 120 h by CellTiter-Glo luminescence assay (Promega) in Capan-1/NCI-H358/AsPC-1/NCI-H1975/A375 cells.

e Mouse liver microsome stability.

f iv dosing in BALB/c mice (vehicle: 1 mg/kg, 10% DMSO, 20% PEG400, 10% Solutol HS15 in water).

g po dosing in BALB/c mice (vehicle: po, 10 mg/kg, 10% DMSO, 20% PEG400, 10% Solutol, 2% HPMC in 50 mM sodium citrate buffer (pH = 4)).

To investigate whether the improved potency and ADME profile of the urea-linked compounds translated to an improved profile *in vivo*, PK/PD of compound **6** was evaluated in the NCI-H441 (*KRAS* ^*G12V/WT*^, NSCLC) xenograft model (Figure 7). Upon single oral administration of compound **6** at either 100 mg/kg or 200 mg/kg, maximal pathway modulation was observed at 2 h post dose, with 87% and 94% inhibition as measured by relative human *DUSP6* mRNA levels, respectively. While these data demonstrated that a RAS­(ON) multi-selective noncovalent tri-complex inhibitor was sufficient to drive pathway modulation in tumor cells *in vivo*, high oral doses (100 and 200 mg/kg) of compound **6** were required, and the observed pathway modulations were transient. Hence, further optimization of potency and ADME properties of the inhibitor was needed to maintain potent and durable pathway suppression within a dosing interval in xenograft tumors *in vivo*.

**Figure 7:**
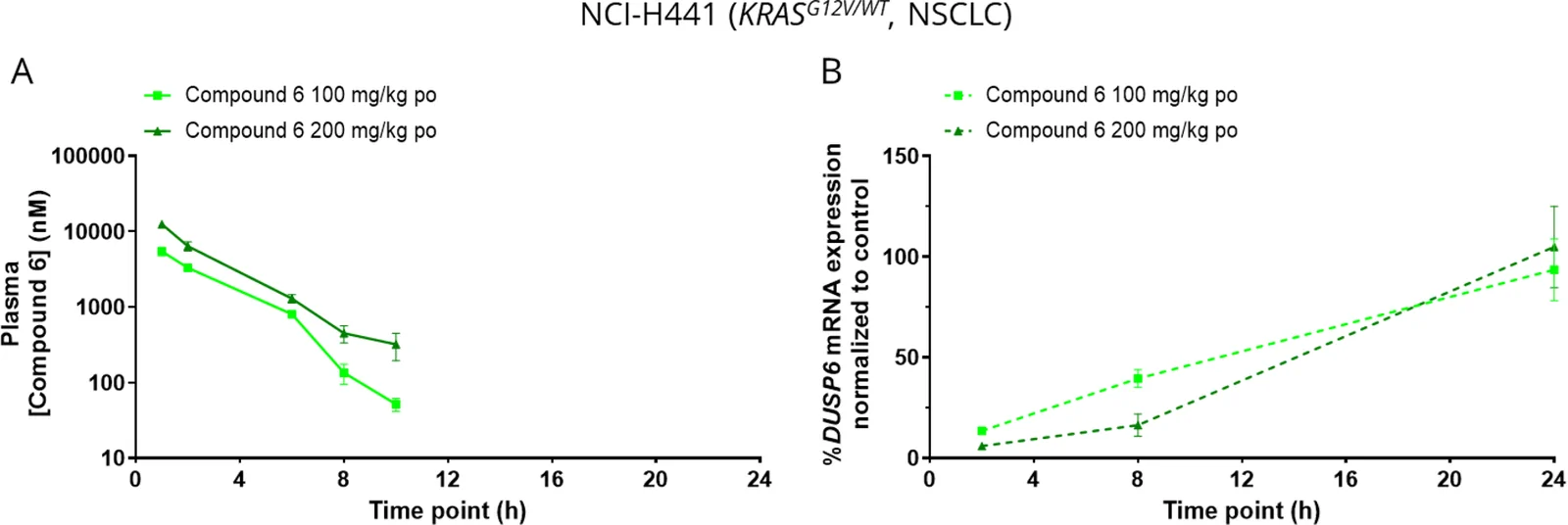
PK/PD profile for compound **6**. NCI-H441 (*KRAS* ^*G12V/WT*^, NSCLC) xenograft tumor-bearing BALB/c nude mice were treated with a single dose of vehicle or compound **6** at 100 or 200 mg/kg. Plasma and tumors were harvested at indicated time points (*n* = 3/time point/dose). A) Plasma PK profiles of compound **6** in tumor-bearing mice. PK profiles are shown as compound **6** concentration in plasma over time. Shades of green represent PK profiles at two tested doses. Values plotted as mean ± SEM. B) PD of compound **6** in NCI-H441 (*KRAS* ^*G12V/WT*^, NSCLC) xenograft tumors, shown as relative change in *DUSP6* mRNA expression. Shades of green represent the two tested doses. Values plotted as mean ± SEM.

Encouraged by initial *in vivo* results, we continued with in depth SAR exploration of the RHS motifs, and further truncation resulted in a series of cyclic amides that exhibited significantly lower MW and a stepwise change in metabolic stability (compounds **8**–**11** MLM T_1/2_ in the range 53–94 min, Table 3). While RHS truncation in compounds **8**–**11** led to a reduced CypA binding affinity (increased K_D1_), likely resulting in the observed reduction in cell potency, this rigidification in combination with lower MW offered a significant improvement in oral bioavailability in mice (compounds **8**–**11** oral bioavailability in rodents in the range of 44%–76%, Table 3). Crystal structures of this series exemplified by compound **10** revealed that the smaller linker allowed for a new, rotated orientation of RAS in the tri-complex compared to the previously described binding pose of compounds **1**, **3**, and **6** (Figure 8A).^24^ In this twisted orientation, the interactions of RAS Y64 in SWII are maintained with both the ligand and CypA W121 (Figure 8B). The flexibility of SWII allows for compression by 3 Å to enable these Y64 interactions. On SWI of RAS, E31 moves toward R69 of CypA, gaining a salt bridge in the twisted orientation (Figure 8Ci). Furthermore, RAS D33 rotates away from CypA K151, which adopts a new rotamer to form a salt bridge with RAS D38 (Figure 8Cii). The rotated orientation allows RAS P34 to make an additional hydrophobic interaction with the thiazole core of compound **10** (Figure 8Ciii). Overall, this twisted binding mode maintains a similar number of key interactions while changing the hydrogen bond donor–acceptor pattern. This new binding mode allows the truncated and lower molecular weight series to retain or even improve activity in the RAS-BRAF RBD disruption assay compared to the urea series, while gaining a significant improvement in exposure profile (Table 3; compound **6** RAS-BRAF RBD disruption KRAS G12V IC_50_= 214, nM; m%F = 17; compound **11** RAS-BRAF disruption KRAS G12V IC_50_ = 84, nM; compound **6** CTG 2D Capan-1 G12V EC_50_ = 16 nM ; compound **11** CTG 2D Capan-1 G12V EC_50_ = 69 nM; m%F = 49). Compounds **8**–**11** did show a slight decrease in cell potency, which is likely driven by the decrease in CypA affinity (increase in the K_D1_) for this series.

**Table 3:**
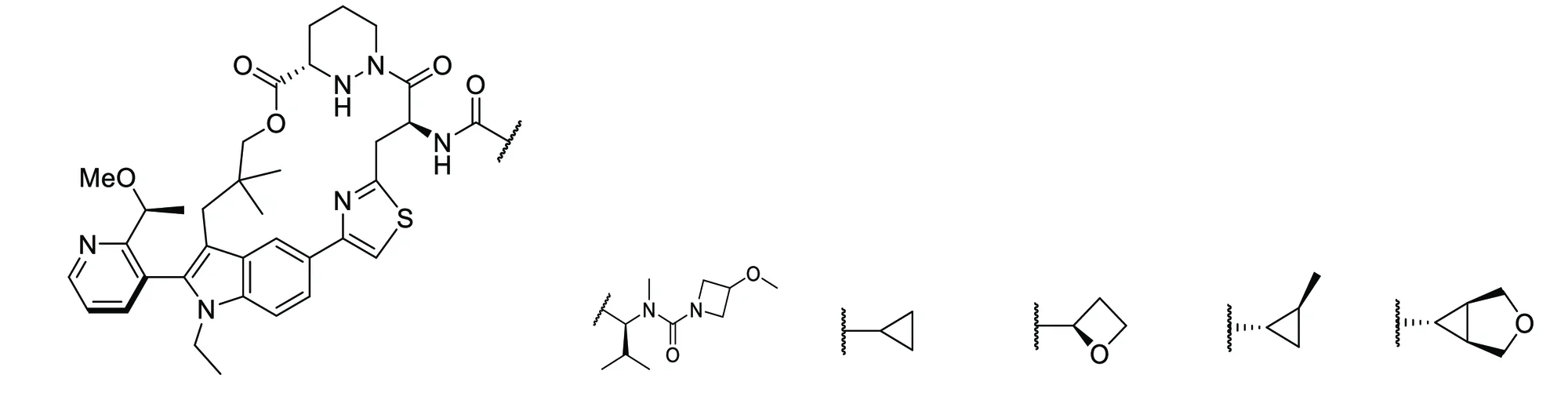

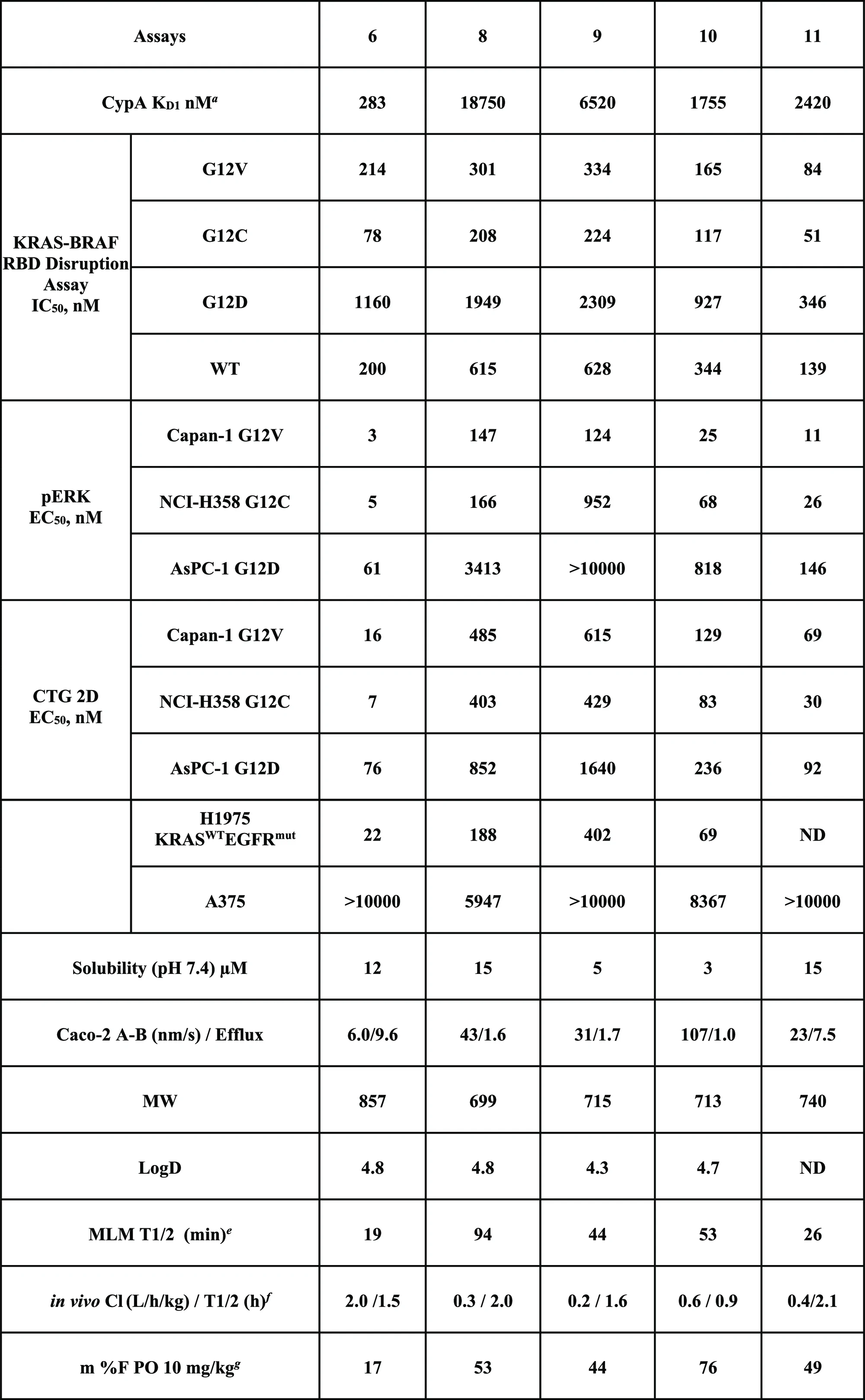
Right Hand Side Chain Optimization

a CypA binding affinity assessed by surface plasmon resonance (SPR).

b KRAS^G12X or WT^GMPPNP-BRAF RBD disruption assay, 3 h incubation.

c pERK1/2 immunoassay (AlphaLISA), 4 h incubation.

d Viability assessed at 120 h by CellTiter-Glo luminescence assay (Promega) in Capan-1/NCI-H358/AsPC-1/NCI-H1975/A375 cells.

e Mouse liver microsome stability.

f iv dosing in BALB/c mice (vehicle: 1 mg/kg, 10% NMP, 10% EtOH, 10% PEG400, 10% Solutol in water).

g po dosing in BALB/c mice (vehicle: po, 10 mg/kg, 10% DMSO, 20% PEG400, 10% Solutol, 2% HPMC in 50 mM sodium citrate buffer (pH = 4)).

**Figure 8:**
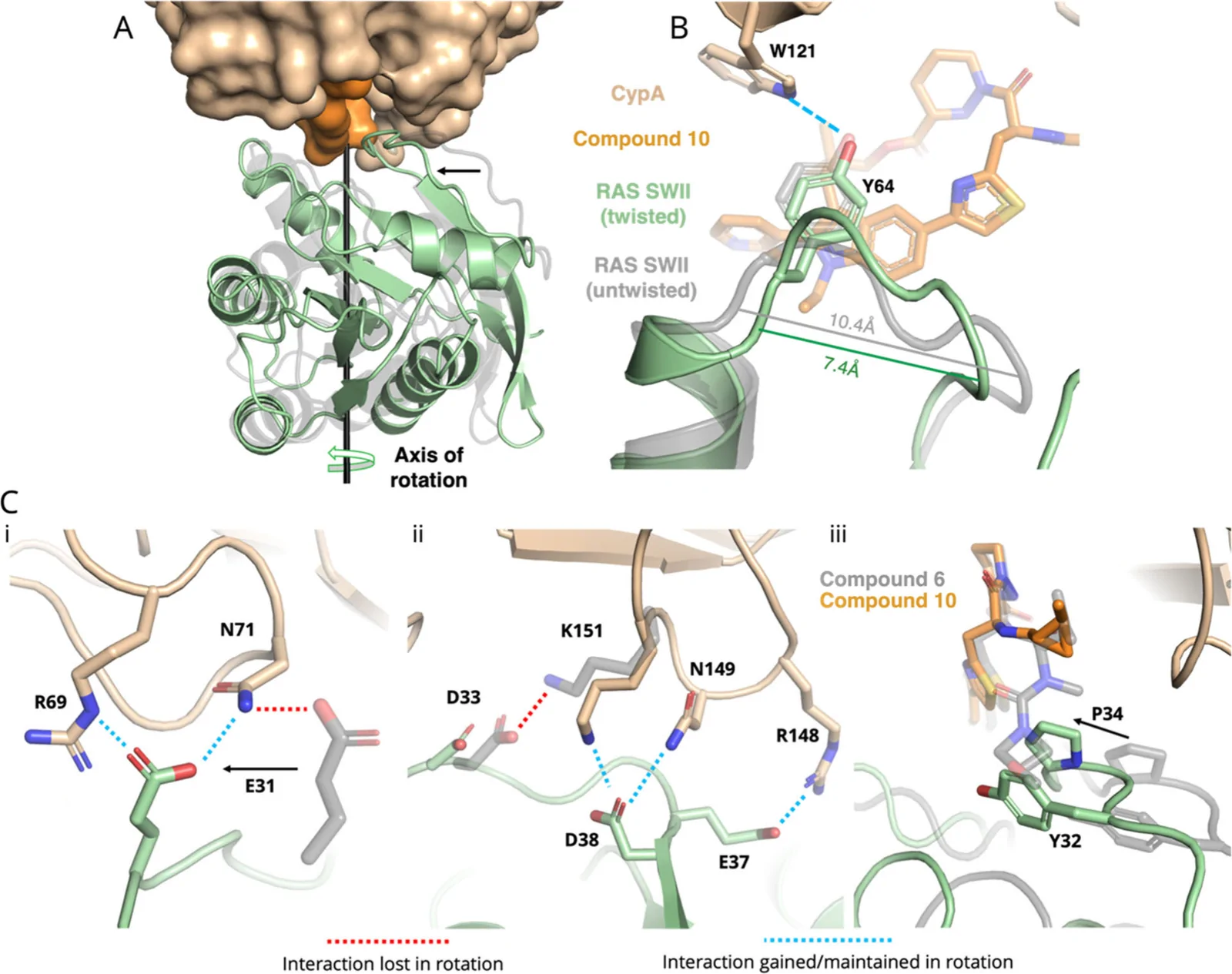
A) Compounds with a truncated linker allow a new tri-complex binding mode. In this mode, RAS is rotated 19° about an axis that lies between SWI and SWII and is adjacent to the indole of the macrocycle. B) Both indole and pyridine of the macrocycle form π-stacking interactions with RAS Y64 while also accepting a hydrogen bond from the CypA W121 side chain. These interactions are maintained in both the original and the twisted orientations. The SWII main chain is compressed by 3 Å, resulting in a new configuration that accommodates the gross rotation of the RAS relative to CypA. C) Specific interactions in SWI: i) In the original orientation (gray), RAS E31 accepts a hydrogen bond from CypA N71 (red dashes). In the twisted orientation, E31 has shifted and is able to form a salt bridge with R69 while accepting a hydrogen bond from the N71 amide nitrogen (blue dashes); ii) RAS D33 forms a salt bridge with CypA K151 (red dashes) in the original orientation. In the twisted orientation, D33 shifts away from K151 and K151 instead forms a salt bridge with D38. N149 and R148 interactions with RAS E37 and D38 are maintained (blue dashes); iii) SWI shifts under the macrocycle side chain, filling open space vacated by the smaller compound in the twisted orientation. In this binding mode, P34 makes interactions with both the thiazole core and cyclopropyl side chain of compound **10** compared to only the urea methyl of compound **6** in the original orientation.

Next, we explored opportunities to further improve potency by focusing on CypA binding (K_D1_) through modifications on the left-hand side (LHS) of the molecule. As demonstrated by the X-ray crystal structure data, the LHS of the molecule interacts with both CypA and SWII regions of RAS and faces the solvent region. Multiple motifs with potential for improving solubility, including piperazine modifications, were explored in combination with the most promising RHS moieties. Introduction of a piperazine moiety on the LHS of the molecule resulted in a significant enhancement in potency and improved aqueous solubility. Representative piperazine-containing analogs are exemplified in Table 4, compounds **12**–**19**. As demonstrated by crystallography data, the piperazine moiety forms a new cation−π interaction with W121 (Figure 9) contributing to an over 20-fold increase in CypA binding affinity (K_D1_ = 55 nM compound **13**) and resulting in a dramatic improvement in potency. Overall, the modification of the SWII moiety, in combination with RHS modifications, resulted in an over 70-fold improvement in cellular potency (CTG 2D Capan-1 EC_50_ G12V = 1.3 nM). The analogs in this series displayed improved metabolic stability and oral exposure, as shown in Table 4. Notably, concentration-dependent partitioning into red blood cells *in vitro* was observed for compounds with a high-affinity binding to CypA (Table 4). For example, for compound **13**, the mouse blood to plasma ratio ranged from 4.2 at 0.1 μM to 1.1 at 10 μM (). Concentration-dependent partitioning behavior was also observed *in vivo* following oral administrations in mice (). High CypA affinity is likely to impact tissue distribution, as CypA is a ubiquitously expressed and abundant intracellular protein in red blood cells, normal tissues, and tumors. Due to observed concentration-dependent blood to plasma partitioning, plasma PK may not be representative of the true systemic exposure to this series of compounds; therefore, further cross-species PK characterization was characterized in whole blood to help rank order compounds.

**Figure 9:**
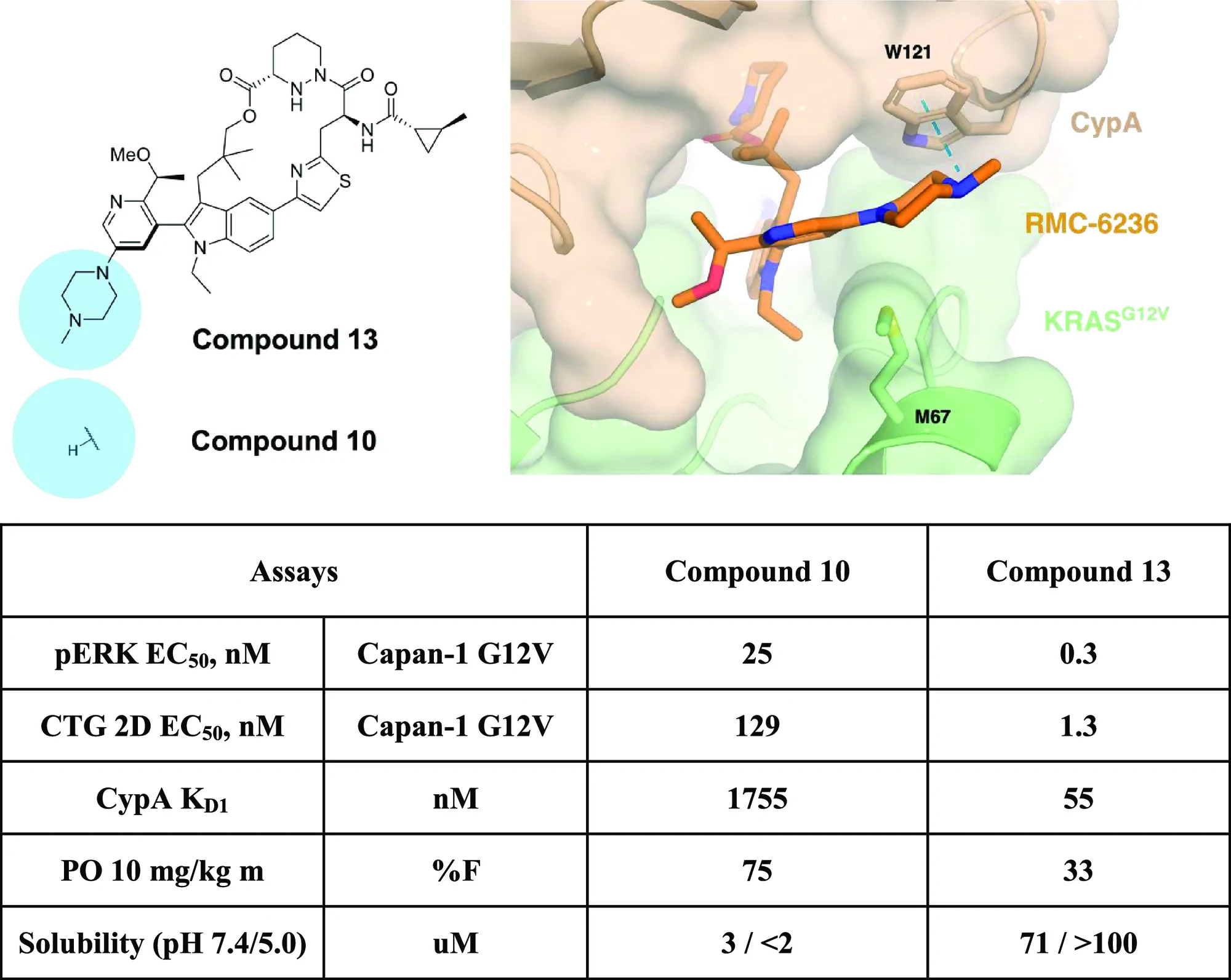
LHS piperazine dramatically improves the potency.

**Table 4:**
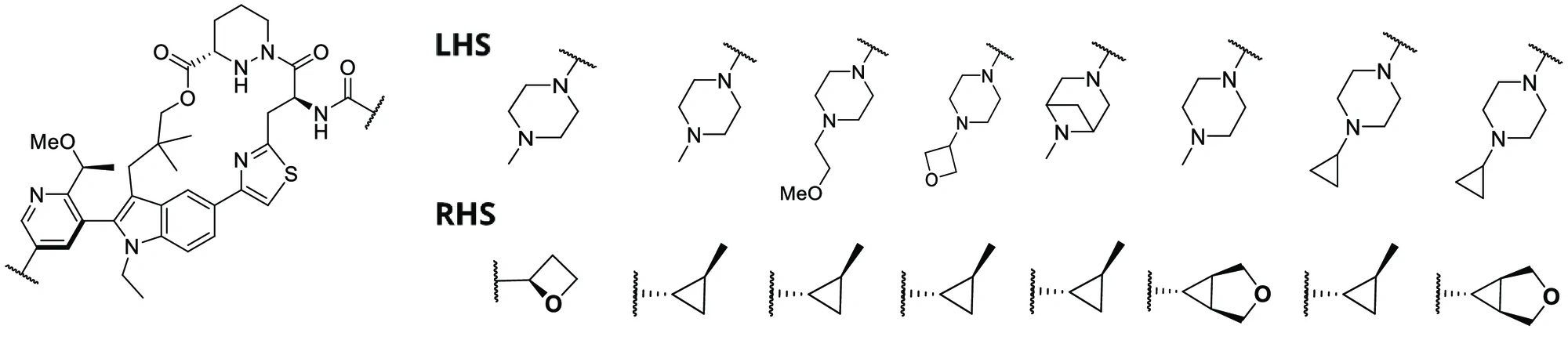

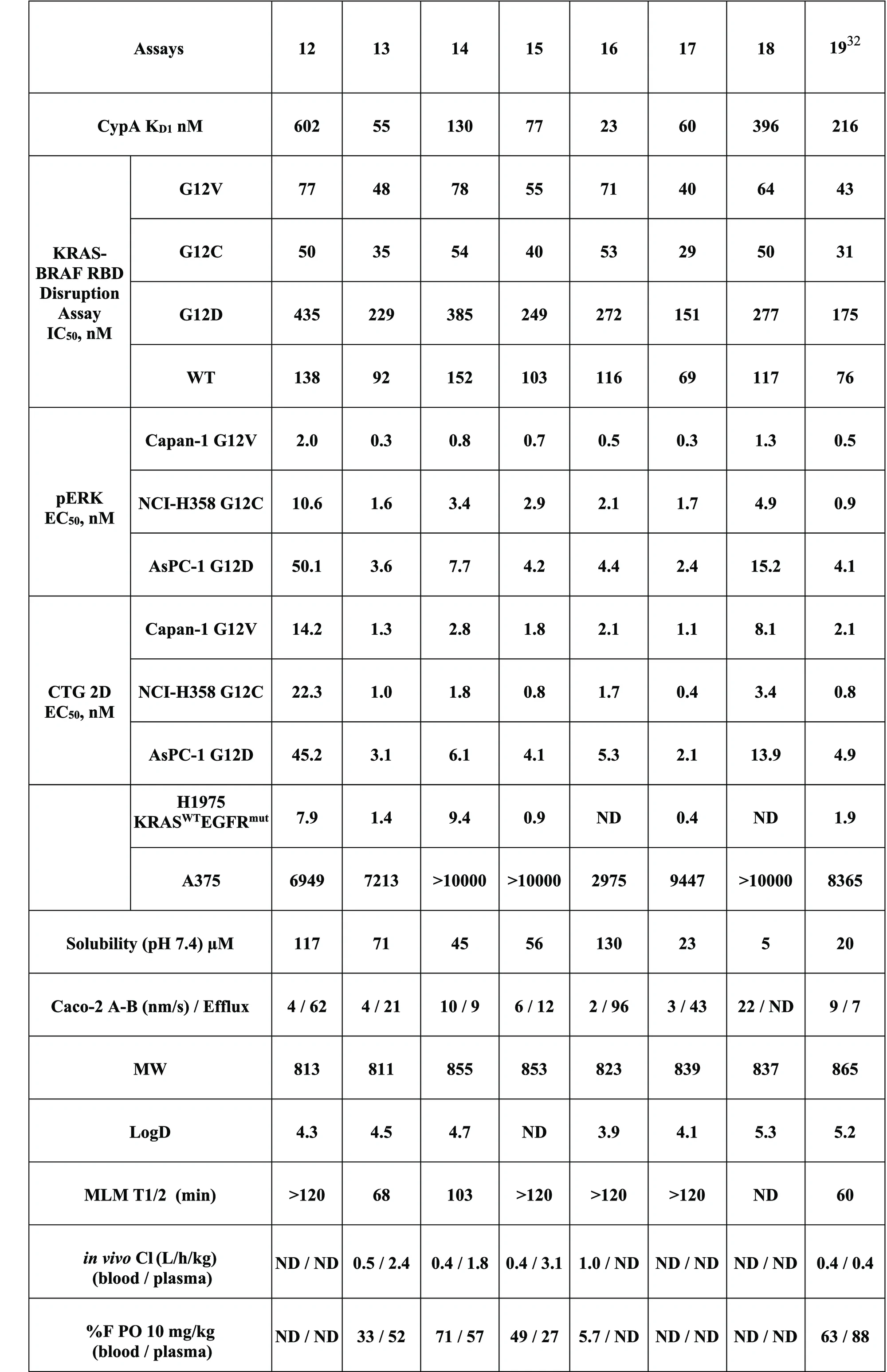
LHS Piperazine Moiety in Combination with Optimized RHS Moiety Dramatically Improves Potency and Drug-like Profile

a CypA binding affinity assessed by surface plasmon resonance (SPR).

b KRAS^G12X or WT^ GMPPNP-bound-BRAF RBD disruption assay, 3 h incubation.

c pERK1/2 immunoassay (AlphaLISA), 4 h incubation.

d Viability assessed at 120 h by CellTiter-Glo luminescence assay (Promega) in Capan-1/NCI-H358/AsPC-1/NCI-H1975/A375 cells.

e Mouse liver microsome stability.

f iv dosing in BALB/c mice (vehicle: 1 mg/kg, 10% NMP, 10% EtOH, 10% PEG400, 10% Solutol in water).

g PO dosing in BALB/c mice (vehicle: po, 10 mg/kg, 10% DMSO, 20% PEG400, 10% Solutol, 2% HPMC in 50 mM sodium citrate buffer (pH = 4)).

Compound **13** (daraxonrasib (RMC-6236)) was selected for advanced characterization based on the combination of cellular potency, solubility, metabolic stability, and *in vivo* PK profile in mice.^18^ Daraxonrasib (RMC-6236) occupies a composite binding pocket between CypA and the SW regions of RAS, similar to previously described tri-complex inhibitors.^32,33^ Daraxonrasib (RMC-6236) makes several contacts with the RAS SWI, forming hydrophobic interactions with conserved residues P34 and I36 (Figure 10a). On SWII (Figure 10b), additional hydrophobic contacts are made with residues A59, Q61, and M67, conserved across multiple mutational variants and WT RAS. Both the indole and pyridine groups of daraxonrasib (RMC-6236) form π–π stacking interactions with RAS Y64 and the daraxonrasib (RMC-6236) thiazole group forms a through-water hydrogen bond to Y64’s hydroxyl moiety.

**Figure 10:**
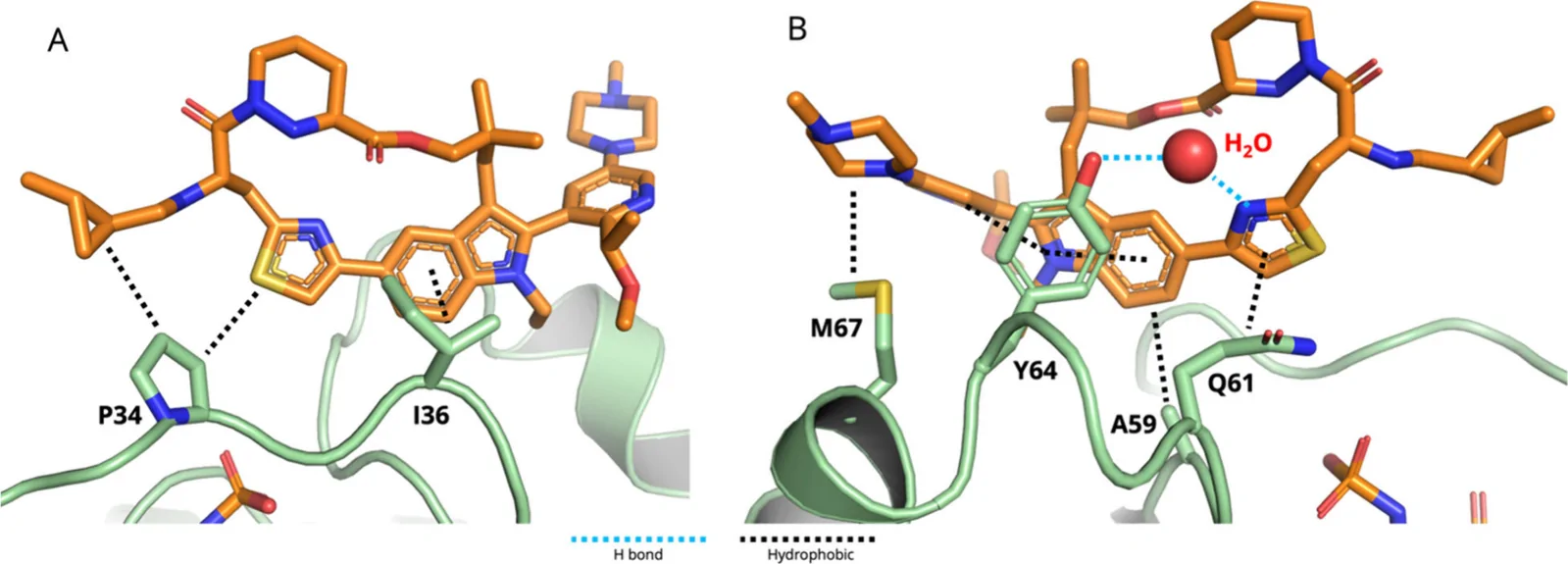
Daraxonrasib (RMC-6236) (orange sticks) bound to the SW regions of human KRAS in the tri-complex (CypA hidden for clarity). A) SWI interactions: the cyclopropyl and thiazole groups of daraxonrasib (RMC-6236) form hydrophobic interactions with KRAS P34 and the indole core of daraxonrasib (RMC-6236) forms hydrophobic interactions with I36. B) SWII interactions: daraxonrasib (RMC-6236) indole core forms hydrophobic interactions with the side chain methyl of KRAS residue A59. The thiazole group of daraxonrasib (RMC-6236) forms hydrophobic interactions with KRAS Q61 and forms a through-water hydrogen bond to Y64. The indole core and pyridine of daraxonrasib (RMC-6236) form π–π stacking interactions with the side chain of RAS Y64. The piperazine group of daraxonrasib (RMC-6236) forms van der Waals interactions with RAS M67 (PDB ID 9BG6).

The groove along the Q61-G12-G13 axis can accommodate bulky RAS mutant side chains, and the vast majority of oncogenic mutations occur at these three positions. The shorter side chain in daraxonrasib (RMC-6236) does not extend toward the P-loop of RAS, leaving the groove containing common oncogenic mutant residues G12, G13, and Q61 unoccupied (Figure 11) and providing a structural rationale for the ability of daraxonrasib (RMC-6236) to bind WT and multiple mutant variants of RAS with similar affinity.^33^

**Figure 11:**
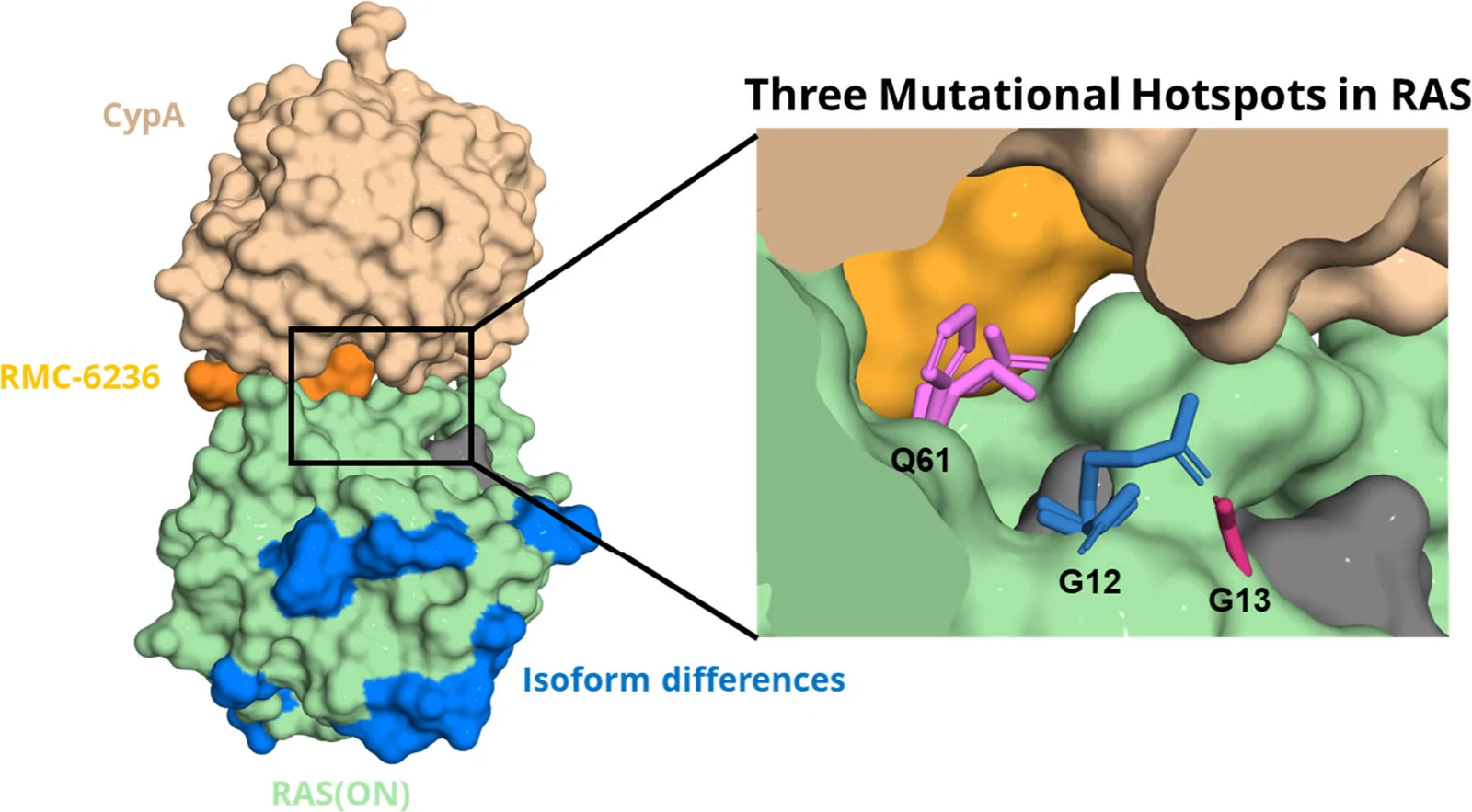
This tri-complex binding mode leaves an unoccupied groove along the Q61-G12-G13 axis. This groove between CypA, daraxonrasib (RMC-6236) , and GMPPNP-bound RAS can accommodate the bulky sidechains of the mutants at the three oncogenic hotspots (PDB IDs 9BG6, 9BG9, 9BGA, 9BGC, 9BG5, 9BGB, 9BG0, 9BG8, 9BG3, and 9BGD).

Additionally, CypA and daraxonrasib (RMC-6236) interact only with the conserved, effector binding lobe of RAS. This binding mode allows daraxonrasib (RMC-6236) to potently bind to the active state of all three H, K, and N RAS isoforms, as the variant residues between the isoforms, shown in blue in Figure 11, lie distant to the binding interface in the tri-complex.

Daraxonrasib (RMC-6236) potency for disruption of BRAF RBD binding to RAS, in the presence of CypA, was nearly identical for wild-type KRAS, NRAS, and HRAS, and varied over approximately a 10-fold range for the different RAS mutants, with the greatest potency for G12C (IC_50_ = 35 nM) and the least potency for G12D (IC_50_ = 229 nM) (Figure 12a). Daraxonrasib (RMC-6236) potently reduced cellular phospho ERK (pERK), a marker of RAS activation, and cellular proliferation in eight cell lines representing a range of RAS variants including wild-type KRAS and G12V, G12C, G12D, G12R, G13D, Q61H, and NRAS Q61R (Figure 12b, c). Daraxonrasib (RMC-6236) exhibited similar potency for pERK inhibition (EC_50_ 1.08–2.61 nM) across these cell lines, with a wider range for inhibition of cellular proliferation (EC_50_ 0.80–27.1 nM).

**Figure 12:**
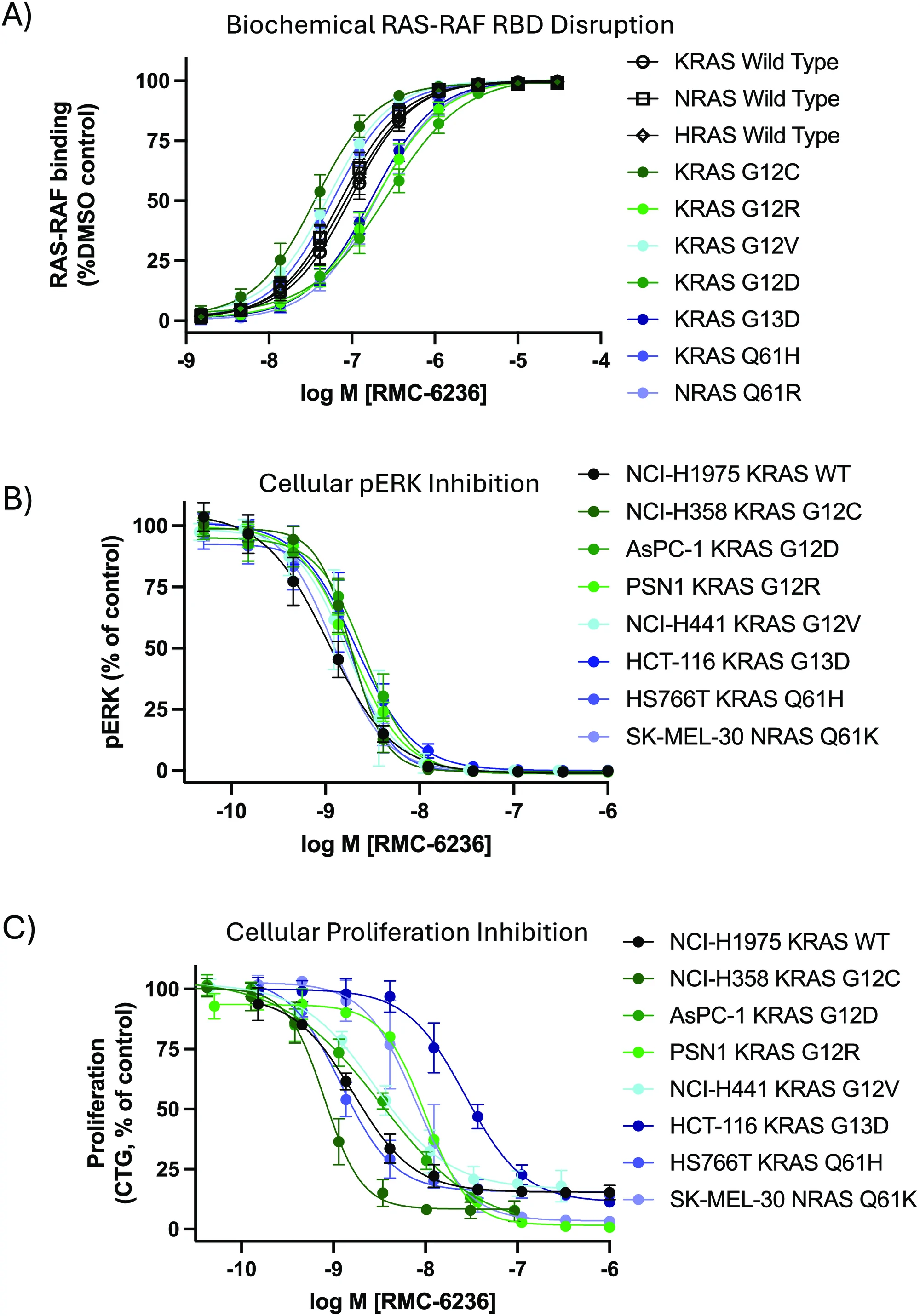
Biochemical and cellular activities of daraxonrasib (RMC-6236). A) Concentration-dependent disruption of interaction between different RAS variants and the RAS binding domain (RBD) of BRAF kinase detected by time-resolved Foerster resonance energy transfer (TR-FRET). B) Inhibition of ERK phosphorylation (pERK) and cellular proliferation. C) in eight cancer cell lines harboring RAS mutants or wild-type RAS.

We then assessed the pharmacokinetic (PK) and pharmacodynamic (PD) profile of daraxonrasib (RMC-6236) in MIA PaCa-2 (*KRAS* ^*G12C/G12C*^, PDAC) xenograft tumor bearing BALB/c nude mice (Figure 13). A single oral administration of daraxonrasib (RMC-6236) at either 3, 10, or 25 mg/kg led to profound dose-dependent suppression of tumor *DUSP6* mRNA levels, with maximal 93%, 95%, and 98% inhibition of *DUSP6* mRNA levels relative to vehicle control achieved at 8 h, respectively. Upon treatment of daraxonrasib (RMC-6236) at 25 mg/kg, MAPK pathway suppression was maintained up to 48 h post single dose, while pathway reactivation was observed in MIA PaCa-2 tumors in lower dose groups. Consistent with the deep and durable RAS signaling modulation, daily daraxonrasib (RMC-6236) treatment resulted in dose-dependent antitumor activity in MIA PaCa-2 (*KRAS* ^*G12C/G12C*^, PDAC) and HPAF-II (*KRAS* ^*G12D/WT*^, PDAC) human tumor xenograft models (Figure 14). Of note, repeated daily administration of daraxonrasib (RMC-6236) at 25 mg/kg was well tolerated as assessed by body weights and could drive deep durable regressions in xenograft tumor models driven by different RAS mutant alleles, e.g., KRAS^G12C^, KRAS^G12D^, and KRAS^G12V^ (Figure 14). Here we are showing a subset of previously described models representative of different tumor types and mutations.^18^

**Figure 13:**
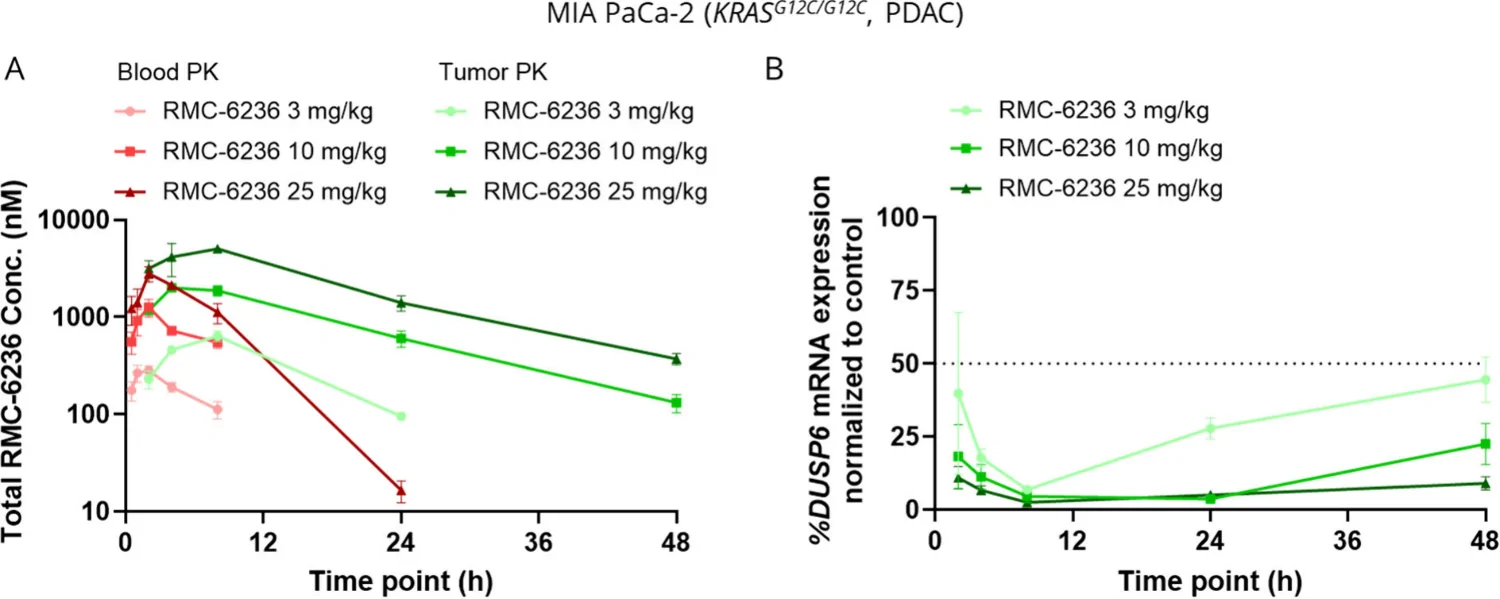
Daraxonrasib (RMC-6236) PK/PD. MIA PaCa-2 (*KRAS* ^*G12C/G12C*^, PDAC) xenograft tumor-bearing BALB/c nude mice were treated with a single dose of vehicle or daraxonrasib (RMC-6236) at 3, 10 or 25 mg/kg. Blood and tumors were harvested at indicated time points (*n* = 3/time point/dose). A) Blood and tumor PK profiles of daraxonrasib (RMC-6236) in MIA PaCa-2 (*KRAS* ^*G12C/G12C*^, PDAC) xenograft tumor-bearing mice. PK profiles are shown as daraxonrasib (RMC-6236) concentration in tumors (green lines) and blood (red lines) over time. Shades of green or red represent the PK profiles at three tested doses. Values plotted as mean ± SEM. B) PD of daraxonrasib (RMC-6236) in MIA PaCa-2 (*KRAS* ^*G12C/G12C*^, PDAC) xenograft tumors, shown as relative change in *DUSP6* mRNA expression. Shades of green represent the three tested doses. Values plotted as mean ± SEM.

**Figure 14:**
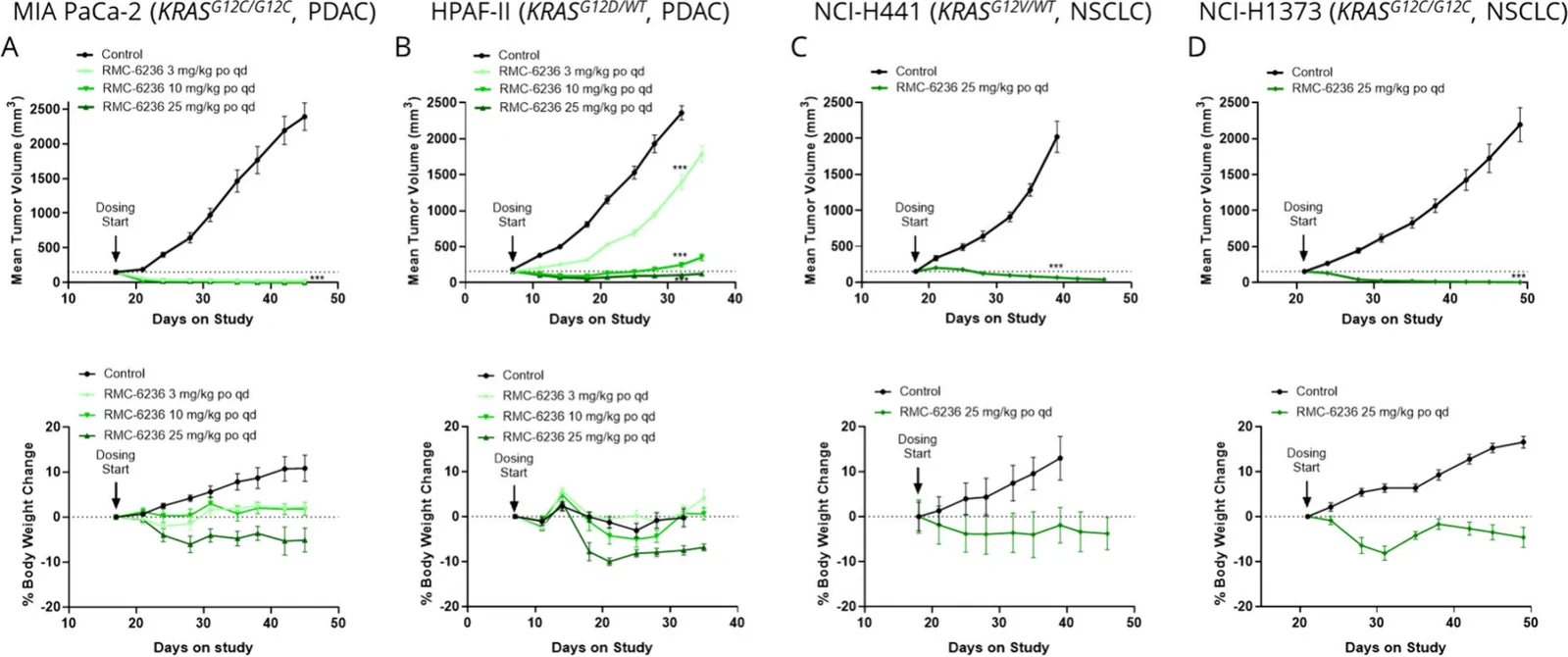
Antitumor activity of daraxonrasib (RMC-6236) in subcutaneous xenograft models of A) MIA PaCa-2 (*KRAS* ^*G12C/G12C*^, PDAC) (*n* = 8 per group), B) HPAF-II (*KRAS* ^*G12D/WT*^, PDAC) (*n* = 9 per group), C) NCI-H441 (*KRAS* ^*G12V/WT*^, NSCLC) (*n* = 6 per group), and D) NCI-H1373 (*KRAS* ^*G12C/G12C*^, NSCLC) (*n* = 8 per group). Tumor-bearing mice were treated with vehicle or daraxonrasib (RMC-6236) at indicated doses for 28 days, and mean tumor volumes of each group were plotted over the course of treatment. Vehicle control and daraxonrasib (RMC-6236) groups were compared by 2-way repeated measures ANOVA on the last measurement day of the vehicle group (***p < 0.001). Dotted line indicates the initial average tumor volume. % Body weight change from baseline were also plotted over the course of treatment. Dotted line indicates the initial average body weight of each group. Error bars indicate SEM.

In cross-species PK assessment, daraxonrasib (RMC-6236) demonstrated low to moderate blood clearance and moderate volume of distribution at steady state following single dose IV administration and moderate oral bioavailability (F% = 24%–33%) across mice, rats, dogs, and cynomolgus monkeys (Table 5). In mice, daraxonrasib (RMC-6236) displayed approximately linear whole blood PK at doses of 10 to 100 mg/kg. Based on favorable *in vitro* and *in vivo* ADME/PK properties, daraxonrasib (RMC-6236) is predicted to be bioavailable for clinical use following daily oral administration.^18^

**Table 5:** RMC-6236 Main Blood PK Parameters in Preclinical Species

	Main blood PK parameters in preclinical species				
Route	Parameters	Mouse	Rat	Dog	Monkey
IV^a^	CL (L/h/kg)	0.5	1.3	0.4	0.5
	*V*_ss_ (L/kg)	1.3	1.9	2.1	2.9
	*t*_1/2_ (h)	2.2	1.7	6.1	6.2
PO^b^	*T*_max_ (h)	1.7	4.0	0.7	3.7
	*t*_1/2_ (h)	2.4	2.8	5.3	4.6
	F%	33	27	33	24

a IV was dosed at 1 mg/kg in all preclinical species.

b PO was dosed at 10 mg/kg (mouse), 60 mg/kg (rat), 5 mg/kg (dog), and 2.5 mg/kg (monkey). Blood samples were collected for bioanalysis.

Daraxonrasib (RMC-6236) was further characterized in several *in vitro* safety assays. daraxonrasib (RMC-6236) is not a potent inhibitor of human Ether-à-go-go-Related Gene (hERG), human L-type calcium channel (hCav1.2) or human sodium channel (hNav1.5), with IC_50_ values of 8.9, >30 and >30 μM, respectively. RMC-6236 showed low risk for off-target activities in an *in vitro* safety pharmacology panel that included GPCRs, enzymes, ion channels, nuclear hormone receptors, and monoamine transporters. Additionally, daraxonrasib (RMC-6236) was not mutagenic and was not genotoxic in the *in vitro* bacterial reverse (Ames) mutation assay and the *in vitro* micronucleus assay using human peripheral blood lymphocytes in the absence or in the presence of mammalian microsomal enzymes (rat S9 mix).

Based on compelling *in vitro* and *in vivo* activity and pharmacological and safety profiles, daraxonrasib (RMC-6236) was selected as a RAS­(ON) multi-selective development candidate and advanced to clinical evaluation.^18^

## Conclusions

Here we describe the structure-enabled journey that led to the discovery of the development candidate daraxonrasib (RMC-6236), an orally bioavailable, RAS­(ON) multi-selective tri-complex inhibitor. This bRo5 macrocyclic molecule is a potent and direct inhibitor of multiple RAS mutants and wild-type isoforms. To target RAS directly we deployed an innovative technology based on tri-complex formation with a small molecule inhibitor, an abundant chaperone protein CypA, and the target protein RAS, leading to formation of a unique composite binding pocket.

With early lead compound **1**, we confirmed that it is possible to achieve disruption of the downstream activity of RAS with a reversible inhibitor and deployed an SAR campaign with a focus on interactions with residues conserved between mutant and WT RAS isoforms to achieve broad-spectrum RAS isoform activity. Continuous optimization of potency (K_D1_ and K_D2_), and concurrent enhancement of drug-like properties led to a potent and orally bioavailable bRo5 macrocyclic series. In this multiparameter optimization, we went through several key milestones. First, the identification of a thiazole central ring enabled significant enhancement of CypA binding, translating into improved cellular potency. Next, the introduction of a rigid methyl cyclopropyl RHS moiety led to an enhancement in metabolic stability and improved oral bioavailability in mice. Additionally, this change resulted in a new binding pose with a twisted orientation, ultimately driving enhancements in SWII interactions. Finally, the introduction of a methyl piperazine on the LHS of the molecule enabled a step change in cellular potency via cation−π interaction with W121 of CypA driving K_D1_ enhancement, and in addition, led to improved aqueous solubility and metabolic stability, major factors contributing to good oral bioavailability. The resulting development candidate daraxonrasib (RMC-6236) demonstrated high metabolic stability *in vitro*, low to moderate metabolic clearance *in vivo*, good oral bioavailability across multiple species, and a low-risk profile in hERG, off-target safety panel, and *in vitro* genotoxicity assays.

Daraxonrasib (RMC-6236) suppressed pERK phosphorylation and cell growth in multiple human RAS-addicted cancer cell lines and demonstrated a high selectivity over RAS-independent tumor cells *in vitro*. A single oral dose of daraxonrasib (RMC-6236) induced dose-dependent, deep, and durable suppression of RAS pathway activation up to 48 h in preclinical xenograft models of RAS mutant cancers *in vivo*. In representative xenograft models of RAS mutant cancers, daily dosing of daraxonrasib (RMC-6236) drove profound tumor regressions at tolerable doses across different tumor types, including PDAC, NSCLC, and CRC.^18^ Previously, we have also demonstrated that, consistent with *in vitro* data, PDAC and NSCLC xenograft models harboring KRAS glycine-12 substitutions (KRAS^G12X^), a somewhat higher response rate and more durable response, consistent with RAS oncogene addiction in these models.^18^

Daraxonrasib (RMC-6236) is a potent, oral, noncovalent, RAS­(ON) multi-selective tri-complex inhibitor. Daraxonrasib (RMC-6236) has the potential to address a large unmet medical need targeting multiple RAS mutants and to overcome clinically observed resistance mechanisms for KRAS^G12C^(OFF) inhibitors. Daraxonrasib (RMC-6236) is currently in clinical evaluation in patients with RAS mutant advanced solid tumors (NCT05379985; NCT06040541; NCT06162221; NCT06445062; NCT06128551).

## Experimental Section

### General Synthetic Procedures

All final compounds were purified to ≥95% purity by either high-performance liquid chromatography (HPLC) or supercritical fluid chromatography (SFC) and were characterized by proton NMR, carbon NMR, and high-resolution mass spectrometry as described below. All materials were obtained from commercial suppliers and used without further purification, unless otherwise noted. Mass spectrometry data collection took place with a Shimadzu LCMS-2020, an Agilent 1260LC-6120/6125MSD, a Shimadzu LCMS-2010EV, or a Waters Acquity UPLC, with either a QDa detector or an SQ Detector 2. Samples were injected in their liquid phase onto a C-18 reverse phase. The compounds were eluted from the column by using an acetonitrile gradient and fed into the mass analyzer. Initial data analysis took place with either Agilent ChemStation, Shimadzu LabSolutions, or Waters MassLynx. NMR data was collected with either a Bruker AVANCE III HD 400 MHz, a Bruker Ascend 500 MHz instrument, or a Varian 400 MHz, and the raw data was analyzed with either TopSpin or Mestrelab Mnova. For clarity, yields have been rounded to the nearest whole number, and ^13^C NMR data have been rounded to two decimal points.

### Synthesis of Compound 13 (**Daraxonrasib (RMC-6236)** )

#### (S)-4-(5-Bromo-6-(1-methoxyethyl)­pyridin-3-yl)­piperazine-1-carboxylate (S10-1)

Into a 3L 3-necked round-bottom flask purged and maintained with an inert atmosphere of argon was placed 3-bromo-5-iodo-2-[(1*S*)-1-methoxyethyl]­pyridine (147 g, 429.8 mmol) benzyl piperazine-1-carboxylate (94.69 g, 429.8 mmol), Pd­(OAc)_2_ (4.83 g, 21.4 mmol), BINAP (5.35 g, 8.6 mmol), Cs_2_CO_3_ (350.14 g, 1074.6 mmol), and toluene (1 L). The resulting solution was stirred overnight at 100 °C in an oil bath. The reaction mixture was cooled to room temperature after completion. The resulting mixture was concentrated under reduced pressure. The residue was purified by silica gel column chromatography with EtOAc/hexane (1:1). Removal of solvent under reduced pressure gave benzyl (*S*)-4-(5-bromo-6-(1-methoxyethyl)­pyridin-3-yl)­piperazine-1-carboxylate (135 g, 65% yield) as a solid. ^1^H NMR (400 MHz, DMSO-*d*_6_) δ: 8.33 (d, *J* = 2.5 Hz, 1H), 7.60 (d, *J* = 2.5 Hz, 1H), 7.42–7.30 (m, 5H), 5.12 (s, 2H), 4.75 (q, *J* = 6.4 Hz, 1H), 3.55 (t, *J* = 4.8 Hz, 4H), 3.29 (t, *J* = 5.2 Hz, 4H), 1.37 (d, *J* = 6.4 Hz, 3H). ^13^C NMR (101 MHz, chloroform-*d*) δ: 155.03, 147.07, 146.23, 136.34, 133.11, 128.66, 128.55, 128.18, 127.91, 120.82, 76.30, 67.43, 57.02, 47.27, 43.03, 20.05. LCMS (ESI): *m*/*z* [M+H] calcd for C_20_H_24_BrN_3_O_3_ 433.1; found 434.1.

#### (S)-4-(6-(1-Methoxyethyl)-5-(4,4,5,5-tetramethyl-1,3,2-dioxaborolan-2-yl)­pyridin-3-yl)­piperazine-1-carboxylate (S10-2)

Into a 3-L 3-necked round-bottom flask purged and maintained with an inert atmosphere of argon was placed benzyl 4-[5-bromo-6-[(1*S*)-1-methoxyethyl]­pyridin-3-yl]­piperazine-1-carboxylate (135 g, 310.8 mmol), bis­(pinacolato)­diboron (86.82 g, 341.9 mmol), Pd­(dppf)­Cl_2_ (22.74 g, 31.0 mmol), KOAc (76.26 g, 777.5 mmol), and toluene (1 L). The resulting solution was stirred for 2 days at 90 °C in an oil bath. The reaction mixture was cooled to room temperature. The resulting mixture was concentrated under reduced pressure. The residue was purified by an alumina column with EtOAc/hexane (1:3). Removal of solvent under reduced pressure gave benzyl (*S*)-4-(6-(1-methoxyethyl)-5-(4,4,5,5-tetramethyl-1,3,2-dioxaborolan-2-yl)­pyridin-3-yl)­piperazine-1-carboxylate (167 g, crude) as a solid. ^1^H NMR (400 MHz, DMSO-*d*_6_) δ: 8.27 (d, *J* = 3.0 Hz, 1H), 7.98 (s, 1H), 7.43–7.29 (m, 6H), 5.12 (s, 2H), 4.56 (dq, *J* = 31.3, 6.5 Hz, 1H), 3.55 (q, *J* = 10.2, 7.5 Hz, 4H), 3.17 (q, *J* = 5.2, 3.9 Hz, 5H), 3.09 (s, 2H), 1.38 (dd, *J* = 12.4, 6.5 Hz, 3H), 1.31 (s, 6H). ^13^C NMR (101 MHz, DMSO-*d*_6_) δ: 154.88, 145.10, 138.94, 137.31, 128.88, 128.86, 128.32, 128.07, 128.03, 84.11, 80.26, 79.15, 66.81, 56.31, 56.25, 48.49, 48.30, 25.21, 24.96, 21.34, 20.73. LCMS (ESI): *m*/*z* [M+H] calcd for C_26_H_36_BN_3_O_5_ 481.3; found 482.1.

#### Benzyl (S)-4-(5-(5-bromo-3-(3-((tert-butyldiphenylsilyl)­oxy)-2,2-dimethylpropyl)-1H-indol-2-yl)-6-(1-methoxyethyl)­pyridin-3-yl)­piperazine-1-carboxylate (S10-3)

Into a 3-L 3-necked round-bottom flask purged and maintained with an inert atmosphere of argon was placed (*S*)-4-(6-(1-methoxyethyl)-5-(4,4,5,5-tetramethyl-1,3,2-dioxaborolan-2-yl)­pyridin-3-yl)­piperazine-1-carboxylate (167 g, 346.9 mmol), 5-bromo-3-[3-[(*tert*-butyldiphenylsilyl)­oxy]-2,2-dimethylpropyl]-2-iodo-1H-indole (224.27 g, 346.9 mmol), Pd­(dppf)­Cl_2_ (25.38 g, 34.6 mmol), dioxane (600 mL), H_2_O (200 mL), K_3_PO_4_ (184.09 g, 867.2 mmol), and toluene (200 mL). The resulting solution was stirred overnight at 70 °C in an oil bath. The reaction mixture was cooled to room temperature and concentrated under reduced pressure. The residue was purified by flash chromatography with EtOAc/hexane (1:1). Removal of solvent under reduced pressure gave benzyl (*S*)-4-(5-(5-bromo-3-(3-((*tert*-butyldiphenylsilyl)­oxy)-2,2-dimethylpropyl)-1*H*-indol-2-yl)-6-(1-methoxyethyl)­pyridin-3-yl)­piperazine-1-carboxylate (146 g, 48% yield) as a solid. ^1^H NMR (400 MHz, DMSO-*d*_6_) δ: 11.32 (s, 1H), 8.43 (d, *J* = 2.8 Hz, 1H), 7.77 (d, *J* = 1.9 Hz, 1H), 7.57–7.47 (m, 4H), 7.46–7.29 (m, 12H), 7.25 (d, *J* = 2.9 Hz, 1H), 7.21 (dd, *J* = 8.6, 1.9 Hz, 1H), 5.12 (s, 2H), 4.10 (q, *J* = 6.2 Hz, 1H), 3.52 (t, *J* = 5.2 Hz, 4H), 3.18 (d, *J* = 5.7 Hz, 4H), 2.87 (s, 3H), 2.51 (m, 2H), 1.44–1.20 (m, 4H), 0.96 (s, 9H), 0.65 (d, *J* = 7.5 Hz, 6H). ^13^C NMR (101 MHz, chloroform-*d*) δ: 149.20, 137.38, 136.44, 135.73, 135.71, 134.19, 133.83, 131.64, 129.64, 129.10, 128.63, 128.28, 128.11, 127.67, 125.82, 125.03, 122.67, 112.43, 111.48, 73.22, 67.49, 55.32, 48.28, 39.09, 32.57, 27.12, 24.45, 24.32, 19.51. LCMS (ESI): *m*/*z* [M+H] calcd for C_49_H_57_BrN_4_O_4_Si 872.3; found 873.3.

#### Benzyl (S)-4-(5-(5-bromo-3-(3-((tert-butyldiphenylsilyl)­oxy)-2,2-dimethylpropyl)-1-ethyl-1H-indol-2-yl)-6-(1-methoxyethyl)­pyridin-3-yl)­piperazine-1-carboxylate (S10-4)

To a stirred mixture of benzyl (*S*)-4-(5-(5-bromo-3-(3-((*tert*-butyldiphenylsilyl)­oxy)-2,2-dimethylpropyl)-1*H*-indol-2-yl)-6-(1-methoxyethyl)­pyridin-3-yl)­piperazine-1-carboxylate (146 g, 167.0 mmol) and Cs_2_CO_3_ (163.28 g, 501.1 mmol) in DMF (1200 mL) was added C_2_H_5_I (52.11 g, 334.0 mmol) in portions at 0 °C under a nitrogen atmosphere. The final reaction mixture was stirred at room temperature for 12 h. The resulting mixture was diluted with EtOAc (1 L) and washed with brine (3 × 1.5 L). The organic layers were dried in anhydrous Na_2_SO_4_. After filtration, the filtrate was concentrated under reduced pressure to give benzyl (*S*)-4-(5-(5-bromo-3-(3-((*tert*-butyldiphenylsilyl)­oxy)-2,2-dimethylpropyl)-1-ethyl-1*H*-indol-2-yl)-6-(1-methoxyethyl)­pyridin-3-yl)­piperazine-1-carboxylate (143 g, crude) as a solid that was used directly for next step without further purification. ^1^H NMR (400 MHz, DMSO-*d*_6_) δ: 8.48 (dd, *J* = 4.4, 2.8 Hz, 1H), 7.81 (dd, *J* = 4.2, 1.9 Hz, 1H), 7.59–7.46 (m, 5H), 7.45–7.34 (m, 10H), 7.33–7.22 (m, 3H), 5.13 (s, 2H), 4.12–3.63 (m, 3H), 3.52 (s, 4H), 3.42 (t, *J* = 9.2 Hz, 1H), 3.37 (s, 3H), 3.28–3.08 (m, 5H), 2.98 (s, 1H), 2.81 (s, 2H), 1.34 (d, *J* = 6.2 Hz, 2H), 1.21 (d, *J* = 6.3 Hz, 1H), 1.14 (t, *J* = 7.1 Hz, 2H), 1.07 (t, *J* = 7.1 Hz, 1H), 0.95 (d, *J* = 9.0 Hz, 9H), 0.77 (s, 1H), 0.69 (d, *J* = 3.9 Hz, 3H), 0.63 (s, 2H). ^13^C NMR (101 MHz, DMSO-*d*_6_) δ: 154.81, 149.77, 145.24, 138.60, 137.24, 137.16, 136.69, 135.58, 135.52, 134.64, 133.48, 130.88, 130.12, 128.81, 128.29, 128.15, 128.08, 128.06, 126.01, 124.13, 122.48, 112.25, 112.12, 110.55, 75.32, 66.83, 56.04, 55.75, 47.87, 27.15, 27.13, 25.17, 25.03, 24.75, 19.37, 19.35, 19.03, 17.91, 15.63, 15.36. LCMS (ESI): *m*/*z* [M+H] calcd for C_51_H_61_BrN_4_O_4_Si 900.4; found 901.4.

#### Benzyl (S)-4-(5-(5-bromo-1-ethyl-3-(3-hydroxy-2,2-dimethylpropyl)-1H-indol-2-yl)-6-(1-methoxyethyl)­pyridin-3-yl)­piperazine-1-carboxylate (S10-5)

To a stirred mixture of benzyl benzyl (*S*)-4-(5-(5-bromo-3-(3-((*tert*-butyldiphenylsilyl)­oxy)-2,2-dimethylpropyl)-1-ethyl-1*H*-indol-2-yl)-6-(1-methoxyethyl)­pyridin-3-yl)­piperazine-1-carboxylate (143 g, 158.5 mmol) in DMF (1250 mL) was added CsF (72.24 g, 475.5 mmol). Then the reaction mixture was stirred at 60 °C for 2 days under a nitrogen atmosphere. The resulting mixture was diluted with EtOAc (1 L) and washed with brine (3 × 1 L). Then the organic phase was concentrated under a reduced pressure. The residue was purified by silica gel column chromatography and eluted with Pet. ether/EtOAc (1:3) to afford two atropisomers of benzyl (*S*)-4-(5-(5-bromo-1-ethyl-3-(3-hydroxy-2,2-dimethylpropyl)-1*H*-indol-2-yl)-6-(1-methoxyethyl)­pyridin-3-yl)­piperazine-1-carboxylate **A** (38 g, 36% yield, RT = 1.677 min in 3 min LCMS­(0.1% FA)) and **B** (34 g, 34% yield, RT = 1.578 min in 3 min LCMS­(0.1% FA)) both as solids. ^1^H NMR (400 MHz, DMSO-*d*_6_) δ: 8.47 (d, *J* = 2.8 Hz, 1H), 7.90 (d, *J* = 1.9 Hz, 1H), 7.55–7.42 (m, 2H), 7.41–7.24 (m, 6H), 5.12 (s, 2H), 4.11–4.01 (m, 1H), 3.86 (m, 1H), 3.56 (d, *J* = 6.2 Hz, 4H), 3.33 (q, *J* = 5.0 Hz, 4H), 3.06 (d, *J* = 10.3 Hz, 1H), 2.98 (d, *J* = 10.3 Hz, 1H), 2.89 (s, 3H), 2.66 (d, *J* = 14.0 Hz, 1H), 2.51 (m, 1H), 2.15 (d, *J* = 14.1 Hz, 1H), 1.37 (d, *J* = 6.2 Hz, 3H), 1.16 (m, 4H), 0.63 (d, *J* = 8.3 Hz, 6H). ^13^C NMR (101 MHz, DMSO-*d*_6_) δ: 154.89, 147.86, 145.90, 137.25, 136.12, 135.68, 134.69, 130.97, 128.84, 128.29, 128.11, 128.04, 124.36, 122.81, 112.28, 112.17, 111.73, 74.76, 70.20, 66.86, 56.06, 47.66, 38.20, 25.37, 25.18, 18.39, 15.35. LCMS (ESI): *m*/*z* [M+H] calcd for C_35_H_43_BrN_4_O_4_ 663.2; found 662.2.

#### (S)-4-(5-(1-Ethyl-3-(3-hydroxy-2,2-dimethylpropyl)-5-(4,4,5,5-tetramethyl-1,3,2-dioxaborolan-2-yl)-1H-indol-2-yl)-6-(1-methoxyethyl)­pyridin-3-yl)­piperazine-1-carboxylate (S10-6)

Into a 500 mL 3-necked round-bottom flask purged and maintained with an inert atmosphere of nitrogen was placed benzyl (*S*)-4-(5-(5-bromo-1-ethyl-3-(3-hydroxy-2,2-dimethylpropyl)-1*H*-indol-2-yl)-6-(1-methoxyethyl)­pyridin-3-yl)­piperazine-1-carboxylate **A** (14 g, 21.1 mmol), bis­(pinacolato)­diboron (5.89 g, 23.21 mmol), Pd­(dppf)­Cl_2_ (1.54 g, 2.1 mmol), KOAc (5.18 g, 52.7 mmol), and toluene (150 mL). The resulting solution was stirred for 5 h at 90 °C in an oil bath. The reaction mixture was cooled to room temperature. The resulting mixture was concentrated under reduced pressure. The residue was purified by silica gel column chromatography, eluted with Pet. ether/EtOAc (1:3) to give benzyl (*S*)-4-(5-(1-ethyl-3-(3-hydroxy-2,2-dimethylpropyl)-5-(4,4,5,5-tetramethyl-1,3,2-dioxaborolan-2-yl)-1*H*-indol-2-yl)-6-(1-methoxyethyl)­pyridin-3-yl)­piperazine-1-carboxylate (12 g, 76% yield) as a solid. ^1^H NMR (400 MHz, DMSO-*d*_6_) δ: 8.48 (d, *J* = 2.6 Hz, 1H), 8.09 (s, 1H), 7.56 (d, *J* = 8.2 Hz, 1H), 7.49–7.21 (m, 7H), 5.10 (s, 2H), 4.48 (m, 1H), 4.08–4.03 (m, 1H), 3.87 (m, 1H), 3.55 (d, *J* = 16.8 Hz, 10H), 3.34–3.19 (m, 4H), 3.07 (m, 2H), 2.82 (d, *J* = 13.8 Hz, 3H), 2.72 (d, *J* = 13.4 Hz, 1H), 2.26 (t, *J* = 15.0 Hz, 1H), 1.95 (s, 1H), 1.52–1.33 (m, 3H), 1.33–1.19 (m, 8H), 1.09 (s, 1H), 0.66 (dd, *J* = 30.5, 13.4 Hz, 6H). ^13^C NMR (101 MHz, DMSO-*d*_6_) δ: 154.90, 149.97, 145.37, 138.41, 137.96, 137.24, 135.68, 128.96, 128.84, 128.29, 128.03, 111.81, 83.52, 75.30, 71.24, 66.84, 60.21, 55.74, 48.06, 38.33, 25.36, 25.23, 25.14, 25.07, 21.11, 18.02, 15.37, 15.29, 14.47. LCMS (ESI): *m*/*z* [M+H] calcd for C_41_H_55_BN_4_O_6_ 710.4; found 711.3.

#### (S)-1-((S)-3-(4-(2-(5-(4-((Benzyloxy)­carbonyl)­piperazin-1-yl)-2-((S)-1-methoxyethyl)­pyridin-3-yl)-1-ethyl-3-(3-hydroxy-2,2-dimethylpropyl)-1H-indol-5-yl)­thiazol-2-yl)-2-((tert-butoxycarbonyl)­amino)­propanoyl)­hexahydropyridazine-3-carboxylate (S10-7)

Into a 250-mL round-bottom flask purged and maintained with an inert atmosphere of argon, was placed benzyl (*S*)-4-(5-(1-ethyl-3-(3-hydroxy-2,2-dimethylpropyl)-5-(4,4,5,5-tetramethyl-1,3,2-dioxaborolan-2-yl)-1*H*-indol-2-yl)-6-(1-methoxyethyl)­pyridin-3-yl)­piperazine-1-carboxylate (10.8 g, 15.2 mmol), methyl (3*S*)-1-[(2*S*)-3-(4-bromo-1,3-thiazol-2-yl)-2-[(*tert*-butoxycarbonyl)­amino]­propanoyl]-1,2-diazinane-3-carboxylate (7.98 g, 16.7 mmol), Pd­(dtbpf)­Cl_2_ (0.99 g, 1.52 mmol), K_3_PO_4_ (8.06 g, 37.9 mmol), toluene (60 mL), dioxane (20 mL), and H_2_O (20 mL). The resulting solution was stirred for 3 h at 70 °C in an oil bath. The reaction mixture was cooled to room temperature. The resulting solution was extracted with EtOAc (2 × 50 mL) and concentrated under reduced pressure. The residue was applied to a silica gel column with EtOAc/hexane (10:1). Removal of the solvent to give methyl (*S*)-1-((*S*)-3-(4-(2-(5-(4-((benzyloxy)­carbonyl)­piperazin-1-yl)-2-((*S*)-1-methoxyethyl)­pyridin-3-yl)-1-ethyl-3-(3-hydroxy-2,2-dimethylpropyl)-1*H*-indol-5-yl)­thiazol-2-yl)-2-(*(tert*-butoxycarbonyl)­amino)­propanoyl)­hexahydropyridazine-3-carboxylate (8 g, 51% yield) as a solid. ^1^H NMR (400 MHz, DMSO-*d*_6_) δ: 8.48 (d, *J* = 2.8 Hz, 1H), 8.22 (d, *J* = 1.6 Hz, 1H), 7.84–7.67 (m, 2H), 7.52 (d, *J* = 8.6 Hz, 1H), 7.42–7.27 (m, 6H), 6.62 (d, *J* = 8.7 Hz, 1H), 5.46–5.24 (m, 2H), 5.12 (s, 2H), 4.45 (t, *J* = 5.1 Hz, 1H), 4.16–3.98 (m, 2H), 3.91 (m, 2H), 3.61 (d, *J* = 25.6 Hz, 8H), 3.29 (m, 5H), 3.11 (dd, *J* = 10.2, 5.3 Hz, 1H), 3.02 (dd, *J* = 10.2, 5.2 Hz, 1H), 2.86 (s, 3H), 2.70 (d, *J* = 14.0 Hz, 1H), 2.51 (p, *J* = 1.8 Hz, 1H), 2.26 (d, *J* = 14.0 Hz, 1H), 1.95–1.80 (m, 1H), 1.77–1.46 (m, 3H), 1.38 (d, *J* = 5.1 Hz, 12H), 1.16 (dd, *J* = 8.4, 5.9 Hz, 4H), 0.66 (d, *J* = 22.3 Hz, 6H). ^13^C NMR (101 MHz, DMSO-*d*_6_) δ: 171.94, 171.33, 166.01, 154.90, 149.90, 145.40, 138.39, 137.28, 136.25, 135.82, 129.48, 128.87, 128.32, 128.07, 127.40, 126.50, 126.30, 120.70, 111.81, 110.23, 78.62, 75.32, 70.98, 66.85, 58.34, 55.76, 52.17, 48.04, 41.31, 38.36, 28.65, 25.27, 25.21, 18.03, 15.45. LCMS (ESI): *m*/*z* [M+H] calcd for C_52_H_68_N_8_O_9_S 980.5; found 980.9.

#### (S)-1-((S)-3-(4-(2-(5-(4-((Benzyloxy)­carbonyl)­piperazin-1-yl)-2-((S)-1-methoxyethyl)­pyridin-3-yl)-1-ethyl-3-(3-hydroxy-2,2-dimethylpropyl)-1H-indol-5-yl)­thiazol-2-yl)-2-((tert-butoxycarbonyl)­amino)­propanoyl)­hexahydropyridazine-3-carboxylic acid (S10-8)

To a stirred mixture of methyl (*S*)-1-((*S*)-3-(4-(2-(5-(4-((benzyloxy)­carbonyl)­piperazin-1-yl)-2-((S)-1-methoxyethyl)­pyridin-3-yl)-1-ethyl-3-(3-hydroxy-2,2-dimethylpropyl)-1*H*-indol-5-yl)­thiazol-2-yl)-2-((*tert*-butoxycarbonyl)­amino)­propanoyl)­hexahydropyridazine-3-carboxylate (12 g, 12.23 mmol) in THF (100 mL)/H_2_O (100 mL) was added LiOH (2.45 g, 61.1 mmol) under a nitrogen atmosphere, and the resulting mixture was stirred for 2 h at room temperature. THF was concentrated under reduced pressure. The pH of aqueous phase was acidified to 5 with HCL (1N) at 0 °C. The aqueous layer was extracted with DCM (3 × 100 mL). The organic phase was concentrated under reduced pressure to give (*S*)-1-((*S*)-3-(4-(2-(5-(4-((benzyloxy)­carbonyl)­piperazin-1-yl)-2-((*S*)-1-methoxyethyl)­pyridin-3-yl)-1-ethyl-3-(3-hydroxy-2,2-dimethylpropyl)-1*H*-indol-5-yl)­thiazol-2-yl)-2-((*tert*-butoxycarbonyl)­amino)­propanoyl)­hexahydropyridazine-3-carboxylic acid (10 g, 85% yield) as a solid. ^1^H NMR (400 MHz, DMSO-*d*_6_) δ: 8.47 (d, *J* = 2.7 Hz, 1H), 8.29 (s, 1H), 7.75 (d, *J* = 8.7 Hz, 2H), 7.59–7.18 (m, 8H), 5.23 (s, 1H), 5.11 (s, 2H), 4.63 (d, *J* = 11.4 Hz, 1H), 4.20 (d, *J* = 11.4 Hz, 1H), 4.06 (m, 3H), 3.89 (m, 2H), 3.62–3.44 (m, 12H), 3.12 (d, *J* = 10.1 Hz, 1H), 3.02 (d, *J* = 10.3 Hz, 2H), 2.86 (d, *J* = 6.7 Hz, 3H), 2.70 (d, *J* = 16.6 Hz, 2H), 2.25 (d, *J* = 13.8 Hz, 1H), 1.96 (s, 1H), 1.71 (s, 2H), 1.28–0.96 (m, 11H), 0.65 (d, *J* = 21.1 Hz, 9H). ^13^C NMR (101 MHz, DMSO-*d*_6_) δ: 165.66, 155.92, 155.24, 154.92, 149.87, 145.37, 137.23, 136.13, 135.75, 128.89, 128.35, 128.05, 111.91, 78.80, 75.28, 70.86, 66.84, 55.79, 47.99, 28.63, 25.31, 25.22, 18.09, 15.45. LCMS (ESI): *m*/*z* [M+H] calcd for C_51_H_66_N_8_O_9_S 966.5; found 967.0.

#### Benzyl 4-(5-((6^3^S,4S,Z)-4-((tert-butoxycarbonyl)­amino)-1^1^-ethyl-10,10-dimethyl-5,7-dioxo-6^1^,6^2^,6^3^,6^4^,6^5^,6^6^-hexahydro-1^1^H-8-oxa-2­(4,2)-thiazola-1­(5,3)-indola-6­(1,3)-pyridazinacycloundecaphane-1^2^-yl)-6-((S)-1-methoxyethyl)­pyridin-3-yl)­piperazine-1-carboxylate (S10-9)

Into a 3-L round-bottom flask purged and maintained with an inert atmosphere of nitrogen, was placed (*S*)-1-((*S*)-3-(4-(2-(5-(4-((benzyloxy)­carbonyl)­piperazin-1-yl)-2-((*S*)-1-methoxyethyl)­pyridin-3-yl)-1-ethyl-3-(3-hydroxy-2,2-dimethylpropyl)-1*H*-indol-5-yl)­thiazol-2-yl)-2-((*tert*-butoxycarbonyl)­amino)­propanoyl)­hexahydropyridazine-3-carboxylic acid (18 g, 18.61 mmol), MeCN (1.8 L), DIPEA (96.21 g, 744.4 mmol), EDCI (107.03 g, 558.3 mmol), HOBT (25.15 g, 186.1 mmol). The resulting solution was stirred overnight at 25 °C. The mixture was concentrated under reduced pressure after the reaction was complete. The resulting solution was diluted with DCM (1 L). The resulting mixture was washed with HCl (3 × 1 L, 1N). The resulting mixture was washed with H_2_O (3 × 1 L). Then the organic layer was concentrated, and the residue was applied onto a silica gel column with EtOAc/hexane (1:1). Removal of solvent under reduced pressure gave benzyl 4-(5-((6^3^ *S*,4*S*,*Z*)-4-((*tert*-butoxycarbonyl)­amino)-1^1^-ethyl-10,10-dimethyl-5,7-dioxo-6^1^,6^2^,6^3^,6^4^,6^5^,6^6^-hexahydro-1^1^ *H*-8-oxa-2­(4,2)-thiazola-1­(5,3)-indola-6­(1,3)-pyridazinacycloundecaphane-1^2^-yl)-6-((*S*)-1-methoxyethyl)­pyridin-3-yl)­piperazine-1-carboxylate (10.4 g, 55% yield) as a solid. ^1^H NMR (400 MHz, DMSO-*d*_6_) δ: 8.50 (d, *J* = 1.7 Hz, 1H), 8.47 (d, *J* = 2.8 Hz, 1H), 7.80 (s, 1H), 7.74 (dd, *J* = 8.6, 1.6 Hz, 1H), 7.55 (d, *J* = 8.7 Hz, 1H), 7.41–7.35 (m, 4H), 7.35–7.22 (m, 3H), 5.24 (t, *J* = 8.7 Hz, 1H), 5.11 (s, 2H), 5.06 (d, *J* = 12.2 Hz, 1H), 4.22 (m, 5H), 3.56 (t, *J* = 7.9 Hz, 6H), 3.29 (t, *J* = 5.3 Hz, 4H), 3.22 (s, 3H), 2.95 (d, *J* = 14.3 Hz, 1H), 2.77 (m, 1H), 2.50 (m, 1H), 2.44 (d, *J* = 14.4 Hz, 1H), 2.08 (t, *J* = 9.2 Hz, 1H), 1.80 (d, *J* = 9.0 Hz, 2H), 1.40 (s, 9H), 1.34 (d, *J* = 6.2 Hz, 4H), 1.08 (s, 1H), 0.95–0.84 (m, 6H), 0.35 (s, 3H). ^13^C NMR (101 MHz, DMSO-*d*_6_) δ: 172.32, 167.04, 155.84, 154.90, 154.62, 149.67, 145.94, 137.44, 137.27, 135.52, 130.20, 128.86, 128.31, 128.06, 127.22, 126.38, 111.60, 78.50, 66.84, 55.82, 47.93, 35.69, 28.70, 26.25, 24.89, 16.49, 15.33. LCMS (ESI): *m*/*z* [M+H] calcd for C_51_H_64_N_8_O_8_S 948.5; found 949.3.

#### tert-Butyl ((6^3^S,4S,Z)-11-ethyl-1^2^-(2-((S)-1-methoxyethyl)-5-(piperazin-1-yl)­pyridin-3-yl)-10,10-dimethyl-5,7-dioxo-6^1^,6^2^,6^3^,6^4^,6^5^,6^6^-hexahydro-1^1^H-8-oxa-2­(4,2)-thiazola-1­(5,3)-indola-6­(1,3)-pyridazinacycloundecaphane-4-yl)­carbamate (Intermediate Macrocycle 4)

Into a 250-mL round-bottom flask purged and maintained with an inert atmosphere of nitrogen was placed benzyl 4-(5-((6^3^ *S*,4*S*,*Z*)-4-((*tert*-butoxycarbonyl)­amino)-1^1^-ethyl-10,10-dimethyl-5,7-dioxo-6^1^,6^2^,6^3^,6^4^,6^5^,6^6^-hexahydro-1^1^ *H*-8-oxa-2­(4,2)-thiazola-1­(5,3)-indola-6­(1,3)-pyridazinacycloundecaphane-1^2^-yl)-6-((*S*)-1-methoxyethyl)­pyridin-3-yl)­piperazine-1-carboxylate (10.40 g, 10.9 mmol), Pd­(OH)_2_/C (5 g, 46.9 mmol), MeOH (100 mL). The resulting solution was stirred for 3 h at room temperature under a 2 atm of H_2_ atmosphere. The solids were filtered out and the filter cake was washed with MeOH (3 × 100 mL). Then the combined organic phase was concentrated under reduced pressure to give *tert*-butyl ((6^3^ *S*,4*S*,*Z*)-1*1*-ethyl-1^2^-(2-((*S*)-1-methoxyethyl)-5-(piperazin-1-yl)­pyridin-3-yl)-10,10-dimethyl-5,7-dioxo-6^1^,6^2^,6^3^,6^4^,6^5^,6^6^-hexahydro-1^1^ *H*-8-oxa-2­(4,2)-thiazola-1­(5,3)-indola-6­(1,3)-pyridazinacycloundecaphane-4-yl)­carbamate (8.5 g, 90% yield) as a solid. ^1^H NMR (400 MHz, DMSO-*d*_6_) δ: 8.54 (d, *J* = 1.7 Hz, 1H), 8.49 (dd, *J* = 8.8, 2.9 Hz, 1H), 7.84 (s, 1H), 7.77 (dd, *J* = 8.6, 1.6 Hz, 1H), 7.59 (d, *J* = 8.7 Hz, 1H), 7.33 (d, *J* = 9.2 Hz, 1H), 7.21 (d, *J* = 2.9 Hz, 1H), 5.79 (s, 1H), 5.28 (t, *J* = 9.0 Hz, 1H), 5.10 (d, *J* = 12.2 Hz, 1H), 4.44–4.05 (m, 6H), 3.63 (t, *J* = 7.7 Hz, 3H), 3.49–3.07 (m, 13H), 3.06–2.94 (m, 2H), 2.89 (dd, *J* = 6.3, 4.0 Hz, 3H), 2.81 (t, *J* = 13.7 Hz, 1H), 2.66 (s, 1H), 2.54 (m, 1H), 2.49 (d, *J* = 14.4 Hz, 1H), 2.13 (d, *J* = 14.4 Hz, 1H), 1.83 (s, 2H), 1.66–1.50 (m, 1H), 1.44 (s, 8H), 1.38 (d, *J* = 6.1 Hz, 4H), 1.12 (s, 1H), 0.95 (d, *J* = 7.1 Hz, 6H), 0.40 (s, 3H). ^13^C NMR (101 MHz, DMSO-*d*_6_) δ: 173.07, 172.31, 167.02, 155.84, 154.63, 146.59, 137.62, 135.50, 130.22, 127.12, 124.89, 119.57, 111.51, 111.27, 78.47, 55.78, 55.33, 48.82, 45.53, 35.68, 28.72, 26.27, 24.90, 16.50, 15.34. LCMS (ESI): *m*/*z* [M+H] calcd for C_43_H_58_N_8_O_6_S 814.4; found 815.3.

#### tert-Butyl ((6^3^S,4S,Z)-1^1^-ethyl-1^2^-(2-((S)-1-methoxyethyl)-5-(4-methylpiperazin-1-yl)­pyridin-3-yl)-10,10-dimethyl-5,7-dioxo-6^1^,6^2^,6^3^,6^4^,6^5^,6^6^-hexahydro-1^1^H-8-oxa-2­(4,2)-thiazola-1­(5,3)-indola-6­(1,3)-pyridazinacycloundecaphane-4-yl)­carbamate (S11-1)

A solution of *tert*-butyl ((6^3^ *S*,4*S*,*Z*)-1*1*-ethyl-1^2^-(2-((*S*)-1-methoxyethyl)-5-(piperazin-1-yl)­pyridin-3-yl)-10,10-dimethyl-5,7-dioxo-6^1^,6^2^,6^3^,6^4^,6^5^,6^6^-hexahydro-1^1^ *H*-8-oxa-2­(4,2)-thiazola-1­(5,3)-indola-6­(1,3)-pyridazinacycloundecaphane-4-yl)­carbamate (1.5 g, 1.58 mmol), Pd­(OH)_2_/C (1.5 g) and Paraformaldehyde (208.85 mg, 4.74 mmol) in MeOH (15 mL) was stirred for 2 h at room temperature under a hydrogen atmosphere. The resulting mixture was filtered, and the filter cake was washed with MeOH (3 × 50 mL). The filtrate was concentrated under a reduced pressure. The residue was purified by reversed-phase flash chromatography with the following conditions: column, C18 silica gel; mobile phase, MeCN in water (10 mmol/L NH_4_HCO_3_), 10% to 70% gradient in 30 min; detector, UV 254 nm. This resulted in tert-butyl ((6^3^S,4S,Z)-1^1^-ethyl-1^2^-(2-((S)-1-methoxyethyl)-5-(4-methylpiperazin-1-yl)­pyridin-3-yl)-10,10-dimethyl-5,7-dioxo-6^1^,6^2^,6^3^,6^4^,6^5^,6^6^-hexahydro-1^1^H-8-oxa-2­(4,2)-thiazola-1­(5,3)-indola-6­(1,3)-pyridazinacycloundecaphane-4-yl)­carbamate (800 mg, 58% yield) as a yellow solid. ^1^H NMR (400 MHz, DMSO-*d*_6_) δ: 8.37–8.15 (m, 2H), 7.61–7.43 (m, 2H), 7.31 (d, *J* = 8.6 Hz, 1H), 7.12–6.90 (m, 2H), 5.17–4.96 (m, 1H), 4.85 (d, *J* = 11.8 Hz, 1H), 4.22–3.86 (m, 5H), 3.39 (q, *J* = 11.7 Hz, 3H), 3.16–2.89 (m, 9H), 2.77 (d, *J* = 14.1 Hz, 1H), 2.57 (d, *J* = 17.4 Hz, 1H), 2.36–2.09 (m, 5H), 1.98 (s, 3H), 1.91–1.81 (m, 1H), 1.66–1.44 (m, 2H), 1.17 (d, *J* = 17.7 Hz, 12H), 0.70 (d, *J* = 8.5 Hz, 6H), 0.16 (s, 3H). ^13^C NMR (101 MHz, DMSO-*d*_6_) δ: 173.04, 172.29, 167.00, 155.81, 154.63, 149.06, 146.09, 137.59, 135.50, 130.21, 127.12, 126.35, 111.52, 78.45, 60.19, 55.76, 54.64, 47.87, 46.24, 35.68, 28.71, 26.26, 24.89, 16.49, 15.34, 14.53. LCMS (ESI): *m*/*z* [M+H] calcd for C_44_H_60_N_8_O_6_S 828.4; found 829.3.

#### (6^3^S,4S,Z)-4-Amino-1^1^-ethyl-1^2^-(2-((S)-1-methoxyethyl)-5-(4-methylpiperazin-1-yl)­pyridin-3-yl)-10,10-dimethyl-6^1^,6^2^,6^3^,6^4^,6^5^,6^6^-hexahydro-1^1^H-8-oxa-2­(4,2)-thiazola-1­(5,3)-indola-6­(1,3)-pyridazinacycloundecaphane-5,7-dione (S11-2)

A solution of *tert*-butyl ((6^3^ *S*,4*S*,*Z*)-1^1^-ethyl-1^2^-(2-((*S*)-1-methoxyethyl)-5-(4-methylpiperazin-1-yl)­pyridin-3-yl)-10,10-dimethyl-5,7-dioxo-6^1^,6^2^,6^3^,6^4^,6^5^,6^6^-hexahydro-1^1^ *H*-8-oxa-2­(4,2)-thiazola-1­(5,3)-indola-6­(1,3)-pyridazinacycloundecaphane-4-yl)­carbamate (800 mg, 0.97 mmol) in DCM (10 mL) was treated with TFA (2 mL) at 0 °C. After 3 min, the resulting solution was warmed to room temperature and then stirred for 2 h. The mixture was then basified to pH 7 with saturated NaHCO_3_ (aq.). The resulting mixture was extracted with DCM (3 × 50 mL) and dried over anhydrous Na_2_SO_4_. After filtration, the filtrate was concentrated under reduced pressure to afford (6^3^ *S*,4*S*,*Z*)-4-amino-1^1^-ethyl-1^2^-(2-((*S*)-1-methoxyethyl)-5-(4-methylpiperazin-1-yl)­pyridin-3-yl)-10,10-dimethyl-6^1^,6^2^,6^3^,6^4^,6^5^,6^6^-hexahydro-1^1^ *H*-8-oxa-2­(4,2)-thiazola-1­(5,3)-indola-6­(1,3)-pyridazinacycloundecaphane-5,7-dione (680 mg, crude) as a solid. ^1^H NMR (400 MHz, DMSO-*d*_6_) δ: 8.48 (dd, *J* = 18.2, 2.2 Hz, 2H), 7.81–7.65 (m, 2H), 7.54 (d, *J* = 8.6 Hz, 1H), 7.23 (d, *J* = 2.9 Hz, 1H), 4.93 (d, *J* = 12.1 Hz, 1H), 4.38 (d, *J* = 8.5 Hz, 1H), 4.29 (p, *J* = 7.3, 5.9 Hz, 2H), 4.21–4.09 (m, 3H), 4.03 (q, *J* = 7.1 Hz, 1H), 3.60 (q, *J* = 10.9 Hz, 2H), 3.21 (s, 3H), 2.97 (d, *J* = 14.5 Hz, 1H), 2.75 (m, 2H), 2.54–2.37 (m, 6H), 2.21 (s, 3H), 2.08 (d, *J* = 12.1 Hz, 1H), 1.99 (s, 2H), 1.88–1.64 (m, 2H), 1.51 (m, 1H), 1.35 (d, *J* = 6.1 Hz, 3H), 1.23 (s, 1H), 1.18 (t, *J* = 7.1 Hz, 2H), 0.97–0.85 (m, 6H), 0.36 (s, 3H). ^13^C NMR (101 MHz, DMSO-*d*_6_) δ: 176.35, 172.38, 168.21, 154.46, 148.98, 146.08, 137.48, 135.44, 130.17, 127.17, 126.50, 125.00, 119.62, 117.54, 111.54, 110.90, 110.75, 75.73, 70.80, 57.85, 55.73, 54.62, 51.37, 47.83, 46.23, 35.73, 26.15, 24.91, 16.50, 15.35. LCMS (ESI): *m*/*z* [M+H] calcd for C_39_H_52_N_8_O_4_S 728; found 729.

#### (1S,2S)-N-((6^3^S,4S,Z)-1^1^-Ethyl-1^2^-(2-((S)-1-methoxyethyl)-5-(4-methylpiperazin-1-yl)­pyridin-3-yl)-10,10-dimethyl-5,7-dioxo-6^1^,6^2^,6^3^,6^4^,6^5^,6^6^-hexahydro-1^1^H-8-oxa-2­(4,2)-thiazola-1­(5,3)-indola-6­(1,3)-pyridazinacycloundecaphane-4-yl)-2-methylcyclopropane-1-carboxamide (compound **13**, daraxonrasib (RMC-6236) )

To a stirred solution of (6^3^ *S*,4*S*,*Z*)-4-amino-1^1^-ethyl-1^2^-(2-((*S*)-1-methoxyethyl)-5-(4-methylpiperazin-1-yl)­pyridin-3-yl)-10,10-dimethyl-6^1^,6^2^,6^3^,6^4^,6^5^,6^6^-hexahydro-1^1^ *H*-8-oxa-2­(4,2)-thiazola-1­(5,3)-indola-6­(1,3)-pyridazinacycloundecaphane-5,7-dione (400 mg, 0.55 mmol) and (1S,2S)-2-methylcyclopropane-1-carboxylic acid (65.92 mg, 0.66 mmol) in DMF (4 mL) was added DIPEA (283.7 mg, 2.2 mmol) and HATU (229.51 mg, 0.60 mmol) in portions at 0 °C under a nitrogen atmosphere. The resulting mixture was stirred for 40 min at 0 °C then the reaction was quenched by the addition of sat. NH_4_Cl (aq.) (30 mL). The resulting mixture was extracted with EtOAc (3 × 10 mL). The combined organic layers were washed with brine (2 × 50 mL) and dried over anhydrous Na_2_SO_4_. After filtration, the filtrate was concentrated under reduced pressure. The resulting crude product was purified by Prep-HPLC under the following conditions (Column: C18 spherical, 20–35 um; 100 A, 80 g; Mobile Phase A:Water­(10 MMOL/L NH_4_HCO_3_), Mobile Phase B:ACN; Flow rate: 60 mL/min; Gradient: 30 to 70 B in 30 min, 254 nm; RT1:15.2) to afford (1*S*,2*S*)-*N*-((6^3^ *S*,4*S*,*Z*)-1^1^-ethyl-1^2^-(2-((*S*)-1-methoxyethyl)-5-(4-methylpiperazin-1-yl)­pyridin-3-yl)-10,10-dimethyl-5,7-dioxo-6^1^,6^2^,6^3^,6^4^,6^5^,6^6^-hexahydro-1^1^ *H*-8-oxa-2­(4,2)-thiazola-1­(5,3)-indola-6­(1,3)-pyridazinacycloundecaphane-4-yl)-2-methylcyclopropane-1-carboxamide (102 mg, 96.4% purity, 22% yield) as a white solid. ^1^H NMR (400 MHz, DMSO-*d*_6_) δ: 8.26 (d, *J* = 22.0 Hz, 3H), 7.50 (d, *J* = 18.5 Hz, 2H), 7.29 (d, *J* = 8.5 Hz, 1H), 6.98 (s, 1H), 5.37 (t, *J* = 9.2 Hz, 1H), 4.84 (d, *J* = 11.7 Hz, 1H), 4.16–3.84 (m, 5H), 3.15 (s, 2H), 3.01 (d, *J* = 18.9 Hz, 9H), 2.74 (d, *J* = 14.0 Hz, 1H), 2.23 (d, *J* = 20.8 Hz, 5H), 1.96 (s, 3H), 1.86 (s, 1H), 1.55 (s, 2H), 1.29 (s, 2H), 1.21–1.01 (m, 3H), 0.84 (s, 4H), 0.68 (s, 8H), 0.31 (s, 1H), 0.14 (s, 3H). ^13^C NMR (100 MHz, DMSO) δ: 172.78, 172.32, 166.74, 154.72, 149.04, 146.09, 137.56, 135.49, 130.18, 127.12, 126.32, 125.00, 119.59, 117.45, 111.51, 111.36, 110.86, 75.72, 70.73, 57.89, 55.81, 54.65, 49.42, 47.87, 46.28, 38.24, 35.69, 33.00, 28.94, 26.23, 24.92, 23.47, 22.58, 21.16, 18.08, 16.54, 15.37, 15.09. HRMS (ESI): *m*/*z* [M+H] calcd for C_44_H_58_N_8_O_5_S 811.43073.; found 811.43236.

See the for schemes and more detailed synthetic information for additional compounds.

## Supplementary Material





## Notes

Experimental details for *in vitro* and *in vivo* studies, additional crystallographic data for compounds **2**, **3**, **6**, **10**, and daraxonrasib (RMC-6236) (PDF)
Molecular formula strings (CSV)

## Notes

Atomic coordinates for X-ray structures of tri-complexes formed with compounds **2**, **3**, **6**, **10**, and daraxonrasib (RMC-6236) have been deposited to the PDB with accession codes: 9BG0, 9BG1, 9BG2, 9BG3, 9BG4, 9BG5, 9BG6, 9BG7, 9BG8, 9BG9, 9BGA, 9BGB, 9BGC, and 9BGD.

## Abbreviations

ADME: absorption, distribution, metabolism, and excretion
AUC_inf_: area under the curve extrapolated to infinity
BID: bis in die (twice a day)
BTK: Bruton’s tyrosine kinase
CDX: cell-line derived xenograft
CL: clearance
*C*_max_: maximum concentration
%F: bioavailability
GDP: guanosine diphosphate
GMPPNP: 5′-guanylyl imidodiphosphate
GTP: guanosine triphosphate
iv: intravenous
KRAS: Kirsten rat sarcoma
LCMS: liquid chromatography mass spectrometry
MS: Mass Spectrometry
NSCLC: non-small cell lung cancer
pERK: phosphorylated ERK
PD: pharmacodynamics
PDX: patient derived xenograft
P-gp: P-glycoprotein
PK: pharmacokinetics
po: per os (oral)
QD: quaque die (once daily)
RAF: rapidly accelerated fibrosarcoma kinase
RAS: rat sarcoma virus
RET: rearranged during transfection
SAR: structure–activity relationship
*T*_max_: time at *C*_max_
WT: wild type
