# Supplementary material for: Discovery of Daraxonrasib (RMC-6236), a Potent and Orally Bioavailable RAS(ON) Multi-selective, Noncovalent Tri-complex Inhibitor for the Treatment of Patients with Multiple RAS-Addicted Cancers
Source: J Med Chem. 2025 Mar 8;68(6):6064–83. doi: 10.1021/acs.jmedchem.4c02314 (PMC13726386; doi:10.1021/acs.jmedchem.4c02314)
Supplement: Supplementary file 1 [file jm4c02314_si_001.pdf]

Supporting Information

Discovery of daraxonrasib (RMC-6236), a potent and orally bioavailable RAS(ON) multi-selective, noncovalent tri-complex inhibitor for the treatment of patients with multiple RAS-addicted cancers

James Cregg, Anne V. Edwards, Stephanie Chang, Bianca J. Lee, John E. Knox, Aidan C. A. Tomlinson, Abby Marquez, Yang Liu, Rebecca Freilich, Naing Aay, Yingyun Wang, Lingyan Jiang, Jingjing Jiang, Zhican Wang, Michael Flagella, David Wildes, Jacqueline A.M. Smith, Mallika Singh, Zhengping Wang, Adrian L. Gill, Elena S. Koltun**

Revolution Medicines, Inc., Redwood City, CA 94063, USA

*e-mail: jcregg@revmed.com; elena@revmed.com

Contents

Supplemental Table 1 3

Supplemental Table 2 4

Supplementary Figure 1 5

Supplemental Table 3 5

Supplemental Table 4 6

Supplemental Figure 2 6

General Synthetic Procedures 6

  Synthesis of Intermediate **1** 7

  Synthesis of Intermediate Macrocycle 1 10

  Synthesis of **Compound 1** 14

  Synthesis of **Intermediate 2** 18

  Synthesis of **Compound 2** 19

Synthesis of Intermediate Macrocycle 2 20
Synthesis of **Compound 3** 25
Synthesis of **Compound 4** 28
Synthesis of **Compound 5** 30
Synthesis of Intermediate 3 31
Synthesis of **Compound 6** 33
Synthesis of **Compound 7** 34
Synthesis of **Compound 8** 36
Synthesis of **Compound 9** 37
Synthesis of **Compound 10** 38
Synthesis of **Compound 11** 39
Synthesis of Common Intermediate Macrocycle 3 40
Synthesis of **Compound 12** 46
Synthesis of Compound 13 (RMC-6236) 49
Synthesis of **Compound 14** 50
Synthesis of **Compound 15** 53
Synthesis of **Compound 16** 55
Synthesis of **Compound 17** 62
Synthesis of **Compound 18** 63
Synthesis of **Compound 19** 65
Methods 67
Tri-complex crystallization 67
Crystallography data collection and refinement 67
Supplementary Table 5. 67
CypA binding affinity 72
RAS-BRAF Disruption by TR-FRET 73
RAS binding affinity 73
Cellular ERK phosphorylation assays 73
2D cell proliferation 74
In Vitro Permeability 74
ePSA Method. 74
Kinetic Solubility Method – PBS at pH 7.4 and Sodium Citrate at pH 5 75
LogD Method. 76
Microsomal Stability 76
Blood-Plasma Partitioning 76

Blood Stability 77
Hepatocyte Stability 77
Mouse PK Studies 77
Rat, Dog, and Monkey PK Studies 77
Bioanalysis of PK Samples 78
Xenograft Studies 78
Cell Culture 78
Efficacy study 78
Single-dose pharmacokinetic/pharmacodynamics (PK/PD) study 79
In vivo pharmacodynamic analysis by DUSP6 qPCR 79
Incidence estimates of mutant RAS cancers 79

Supplemental Table 1: Estimated incidence of select RAS mutant solid tumors

Cancer Type	Estimated US incidence treated with systemic therapy (2023)	RAS G12X %	RAS G12X incidence	RAS G13X %	RAS G13X incidence	RAS Q61X %	RAS Q61X incidence	RASmut %	RASmut incidence
**Biliary**	**12220**	**19.9**	**2400**	**1.7**	**200**	**3**	**300**	**24.6**	**2900**
**Bladder**	**16710**	**4.6**	**800**	**0.9**	**100**	**1.4**	**200**	**6.9**	**1100**
**CRC**	**153020**	**34.9**	**53500**	**9.1**	**14100**	**4.8**	**7400**	**48.8**	**75000**
**CUP**	**32590**	**17.7**	**5600**	**2**	**600**	**3.1**	**900**	**22.8**	**7100**
**Endometrial**	**13030**	**14.9**	**1900**	**2.4**	**300**	**1.4**	**100**	**18.7**	**2300**
**Esophageal**	**21560**	**4.2**	**900**	**1**	**200**	**0.5**	**0**	**5.7**	**1100**
**HNSCC**	**46359**	**1.9**	**600**	**0.7**	**200**	**1.1**	**400**	**3.7**	**1200**

**Melanoma**	**7990**	**2.3**	**100**	**2.2**	**100**	**24.6**	**1900**	**29.1**	**2100**
**NSCLC**	**202589**	**24.6**	**49800**	**2.2**	**4400**	**2.7**	**5400**	**29.5**	**59600**
**Ovarian**	**19710**	**7.8**	**1500**	**0.5**	**100**	**1.2**	**100**	**9.5**	**1700**
**PDAC**	**57645**	**85**	**49200**	**0.7**	**400**	**6.3**	**3700**	**92**	**53300**
**Renal**	**81800**	**2.1**	**1900**	**0.5**	**400**	**1.1**	**1000**	**3.7**	**3300**
**Stomach**	**26500**	**7**	**1800**	**1.9**	**500**	**1.1**	**200**	**10**	**2500**
**Estimated Incidence (Total)**			**170000**		**21600**		**21600**		**213200**
**Hotspot Percentage (Total)**			**80%**		**10%**		**10%**		

Supplemental Table 2: Specific RAS Mutations in Solid Tumors

Indication	KRAS G12D	KRAS G12C	KRAS G12V	KRAS Other	NRAS All	HRAS All	Indication Total
**CRC**	**22800**	**5500**	**15600**	**24700**	**6400**	**0**	**75000**
**NSCLC**	**8300**	**23300**	**11100**	**14900**	**1600**	**400**	**59600**
**OTHER**	**6800**	**2200**	**4700**	**5500**	**3200**	**2900**	**25300**
**PDAC**	**22800**	**900**	**16400**	**13000**	**200**	**0**	**53300**

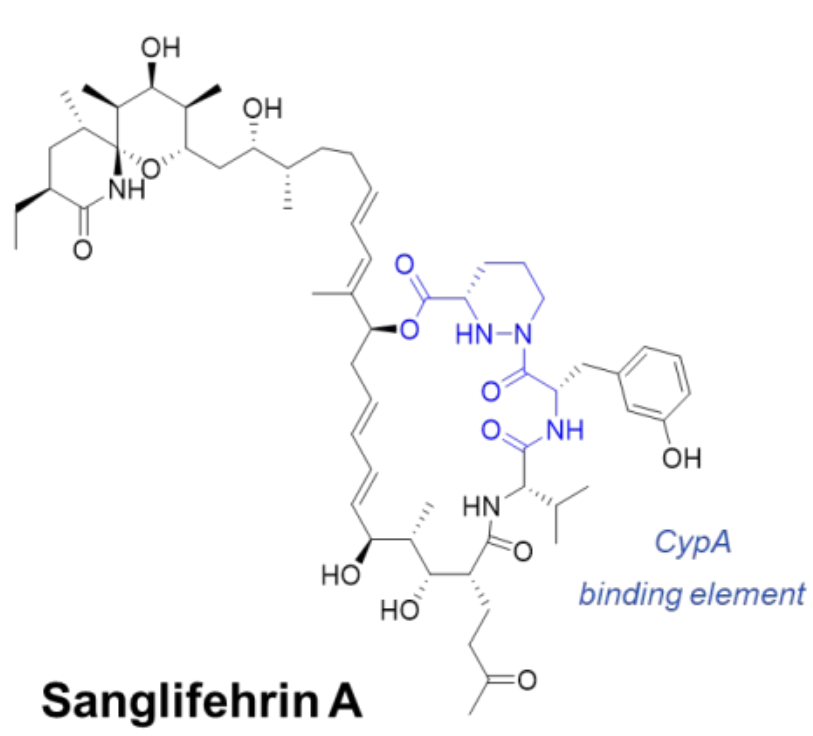

Sanglifehrin A

Prototype for Tri-complex Ligand

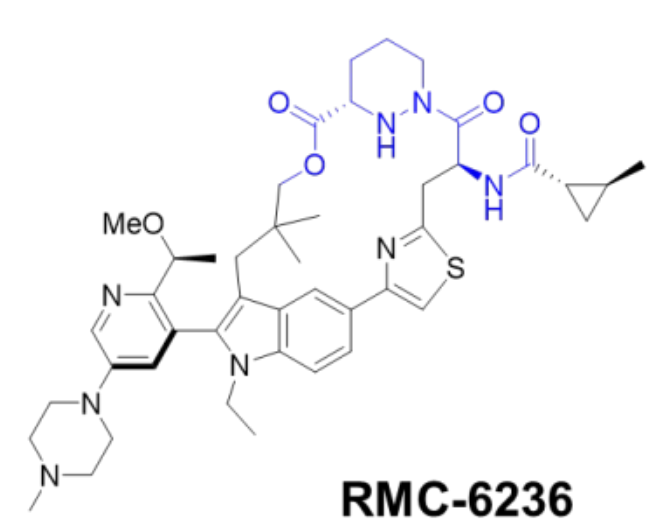

RMC-6236

Supplementary Figure 1. Prototype for the CypA binding portion of RMC-6236.

Supplemental Table 3. Replacements of the phenyl moiety in the macrocycle

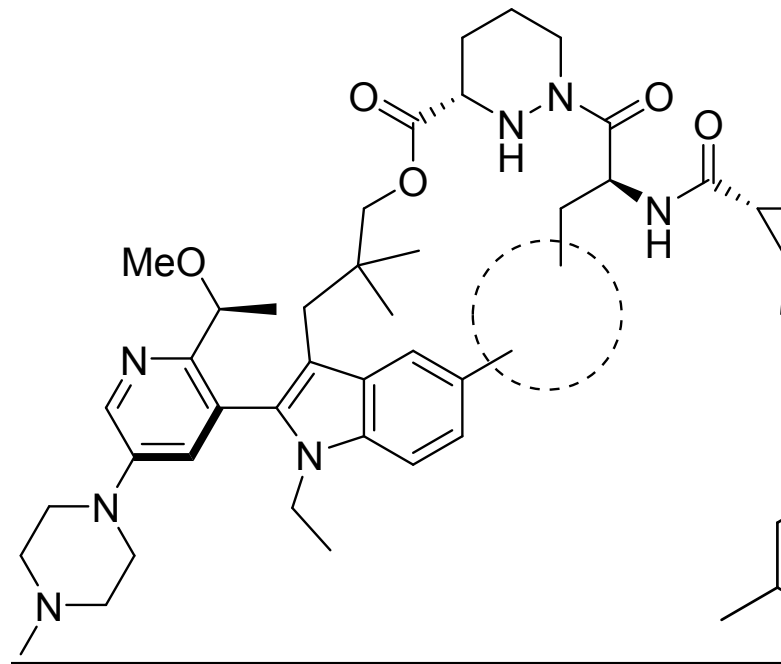

		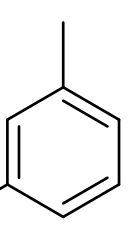	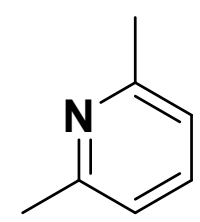	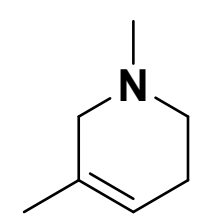	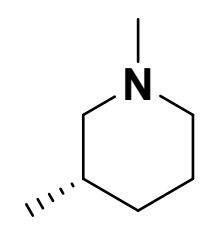	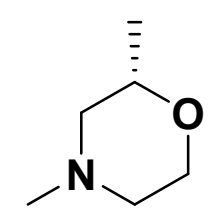	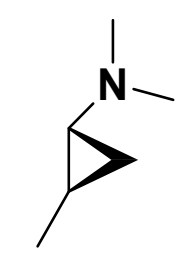
**Assays**		**20**	**21**	**22**	**23**	**24**	**25**
**KRAS-BRAF Disruption Assay IC$_{50}$, nM**	**G12V**	**241**	**429**	**632**	**3538**	**1100**	**3347**
	**G12C**	**186**	**302**	**463**	**2013**	**733**	**2205**
	**G12D**	**1405**	**1778**	**3690**	**3150**	**4561**	**14468**
	**WT**	**378**	**609**	**1205**	**2041**	**1430**	**5805**
**CypA KD1 nM**		**602**	**228**	**670**	**1160**	**177**	**52**
**pERK EC$_{50}$, nM**	**Capan-1 G12V**	**25**	**10**	**46**	**295**	**19**	**16**
	**NCI-H358 G12C**		**26**			**45**	
	**AsPC-1 G12D**	**295**	**128**	**1791**	**>1000**	**469**	
**CTG 2D EC$_{50}$, nM**	**Capan-1 G12V**	**162**	**67**	**518**	**>972**	**242**	**23**
	**NCI-H358 G12C**		**34**			**154**	
	**AsPC-1 G12D**	**457**	**244**	**1660**	**>972**	**1349**	
	**A375**	**4240**	**>10000**	**>10000**	**>10000**	**>10000**	**4436**

Supplemental Table 4. ADME properties of RMC-6236.

Caco-2 permeability, $P_{app,A-B}$ / efflux ratio	4 nm/s / 21
plasma protein binding (m/ h)	98.9% / 97.7%
blood stability, $t_{1/2}$ (m/ h)	>240 / >240 min
in vitro blood-plasma partitioning, mouse (0.1 µM/ 1 µM/ 10 µM)	4.2 / 3.0 / 1.1
in vitro blood-plasma partitioning, human (0.1 µM/ 1 µM/ 10 µM)	3.0 / 1.9 / 1.0
hepatocyte stability, $t_{1/2}$ (m/ r/ d/ c/ h)	166 / 116 / 184 / 50 / 103 min

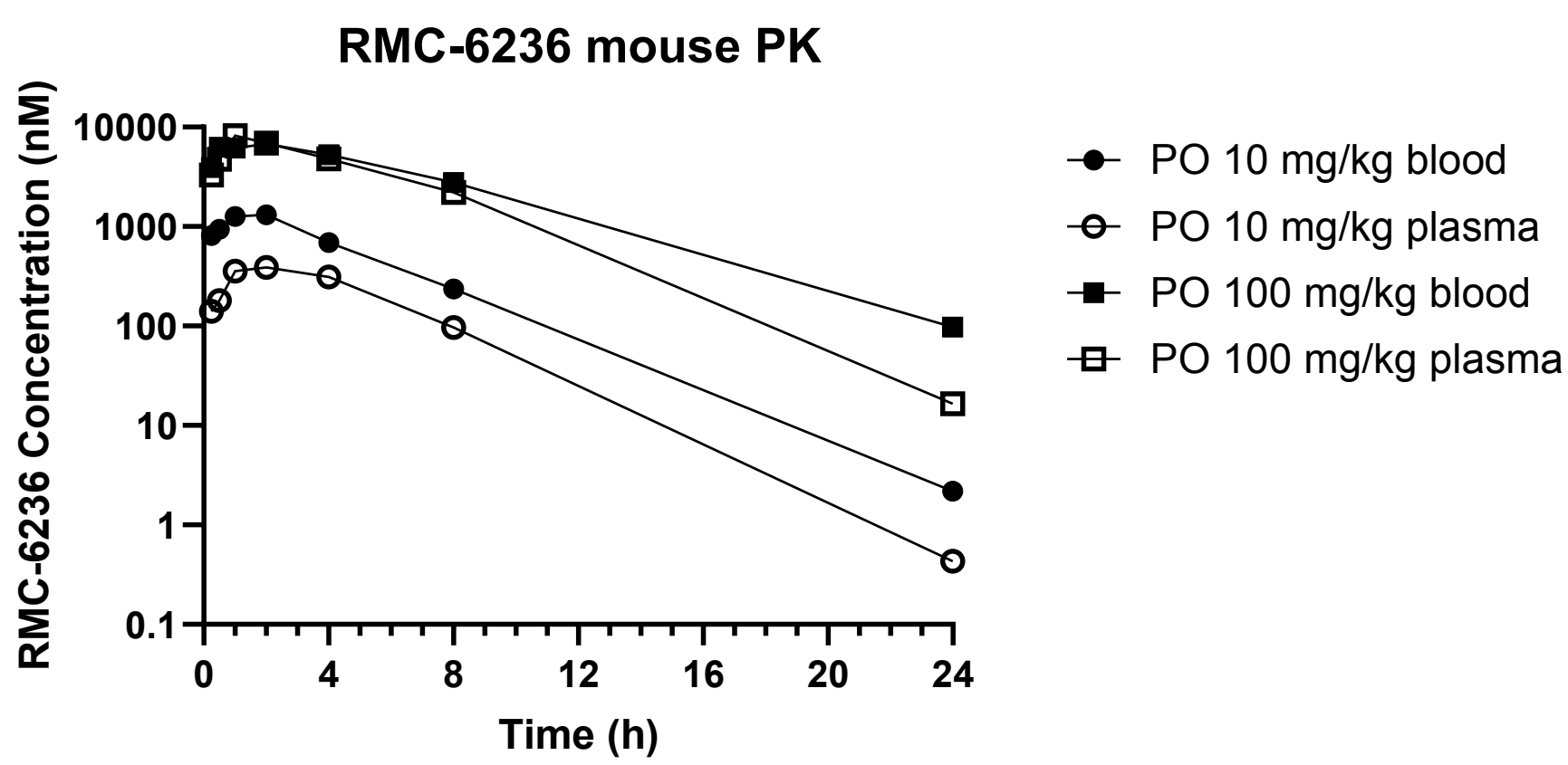

Supplemental Figure 2. Mouse oral PK profiles demonstrate concentration-dependent *in vivo* blood/plasma partitioning of RMC-6236.

General Synthetic Procedures

All final compounds were purified to ≥95% purity by either high-performance liquid chromatography (HPLC) or supercritical fluid chromatography (SFC) and were characterized by proton NMR, carbon NMR, and high-resolution mass spectrometry as described below. All materials were obtained from

commercial suppliers and used without further purification unless otherwise noted. Mass spectrometry data collection took place with a Shimadzu LCMS-2020, an Agilent 1260LC-6120/6125MSD, a Shimadzu LCMS-2010EV, or a Waters Acquity UPLC, with either a QDa detector or SQ Detector 2. Samples were injected in their liquid phase onto a C-18 reverse phase. The compounds were eluted from the column using an acetonitrile gradient and fed into the mass analyzer. Initial data analysis took place with either Agilent ChemStation, Shimadzu LabSolutions, or Waters MassLynx. NMR data was collected with either a Bruker AVANCE III HD 400MHz, a Bruker Ascend 500MHz instrument, or a Varian 400MHz, and the raw data was analyzed with either TopSpin or Mestrelab Mnova. For clarity, yields have been rounded to the nearest whole number, and ^{13}C NMR data have been rounded to two decimal points.

Synthesis of Intermediate 1

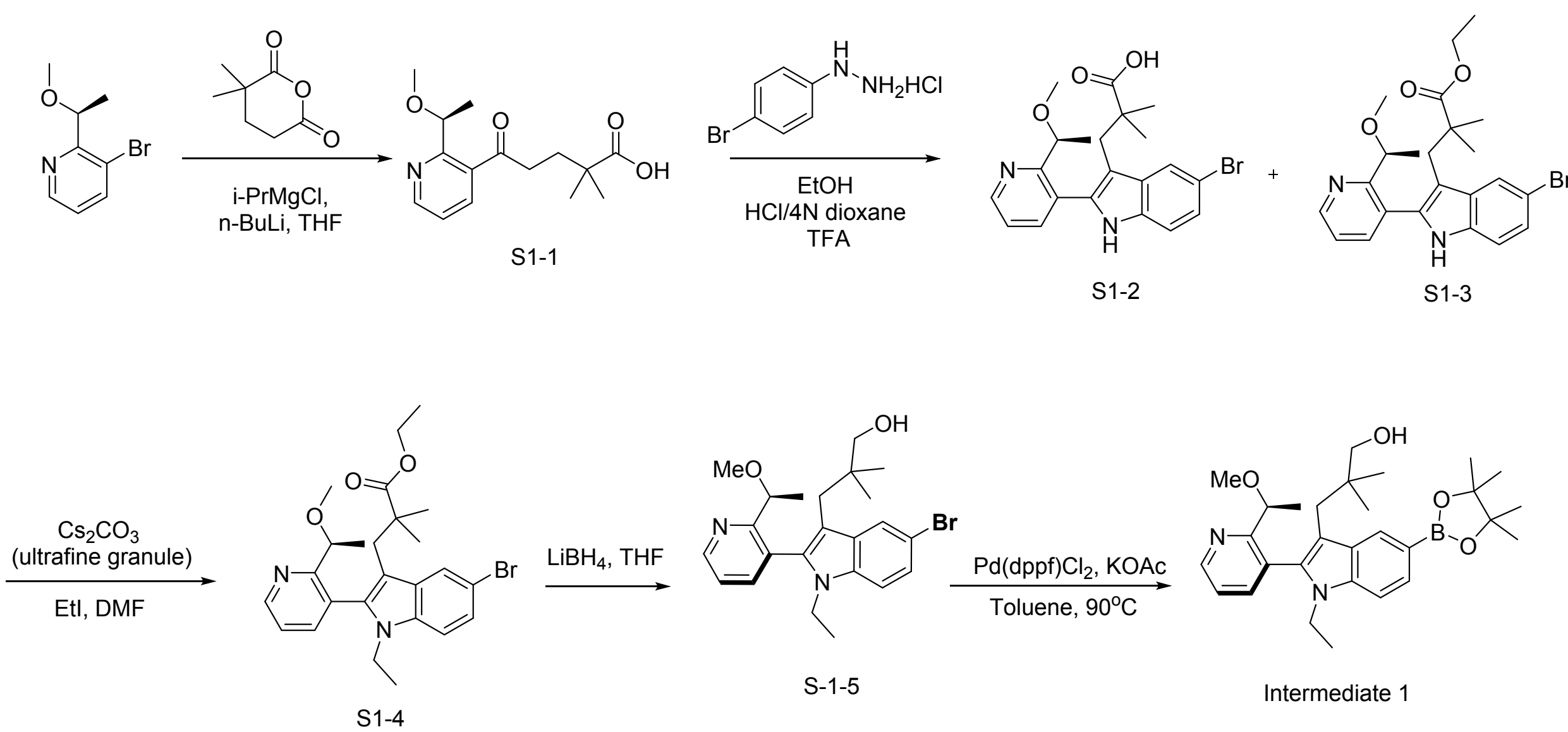

5-[2-[(1S)-1-Methoxyethyl]pyridin-3-yl]-2,2-dimethyl-5-oxopentanoic acid (S1-1). To a mixture of *i*-PrMgCl (2M in in THF, 0.5 L) at -10 °C under an atmosphere of N_2 was added *n*-BuLi, 2.5 M in hexane (333 mL, 833 mmol) dropwise over 15 min. The mixture was stirred for 30 min at -10 °C then 3-bromo-2-[(1*S*)-1-methoxyethyl]pyridine (180 g, 833 mmol) in THF (0.5 L) added dropwise over 30 min at -10 °C. The resulting mixture was warmed to -5 °C and stirred for 1 h, then 3,3-dimethyloxane-2,6-dione (118 g, 833 mmol) in THF (1.2 L) was added dropwise over 30 min at -5 °C. The mixture was warmed to 0 °C and stirred for 1.5 h, then quenched with the addition of pre-cooled 4M HCl in 1,4-dioxane (0.6 L) at 0 °C to adjust pH ~5. The mixture was diluted with ice-water (3 L) and extracted with EtOAc (3 x 2.5 L). The combined organic layers were dried over anhydrous Na_2SO_4, filtered, the filtrate

was concentrated under reduced pressure, and the residue was purified by silica gel column chromatography to give 5-[2-[(1*S*)-1-methoxyethyl]pyridin-3-yl]-2,2-dimethyl-5-oxopentanoic acid (S1-1) (87 g, 34% yield) as a solid. ^{1}H NMR (400 MHz, DMSO-d_6) δ 12.22 (s, 1H), 8.60 (dd, *J* = 4.8, 1.7 Hz, 1H), 7.89 (dd, *J* = 7.7, 1.7 Hz, 1H), 7.39 (dd, *J* = 7.7, 4.8 Hz, 1H), 4.56 (q, *J* = 6.5 Hz, 1H), 3.12 (s, 3H), 2.82 – 2.80 (m, 2H), 1.86 – 1.75 (m, 2H), 1.43 (d, *J* = 6.5 Hz, 3H), 1.13 (s, 6H); ^{13}C NMR (101 MHz, DMSO) δ 204.46, 178.94, 158.72, 149.90, 135.99, 134.91, 122.55, 78.17, 56.70, 41.03, 39.08, 34.12, 25.35, 25.29, 19.88. LCMS (ESI): m/z [M+H] calc'd for $C_{15}H_{21}NO_4$ 279.2; found 280.1.

3-(5-Bromo-2-[2-[(1S)-1-methoxyethyl]pyridin-3-yl]-1H-indol-3-yl)-2,2-dimethylpropanoic acid (S1-2) and ethyl (S)-3-(5-bromo-2-(2-(1-methoxyethyl)pyridin-3-yl)-1H-indol-3-yl)-2,2-dimethylpropanoate (S1-3) To a mixture of 5-[2-[(1*S*)-1-methoxyethyl]pyridin-3-yl]-2,2-dimethyl-5-oxopentanoic acid (78 g, 279 mmol) in EtOH (0.78 L) at rt under an atmosphere of N_2 was added (4-bromophenyl)hydrazine HCl salt (68.7 g, 307 mmol) in portions. The mixture was heated to 85 °C and stirred for 2 h, cooled to rt, then 4M HCl in 1,4-dioxane (69.8 mL, 279 mmol) added dropwise. The mixture was heated to 85 °C and stirred for an additional 3 h, then concentrated under reduced pressure, and the residue was dissolved in TFA (0.78 L). The mixture was heated to 60 °C and stirred for 1.5 h, concentrated under reduced pressure, and the residue adjusted to pH ~5 with saturated $NaHCO_3$, then extracted with EtOAc (3 x 1.5 L). The combined organic layers were dried over anhydrous Na_2SO_4, filtered, the filtrate concentrated under reduced pressure, and the residue was purified by silica gel column chromatography to give 3-(5-bromo-2-[2-[(1*S*)-1-methoxyethyl]pyridin-3-yl]-1*H*-indol-3-yl)-2,2-dimethylpropanoic acid (S1-2) and ethyl (S)-3-(5-bromo-2-(2-(1-methoxyethyl)pyridin-3-yl)-1H-indol-3-yl)-2,2-dimethylpropanoate(S1-3) (78 g, crude) as a solid. ^{1}H NMR (400 MHz, DMSO-d_6) δ 11.41 (s, 1H), 8.72 (dd, *J* = 4.8, 1.7 Hz, 1H), 7.81 (dd, *J* = 7.7, 1.8 Hz, 1H), 7.67 (d, *J* = 1.9 Hz, 1H), 7.47 (dd, *J* = 7.7, 4.7 Hz, 1H), 7.32 (d, *J* = 8.6 Hz, 1H), 7.22 (dd, *J* = 8.6, 1.9 Hz, 1H), 4.24 (q, *J* = 6.3 Hz, 1H), 3.84 – 3.78 (m, 2H), 2.92 (d, *J* = 21.6 Hz, 4H), 2.78 (d, *J* = 34.5 Hz, 1H), 1.39 (d, *J* = 6.3 Hz, 3H), 1.04 (t, *J* = 7.1 Hz, 3H), 0.92 (d, *J* = 2.7 Hz, 6H); ^{13}C NMR (101 MHz, DMSO) δ 177.11, 150.04, 139.80, 135.34, 135.04, 130.65, 128.40, 124.25, 122.73, 122.01, 113.58, 111.97, 109.55, 75.78, 60.40, 56.02, 43.78, 36.21, 34.70, 31.21, 25.67, 25.58, 14.16. LCMS (ESI): m/z [M+H] calc'd for $C_{21}H_{23}BrN_2O_3$ 430.1 and $C_{23}H_{27}BrN_2O_3$ 458.1; found 431.1 and 459.1. ^{1}H NMR (400 MHz, DMSO-d_6) δ 11.49 (d, *J* = 2.8 Hz, 1H), 8.75 (dd, *J* = 4.9, 1.7 Hz, 1H), 7.95 (d, *J* = 7.7 Hz, 1H), 7.79 (d, *J* = 1.9 Hz, 1H), 7.56 (dd, *J* = 7.8, 4.9 Hz, 1H), 7.36 – 7.20 (m, 2H), 4.26 (q, *J* = 6.3 Hz, 1H), 2.99 (s, 3H), 2.94 – 2.69 (m, 2H), 1.36 (d, *J* = 6.3 Hz, 3H), 0.90 (d, *J* = 4.0 Hz, 6H); ^{13}C NMR (101 MHz, DMSO) δ 179.26, 158.47, 148.83, 141.67, 135.17, 134.45, 130.74, 128.94, 124.44, 123.41, 122.50, 113.67, 112.11, 110.25, 75.53, 56.38, 43.57, 34.47, 25.90, 25.63, 21.19.

Ethyl 3-(5-bromo-1-ethyl-2-[2-[(1S)-1-methoxyethyl]pyridin-3-yl]indol-3-yl)-2,2-dimethylpropanoate (S1-4) To a mixture of 3-(5-bromo-2-[2-[(1*S*)-1-methoxyethyl]pyridin-3-yl]-1*H*-indol-3-yl)-2,2-dimethylpropanoic acid and ethyl (S)-3-(5-bromo-2-(2-(1-methoxyethyl)pyridin-3-yl)-1*H*-indol-3-yl)-2,2-dimethylpropanoate (198 g, 459 mmol) in DMF (1.8 L) at 0 °C under an atmosphere of N_2 was added Cs_2CO_3 (449 g, 1.38 mol) in portions. EtI (215 g, 1.38 mmol) in DMF (200 mL) was then added dropwise at 0 °C. The mixture was warmed to rt and stirred for 4 h then diluted with brine (5 L) and extracted with EtOAc (3 x 2.5 L). The combined organic layers were washed with brine (2 x 1.5 L), dried over anhydrous Na_2SO_4, and filtered. The filtrate was concentrated under reduced pressure and the residue was purified by silica gel column chromatography to give ethyl 3-(5-bromo-1-ethyl-2-[2-[(1*S*)-1-methoxyethyl]pyridin-3-yl]indol-3-yl)-2,2-dimethylpropanoate (160 g, 57% yield) as a solid. ^{1}H NMR (400 MHz, Chloroform-*d*) δ 8.84 – 8.82 (m, 1H), 7.73 (dd, *J* = 7.3, 1.9 Hz, 1H), 7.72– 7.65 (m, 1H), 7.41 – 7.29 (m, 2H), 7.24 – 7.22 (m, 1H), 4.17 – 3.92 (m, 4H), 3.90 – 3.75 (m, 1H), 3.29 – 3.04 (m, 4H), 2.60 (dd, *J* = 63.6, 14.4 Hz, 1H), 1.48 (d, *J* = 6.3 Hz, 1.6H), 1.27 (d, *J* = 6.4 Hz, 1.8H), 1.23 – 1.14 (m, 6H), 1.06 – 0.99 (m, 6H); ^{13}C NMR (101 MHz, DMSO) δ 177.27, 177.08, 160.05, 159.57, 150.70, 150.46, 140.89, 140.72, 136.91, 136.51, 134.70, 130.17, 130.10, 126.64, 126.05, 124.37, 124.33, 122.92, 122.66, 122.33, 122.24, 112.44, 112.26, 112.24, 110.05, 109.83, 75.84, 75.76, 60.48, 56.23, 56.03, 43.61, 43.54, 38.88, 35.19, 34.81, 26.55, 26.14, 25.31, 25.13, 18.79, 17.86, 15.46, 15.15, 14.19, 14.16. LCMS (ESI): m/z [M+H] calc'd for $C_{25}H_{31}BrN_2O_3$ 486.2; found 487.2.

3-(5-Bromo-1-ethyl-2-(2-((S)-1-methoxyethyl)pyridin-3-yl)-1H-indol-3-yl)-2,2-dimethylpropan-1-ol (S1-5) To a mixture of ethyl 3-(5-bromo-1-ethyl-2-[2-[(1*S*)-1-methoxyethyl]pyridin-3-yl]indol-3-yl)-2,2-dimethylpropanoate (160 g, 328 mmol) in THF (1.6 L) at 0 °C under a nitrogen atmosphere was added $LiBH_4$ (28.6 g, 1.3 mol). The mixture was heated to 60 °C for 16 h, cooled, and quenched with pre-cooled (0 °C) aqueous NH_4Cl (5 L). The mixture was extracted with EtOAc (3 x 2 L) and the combined organic layers were washed with brine (2 x 1 L), dried over anhydrous Na_2SO_4, and filtered. The filtrate was concentrated under reduced pressure and the residue was purified by silica gel column chromatography to give to two atropisomers (as single atropisomers) of 3-(5-bromo-1-ethyl-2-(2-((*S*)-1-methoxyethyl)pyridin-3-yl)-1*H*-indol-3-yl)-2,2-dimethylpropan-1-ol (60 g, 38% yield) and (40 g, 26% yield) both as solids. ^{1}H NMR (400 MHz, DMSO-d_6) δ 8.74 (dd, *J* = 4.8, 1.7 Hz, 1H), 7.93 – 7.80 (m, 2H), 7.55 – 7.45 (m, 2H), 7.28 (dd, *J* = 8.7, 1.8 Hz, 1H), 4.50 (t, *J* = 5.2 Hz, 1H), 4.18 – 3.98 (m, 2H), 3.84 – 3.80 (m, 1H), 3.05 (dd, *J* = 10.2, 5.3 Hz, 1H), 2.96 (dd, *J* = 10.2, 5.2 Hz, 1H), 2.85 (d, *J* = 0.6 Hz, 3H), 2.65 (d, *J* = 13.9 Hz, 1H), 2.12 (d, *J* = 14.0 Hz, 1H), 1.39 (d, *J* = 6.3 Hz, 3H), 1.10 (t, *J* = 7.1 Hz, 3H), 0.58 (d, *J* = 9.9 Hz, 6H); ^{13}C NMR (101 MHz, DMSO) δ 159.64, 150.30, 140.81, 136.55, 134.67, 131.00, 127.09, 124.21, 122.84, 122.72, 112.21, 112.13, 111.28, 75.83, 70.30, 56.03, 38.82, 38.16, 33.20, 25.37, 25.03, 17.90, 15.21. LCMS (ESI): m/z [M+H] calc'd for $C_{23}H_{29}BrN_2O_2$ 444.1; found 445.2.

(S)-3-(1-Ethyl-2-(2-(1-methoxyethyl)pyridin-3-yl)-5-(4,4,5,5-tetramethyl-1,3,2-dioxaborolan-2-yl)-1H-indol-3-yl)-2,2-dimethylpropan-1-ol (Intermediate 1) To a stirred solution of (S)-3-(5-bromo-1-ethyl-2-(2-(1-methoxyethyl)pyridin-3-yl)-1*H*-indol-3-yl)-2,2-dimethylpropan -1-ol (16.00 g, 35.92 mmol) in toluene (160.00 mL) was added 4,4,4',4',5,5,5',5'-octamethyl-2,2'-bi (1,3,2-dioxaborolane) (13.68 g, 53.88 mmol), KOAc (7.76 g, 79.03 mmol) and Pd(dppf)Cl_2 (2.63 g, 3.59 mmol) at room temperature under argon atmosphere. The resulting mixture was stirred for 3 h at 90 °C. After filtration, the filtrate was concentrated under reduced pressure. The residue was purified by silica gel column chromatography to afford (*S*)-3-(1-ethyl-2-(2-(1-methoxyethyl)pyridin-3-yl)-5-(4,4,5,5-tetramethyl-1,3,2-dioxaborolan-2-yl)-1*H*-indol-3-yl)-2,2-dimethylpropan-1-ol (13 g, 66% yield) as a solid. ^{1}H NMR (400 MHz, DMSO-d_6) δ 8.75 – 8.73 (m, 1H), 8.03 (s, 1H), 7.86 (dd, *J* = 7.7, 1.8 Hz, 1H), 7.54 – 7.47 (m, 3H), 4.46 (t, *J* = 5.4 Hz, 1H), 4.11 – 4.01 (m, 2H), 3.81 (dd, *J* = 14.7, 7.3 Hz, 1H), 3.06 (dd, *J* = 10.3, 5.5 Hz, 1H), 2.97 (dd, *J* = 10.3, 5.4 Hz, 1H), 2.83 (s, 3H), 2.69 (d, *J* = 14.1 Hz, 1H), 2.18 (d, *J* = 14.0 Hz, 1H), 1.39 (d, *J* = 6.2 Hz, 3H), 1.32 (d, *J* = 1.9 Hz, 12H), 1.11 (t, *J* = 7.1 Hz, 3H), 0.64 (s, 3H), 0.54 (s, 3H); ^{13}C NMR (101 MHz, DMSO) δ 170.66, 159.70, 150.13, 140.88, 138.01, 135.20, 128.89, 127.93, 127.44, 122.80, 112.05, 109.60, 83.50, 75.75, 71.14, 60.16, 56.00, 38.60, 38.32, 33.54, 25.41, 25.23, 25.09, 25.07, 25.02, 21.14, 17.92, 15.17, 14.50. LCMS (ESI): m/z [M+H] calc'd for $C_{29}H_{41}BN_2O_4$ 492.3; found 493.2.

Synthesis of Intermediate Macrocycle 1

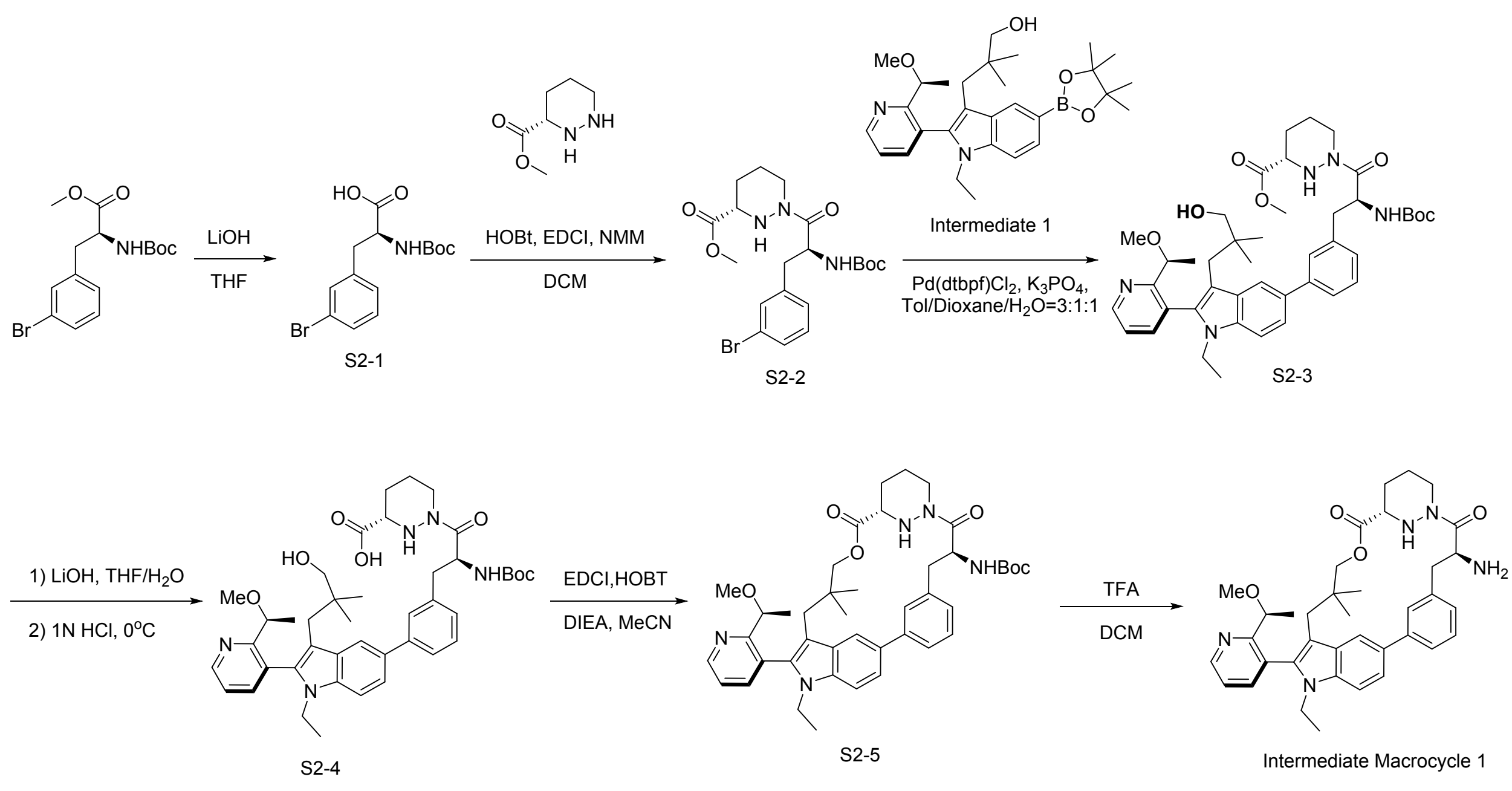

(2S)-3-(3-Bromophenyl)-2-[(tert-butoxycarbonyl)amino]propanoic acid (S2-1) To a stirred solution of methyl (2*S*)-3-(3-bromophenyl)-2-[(*tert*-butoxycarbonyl)amino]propanoate (2 g, 5.58 mmol) in THF (5 mL) was added LiOH·H_2O (702.78 mg, 16.75 mmol) in H_2O (5 mL) dropwise at 0 °C. The

resulting reaction was stirred overnight at room temperature then acidified to pH 5~6 with HCl (aq.). The resulting mixture was extracted with EtOAc (3 x 5 mL). The combined organic layers were dried over anhydrous Na_2SO_4. After filtration, the filtrate was concentrated under reduced pressure to afford (2*S*)-3-(3-bromophenyl)-2-[(*tert*-butoxycarbonyl)amino]propanoic acid (2.2 g, crude) as a solid.^{1}H NMR (400 MHz, DMSO-d_6) δ 7.46 – 7.36 (m, 2H), 7.24 (d, *J* = 6.2 Hz, 2H), 6.98 (d, *J* = 8.3 Hz, 1H),4.10 – 4.07 (m, 1H), 3.04 (dd, *J* = 13.7, 4.5 Hz, 1H), 2.81 (dd, *J* = 13.7, 10.2 Hz, 1H), 1.32 (s, 9H); ^{13}C NMR (101 MHz, DMSO) δ 173.79, 155.81, 141.36, 132.37, 130.61, 129.62, 128.65, 121.85, 78.53, 55.26, 36.56, 28.57.; LCMS (ESI): m/z [M+H] calc'd for $C_{14}H_{18}BrNO_4$ 343.3; found 344.2.

Methyl (3S)-1-[(2S)-3-(3-bromophenyl)-2-[(tert-butoxycarbonyl)amino]propanoyl]-1,2-diazinane-3-carboxylate (S2-2) To a stirred solution of methyl (3*S*)-1,2-diazinane-3-carboxylate (942.44 mg, 6.53 mmol) in DCM (15 mL) was added NMM (1.44 mL, 13.07 mmol) dropwise at 0 °C and stirred for 5 min. To the above mixture was added (2S)-3-(3-bromophenyl)-2-[(*tert*-butoxycarbonyl)amino]propanoic acid (1.5 g, 4.36 mmol), HOBt (176.66 mg, 1.307 mmol), and EDCI (1.67 g, 8.716 mmol) at 0 °C. The resulting mixture was stirred for 1 h at room temperature then concentrated under reduced pressure to remove the majority of the DCM. The desired product was purified by trituration with DCM (5 mL). The precipitated solids were collected by filtration and washed with DCM (3 x 5 mL). This resulted in methyl (3*S*)-1-[(2*S*)-3-(3-bromophenyl)-2-[(*tert*-butoxycarbonyl)amino]propanoyl]-1,2-diazinane-3-carboxylate (1.5 g, 73% yield) as a solid. ^{1}H NMR (400 MHz, DMSO-d_6) δ 7.41 – 7.39 (m, 2H), 7.26 – 7.23 (m, 2H), 6.69 (d, *J* = 8.8 Hz, 1H), 5.28 (d, *J* = 10.3 Hz, 1H), 5.09 – 5.04 (m, 1H), 3.91 (d, *J* = 22.3 Hz, 1H), 3.70 (s, 3H), 3.36 – 3.35 (m, 1H), 2.99 (s, 1H), 2.85 (dd, *J* = 13.3, 4.0 Hz, 1H), 2.63 (dd, *J* = 13.4, 9.4 Hz, 1H), 1.89 – 1.80 (m, 1H), 1.72 (d, *J* = 11.7 Hz, 1H), 1.61 (d, *J* = 11.3 Hz, 1H), 1.51 (s, 1H), 1.31 (s, 9H); ^{13}C NMR (101 MHz, DMSO) δ 172.48, 171.84, 155.42, 141.91, 132.29, 130.63, 129.56, 128.76, 121.84, 78.32, 58.45, 52.33, 41.02, 37.54, 28.58, 28.21, 27.27, 22.80.; LCMS (ESI): m/z [M+H] calc'd for $C_{20}H_{28}BrN_3O_5$ 469.3; found 470.3.

(S)-1-((S)-2-((tert-Butoxycarbonyl)amino)-3-(3-(1-ethyl-3-(3-hydroxy-2,2-dimethylpropyl)-2-(2-((S)-1-methoxyethyl)pyridin-3-yl)-1H-indol-5-yl)phenyl)propanoyl)hexahydropyridazine-3-carboxylate (S2-3) A mixture of methyl (3*S*)-1-[(2*S*)-3-(3-bromophenyl)-2-[(*tert*-butoxycarbonyl)amino]propanoyl]-1,2-diazinane-3-carboxylate (867 mg, 1.84 mmol), 3-[(2M)-1-ethyl-2-{2-[(1*S*)-1-methoxyethyl]pyridin-3-yl}-5-(4,4,5,5-tetramethyl-1,3,2-dioxaborolan-2-yl)indol-3-yl]-2,2-dimethylpropan-1-ol (1.00 g, 2.03 mmol), Pd(dtbpf)Cl_2 (240.27 mg, 0.37 mmol) and K_3PO_4 (0.12 g, 0.55 mmol) in toluene (9 mL), dioxane (3 mL) and H_2O (3 mL) was stirred for 1 h at 80 °C under a nitrogen atmosphere. The reaction was cooled to 0 °C and quenched by the addition of H_2O (10 mL). The resulting mixture was extracted with EtOAc (3 x 8 mL). The combined organic layers were dried over anhydrous Na_2SO_4. After filtration, the filtrate was

concentrated under reduced pressure. The residue was purified by reverse phase chromatography to afford methyl (*S*)-1-((*S*)-2-((*tert*-butoxycarbonyl)amino)-3-(3-(1-ethyl-3-(3-hydroxy-2,2-dimethylpropyl)-2-(2-((S)-1-methoxyethyl)pyridin-3-yl)-1*H*-indol-5-yl)phenyl)propanoyl)hexahydropyridazine-3-carboxylate (312 mg, 22% yield) as a solid. ^{1}H NMR (400 MHz, DMSO-d_6) δ 8.76 – 8.74 (m, 1H), 7.98 – 7.80 (m, 2H), 7.62 – 7.48 (m, 4H), 7.47 – 7.30 (m, 2H), 7.15 (d, *J* = 7.5 Hz, 1H), 6.65 (s, 1H), 5.32 – 5.09 (m, 2H), 4.46 (d, *J* = 5.4 Hz, 1H), 4.20 – 3.92 (m, 3H), 3.89 – 3.72 (m, 1H), 3.59 (s, 3H), 3.25 – 3.06 (m, 2H), 3.04 – 2.90 (m, 3H), 2.88 (s, 3H), 2.77 (dd, *J* = 24.6, 11.4 Hz, 2H), 2.20 (d, *J* = 14.1 Hz, 1H), 1.80 (s, 1H), 1.67 (s, 1H), 1.58 (s, 1H), 1.41 (d, *J* = 6.3 Hz, 4H), 1.31 (s, 7H), 1.21 (s, 1H), 1.15 (t, *J* = 7.2 Hz, 3H), 0.63 (d, *J* = 23.6 Hz, 6H); ^{13}C NMR (101 MHz, DMSO) δ 172.86, 171.91, 159.76, 155.45, 150.26, 142.39, 140.94, 139.20, 135.67, 132.19, 129.74, 129.04, 128.50, 127.74, 125.29, 122.90, 121.32, 118.80, 112.08, 110.52, 78.27, 75.82, 70.71, 58.43, 56.09, 52.14, 38.77, 38.38, 33.54, 28.68, 27.48, 25.39, 25.22, 22.93, 18.16, 15.39.; LCMS (ESI): m/z [M+H] calc'd for $C_{43}H_{57}N_5O_7$ 755.3; found 756.3.

(S)-1-((S)-2-((tert-Butoxycarbonyl)amino)-3-(3-(1-ethyl-3-(3-hydroxy-2,2-dimethylpropyl)-2-(2-((S)-1-methoxyethyl)pyridin-3-yl)-1H-indol-5-yl)phenyl)propanoyl)hexahydropyridazine-3-carboxylic acid (S2-4) To a stirred solution of methyl (*S*)-1-((*S*)-2-((*tert*-butoxycarbonyl)amino)-3-(3-(1-ethyl-3-(3-hydroxy-2,2-dimethylpropyl)-2-(2-((*S*)-1-methoxyethyl)pyridin-3-yl)-1*H*-indol-5-yl)phenyl)propanoyl)hexahydropyridazine-3-carboxylate (200 mg, 0.27 mmol) in THF (4 mL) was added $LiOH{\cdot}H_2O$ (33.30 mg, 0.78 mmol) in H_2O (4 mL) dropwise at 0 °C. The resulting reaction was stirred for 1 h at room temperature then acidified to pH 5~6 with HCl (aq.). The resulting mixture was extracted with EtOAc (3 x 5 mL). The combined organic layers were dried over anhydrous Na_2SO_4. After filtration, the filtrate was concentrated under reduced pressure to afford (*S*)-1-((*S*)-2-((*tert*-butoxycarbonyl)amino)-3-(3-(1-ethyl-3-(3-hydroxy-2,2-dimethylpropyl)-2-(2-((*S*)-1-methoxyethyl)pyridin-3-yl)-1*H*-indol-5-yl)phenyl)propanoyl)hexahydropyridazine-3-carboxylic acid (228 mg, crude) as a solid. The crude product was used in the next step directly without further purification. ^{1}H NMR (400 MHz, DMSO-d_6) δ 12.72 (s, 1H), 8.75 (dd, *J* = 4.7, 1.7 Hz, 1H), 7.93 (s, 1H), 7.86 (dd, *J* = 7.7, 1.8 Hz, 1H), 7.57 (d, *J* = 8.6 Hz, 1H), 7.54 – 7.48 (m, 3H), 7.44 (dd, *J* = 8.6, 1.7 Hz, 1H), 7.34 (t, *J* = 7.7 Hz, 1H), 7.16 (d, *J* = 7.6 Hz, 1H), 6.64 (d, *J* = 8.7 Hz, 1H), 5.27 – 5.15 (m, 1H), 5.04 (d, *J* = 11.2 Hz, 1H), 4.49 (s, 1H), 4.15 – 4.12 (m, 3H), 3.83 (dd, *J* = 14.6, 7.3 Hz, 1H), 3.10 (d, *J* = 10.4 Hz, 2H), 3.04 – 2.92 (m, 2H), 2.89 (s, 3H), 2.84 – 2.69 (m, 2H), 2.21 (d, *J* = 14.1 Hz, 1H), 1.86 (d, *J* = 11.2 Hz, 1H), 1.70 (d, *J* = 11.9 Hz, 1H), 1.41 (d, *J* = 6.2 Hz, 5H), 1.31 (s, 7H), 1.29 – 1.17 (m, 4H), 0.66 (s, 3H), 0.60 (s, 3H); ^{13}C NMR (101 MHz, DMSO) δ 173.21, 172.70, 159.73, 155.45, 150.25, 142.27, 140.92, 139.19, 135.62, 135.58, 132.12, 129.68, 129.03, 128.44, 127.65, 127.48, 125.24, 122.85, 121.30, 118.76, 112.06, 110.56, 78.24, 75.74, 70.69, 58.60, 56.04, 52.27, 41.00, 38.72, 38.31, 38.19, 33.47, 28.64, 28.33, 27.96, 25.34, 25.19, 23.31, 18.19, 15.37.; LCMS (ESI): m/z [M+H] calc'd for $C_{42}H_{55}N_5O_7$ 741.3; found 742.3.

tert-Butyl ((6^{3}S,4S)-11-ethyl-1^2-(2-((S)-1-methoxyethyl)pyridin-3-yl)-10,10-dimethyl-5,7-dioxo-6^1,6^2,6^3,6^4,6^5,6^6-hexahydro-1^{1}H-8-oxa-1(5,3)-indola-6(1,3)-pyridazina-2(1,3)-benzenacycloundecaphane-4-yl)carbamate (S2-5) To a stirred solution of (*S*)-1-((*S*)-2-((*tert*-butoxycarbonyl)amino)-3-(3-(1-ethyl-3-(3-hydroxy-2,2-dimethylpropyl)-2-(2-((*S*)-1-methoxyethyl)pyridin-3-yl)-1*H*-indol-5-yl)phenyl)propanoyl)hexahydropyridazine-3-carboxylic acid (40 g, 53.91 mmol) and DIPEA (209.04 g, 1617.39 mmol) in DCM (3 L) was added HOBT (36.42 g, 269.57 mmol) and EDCI (289.39 g, 1509.57 mmol) in portions at 0 °C. The resulting mixture was stirred for 16 h at room temperature then washed with H_2O (3 x 1 L). The combined organic layers were concentrated under reduced pressure and purified by silica gel column chromatography to afford *tert*-butyl ((6^3*S*,4*S*)-11-ethyl-1^2-(2-((*S*)-1-methoxyethyl)pyridin-3-yl)-10,10-dimethyl-5,7-dioxo-6^1,6^2,6^3,6^4,6^5,6^6-hexahydro-1^1*H*-8-oxa-1(5,3)-indola-6(1,3)-pyridazina-2(1,3)-benzenacycloundecaphane-4-yl)carbamate (30 g, 73.79%) as a solid. ^{1}H NMR (400 MHz, DMSO-d_6) δ 8.78 – 8.76 (m, 1H), 8.00 (s, 1H), 7.92 – 7.81 (m, 2H), 7.75 – 7.65 (m, 2H), 7.61 (d, *J* = 8.7 Hz, 1H), 7.54 (dd, *J* = 7.7, 4.7 Hz, 1H), 7.32 (t, *J* = 7.7 Hz, 1H), 7.16 (d, *J* = 7.5 Hz, 1H), 7.07 (d, *J* = 8.7 Hz, 1H), 5.38 (d, *J* = 12.2 Hz, 1H), 5.07 (dd, *J* = 9.9, 7.6 Hz, 1H), 4.36 – 4.13 (m, 3H), 4.07 – 3.88 (m, 2H), 3.74 – 3.57 (m, 2H), 3.44 (s, 4H), 2.88 – 2.78 (m, 4H), 2.01 (d, *J* = 12.0 Hz, 1H), 1.82 (d, *J* = 12.4 Hz, 1H), 1.73 – 1.50 (m, 2H), 1.40 (d, *J* = 6.2 Hz, 3H), 1.33 (s, 7H), 1.15 (s, 2H), 0.98 (t, *J* = 7.1 Hz, 3H), 0.74 (s, 3H), 0.58 (s, 3H); ^{13}C NMR (101 MHz, DMSO) δ 173.42, 171.97, 159.31, 155.85, 150.06, 141.28, 140.99, 140.18, 136.61, 135.79, 130.36, 130.15, 129.50, 129.09, 128.03, 127.36, 125.46, 123.78, 123.49, 120.06, 115.97, 111.51, 111.19, 110.23, 78.14, 75.96, 70.98, 59.51, 56.16, 53.92, 38.45, 38.29, 35.96, 32.77, 31.76, 28.71, 25.91, 25.37, 17.24, 15.14.; LCMS (ESI): m/z [M+H] calc'd for $C_{42}H_{53}N_5O_6$ 723.3; found 724.3.

((6^{3}S,4S)-4-Amino-1^1-ethyl-1^2-(2-((S)-1-methoxyethyl)pyridin-3-yl)-10,10-dimethyl-6^1,6^2,6^3,6^4,6^5,6^6-hexahydro-1^{1}H-8-oxa-1(5,3)-indola-6(1,3)-pyridazina-2(1,3)-benzenacycloundecaphane-5,7-dione (Intermediate macrocycle 1) To a solution of *tert*-butyl ((6^3*S*,4*S*)-11-ethyl-1^2-(2-((*S*)-1-methoxyethyl)pyridin-3-yl)-10,10-dimethyl-5,7-dioxo-6^1,6^2,6^3,6^4,6^5,6^6-hexahydro-1^1*H*-8-oxa-1(5,3)-indola-6(1,3)-pyridazina-2(1,3)-benzenacycloundecaphane-4-yl)carbamate (5 g, 6.907 mmol) in dioxane (20 mL) was added HCl(gas) in 1,4-dioxane (30 mL) at 0 °C. The reaction was stirred for 1 h at 0 °C then the resulting mixture was concentrated under reduced pressure to afford ((6^3*S*,4*S*)-4-amino-1^1-ethyl-1^2-(2-((*S*)-1-methoxyethyl)pyridin-3-yl)-10,10-dimethyl-6^1,6^2,6^3,6^4,6^5,6^6-hexahydro-1^1*H*-8-oxa-1(5,3)-indola-6(1,3)-pyridazina-2(1,3)-benzenacycloundecaphane-5,7-dione (5 g, 99% yield) as an oil which was used in the next step directly without further purification. ^{1}H NMR (400 MHz, DMSO-d_6) δ 8.78 – 8.76 (m, 1H), 8.46 (s, 2H), 7.87 –7.84 (m, 2H), 7.70 – 7.52 (m, 4H), 7.41 (t, *J* = 7.6 Hz, 1H), 7.17 (d, *J* = 7.5 Hz, 1H), 5.49 (d, *J* = 11.9 Hz, 1H), 4.81 (dd, *J* = 7.9, 3.6 Hz, 1H), 4.34 – 4.15 (m, 2H), 4.22 – 4.00 (m, 1H), 3.66 – 3.56 (m, 2H), 3.21 – 3.12 (m, 1H), 3.07 (s, 2H), 2.99 (dd, *J* = 14.7, 7.9 Hz, 1H), 2.90 – 2.69

(m, 3H), 2.57 (d, J = 13.8 Hz, 1H), 1.96 (d, J = 7.9 Hz, 1H), 1.87 – 1.77 (m, 1H), 1.57 (d, J = 9.9 Hz, 2H), 1.48 – 1.33 (m, 5H), 1.22 (d, J = 9.8 Hz, 1H), 1.21 – 1.00 (m, 3H), 0.90 – 0.76 (m, 2H), 0.72 (s, 2H), 0.59 (s, 2H); ^{13}C NMR (101 MHz, DMSO) δ 171.50, 169.64, 163.53, 159.37, 150.27, 141.81, 140.62, 137.81, 136.49, 135.64, 131.08, 129.93, 129.45, 127.89, 127.27, 126.45, 125.21, 123.35, 120.44, 117.26, 111.39, 111.01, 76.02, 71.25, 58.87, 56.10, 51.85, 41.18, 38.50, 37.48, 35.83, 32.80, 29.48, 28.60, 28.48, 25.74, 25.56, 23.04, 19.83, 18.99, 17.36, 15.16, 14.37, 1.60.; LCMS (ESI): m/z [M+H] calc'd for $C_{37}H_{45}N_5O_4$ 623.3; found 624.3.

Synthesis of Compound 1

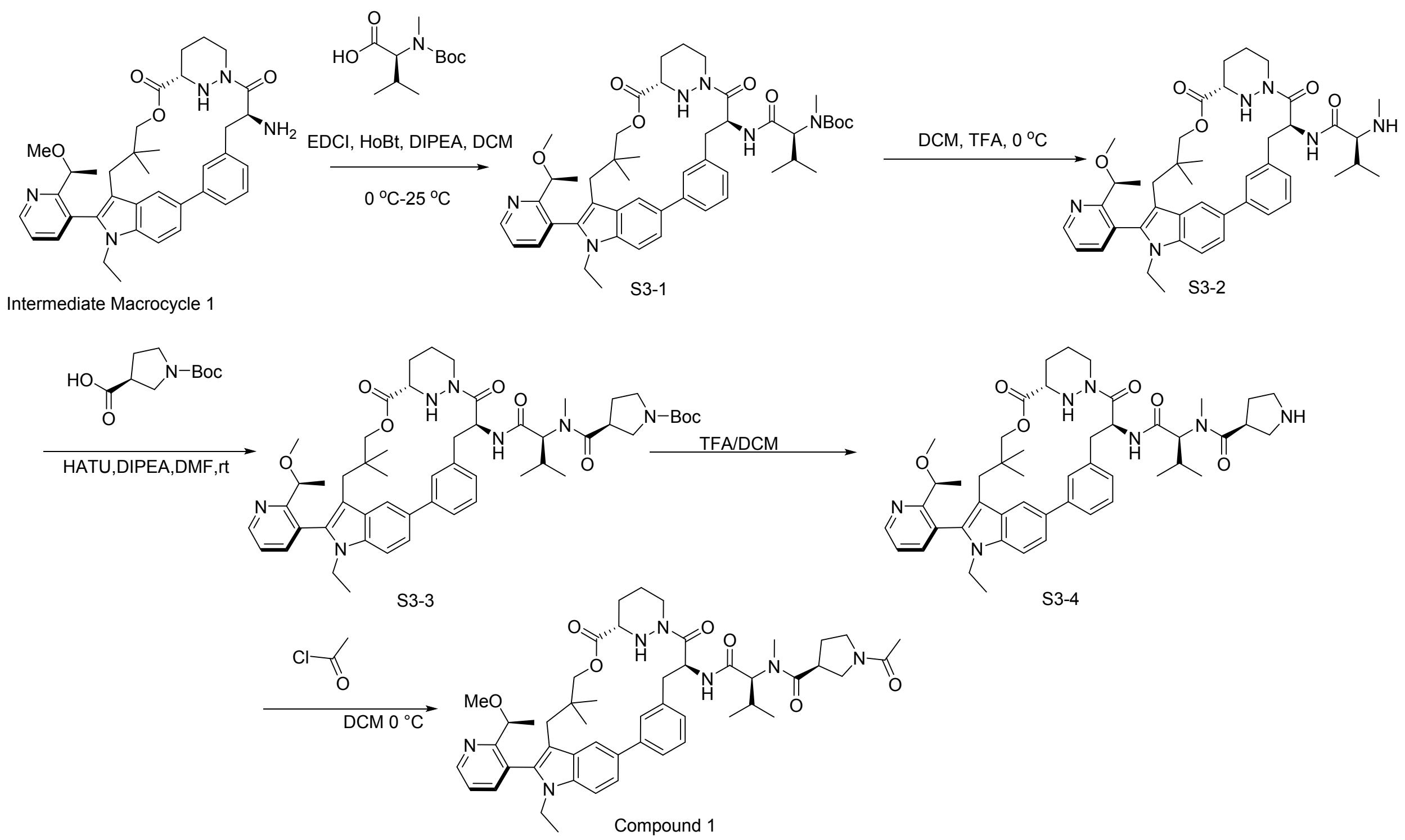

tert-Butyl ((2S)-1-(((6³S,4S)-1¹-ethyl-1²-(2-((S)-1-methoxyethyl) pyridin-3-yl)-10,10-dimethyl-5,7-dioxo-6¹,6²,6³,6⁴,6⁵,6⁶-hexahydro-1¹H-8-oxa-1(5,3)-indola-6(1,3)-pyridazina-2(1,3)-benzenacycloundecaphane-4-yl) amino)-3-methyl-1-oxobutan-2-yl) (methyl)carbamate (S3-1) To a stirred solution of (6³*S*,4*S*)-4-amino-1¹-ethyl-1²-(2-((*S*)-1-methoxyethyl) pyridin-3-yl)-10,10-dimethyl-6¹,6²,6³,6⁴,6⁵,6⁶-hexahydro-1¹*H*-8-oxa-1(5,3)-indola-6(1,3)-pyridazina-2(1,3)-benzenacycloundecaphane-5,7-dione (5 g, 8.02 mmol) and (2S)-2-[(*tert*-butoxycarbonyl)(methyl)amino]-3-methylbutanoic acid (2.22 g, 9.62 mmol) in DMF (50.00 mL) was added HATU (6.10 g, 16.03 mmol) followed by DIPEA (10.36 g, 80.15 mmol) dropwise at 0 °C. The resulting reaction was stirred for 1 h then quenched with H_2O (50 mL). The resulting mixture was extracted with EtOAc (2 x 50 mL). The combined organic layers were washed with brine (3 x 100 mL) and dried over anhydrous Na_2SO_4. After filtration, the filtrate was

concentrated under reduced pressure. The residue was purified by silica gel column chromatography, eluted with DCM / MeOH (10:1) to afford *tert*-butyl ((2*S*)-1-(((6^3*S*,4*S*)-1^1-ethyl-1^2-(2-((*S*)-1-methoxyethyl) pyridin-3-yl)-10,10-dimethyl-5,7-dioxo-6^1,6^2,6^3,6^4,6^5,6^6-hexahydro-1^1*H*-8-oxa-1(5,3)-indola-6(1,3)-pyridazina-2(1,3)-benzenacycloundecaphane-4-yl) amino)-3-methyl-1-oxobutan-2-yl) (methyl)carbamate (5 g, 67.07%) as a solid. ^{1}H NMR (400 MHz, DMSO-d_6) δ 8.77 (dd, *J* = 4.7, 1.7 Hz, 1H), 8.14 (s, 1H), 8.00 (s, 1H), 7.83 (dd, *J* = 7.7, 1.8 Hz, 2H), 7.72 (d, *J* = 8.7 Hz, 1H), 7.67 (d, *J* = 7.8 Hz, 1H), 7.62 (d, *J* = 8.6 Hz, 1H), 7.53 (dd, *J* = 7.7, 4.7 Hz, 1H), 7.23 (t, *J* = 7.7 Hz, 1H), 7.15 (d, *J* = 7.5 Hz, 1H), 5.32 (s, 2H), 4.34 – 4.18 (m, 4H), 4.08 (s, 1H), 3.69 (d, *J* = 10.9 Hz, 1H), 3.61 (d, *J* = 10.9 Hz, 1H), 3.15 (s, 3H), 2.88 (d, *J* = 11.1 Hz, 2H), 2.77 (s, 2H), 2.65 (s, 4H), 2.05 (s, 1H), 1.83 (d, *J* = 12.3 Hz, 1H), 1.69 (d, *J* = 12.1 Hz, 1H), 1.56 (d, *J* = 13.2 Hz, 1H), 1.47 (s, 9H), 1.39 (d, *J* = 6.1 Hz, 3H), 0.93 (dd, *J* = 15.7, 8.0 Hz, 6H), 0.79 (s, 6H), 0.50 (s, 3H); ^{13}C NMR (101 MHz, DMSO) δ 170.79, 170.63, 169.99, 168.29, 157.36, 153.98, 153.35, 148.15, 138.97, 138.55, 138.27, 134.89, 133.77, 128.36, 128.20, 126.98, 125.21, 124.88, 123.77, 121.86, 121.37, 118.07, 116.34, 113.98, 109.45, 109.13, 77.39, 77.23, 74.05, 68.79, 62.22, 61.15, 54.05, 49.85, 33.89, 30.71, 27.97, 27.55, 27.19, 26.53, 25.27, 24.79, 24.02, 23.07, 21.39, 17.77, 17.32, 17.06, 14.85, 13.01.; LCMS (ESI): m/z [M+H] calc'd for $C_{48}H_{64}N_6O_7$ 836.3; found 837.2.

(2S)-N-((6^{3}S,4S)-1^1-Ethyl-1^2-(2-((S)-1-methoxyethyl) pyridin-3-yl)-10,10-dimethyl-5,7-dioxo-6^1,6^2,6^3,6^4,6^5,6^6-hexahydro-1^{1}H-8-oxa-1(5,3)-indola-6(1,3)-pyridazina-2(1,3)-benzenacycloundecaphane-4-yl)-3-methyl-2-(methylamino) butanamide (S3-2) To a solution of *tert*-butyl ((2*S*)-1-(((6^3*S*,4*S*)-1^1-ethyl-1^2-(2-((*S*)-1-methoxyethyl) pyridin-3-yl)-10,10-dimethyl-5,7-dioxo-6^1,6^2,6^3,6^4,6^5,6^6-hexahydro-1^{1}H-8-oxa-1(5,3)-indola-6(1,3)-pyridazina-2(1,3)-benzenacycloundecaphane-4-yl) amino)-3-methyl-1-oxobutan-2-yl) (methyl)carbamate (5 g, 5.97 mmol) in DCM (30 mL) was added TFA (10 mL, 134.63 mmol) dropwise and the reaction stirred for 1 h at 0 °C. The resulting mixture was concentrated under reduced pressure. The residue was dissolved in EtOAc (50 mL) and neutralized to pH 9 with saturated $NaHCO_3$ (100 mL). The resulting mixture was extracted with EtOAc (3 x 30 mL). The combined organic layers were washed with brine (1 x 100 mL) and dried over anhydrous Na_2SO_4. After filtration, the filtrate was concentrated under reduced pressure. This resulted in (2*S*)-N-((6^3*S*,4*S*)-1^1-ethyl-1^2-(2-((*S*)-1-methoxyethyl) pyridin-3-yl)-10,10-dimethyl-5,7-dioxo-6^1,6^2,6^3,6^4,6^5,6^6-hexahydro-1^1*H*-8-oxa-1(5,3)-indola-6(1,3)-pyridazina-2(1,3)-benzenacycloundecaphane-4-yl)-3-methyl-2-(methylamino) butanamide (5 g, crude) as a solid. The crude product was used in the next step directly without further purification. ^{1}H NMR (400 MHz, DMSO-d_6) δ 8.77 (dd, *J* = 4.7, 1.7 Hz, 1H), 8.51 (s, 1H), 8.02 (s, 1H), 7.91 (s, 1H), 7.84 (dd, *J* = 7.7, 1.8 Hz, 1H), 7.72 (dd, *J* = 8.8, 1.7 Hz, 1H), 7.67 (d, *J* = 8.0 Hz, 1H), 7.62 (d, *J* = 8.7 Hz, 1H), 7.53 (dd, *J* = 7.8, 4.7 Hz, 1H), 7.28 (t, *J* = 7.7 Hz, 1H), 7.13 (d, *J* = 7.6 Hz, 1H), 5.59 – 5.40 (m, 2H), 4.34 – 4.19 (m, 3H), 4.02 – 4.01 (m, 1H), 3.65 (q, *J* = 11.0 Hz, 2H), 3.11 (s, 3H), 2.99 (d, *J* = 13.7 Hz,

1H), 2.85 – 2.75 (m, 2H), 2.72 (d, *J* = 8.7 Hz, 2H), 2.21 (s, 2H), 2.03 (d, *J* = 12.9 Hz, 1H), 1.92 – 1.78 (m, 2H), 1.68 (d, *J* = 12.6 Hz, 1H), 1.56 (d, *J* = 16.2 Hz, 1H), 1.40 (d, *J* = 6.0 Hz, 3H), 1.00 – 0.90 (m, 5H), 0.90 – 0.72 (m, 6H), 0.55 (s, 3H); ^{13}C NMR (101 MHz, DMSO) δ 172.50, 172.03, 170.77, 169.98, 159.40, 150.22, 140.65, 140.31, 136.82, 135.78, 130.34, 130.18, 129.02, 127.27, 127.10, 125.75, 123.93, 123.36, 120.02, 116.07, 111.43, 111.15, 76.08, 70.92, 68.54, 60.20, 56.07, 52.06, 38.42, 35.97, 33.80, 32.76, 30.72, 29.26, 25.92, 25.28, 23.40, 21.13, 19.21, 19.18, 17.08, 15.07, 14.48.; LCMS (ESI): m/z [M+H] calc'd for $C_{43}H_{56}N_6O_5$ 736.3; found 737.2.

tert-Butyl (3S)-3-(((2S)-1-(((6^{3}S,4S)-1^1-ethyl-1^2-(2-((S)-1-methoxyethyl) pyridin-3-yl)-10,10-dimethyl-5,7-dioxo-6^1,6^2,6^3,6^4,6^5,6^6-hexahydro-1^{1}H-8-oxa-1(5,3)-indola-6(1,3)-pyridazina-2(1,3)-benzenacycloundecaphane-4-yl) amino)-3-methyl-1-oxobutan-2-yl) (methyl)carbamoyl) pyrrolidine-1-carboxylate (S3-3) To a stirred solution of (2*S*)-N-((6^3*S*,4*S*)-1^1-ethyl-1^2-(2-((*S*)-1-methoxyethyl) pyridin-3-yl)-10,10-dimethyl-5,7-dioxo-6^1,6^2,6^3,6^4,6^5,6^6-hexahydro-1^1*H*-8-oxa-1(5,3)-indola-6(1,3)-pyridazina-2(1,3)-benzenacycloundecaphane-4-yl)-3-methyl-2-(methylamino) butanamide (3 g, 4.07 mmol) and (S)-1-(*tert*-butoxycarbonyl)pyrrolidine-3-carboxylic acid (1.75 g, 8.14 mmol) in DMF (30 mL) was added HATU (2.32 g, 6.11 mmol) and DIPEA (2.63 g, 20.36 mmol) dropwise at 0 °C. The resulting mixture was stirred for 1 h then quenched by the addition of H_2O (50 mL). The resulting mixture was extracted with EtOAc (3 x 30 mL). The combined organic layers were washed with brine (3 x 50 mL) and dried over anhydrous Na_2SO_4. After filtration, the filtrate was concentrated under reduced pressure. The residue was purified by silica gel column chromatography, eluted with DCM / MeOH (10:1) to afford *tert*-butyl (3*S*)-3-(((2*S*)-1-(((6^3*S*,4*S*)-1^1-ethyl-1^2-(2-((*S*)-1-methoxyethyl) pyridin-3-yl)-10,10-dimethyl-5,7-dioxo-6^1,6^2,6^3,6^4,6^5,6^6-hexahydro-1^1*H*-8-oxa-1(5,3)-indola-6(1,3)-pyridazina-2(1,3)-benzenacycloundecaphane-4-yl) amino)-3-methyl-1-oxobutan-2-yl) (methyl)carbamoyl) pyrrolidine-1-carboxylate (3 g, 71.00%) as a solid. ^{1}H NMR (400 MHz, DMSO-d_6) δ 8.77 (dd, *J* = 4.8, 1.7 Hz, 1H), 8.77 – 8.21 (m, 1H), 8.00 (s, 1H), 7.91 – 7.80 (m, 2H), 7.77 – 7.59 (m, 3H), 7.54 – 7.53 (m, 1H), 7.50 – 7.25 (m, 1H), 7.14 – 6.99 (m, 1H), 5.34 – 5.32 (m, 2H), 4.71 – 4.68 (m, 1H), 4.26 (dd, *J* = 6.2, 3.3 Hz, 3H), 3.99 (d, *J* = 13.7 Hz, 1H), 3.72 – 3.51 (m, 3H), 3.51 – 3.37 (m, 2H), 3.28 (s, 1H), 3.12 (d, *J* = 5.3 Hz, 3H), 3.06 – 2.89 (m, 1H), 2.76 (t, *J* = 17.9 Hz, 7H), 2.04 – 1.94 (m, 3H), 1.93 (d, *J* = 11.1 Hz, 1H), 1.83 (d, *J* = 12.1 Hz, 1H), 1.69 (s, 1H), 1.57 (s, 1H), 1.50 – 1.26 (m, 13H), 1.25 (d, *J* = 9.1 Hz, 1H), 1.02 – 0.92 (m, 4H), 0.91 – 0.66 (m, 8H), 0.54 (s, 3H); ^{13}C NMR (101 MHz, DMSO) δ 171.11, 170.64, 170.52, 169.99, 168.24, 167.80, 157.40, 151.89, 151.77, 148.18, 138.99, 138.60, 138.41, 138.21, 134.85, 133.82, 133.77, 128.19, 126.91, 125.24, 123.58, 122.00, 121.34, 118.00, 116.35, 113.94, 109.42, 109.16, 76.85, 76.75, 76.62, 74.03, 68.87, 62.79, 59.56, 54.07, 47.60, 46.73, 43.78, 36.37, 33.93, 30.71, 28.76, 27.84, 27.51, 27.24, 26.62, 26.56, 25.96, 24.62, 23.98, 23.89, 23.27, 23.14, 21.41, 18.07, 17.52, 17.42, 16.91, 14.96, 13.03.; LCMS (ESI): m/z [M+H] calc'd for $C_{53}H_{71}N_7O_8$ 933.5; found 934.5.

(3S)-N-((2S)-1-(((6^{3}S,4S)-1^1-Ethyl-1^2-(2-((S)-1-methoxyethyl) pyridin-3-yl)-10,10-dimethyl-5,7-dioxo-6^1,6^2,6^3,6^4,6^5,6^6-hexahydro-1^{1}H-8-oxa-1(5,3)-indola-6(1,3)-pyridazina-2(1,3)-benzenacycloundecaphane-4-yl) amino)-3-methyl-1-oxobutan-2-yl)-N-methylpyrrolidine-3-carboxamide (S3-4) To a solution of *tert*-butyl (3*S*)-3-(((2*S*)-1-(((6^3*S*,4*S*)-1^1-ethyl-1^2-(2-((*S*)-1-methoxyethyl) pyridin-3-yl)-10,10-dimethyl-5,7-dioxo-6^1,6^2,6^3,6^4,6^5,6^6-hexahydro-1^1*H*-8-oxa-1(5,3)-indola-6(1,3)-pyridazina-2(1,3)-benzenacycloundecaphane-4-yl) amino)-3-methyl-1-oxobutan-2-yl) (methyl)carbamoyl) pyrrolidine-1-carboxylate (3 g, 3.21 mmol) in DCM (24 mL) was added TFA (8 mL, 107.71 mmol) and the reaction stirred for 1 h at 0 °C. The resulting mixture was concentrated under reduced pressure. The residue was dissolved in EtOAc (20 mL) and neutralized to pH 9 with saturated $NaHCO_3$ (100 mL). The resulting mixture was extracted with EtOAc (3 x 30 mL). The combined organic layers were washed with brine (1 x 100 mL) and dried over anhydrous Na_2SO_4. After filtration, the filtrate was concentrated under reduced pressure. This resulted in (3*S*)-*N*-((2*S*)-1-(((6^3*S*,4*S*)-1^1-ethyl-1^2-(2-((*S*)-1-methoxyethyl) pyridin-3-yl)-10,10-dimethyl-5,7-dioxo-6^1,6^2,6^3,6^4,6^5,6^6-hexahydro-1^1*H*-8-oxa-1(5,3)-indola-6(1,3)-pyridazina-2(1,3)-benzenacycloundecaphane-4-yl) amino)-3-methyl-1-oxobutan-2-yl)-N-methylpyrrolidine-3-carboxamide (3 g, crude) as a solid. The crude product was used in the next step directly without further purification. ^{1}H NMR (400 MHz, DMSO-d_6) δ 8.77 (dd, *J* = 4.7, 1.8 Hz, 1H), 8.77 – 8.21 (m, 1H), 8.00 (s, 1H), 7.94 – 7.81 (m, 2H), 7.75 – 7.59 (m, 3H), 7.58 – 7.47 (m, 1H), 7.28 (dt, *J* = 20.3, 7.7 Hz, 1H), 7.08 (dd, *J* = 15.9, 7.5 Hz, 1H), 5.37 (d, *J* = 9.0 Hz, 2H), 4.69 – 4.43 (m, 1H), 4.37 – 4.14 (m, 3H), 3.96 – 3.95(m, 1H),3.70 – 3.61 (m, 2H), 3.54 – 3.42 (m, 2H), 3.25 – 3.13 (m, 3H), 3.10 (d, *J* = 8.0 Hz, 3H), 3.00 (d, *J* = 13.6 Hz, 1H), 2.90 – 2.67 (m, 7H), 2.25 – 2.15 (m, 3H), 1.83 – 1.75 (m, 2H), 1.74 – 1.48 (m, 3H), 1.40 (d, *J* = 6.1 Hz, 3H), 1.25 (d, *J* = 9.2 Hz, 1H), 0.98 (d, *J* = 6.7 Hz, 4H), 0.92 (d, *J* = 6.4 Hz, 2H), 0.81 – 0.68 (m, 6H), 0.57 (d, *J* = 11.3 Hz, 3H); ^{13}C NMR (101 MHz, DMSO) δ 172.67, 172.62, 172.38, 172.01, 170.12, 169.73, 159.98, 159.66, 159.41, 159.35, 159.03, 150.25, 140.95, 140.76, 140.67, 140.31, 140.21, 136.83, 136.77, 135.81, 135.78, 130.27, 130.16, 129.09, 128.97, 127.23, 127.09, 125.61, 123.83, 123.35, 122.05, 119.99, 119.09, 116.13, 115.93, 113.17, 111.43, 111.18, 76.03, 70.95, 64.88, 61.74, 56.09, 48.05, 47.63, 45.47, 45.23, 39.30, 38.42, 38.19, 35.95, 32.74, 30.98, 29.85, 29.60, 29.25, 28.94, 28.12, 26.86, 25.91, 25.82, 25.39, 25.25, 23.41, 20.13, 19.43, 19.38, 18.98, 17.24, 17.10, 15.07, 1.51.; LCMS (ESI): m/z [M+H] calc'd for $C_{48}H_{63}N_7O_6$ 833.5; found 834.5.

(3S)-1-Acetyl-N-((2S)-1-(((6^{3}S,4S)-1^1-ethyl-1^2-(2-((S)-1-methoxyethyl)pyridin-3-yl)-10,10-dimethyl-5,7-dioxo-6^1,6^2,6^3,6^4,6^5,6^6-hexahydro-1^{1}H-8-oxa-1(5,3)-indola-6(1,3)-pyridazina-2(1,3)-benzenacycloundecaphane-4-yl)amino)-3-methyl-1-oxobutan-2-yl)-N-methylpyrrolidine-3-carboxamide (Compound 1) To a stirred solution of (3*S*)-N-((2*S*)-1-(((6^3*S*,4*S*)-1^1-ethyl-12-(2-((S)-1-methoxyethyl)pyridin-3-yl)-10,10-dimethyl-5,7-dioxo-6^1,6^2,6^3,6^4,6^5,6^6-hexahydro-1^1*H*-8-oxa-1(5,3)-indola-6(1,3)-pyridazina-2(1,3)-benzenacycloundecaphane-4-yl)amino)-3-methyl-1-oxobutan-2-yl)-*N*-

methylpyrrolidine-3-carboxamide (290 mg, 0.35 mmol) and TEA (105.55 mg, 1.04 mmol) in DCM (5 mL) was added acetyl chloride (30.02 mg, 0.38 mmol) dropwise at 0 °C under a nitrogen atmosphere. The resulting mixture was stirred for 2 h at 0 °C then quenched with H_2O (10 mL). The resulting mixture was extracted with EtOAc (2 x 10 mL). The combined organic layers were washed with brine (2 x 10 mL) and dried over anhydrous Na_2SO_4. After filtration, the filtrate was concentrated under reduced pressure. The residue was purified by Prep-TLC (DCM / MeOH 15:1) to afford the crude product (80 mg) as a solid. This was further purified by reverse phase chromatography with the following conditions (Column: XBridge Prep OBD C18 Column, 30*150 mm, 5µm; Mobile Phase A: Water (10 mmol/L NH_4HCO_3), Mobile Phase B: ACN; Flow rate: 60 mL/min; Gradient: 36% B to 58% B in 8 min, 58% B; Wave Length: 254/220 nm; RT1(min): 7.95) to afford(3*S*)-1-acetyl-N-((2*S*)-1-(((6^3*S*,4*S*)-1^1-ethyl-1^2-(2-((*S*)-1-methoxyethyl)pyridin-3-yl)-10,10-dimethyl-5,7-dioxo-6^1,6^2,6^3,6^4,6^5,6^6-hexahydro-1^1*H*-8-oxa-1(5,3)-indola-6(1,3)-pyridazina-2(1,3)-benzenacycloundecaphane-4-yl)amino)-3-methyl-1-oxobutan-2-yl)-*N*-methylpyrrolidine-3-carboxamide (28.5 mg, 10% yield) as a solid. ^{1}H NMR (400 MHz, DMSO-d_6) δ 8.81 – 8.74 (m, 1H), 8.72 – 8.18 (m, 1H), 8.00 (s, 1H), 7.92 – 7.81 (m, 2H), 7.77 – 7.58 (m, 3H), 7.54 (dd, *J* = 7.7, 4.8 Hz, 1H), 7.36 – 7.18 (m, 1H), 7.09 (q, *J* = 7.5 Hz, 1H), 5.47 – 5.25 (m, 2H), 4.36 – 4.19 (m, 3H), 4.03 – 4.01 (m, 3H), 3.73 – 3.49 (m, 7H), 3.13 – 3.11 (m, 3H), 2.97 – 2.66 (m, 8H), 2.22 – 1.92 (m, 7H), 1.87 – 1.53 (m, 4H), 1.40 (d, *J* = 6.1 Hz, 3H), 1.02 – 0.87 (m, 6H), 0.86 – 0.70 (m, 6H), 0.54 (s, 3H); ^{13}C NMR (101 MHz, DMSO) δ 172.76, 172.52, 172.03, 170.37, 169.82, 159.29, 150.11, 140.88, 140.25, 136.74, 135.83, 135.78, 130.15, 128.99, 127.31, 127.02, 125.64, 123.90, 123.51, 120.07, 115.91, 111.49, 75.99, 70.89, 70.27, 64.73, 61.57, 56.16, 49.66, 49.18, 48.39, 47.03, 46.84, 45.44, 45.22, 35.95, 32.72, 30.86, 30.19, 29.30, 26.00, 25.92, 25.20, 22.77, 22.62, 20.10, 19.55, 19.01, 17.10, 15.10.; HRMS(ESI$^+$, m/z) calc'd for $(C_{50}H_{66}N_7O_7)^+$: 876.50182 [M+H]$^+$, found 876.49890.

Synthesis of Intermediate 2

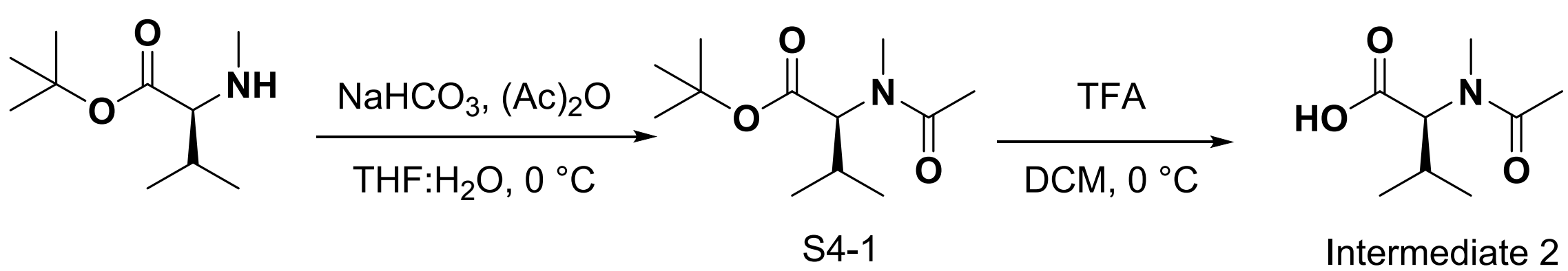

tert-Butyl N-acetyl-N-methyl-L-valinate (S4-1) Into a 250 mL round-bottom flask were added *tert*-butyl (2*S*)-3-methyl-2-(methylamino)butanoate (1500 mg, 8.009 mmol), THF (10 mL), H_2O (10 mL), $NaHCO_3$ (2691.31 mg, 32.04 mmol), and $(Ac)_2O$ (1226.49 mg, 12.01 mmol) at 0 °C. The resulting mixture was stirred at 0 °C for 3 h under an air atmosphere. The resulting mixture was concentrated under reduced

pressure. The aqueous layer was extracted with EtOAc (4 x 150 mL). The combined organic layers were washed with brine (1 x 100 mL), dried over anhydrous Na_2SO_4. After filtration, the filtrate was concentrated under reduced pressure. The residue was purified by reverse phase chromatography (10→50% MeCN/H_2O) to afford the desired product (1600 mg) as an oil. ^{1}H NMR (400 MHz, DMSO-d_6) δ 4.57 (d, J = 10.4 Hz, 1H), 2.90 (s, 3H), 2.15 – 2.03 (m, 1H), 2.03 (s, 3H), 1.41 (s, 9H), 0.94 (d, J = 6.5 Hz, 3H), 0.76 (d, J = 6.7 Hz, 3H). ^{113}C NMR (101 MHz, DMSO-d_6) δ 170.92, 170.19, 81.02, 62.04, 32.11, 28.02, 27.42, 22.13, 20.01, 19.28. HRMS (ESI$^+$, m/z) calc'd for $(C_{12}H_{23}NO_3)^+$: 229.16779 [M+H]$^+$, found 230.17439.

N-Acetyl-N-methyl-L-valine (Intermediate 2) Into a 40 mL vial were added *tert*-butyl *N*-acetyl-*N*-methyl-*L*-valinate (500 mg, 2.180 mmol), DCM (5 mL) and TFA (2 mL) at 0 °C. The resulting mixture was stirred at 0 °C →15 °C for 8 h under an air atmosphere. The resulting mixture was concentrated under reduced pressure to afford the desired product (160 mg, 42% yield) as an oil. ^{1}H NMR (400 MHz, DMSO-d_6) δ 4.61 (d, J = 10.4 Hz, 1H), 2.89 (s, 3H), 2.17 – 2.04 (m, 1H), 2.03 (s, 3H), 0.95 (d, J = 6.5 Hz, 3H), 0.77 (d, J = 6.7 Hz, 3H). ^{13}C NMR (101 MHz, DMSO-d_6) δ 172.56, 170.98, 61.37, 32.36, 28.40, 27.26, 22.15, 20.26, 19.31. HRMS (ESI$^+$, m/z) calculated for $(C_8H_{15}NO_3)^+$: 173.10519 [M+H]$^+$, found 174.11220.

Synthesis of Compound 2

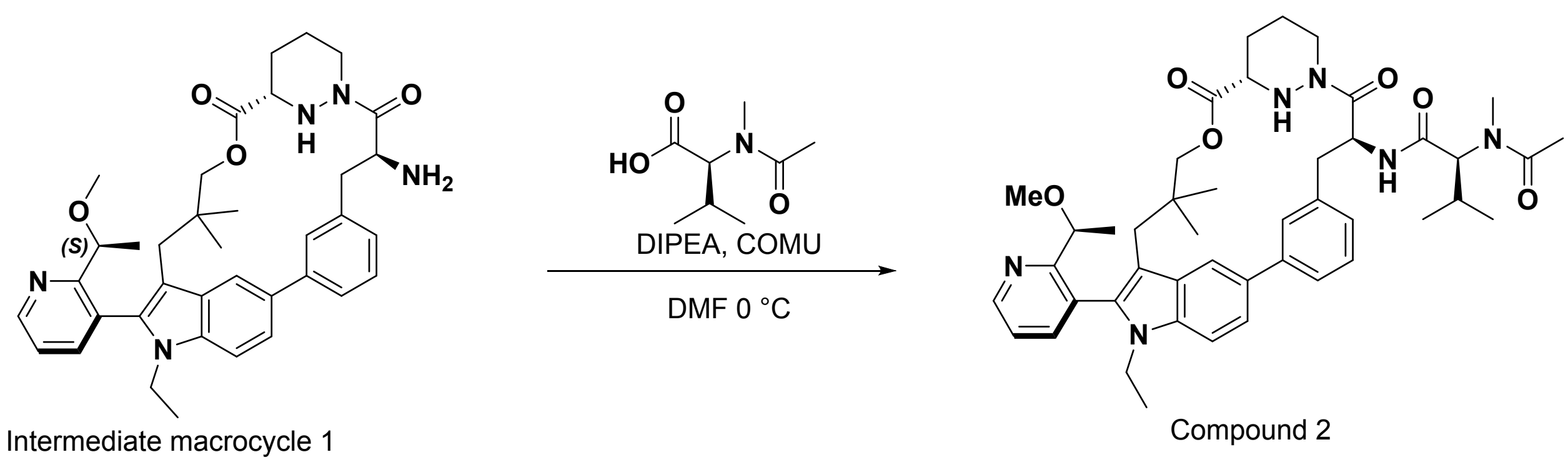

(2S)-N-((6^{3}S,4S)-1^1-ethyl-1^2-(2-((S)-1-methoxyethyl)pyridin-3-yl)-10,10-dimethyl-5,7-dioxo-6^1,6^2,6^3,6^4,6^5,6^6-hexahydro-1^{1}H-8-oxa-1(5,3)-indola-6(1,3)-pyridazina-2(1,3)-benzenacycloundecaphane-4-yl)-3-methyl-2-(N-methylacetamido)butanamide (Compound 2) To a stirred solution of (6^3*S*,4*S*)-4-amino-1^1-ethyl-1^2-(2-((*S*)-1-methoxyethyl)pyridin-3-yl)-10,10-dimethyl-6^1,6^2,6^3,6^4,6^5,6^6-hexahydro-1^1*H*-8-oxa-1(5,3)-indola-6(1,3)-pyridazina-2(1,3)-benzenacycloundecaphane-5,7-dione (250 mg, 0.401 mmol) and DIPEA (155.40 mg, 1.20 mmol) in DMF (1 mL) were added (2*S*)-3-methyl-2-(*N*-methylacetamido)butanoic acid (83.30 mg, 0.481 mmol) and COMU (257.46 mg, 0.602 mmol) at 0 °C under an air atmosphere. The resulting mixture was stirred at room temperature for additional 1 h. The

reaction was quenched by the addition of H_2O (50mL) at room temperature. The resulting mixture was extracted with EtOAc (3 x 50 mL). The combined organic layers were washed with brine (1 x 50 mL), dried over anhydrous Na_2SO_4. After filtration, the filtrate was concentrated under reduced pressure. The residue was purified by Prep-TLC (EtOAc) to afford the desired product (150 mg, 48% yield) as a solid. ^1H NMR (400 MHz, DMSO-d_6) δ 8.77 (dd, J = 4.8, 1.7 Hz, 1H), 7.89 – 8.00 (m, J = 2.3 Hz, 2H), 7.89 – 7.79 (m, 2H), 7.72 – 7.56 (m, 3H), 7.53 (dd, J = 7.8, 4.8 Hz, 1H), 7.27 (dt, J = 17.2, 7.7 Hz, 1H), 7.13 – 7.08 (m, 1H), 5.41 – 5.30 (m, 2H), 4.34 – 4.20 (m, 3H), 4.10 – 3.91 (m, 2H), 3.69 – 3.53 (m, 5H), 3.13 – 3.07 (m, 6H), 2.97 – 2.77 (m, 5H), 2.66 (s, 2H), 2.17 – 2.02 (m, 5H), 1.83 – 1.57 (m, 3H), 1.43 – 1.37 (m, 3H), 0.94 (dt, J = 17.0, 7.0 Hz, 6H), 0.87 – 0.71 (m, 6H), 0.53 (d, J = 12.0 Hz, 3H). ^{13}C NMR (101 MHz, DMSO-d_6) δ 172.62, 172.12, 170.12, 164.23, 159.40, 150.24, 140.96, 140.83, 140.70, 140.31, 136.89, 135.80, 130.29, 130.21, 129.07, 127.25, 127.17, 126.84, 125.60, 123.98, 123.83, 123.42, 120.05, 115.92, 111.44, 111.27, 76.03, 70.87, 66.37, 66.23, 60.94, 59.27, 56.14, 52.12, 47.45, 38.42, 35.97, 32.73, 31.59, 29.24, 28.55, 27.72, 26.86, 25.99, 25.95, 25.24, 23.41, 22.53, 22.40, 20.00, 19.62, 19.29, 19.22, 17.12, 17.05, 15.13, 15.11. HRMS (ESI$^+$, m/z) calculated for $(C_{45}H_{58}N_6O_6)^+$: 778.44178 [M+H]$^+$, found 779.44714.

Synthesis of Intermediate Macrocycle 2

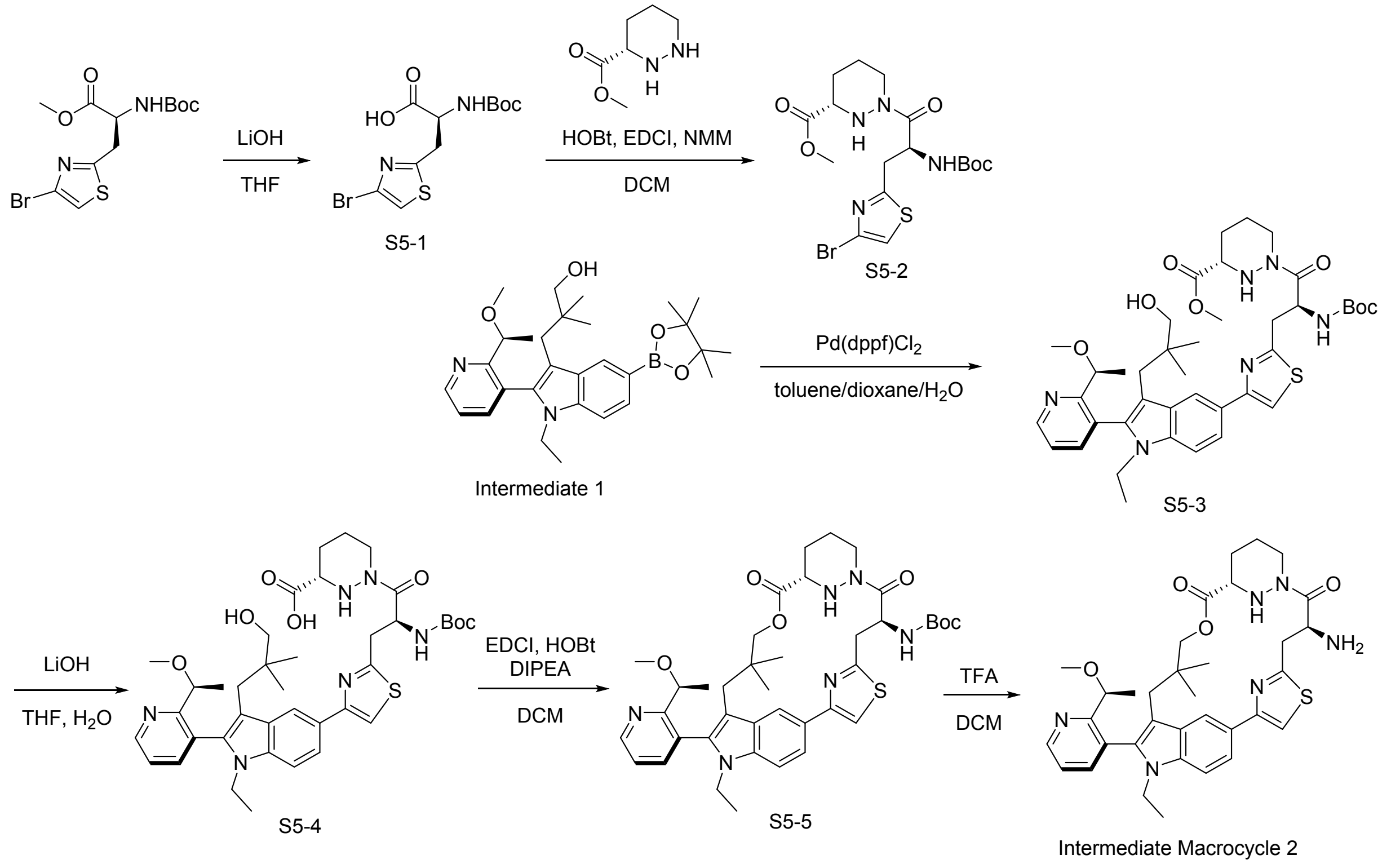

(S)-3-(4-Bromothiazol-2-yl)-2-((tert-butoxycarbonyl)amino)propanoic acid (S5-1) To a solution of methyl (2*S*)-3-(4-bromo-1,3-thiazol-2-yl)-2-[(*tert*-butoxycarbonyl)amino]propanoate (110 g, 301.2 mmol) in THF (500 mL) and H_2O (200 mL) at room temperature was added LiOH (21.64 g, 903.6 mmol). The solution was stirred for 1 h and was then concentrated under reduced pressure. The residue was adjusted to pH 6 with 1 M HCl and then extracted with DCM (3 x 500 mL). The combined organic layers were dried over Na_2SO_4, filtered, and concentrated under reduced pressure to give (*S*)-3-(4-bromothiazol-2-yl)-2-((*tert*-butoxycarbonyl)amino)propanoic acid (108 g, crude) as an oil . ^{1}H NMR (400 MHz, DMSO-d6) δ 7.70 (s, 1H), 7.24 (d, J = 8.6 Hz, 1H), 4.28 (m, 1H), 3.44 (dd, J = 15.0, 4.5 Hz, 1H), 3.27 (dd, J = 14.9, 9.8 Hz, 1H), 1.36 (s, 9H). ^{13}C NMR (101 MHz, DMSO-d6) δ 172.81, 168.56, 155.73, 123.40, 118.64, 78.85, 53.76, 34.86, 28.58. LCMS (ESI): m/z [M+H] calc'd for $C_{11}H_{16}BrN_2O_4S$ 351.0; found 351.0.

(S)-1-((S)-3-(4-Bromothiazol-2-yl)-2-((tert-butoxycarbonyl)amino)propanoyl)hexahydropyridazine-3-carboxylate (S5-2) To a solution of (*S*)-3-(4-bromothiazol-2-yl)-2-((*tert*-butoxycarbonyl)amino)propanoic acid (70 g, 199.3 mmol) in DCM (500 mL) at 0 °C was added methyl (3*S*)-1,2-diazinane-3-carboxylate bis(trifluoroacetic acid) salt (111.28 g, 298.96 mmol), NMM (219.12 mL, 1993.0 mmol), EDCI (76.41 g, 398.6 mmol) and HOBt (5.39 g, 39.89 mmol). The solution was warmed to room temperature and stirred for 1 h. The reaction was then quenched with H_2O (500 mL) and was extracted with EtOAc (3 x 500 mL). The combined organic layers were dried over Na_2SO_4, filtered, and concentrated under reduced pressure. The residue was purified by silica gel column chromatography to give methyl (*S*)-1-((*S*)-3-(4-bromothiazol-2-yl)-2-((*tert*-butoxycarbonyl)amino)propanoyl)hexahydropyridazine-3-carboxylate (88.1 g, 93% yield) as an oil. ^{1}H NMR (400 MHz, DMSO-d6) δ 7.69 (s, 1H), 6.66 (d, J = 8.9 Hz, 1H), 5.34 (d, J = 9.5 Hz, 1H), 5.24 (m, 1H), 3.85 – 3.71 (m, 1H), 3.67 (s, 3H), 3.60 (q, J = 8.7 Hz, 1H), 3.27 (d, J = 4.0 Hz, 1H), 3.13 (dd, J = 14.7, 8.8 Hz, 2H), 1.96 – 1.78 (m, 1H), 1.68 (m, 2H), 1.57 (m, 1H), 1.35 (s, 9H). ^{13}C NMR (101 MHz, DMSO-d6) δ 171.90, 171.19, 168.68, 155.22, 123.23, 118.79, 78.57, 58.14, 52.20, 50.94, 41.23, 35.91, 28.61, 22.52. LCMS (ESI): m/z [M+H] calc'd for $C_{17}H_{26}BrN_4O_5S$ 477.1; found 477.1.

(3S)-1-[(2S)-2-[(tert-Butoxycarbonyl)amino]-3-{4-[(2M)-1-ethyl-3-(3-hydroxy-2,2-dimethylpropyl)-2-{2-[(1S)-1-methoxyethyl]pyridin-3-yl}indol-5-yl]-1,3-thiazol-2-yl}propanoyl]-1,2-diazinane-3-carboxylate (S5-3) A mixture of methyl (3*S*)-1-[(2*S*)-3-(4-bromo-1,3-thiazol-2-yl)-2-[(*tert*-butoxycarbonyl)amino]propanoyl]-1,2-diazinane-3-carboxylate (2.5 g, 5.24 mmol), 3-[(2M)-1-ethyl-2-{2-[(1*S*)-1-methoxyethyl]pyridin-3-yl}-5-(4,4,5,5-tetramethyl-1,3,2-dioxaborolan-2-yl)indol-3-yl]-2,2-dimethylpropan-1-ol (2.84 g, 5.76 mmol), Pd(dppf)Cl_2 (0.77 g, 1.05 mmol) and K_3PO_4 (2.78 g, 13.09 mmol) in toluene (24 mL), H_2O (8 mL) and dioxane (8 mL) was stirred for 2 h at 70 °C under an argon

atmosphere. The resulting mixture was diluted with H_2O (32 mL) then extracted with EtOAc (2 x 20 mL). The combined organic layers were washed with brine (1 x 20 mL), dried over anhydrous Na_2SO_4. After filtration, the filtrate was concentrated under reduced pressure. The residue was purified by reverse phase flash with the following conditions (Column:C18 Column; Mobile Phase A: Water (0.05% $NH_3 \cdot H_2O$), Mobile Phase B: ACN; Flow rate: 70 mL/min; Gradient: 0% B to 100% B in 30 min, 49% B; Wave Length: 254/220 nm; RT1(min): 5; Number of Runs: 0) to afford methyl (3*S*)-1-[(2*S*)-2-[(*tert*-butoxycarbonyl)amino]-3-{4-[(2M)-1-ethyl-3-(3-hydroxy-2,2-dimethylpropyl)-2-{2-[(1*S*)-1-methoxyethyl]pyridin-3-yl}indol-5-yl]-1,3-thiazol-2-yl}propanoyl]-1,2-diazinane-3-carboxylate (2 g, 50% yield) as a solid. ^{1}H NMR (400 MHz, DMSO-d_6) δ 8.74 (dd, *J* = 4.8, 1.7 Hz, 1H), 8.21 (d, *J* = 1.6 Hz, 1H), 7.86 (dd, *J* = 7.7, 1.8 Hz, 1H), 7.82 – 7.74 (m, 2H), 7.60 – 7.44 (m, 2H), 6.60 (d, *J* = 8.8 Hz, 1H), 5.40 – 5.21 (m, 2H), 4.42 (t, *J* = 5.2 Hz, 1H), 4.11 (dq, *J* = 26.5, 7.2, 6.7 Hz, 2H), 3.90 – 3.76 (m, 2H), 3.63 (s, 4H), 3.36 (dd, *J* = 14.8, 4.3 Hz, 1H), 3.26 (d, *J* = 15.1 Hz, 1H), 3.08 (dd, *J* = 10.2, 5.3 Hz, 1H), 2.99 (dd, *J* = 10.2, 5.3 Hz, 1H), 2.88 (s, 3H), 2.70 (d, *J* = 14.1 Hz, 1H), 2.22 (d, *J* = 14.0 Hz, 1H), 2.07 (s, 3H), 1.96 – 1.82 (m, 1H), 1.69 (s, 2H), 1.63 – 1.49 (m, 1H), 1.39 (d, *J* = 13.7 Hz, 12H), 1.13 (t, *J* = 7.1 Hz, 3H). ^{13}C NMR (101 MHz, $CDCl_3$) δ 169.81, 169.24, 168.95, 162.72, 158.89, 154.71, 153.31, 148.60, 138.36, 133.86, 133.20, 127.55, 125.13, 124.64, 120.14, 118.90, 117.09, 116.88, 114.56, 110.97, 109.16, 108.79, 107.67, 77.67, 75.60, 75.28, 74.96, 73.94, 69.11, 56.59, 54.58, 50.36, 48.91, 40.32, 36.79, 35.94, 34.88, 34.58, 31.40, 27.82, 26.64, 26.55, 24.59, 23.44, 23.11, 22.25, 20.77, 16.42, 13.32, 0.00, -1.85. LCMS (ESI): m/z [M+H] calc'd for $C_{40}H_{54}N_6O_7$ 762; found 763.

(3S)-1-[(2S)-2-[(tert-Butoxycarbonyl)amino]-3-{4-[(2M)-1-ethyl-3-(3-hydroxy-2,2-dimethylpropyl)-2-{2-[(1S)-1-methoxyethyl]pyridin-3-yl}indol-5-yl]-1,3-thiazol-2-yl}propanoyl]-1,2-diazinane-3-carboxylic acid (S5-4) To a stirred solution of methyl (3*S*)-1-[(2*S*)-2-[(*tert*-butoxycarbonyl)amino]-3-{4-[(2M)-1-ethyl-3-(3-hydroxy-2,2-dimethylpropyl)-2-{2-[(1*S*)-1-methoxyethyl]pyridin-3-yl}indol-5-yl]-1,3-thiazol-2-yl}propanoyl]-1,2-diazinane-3-carboxylate (1 g, 1.31 mmol) in THF (5 mL) was added $LiOH \cdot H_2O$ (164.99 mg, 3.93 mmol) in H_2O (5 mL) dropwise at 0 °C. The resulting mixture was stirred for 2 h at room temperature then the solution was acidified to pH 5 with 1M HCl (aq.). The resulting mixture was extracted with EtOAc (3 x 10 mL). The combined organic layers were dried over anhydrous Na_2SO_4. After filtration, the filtrate was concentrated under reduced pressure. This resulted in (3*S*)-1-[(2*S*)-2-[(*tert*-butoxycarbonyl)amino]-3-{4-[(2M)-1-ethyl-3-(3-hydroxy-2,2-dimethylpropyl)-2-{2-[(1*S*)-1-methoxyethyl]pyridin-3-yl}indol-5-yl]-1,3-thiazol-2-yl}propanoyl]-1,2-diazinane-3-carboxylic acid (871 mg, crude) as a solid. ^{1}H NMR (400 MHz, DMSO-d_6) δ 8.74 (dd, *J* = 4.8, 1.7 Hz, 1H), 8.24 (dd, *J* = 13.1, 1.7 Hz, 1H), 7.87 (dd, *J* = 7.8, 1.6 Hz, 1H), 7.78 (dd, *J* = 7.3, 3.0 Hz, 2H), 7.58 – 7.48 (m, 2H), 6.57 (d, *J* = 8.6 Hz, 1H), 5.32 – 5.22 (m, 1H), 5.13 (d, *J* = 11.0 Hz, 1H), 4.39 (dt, *J* = 8.8, 4.5 Hz, 1H), 4.14 (dd, *J* =

6.3, 3.5 Hz, 2H), 3.83 (ddd, *J* = 14.7, 7.3, 2.8 Hz, 1H), 3.62 – 3.48 (m, 2H), 3.16 – 2.96 (m, 3H), 2.87 (d, *J* = 4.9 Hz, 4H), 2.70 (dd, *J* = 14.1, 6.4 Hz, 1H), 2.22 (dd, *J* = 14.1, 5.4 Hz, 1H), 1.95 (d, *J* = 12.3 Hz, 1H), 1.79 – 1.69 (m, 1H), 1.62 – 1.53 (m, 1H), 1.39 (dd, *J* = 12.4, 5.2 Hz, 13H), 1.32 (s, 1H), 1.16 – 1.08 (m, 3H), 0.67 (d, *J* = 2.2 Hz, 3H), 0.59 (s, 3H). ^{13}C NMR (101 MHz, $CDCl_3$) δ 173.65, 173.21, 171.29, 171.24, 165.80, 165.12, 160.68, 160.61, 156.43, 156.12, 155.44, 150.30, 150.23, 140.60, 135.77, 135.72, 134.97, 134.84, 129.40, 129.37, 127.16, 127.06, 126.34, 125.75, 122.13, 120.75, 120.33, 119.18, 119.02, 113.11, 112.97, 111.33, 110.72, 109.73, 109.60, 80.01, 79.82, 77.44, 77.12, 76.80, 75.75, 71.22, 71.00, 67.93, 60.43, 58.54, 56.44, 52.70, 50.85, 42.14, 38.71, 38.64, 37.79, 37.61, 36.32, 35.04, 33.26, 32.94, 31.92, 29.68, 28.39, 28.36, 25.58, 25.55, 25.24, 24.82, 24.71, 22.89, 22.68, 21.04, 18.52, 18.38, 15.21, 15.18, 14.19, 0.01. LCMS (ESI): m/z [M+H] calc'd for $C_{39}H_{52}N_6O_7S$ 748; found 749.

((6^{3}S,4S,Z)-1^1-Ethyl-1^2-(2-((S)-1-methoxyethyl)pyridin-3-yl)-10,10-dimethyl-5,7-dioxo-6^1,6^2,6^3,6^4,6^5,6^6-hexahydro-1^{1}H-8-oxa-2(4,2)-thiazola-1(5,3)-indola-6(1,3)-pyridazinacycloundecaphane-4-yl)carbamate (S5-5) To a stirred solution of (3*S*)-1-[(2*S*)-2-[(*tert*-butoxycarbonyl)amino]-3-{4-[(2M)-1-ethyl-3-(3-hydroxy-2,2-dimethylpropyl)-2-{2-[(1*S*)-1-methoxyethyl]pyridin-3-yl}indol-5-yl]-1,3-thiazol-2-yl}propanoyl]-1,2-diazinane-3-carboxylic acid (500 mg, 0.668 mmol) and DIPEA (3.49 mL, 20.04 mmol) in DCM (5 mL) was added EDCI (3.58 g, 18.70 mmol) and HOBT (451.06 mg, 3.34 mmol) portion wise at 0 °C under a nitrogen atmosphere. The resulting mixture was stirred for 16 h at room temperature then quenched by the addition of H_2O (10 mL). The resulting mixture was extracted with DCM (3 x 100 mL). The combined organic layers were washed with brine (1 x 10 mL), dried over anhydrous Na_2SO_4. After filtration, the filtrate was concentrated under reduced pressure. The residue was purified by silica gel column chromatography, eluted with Pet. ether / EtOAc (1:1) to afford *tert*-butyl ((6^3*S*,4*S*,*Z*)-1^1-ethyl-1^2-(2-((*S*)-1-methoxyethyl)pyridin-3-yl)-10,10-dimethyl-5,7-dioxo-6^1,6^2,6^3,6^4,6^5,6^6-hexahydro-1^1*H*-8-oxa-2(4,2)-thiazola-1(5,3)-indola-6(1,3)-pyridazinacycloundecaphane-4-yl)carbamate (300 mg, 61% yield) as a solid. ^{1}H NMR (400 MHz, DMSO-d_6) δ 8.75 (dd, *J* = 4.7, 1.8 Hz, 1H), 8.50 (d, *J* = 1.7 Hz, 1H), 7.83 (s, 1H), 7.77 (ddd, *J* = 15.2, 8.2, 1.7 Hz, 2H), 7.58 (d, *J* = 8.6 Hz, 1H), 7.52 (dd, *J* = 7.7, 4.7 Hz, 1H), 7.32 (d, *J* = 9.2 Hz, 1H), 5.23 (t, *J* = 8.9 Hz, 1H), 5.08 (d, *J* = 12.2 Hz, 1H), 4.38 – 4.26 (m, 2H), 4.26 – 4.08 (m, 3H), 3.63 – 3.50 (m, 2H), 3.27 (s, 3H), 3.17 (q, *J* = 7.2 Hz, 4H), 3.00 – 2.91 (m, 1H), 2.81 – 2.70 (m, 1H), 2.39 (d, *J* = 14.4 Hz, 2H), 2.25 (d, *J* = 7.0 Hz, 1H), 2.11 (s, 3H), 1.79 (s, 2H), 1.61 (p, *J* = 6.8 Hz, 1H), 1.40 (s, 7H), 1.37 (d, *J* = 6.1 Hz, 4H), 1.25 (d, *J* = 9.3 Hz, 2H), 1.15 (t, *J* = 7.2 Hz, 2H), 0.93 – 0.80 (m, 6H). ^{13}C NMR (101 MHz, $CDCl_3$) δ 173.51, 171.97, 165.63, 159.79, 159.14, 155.59, 154.93, 150.49, 150.11, 140.54, 140.12, 136.25, 135.49, 130.28, 127.09, 126.47, 122.78, 121.92, 121.77, 120.78, 119.47, 118.07, 112.28, 110.42, 109.92, 109.50, 79.82, 77.47, 77.15, 76.83, 76.03, 75.56, 70.82, 69.67, 58.57, 57.48, 56.91, 56.52, 56.39, 50.67, 45.50, 45.10, 44.85, 41.71, 41.47, 40.37, 39.22, 38.78, 38.16, 37.28,

35.75, 35.27, 32.63, 32.18, 31.91, 31.51, 31.44, 30.19, 30.13, 29.68, 29.65, 29.34, 29.09, 28.38, 28.35, 27.36, 25.99, 24.95, 23.47, 23.27, 22.68, 18.45, 16.74, 16.46, 15.50, 15.25, 14.76, 14.14, 0.02. LCMS (ESI): m/z [M+H] calc'd for $C_{39}H_{50}N_6O_6S$ 730; found 731.

(6^{3}S,4S,Z)-4-Amino-1^1-ethyl-1^2-(2-((S)-1-methoxyethyl)pyridin-3-yl)-10,10-dimethyl-6^1,6^2,6^3,6^4,6^5,6^6-hexahydro-1^{1}H-8-oxa-2(4,2)-thiazola-1(5,3)-indola-6(1,3)-pyridazinacycloundecaphane-5,7-dione (Intermediate macrocycle 3) To a stirred solution of *tert*-butyl ((6^3*S*,4*S*,*Z*)-1^1-ethyl-1^2-(2-((*S*)-1-methoxyethyl)pyridin-3-yl)-10,10-dimethyl-5,7-dioxo-6^1,6^2,6^3,6^4,6^5,6^6-hexahydro-1^1*H*-8-oxa-2(4,2)-thiazola-1(5,3)-indola-6(1,3)-pyridazinacycloundecaphane-4-yl)carbamate (200 mg, 0.274 mmol) in DCM (3 mL) was added TFA (1 mL, 13.46 mmol) dropwise at 0 °C. The resulting mixture was stirred for 1 h at 25 °C then concentrated under reduced pressure. The residue was dissolved in DCM (5 mL) and the resulting solution was basified to pH 8 with saturated $NaHCO_3$ (aq.). The resulting mixture was extracted with DCM (3 x 5 mL). The combined organic layers were washed with brine (1 x 10 mL), dried over anhydrous Na_2SO_4. After filtration, the filtrate was concentrated under reduced pressure. This resulted in (6^3*S*,4*S*,*Z*)-4-amino-1^1-ethyl-1^2-(2-((*S*)-1-methoxyethyl)pyridin-3-yl)-10,10-dimethyl-6^1,6^2,6^3,6^4,6^5,6^6-hexahydro-1^1*H*-8-oxa-2(4,2)-thiazola-1(5,3)-indola-6(1,3)-pyridazinacycloundecaphane-5,7-dione (160 mg, crude) as a solid. ^{1}H NMR (400 MHz, DMSO-d_6) δ 8.75 (dd, *J* = 4.7, 1.8 Hz, 1H), 8.49 (d, *J* = 1.6 Hz, 1H), 7.80 (d, *J* = 8.7 Hz, 2H), 7.74 (dd, *J* = 8.6, 1.6 Hz, 1H), 7.57 (d, *J* = 8.6 Hz, 1H), 7.53 – 7.48 (m, 1H), 4.93 (d, *J* = 12.1 Hz, 1H), 4.39 – 4.24 (m, 4H), 4.17 – 4.05 (m, 2H), 3.58 (q, *J* = 10.9 Hz, 2H), 3.25 (s, 3H), 3.21 (d, *J* = 14.2 Hz, 1H), 2.96 (d, *J* = 14.4 Hz, 1H), 2.73 (ddd, *J* = 14.0, 8.6, 4.5 Hz, 2H), 2.40 (d, *J* = 14.4 Hz, 1H), 2.08 (q, *J* = 7.7, 5.2 Hz, 1H), 1.98 (d, *J* = 7.9 Hz, 1H), 1.84 – 1.61 (m, 2H), 1.50 (dd, *J* = 12.4, 4.3 Hz, 1H), 1.37 (d, *J* = 6.0 Hz, 3H), 0.87 (d, *J* = 5.8 Hz, 6H), 0.32 (s, 3H). ^{13}C NMR (101 MHz, $CDCl_3$) δ 174.93, 170.56, 165.48, 157.93, 153.13, 148.27, 138.29, 134.38, 133.66, 128.41, 125.25, 124.67, 120.92, 117.68, 116.22, 110.46, 108.20, 108.10, 75.61, 75.29, 74.98, 74.18, 69.25, 55.73, 54.50, 49.49, 40.42, 40.10, 36.37, 33.87, 30.76, 27.83, 27.31, 24.07, 23.18, 21.50, 14.69, 12.91, 0.00, -0.83. LCMS (ESI): m/z [M+H] calc'd for $C_{34}H_{42}N_6O_4S$ 630; found 631.

Synthesis of Compound 3

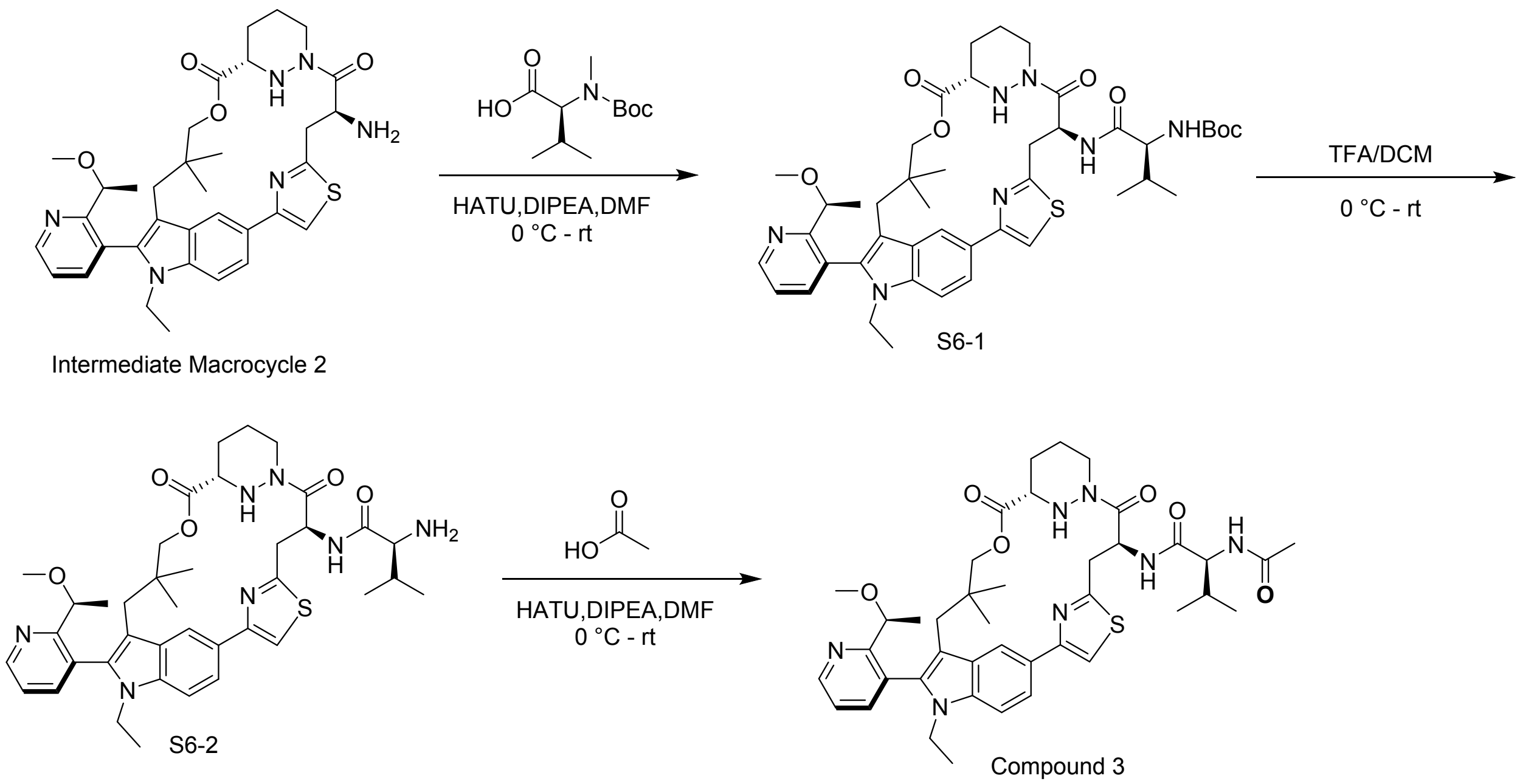

tert-butyl ((2S)-1-(((6^{3}S,4S,Z)-1^1-Ethyl-1^2-(2-((S)-1-methoxyethyl)pyridin-3-yl)-10,10-dimethyl-5,7-dioxo-6^1,6^2,6^3,6^4,6^5,6^6-hexahydro-1^{1}H-8-oxa-2(4,2)-thiazola-1(5,3)-indola-6(1,3)-pyridazinacycloundecaphane-4-yl)amino)-3-methyl-1-oxobutan-2-yl)carbamate (S6-1) To a stirred solution of (6^3*S*,4*S*,*Z*)-4-amino-1^1-ethyl-1^2-(2-((S)-1-methoxyethyl)pyridin-3-yl)-10,10-dimethyl-6^1,6^2,6^3,6^4,6^5,6^6-hexahydro-1^1*H*-8-oxa-2(4,2)-thiazola-1(5,3)-indola-6(1,3)-pyridazinacycloundecaphane-5,7-dione (500 mg, 0.79 mmol) and DIPEA (4.10 g, 31.72 mmol) in DMF (8 mL) were added (2*S*)-2-[(*tert*-butoxycarbonyl)amino]-3-methylbutanoic acid (258.32 mg, 1.19 mmol) and HATU (452.08 mg, 1.19 mmol) in portions at 0 °C under an air atmosphere. The resulting mixture was stirred for 1 h at room temperature. The reaction was quenched by the addition of H_2O (10 mL) at 0 °C. The resulting mixture was extracted with EtOAc (2 x 10 mL). The combined organic layers were washed with brine (3 x 30 mL), dried over anhydrous Na_2SO_4. After filtration, the filtrate was concentrated under reduced pressure. The residue was purified by reverse phase chromatography with the following conditions (Column:C18 Column; Mobile Phase A: Water (0.05% $NH_3 \cdot H_2O$), Mobile Phase B: ACN; Flow rate: 60 mL/min; Gradient: 0% B to 100% B in 30 min, 64% B; Wave Length: 254/220 nm; RT1(min): 5; Number of Runs: 0) to afford *tert*-butyl ((2S)-1-(((6^{3}S,4S,Z)-1^1-ethyl-1^2-(2-((S)-1-methoxyethyl)pyridin-3-yl)-10,10-dimethyl-5,7-dioxo-6^1,6^2,6^3,6^4,6^5,6^6-hexahydro-1^{1}H-8-oxa-2(4,2)-thiazola-1(5,3)-indola-6(1,3)-pyridazinacycloundecaphane-4-yl)amino)-3-methyl-1-oxobutan-2-yl)carbamate (567 mg, 86% yield) as a solid. ^{1}H NMR (400 MHz, Chloroform-*d*) δ 8.88 – 8.79 (m, 1H), 8.58 (d, *J* = 1.6 Hz, 1H), 7.69 (d, *J* = 7.6 Hz, 1H), 7.60 (dd, *J* = 8.5, 1.6 Hz, 1H),

7.44 – 7.38 (m, 1H), 7.35 (d, *J* = 8.6 Hz, 1H), 7.28 (s, 1H), 6.71 (d, *J* = 9.5 Hz, 1H), 5.88 (t, *J* = 8.6 Hz, 1H), 5.12 (d, *J* = 9.1 Hz, 1H), 4.57 (d, *J* = 13.1 Hz, 1H), 4.39 (dd, *J* = 16.5, 10.3 Hz, 2H), 4.18 (ddq, *J* = 28.8, 14.4, 7.3 Hz, 2H), 4.01 (d, *J* = 12.2 Hz, 2H), 3.78 (q, *J* = 11.1 Hz, 2H), 3.59 – 3.45 (m, 1H), 3.41 (s, 3H), 3.25 – 3.06 (m, 2H), 2.67 (t, *J* = 12.3 Hz, 1H), 2.47 (d, *J* = 14.4 Hz, 1H), 2.18 (dd, *J* = 32.4, 10.4 Hz, 2H), 1.86 (dd, *J* = 67.1, 13.2 Hz, 3H), 1.70 – 1.54 (m, 3H), 1.49 (d, *J* = 4.8 Hz, 12H), 1.26 (t, *J* = 7.1 Hz, 1H), 1.03 – 0.86 (m, 12H), 0.43 (s, 3H). ^{13}C NMR (101 MHz, $CDCl_3$) δ 172.73, 171.98, 171.84, 171.43, 171.19, 169.60, 165.50, 163.32, 162.41, 160.80, 159.83, 157.76, 155.89, 155.05, 150.52, 150.13, 140.27, 140.12, 136.17, 135.68, 135.49, 134.71, 130.23, 129.63, 127.10, 127.03, 126.83, 126.48, 122.76, 121.78, 120.86, 119.56, 119.38, 118.09, 112.32, 112.25, 111.54, 110.62, 109.91, 109.54, 79.65, 77.46, 77.14, 76.82, 76.03, 75.57, 73.08, 70.97, 60.41, 60.01, 58.53, 57.54, 56.52, 56.41, 53.49, 49.42, 45.18, 41.78, 39.71, 38.80, 38.19, 37.25, 35.73, 32.65, 32.13, 31.23, 31.04, 29.09, 28.44, 28.36, 26.33, 25.98, 25.29, 25.04, 23.55, 23.27, 21.07, 19.38, 18.50, 18.03, 17.61, 16.54, 15.25, 14.78, 14.21, 0.02. LCMS (ESI): m/z [M+H] calc'd for $C_{44}H_{59}N_7O_7S$ 829.0; found 830.0.

(2S)-2-Amino-N-((6^{3}S,4S,Z)-1^1-ethyl-1^2-(2-((S)-1-methoxyethyl)pyridin-3-yl)-10,10-dimethyl-5,7-dioxo-6^1,6^2,6^3,6^4,6^5,6^6-hexahydro-1^{1}H-8-oxa-2(4,2)-thiazola-1(5,3)-indola-6(1,3)-pyridazinacycloundecaphane-4-yl)-3-methylbutanamide (S6-2) To a stirred solution of *tert*-butyl ((2S)-1-(((6^{3}S,4S,Z)-1^1-ethyl-1^2-(2-((S)-1-methoxyethyl)pyridin-3-yl)-10,10-dimethyl-5,7-dioxo-6^1,6^2,6^3,6^4,6^5,6^6-hexahydro-1^{1}H-8-oxa-2(4,2)-thiazola-1(5,3)-indola-6(1,3)-pyridazinacycloundecaphane-4-yl)amino)-3-methyl-1-oxobutan-2-yl)carbamate (400 mg, 0.48 mmol) in DCM (3 mL) was added TFA (1 mL, 13.46 mmol) dropwise at 0 °C under an air atmosphere. The resulting mixture was stirred for 1 h at room temperature. The resulting mixture was concentrated under reduced pressure. The residue was dissolved in DCM:MeOH (10:1) (5 mL). The residue was neutralized to pH 8 with saturated $NaHCO_3$ (aq.). The resulting mixture was extracted with DCM:MeOH (10:1) (3 x 5 mL). The combined organic layers were washed with brine (1 x 10 mL), dried over anhydrous Na2SO4. After filtration, the filtrate was concentrated under reduced pressure. This resulted in (2*S*)-2-amino-N-((6^3*S*,4*S*,*Z*)-1^1-ethyl-1^2-(2-((*S*)-1-methoxyethyl)pyridin-3-yl)-10,10-dimethyl-5,7-dioxo-6^1,6^2,6^3,6^4,6^5,6^6-hexahydro-1^1*H*-8-oxa-2(4,2)-thiazola-1(5,3)-indola-6(1,3)-pyridazinacycloundecaphane-4-yl)-3-methylbutanamide (335 mg, crude) as a solid. ^{1}H NMR (400 MHz, DMSO-d_6) δ 8.76 (dd, *J* = 4.7, 1.8 Hz, 1H), 8.49 (d, *J* = 1.7 Hz, 1H), 8.32 (d, *J* = 9.3 Hz, 1H), 7.83 (s, 1H), 7.77 (ddd, *J* = 17.7, 8.2, 1.7 Hz, 2H), 7.58 (d, *J* = 8.6 Hz, 1H), 7.52 (dd, *J* = 7.7, 4.7 Hz, 1H), 5.58 (t, *J* = 9.1 Hz, 1H), 5.15 (d, *J* = 12.2 Hz, 1H), 4.39 – 4.19 (m, 4H), 4.16 – 4.06 (m, 1H), 3.58 (s, 2H), 3.27 (s, 3H), 3.20 (dd, *J* = 14.8, 8.9 Hz, 1H), 3.07 – 2.90 (m, 2H), 2.76 (td, *J* = 12.3, 5.4 Hz, 1H), 2.40 (d, *J* = 14.4 Hz, 1H), 2.14 – 2.05 (m, 1H), 1.97 – 1.91 (m, 1H), 1.79 (s, 4H), 1.56 – 1.47 (m, 1H), 1.37 (d, *J* = 6.1 Hz, 3H), 0.99 – 0.76 (m, 12H), 0.32 (s, 3H). ^{13}C NMR (101 MHz, $CDCl_3$) δ 174.79, 174.43, 173.14, 171.95, 171.12, 169.58, 165.57, 163.68, 162.42, 160.80, 159.84, 157.90, 155.09, 150.49, 150.12, 140.26, 140.11,

136.21, 135.69, 135.52, 134.70, 130.24, 129.70, 127.08, 126.86, 126.43, 122.73, 121.74, 120.82, 119.53, 118.13, 112.32, 112.25, 111.24, 110.44, 109.92, 109.48, 77.47, 77.16, 76.84, 76.03, 75.56, 73.07, 70.91, 60.47, 60.37, 58.56, 57.58, 56.51, 56.39, 53.49, 49.08, 48.96, 45.13, 41.71, 39.74, 38.79, 38.18, 37.31, 35.74, 35.51, 32.65, 32.09, 31.11, 31.00, 29.68, 29.12, 27.58, 26.35, 25.99, 25.21, 25.01, 24.46, 23.60, 23.28, 21.04, 19.84, 18.43, 16.53, 16.50, 15.23, 14.76, 14.20, 0.77, 0.01. LCMS (ESI): m/z [M+H] calc'd for $C_{39}H_{51}N_7O_5S$ 729.0; found 730.0.

(2S)-2-Acetamido-N-((63S,4S,Z)-11-ethyl-12-(2-((S)-1-methoxyethyl)pyridin-3-yl)-10,10-dimethyl-5,7-dioxo-61,62,63,64,65,66-hexahydro-11H-8-oxa-2(4,2)-thiazola-1(5,3)-indola-6(1,3)-pyridazinacycloundecaphane-4-yl)-3-methylbutanamide (Compound 3) To a stirred solution of (2*S*)-2-amino-N-((6^3*S*,4*S*,*Z*)-1^1-ethyl-1^2-(2-((*S*)-1-methoxyethyl)pyridin-3-yl)-10,10-dimethyl-5,7-dioxo-6^1,6^2,6^3,6^4,6^5,6^6-hexahydro-1^1*H*-8-oxa-2(4,2)-thiazola-1(5,3)-indola-6(1,3)-pyridazinacycloundecaphane-4-yl)-3-methylbutanamide (200 mg, 0.27 mmol) and DIPEA (106.24 mg, 0.82 mmol) in DMF (2 mL) were added acetic acid (32.91 mg, 0.55 mmol) and HATU (156.27 mg, 0.41 mmol) in portions at 0 °C. The resulting mixture was stirred for 1 h at room temperature then the reaction was quenched by the addition of H_2O (5 mL). The resulting mixture was extracted with DCM (3 x 5 mL). The combined organic layers were washed with brine (3 x 10 mL), dried over anhydrous Na_2SO_4. After filtration, the filtrate was concentrated under reduced pressure. The residue was purified by reverse phase chromatography with the following conditions (Column:C18 Column; Mobile Phase A: Water (0.05% $NH_3 \cdot H_2O$), Mobile Phase B: ACN; Flow rate: 50 mL/min; Gradient: 0% B to 100% B in 25 min, 60% B; Wave Length: 254/220 nm; RT1(min): 5; Number of Runs: 0) to afford (2S)-2-acetamido-N-((63S,4S,Z)-11-ethyl-12-(2-((S)-1-methoxyethyl)pyridin-3-yl)-10,10-dimethyl-5,7-dioxo-61,62,63,64,65,66-hexahydro-11H-8-oxa-2(4,2)-thiazola-1(5,3)-indola-6(1,3)-pyridazinacycloundecaphane-4-yl)-3-methylbutanamide (142 mg, 67% yield) as a solid. ^{1}H NMR (400 MHz, DMSO-d_6) δ 8.75 (dd, *J* = 4.7, 1.7 Hz, 1H), 8.49 (d, *J* = 1.6 Hz, 1H), 8.35 (d, *J* = 8.7 Hz, 1H), 7.85 (d, *J* = 9.9 Hz, 2H), 7.79 (dd, *J* = 7.7, 1.8 Hz, 1H), 7.75 (dd, *J* = 8.6, 1.6 Hz, 1H), 7.58 (d, *J* = 8.7 Hz, 1H), 7.52 (dd, *J* = 7.7, 4.7 Hz, 1H), 5.50 (t, *J* = 8.8 Hz, 1H), 5.13 (d, *J* = 12.1 Hz, 1H), 4.38 – 4.05 (m, 6H), 3.60 – 3.48 (m, 2H), 3.26 (s, 3H), 3.20 (dd, *J* = 14.8, 9.0 Hz, 1H), 2.95 (d, *J* = 14.3 Hz, 1H), 2.76 (td, *J* = 12.6, 4.9 Hz, 1H), 2.40 (d, *J* = 14.3 Hz, 1H), 2.09 (d, *J* = 10.6 Hz, 1H), 1.97 (q, *J* = 6.9 Hz, 1H), 1.90 (s, 3H), 1.79 (s, 2H), 1.50 (dd, *J* = 11.8, 5.6 Hz, 2H), 1.37 (d, *J* = 6.0 Hz, 3H), 1.23 (s, 2H), 0.93 – 0.88 (m, 6H), 0.88 – 0.82 (m, 6H). ^{13}C NMR (101 MHz, $CDCl_3$) δ 172.72, 172.04, 172.01, 171.24, 170.38, 165.65, 159.75, 157.68, 155.00, 150.09, 140.13, 136.18, 135.46, 130.24, 127.10, 126.79, 126.45, 122.78, 120.85, 119.59, 118.04, 112.33, 110.66, 109.90, 77.41, 77.09, 76.78, 76.01, 70.89, 58.25, 57.62, 56.51, 56.36, 49.99, 41.65, 38.47, 38.17, 37.27, 35.70, 32.61, 31.90, 31.45, 29.67, 29.34, 29.06, 25.99, 25.26, 25.02, 23.27, 23.23, 22.68, 19.23, 18.52, 18.39, 16.49, 15.25, 14.74, 14.13, 1.87, -0.00. HRMS (ESI$^+$, m/z): m/z [M+H] calc'd for $C_{41}H_{54}N_7O_6S$ 772.3851;

found 772.8328.

Synthesis of Compound 4

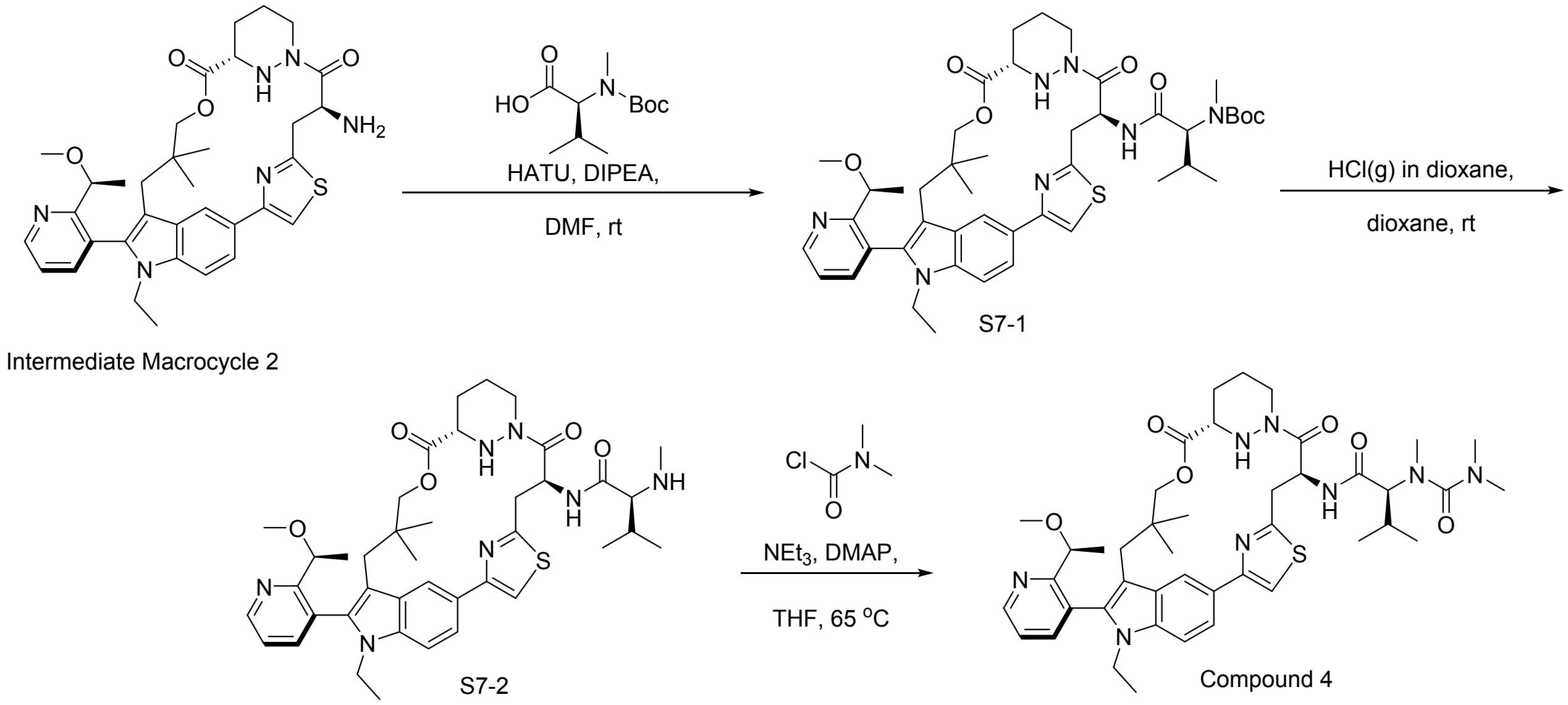

tert-Butyl ((2S)-1-(((6^{3}S,4S,Z)-11-ethyl-1^2-(2-((S)-1-methoxyethyl)pyridin-3-yl)-10,10-dimethyl-5,7-dioxo-6^1,6^2,6^3,6^4,6^5,6^6-hexahydro-1^{1}H-8-oxa-2(4,2)-thiazola-1(5,3)-indola-6(1,3)-pyridazinacycloundecaphane-4-yl)amino)-3-methyl-1-oxobutan-2-yl)(methyl)carbamate (S7-1) To a stirred solution of (6^3*S*,4*S*,*Z*)-4-amino-1^1-ethyl-1^2-(2-((*S*)-1-methoxyethyl)pyridin-3-yl)-10,10-dimethyl-6^1,6^2,6^3,6^4,6^5,6^6-hexahydro-1^1*H*-8-oxa-2(4,2)-thiazola-1(5,3)-indola-6(1,3)-pyridazinacycloundecaphane-5,7-dione (190 mg, 0.30 mmol) and DIPEA (389.29 mg, 3.01 mmol) in DMF (5 mL) were added (2*S*)-2-[(*tert*-butoxycarbonyl)(methyl)amino]-3-methylbutanoic acid (104.50 mg, 0.45 mmol) and HATU (229.05 mg, 0.60 mmol) in portions at 0 °C under an air atmosphere. The resulting mixture was stirred at room temperature for 1 h. The reaction was quenched with H_2O at 0 °C. The resulting mixture was extracted with EtOAc (3 x 15 mL). The combined organic layers were washed with brine (3 x 30 mL), dried over anhydrous Na_2SO_4. After filtration, the filtrate was concentrated under reduced pressure. The residue was purified by reversed-phase flash chromatography with the following conditions (Mobile Phase A: Water(10 mmol/L NH4HCO3), Mobile Phase B: ACN; Flow rate: 50 mL/min; Gradient: 0 % B to 100 % B in 30 min, 82 % B; Wave Length: 254/220 nm) to afford *tert*-butyl ((2*S*)-1-(((6^3*S*,4*S*,*Z*)-11-ethyl-1^2-(2-((*S*)-1-methoxyethyl)pyridin-3-yl)-10,10-dimethyl-5,7-dioxo-6^1,6^2,6^3,6^4,6^5,6^6-hexahydro-1^1*H*-8-oxa-2(4,2)-thiazola-1(5,3)-indola-6(1,3)-pyridazinacycloundecaphane-4-yl)amino)-3-methyl-1-oxobutan-2-yl)(methyl)carbamate (180 mg, 65 % yield) as a solid. ^{1}H NMR (400 MHz, DMSO-d_6) δ 8.83 – 8.69 (m, 1H), 8.51 (s, 2H), 7.91 – 7.71 (m, 3H), 7.64 – 7.46 (m, 2H), 5.62 – 5.36 (m, 1H), 5.11 (t, *J* = 13.6 Hz, 1H), 4.49 – 3.97 (m, 6H), 3.72 – 3.49 (m, 2H), 3.33 (s, 3H), 2.98 (d, *J* = 14.4 Hz, 1H), 2.86 – 2.656 (m, 4H), 2.40 (d, *J* = 14.3 Hz, 1H), 2.17 – 1.96 (m, 2H), 1.88 – 1.70 (m, 2H), 1.64 – 1.45 (m, 10H), 1.43 –

1.28 (m, 4H), 1.01 – 0.70 (m, 12H), 0.32 (s, 3H). ^{13}C NMR (101 MHz, DMSO-d_6) δ 172.30, 170.85, 166.87, 166.70, 159.26, 155.92, 155.39, 154.61, 150.11, 140.58, 136.95, 136.46, 135.62, 130.13, 129.95, 129.38, 128.54, 127.17, 126.40, 123.65, 119.75, 117.41, 111.88, 111.51, 111.03, 79.45, 79.26, 76.17, 70.63, 64.28, 63.12, 57.74, 56.06, 49.73, 38.07, 37.44, 35.70, 32.82, 30.26, 29.83, 28.89, 28.67, 27.41, 27.07, 26.16, 24.83, 23.43, 19.86, 19.20, 16.47, 15.11. HRMS (ESI$^+$, m/z): m/z [M+H] calc'd for $C_{45}H_{61}N_7O_7S$ 844.44259; found 844.44092.

(2S)-N-((6^{3}S,4S,Z)-1^{1}-Ethyl-1^{2}-(2-((S)-1-methoxyethyl)pyridin-3-yl)-10,10-dimethyl-5,7-dioxo-6^{1},6^{2},6^{3},6^{4},6^{5},6^{6}-hexahydro-1^{1}H-8-oxa-2(4,2)-thiazola-1(5,3)-indola-6(1,3)-pyridazinacycloundecaphane-4-yl)-3-methyl-2-(methylamino)butanamide; hydrogen chloride salt (S7-2) To a stirred solution of *tert*-butyl ((2*S*)-1-(((6^{3}*S*,4*S*,*Z*)-11-ethyl-1^{2}-(2-((*S*)-1-methoxyethyl)pyridin-3-yl)-10,10-dimethyl-5,7-dioxo-6^{1},6^{2},6^{3},6^{4},6^{5},6^{6}-hexahydro-1^{1}*H*-8-oxa-2(4,2)-thiazola-1(5,3)-indola-6(1,3)-pyridazinacycloundecaphane-4-yl)amino)-3-methyl-1-oxobutan-2-yl)(methyl)carbamate (100 mg, 0.12 mmol) in 1,4-dioxane (3 mL) was added HCl(gas)in 1,4-dioxane (4 M, 3 mL) in portions at 0 °C under an air atmosphere. The resulting mixture was stirred at room temperature for 1 h. The resulting mixture was concentrated under reduced pressure. The crude product (80 mg, crude) was used in the next step directly without further purification. ^{1}H NMR (400 MHz, Methanol-d_4) δ 9.18 – 8.84 (m, 2H), 8.58 (s, 1H), 8.44 – 8.21 (d, *J* = 9.1 Hz, 1H), 7.93 – 7.48 (m, 3H), 5.94 (d, *J* = 8.0 Hz, 1H), 4.82 – 4.63 (m, 1H), 4.56 – 4.35 (m, 3H), 4.08 – 3.86 (m, 2H), 3.84 – 3.76 (m, 1H), 3.76 – 3.71 (m, 2H), 3.70 – 3.62 (m, 4H), 3.61 – 3.56 (m, 2H), 3.54 (s, 3H), 2.96 – 2.80 (m, 1H), 2.79 – 2.68 (m, 1H), 2.62 (s, 3H), 2.38 – 2.04 (m, 2H), 2.00 – 1.89 (m, 1H), 1.88– 1.76 (m, 1H), 1.72 – 1.58 (m, 1H), 1.56 – 1.42 (m, 3H), 1.29 (s, 1H), 1.11 (d, *J* = 6.7 Hz, 3H), 1.05 (d, *J* = 6.9 Hz, 4H), 0.87 (s, 3H), 0.63 (s, 3H). ^{13}C NMR (101 MHz, Methanol-d_4) δ 171.91, 171.12, 166.54, 154.89, 150.94, 143.05, 136.92, 131.45, 130.28, 129.88, 127.19, 120.98, 115.54, 112.73, 111.33, 73.52, 72.18, 71.06, 66.87, 62.92, 60.80, 58.39, 56.45, 50.98, 42.49, 41.28, 38.96, 36.01, 32.19, 30.12, 29.37, 28.48, 24.80, 24.23, 23.06, 17.41, 17.17, 17.03, 13.92. HRMS (ESI$^+$, m/z): m/z [M+H] calc'd for $C_{40}H_{53}N_7O_5S$ 744.39016; found 744.38898.

(2S)-N-((6^{3}S,4S,Z)-1^{1}-Ethyl-1^{2}-(2-((S)-1-methoxyethyl)pyridin-3-yl)-10,10-dimethyl-5,7-dioxo-6^{1},6^{2},6^{3},6^{4},6^{5},6^{6}-hexahydro-1^{1}H-8-oxa-2(4,2)-thiazola-1(5,3)-indola-6(1,3)-pyridazinacycloundecaphane-4-yl)-3-methyl-2-(1,3,3-trimethylureido)butanamide (Compound 4) To a stirred mixture of (2*S*)-*N*-((6^{3}*S*,4*S*,*Z*)-1^{1}-ethyl-1^{2}-(2-((*S*)-1-methoxyethyl)pyridin-3-yl)-10,10-dimethyl-5,7-dioxo-6^{1},6^{2},6^{3},6^{4},6^{5},6^{6}-hexahydro-1^{1}*H*-8-oxa-2(4,2)-thiazola-1(5,3)-indola-6(1,3)-pyridazinacycloundecaphane-4-yl)-3-methyl-2-(methylamino)butanamide; hydrogen chloride salt (80 mg, 0.11 mmol) and NEt_3 (108.81 mg, 1.08 mmol) in THF (5 mL) was added DMAP (13.14 mg, 0.11 mmol) and in portions at 0 °C under an argon atmosphere. The resulting mixture was stirred at 0 °C for 10 min. To the above mixture was added

dimethylcarbamyl chloride (115.64 mg, 1.08 mmol) in portions at 0 °C. The resulting mixture was stirred at 65 °C for an additional 16 h. The reaction was quenched with H_2O at 0 °C and extracted with EtOAc (3 x 30 mL). The combined organic layers were dried over anhydrous Na_2SO_4. After filtration, the filtrate was concentrated under reduced pressure. The residue was purified by reversed-phase flash chromatography with the following conditions (Mobile Phase A: Water(10 mmol/L NH4HCO3), Mobile Phase B: ACN; Flow rate: 40 mL/min; Gradient: 0 % B to100 % B in 30 min, 85 % B; Wave Length: 254/220 nm) to afford(2*S*)-*N*-((6^3*S*,4*S*,*Z*)-1^1-ethyl-1^2-(2-((S)-1-methoxyethyl)pyridin-3-yl)-10,10-dimethyl-5,7-dioxo-6^1,6^2,6^3,6^4,6^5,6^6-hexahydro-1^1*H*-8-oxa-2(4,2)-thiazola-1(5,3)-indola-6(1,3)-pyridazinacycloundecaphane-4-yl)-3-methyl-2-(1,3,3-trimethylureido)butanamide (60 mg, 63% yield) as a solid. ^{1}H NMR (400 MHz, DMSO-d_6) δ 8.78 – 8.73 (m, 1H), 8.55 – 8.30 (m, 2H), 7.90 – 7.67 (m, 3H), 7.64 – 7.43 (m, 2H), 5.55 (t, *J* = 8.9 Hz, 1H), 5.14 (d, *J* = 12.1 Hz, 1H), 4.05 – 4.45 (m, 5H), 3.87 (d, *J* = 10.9 Hz, 1H), 3.66 – 3.50 (m, 2H), 3.40 – 3.30 (m, 4H), 3.17 – 3.03 (m, 1H), 2.96 (d, *J* = 14.4 Hz, 1H), 2.88 – 2.70 (m, 10H), 2.40 (d, *J* = 14.3 Hz, 1H), 2.10 – 2.05 (m, 2H), 1.87 – 1.70 (m, 2H), 1.61 – 1.45 (m, 1H), 1.37 (d, *J* = 6.0 Hz, 3H), 1.23 (s, 1H), 0.99 – 0.74 (m, 12H), 0.32 (s, 3H). ^{13}C NMR (101 MHz, DMSO-d_6) δ 172.27, 170.89, 166.50, 165.61, 159.25, 154.75, 150.12, 140.60, 136.96, 135.64, 130.12, 127.16, 126.35, 123.66, 119.73, 117.39, 111.89, 111.61, 111.06, 76.17, 70.62, 65.66, 57.76, 56.07, 49.25, 40.73, 39.04, 38.28, 38.08, 35.70, 33.23, 32.81, 28.90, 26.52, 26.15, 24.82, 23.40, 20.27, 19.47, 16.50, 15.11. HRMS (ESI$^+$, m/z): m/z [M+H] calc'd for $C_{43}H_{58}N_8O_6S$ 815.42728; found 815.42578.

Synthesis of Compound 5

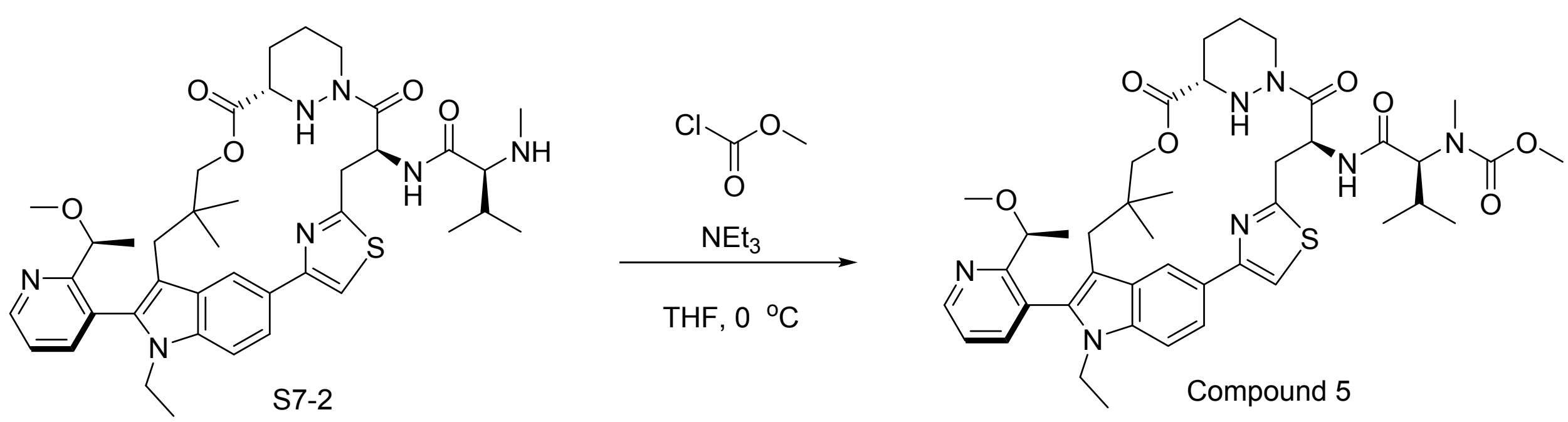

Methyl ((2S)-1-(((6^3S,4S,Z)-1^1-ethyl-1^2-(2-((S)-1-methoxyethyl)pyridin-3-yl)-10,10-dimethyl-5,7-dioxo-6^1,6^2,6^3,6^4,6^5,6^6-hexahydro-1^1H-8-oxa-2(4,2)-thiazola-1(5,3)-indola-6(1,3)-pyridazinacycloundecaphane-4-yl)amino)-3-methyl-1-oxobutan-2-yl)(methyl)carbamate (Compound 5) To a stirred mixture of (2*S*)-*N*-((6^3*S*,4*S*,*Z*)-1^1-ethyl-1^2-(2-((S)-1-methoxyethyl)pyridin-3-yl)-10,10-dimethyl-5,7-dioxo-6^1,6^2,6^3,6^4,6^5,6^6-hexahydro-1^1*H*-8-oxa-2(4,2)-thiazola-1(5,3)-indola-6(1,3)-pyridazinacycloundecaphane-4-yl)-3-methyl-2-(methylamino)butanamide (100.00 mg, 0.13 mmol) and NEt_3 (40.80 mg, 0.40 mmol) in THF (1.00 mL) was added methyl chloroformate (15.24 mg, 0.16 mmol) at 0 °C under a nitrogen atmosphere. The

resulting mixture was stirred for 2 h at room temperature. The resulting mixture was diluted with H_2O (6 mL).The aqueous layer was extracted with DCM (3 x 8 mL).The resulting mixture was concentrated under reduced pressure. The crude product (100 mg) was purified by reverse phase chromatography with the following conditions (Column: Venusil XBP C18(2) , 50 x 250mm 10um; Mobile Phase A:Water, Mobile Phase B:ACN; Flow rate:100 mL/min; Gradient:45 B to 75 B in 30 min; 254/220 nm; RT1:23)to afford methyl ((2*S*)-1-(((6^3*S*,4*S*,*Z*)-1^1-ethyl-1^2-(2-((*S*)-1-methoxyethyl)pyridin-3-yl)-10,10-dimethyl-5,7-dioxo-6^1,6^2,6^3,6^4,6^5,6^6-hexahydro-1^1*H*-8-oxa-2(4,2)-thiazola-1(5,3)-indola-6(1,3)-pyridazinacycloundecaphane-4-yl)amino)-3-methyl-1-oxobutan-2-yl)(methyl)carbamate (30 mg, 28% yield) as a solid. ^{1}H NMR (400 MHz, DMSO-d_6) δ 8.75 (dd, *J* = 4.7, 1.7 Hz, 1H), 8.57 (s, 1H), 8.49 (s, 1H), 7.85 (s, 1H), 7.77 (ddd, *J* = 18.0, 8.1, 1.7 Hz, 2H), 7.57 (d, *J* = 8.7 Hz, 1H), 7.52 (dd, *J* = 7.7, 4.7 Hz, 1H), 5.45 (s, 1H), 5.08 (d, *J* = 12.2 Hz, 1H), 4.31 (dq, *J* = 12.3, 6.7, 6.1 Hz, 3H), 4.25 – 4.07 (m, 3H), 3.69 (s, 5H), 3.62 – 3.51 (m, 4H), 2.96 (d, *J* = 14.9 Hz, 1H), 2.82 (s, 3H), 2.76 (s, 1H), 2.39 (d, *J* = 14.4 Hz, 2H), 2.07 (td, *J* = 12.8, 6.6 Hz, 2H), 1.79 (s, 2H), 1.52 (d, *J* = 10.1 Hz, 1H), 1.36 (d, *J* = 6.0 Hz, 6H), 0.90 (s, 4H), 0.85 (d, *J* = 7.0 Hz, 1H), 0.83 (s, 5H), 0.32 (s, 3H). LCMS (ESI): m/z [M+H] calc'd for $C_{43}H_{58}N_8O_6S$ 802.39; found 802.6.

Synthesis of Intermediate 3

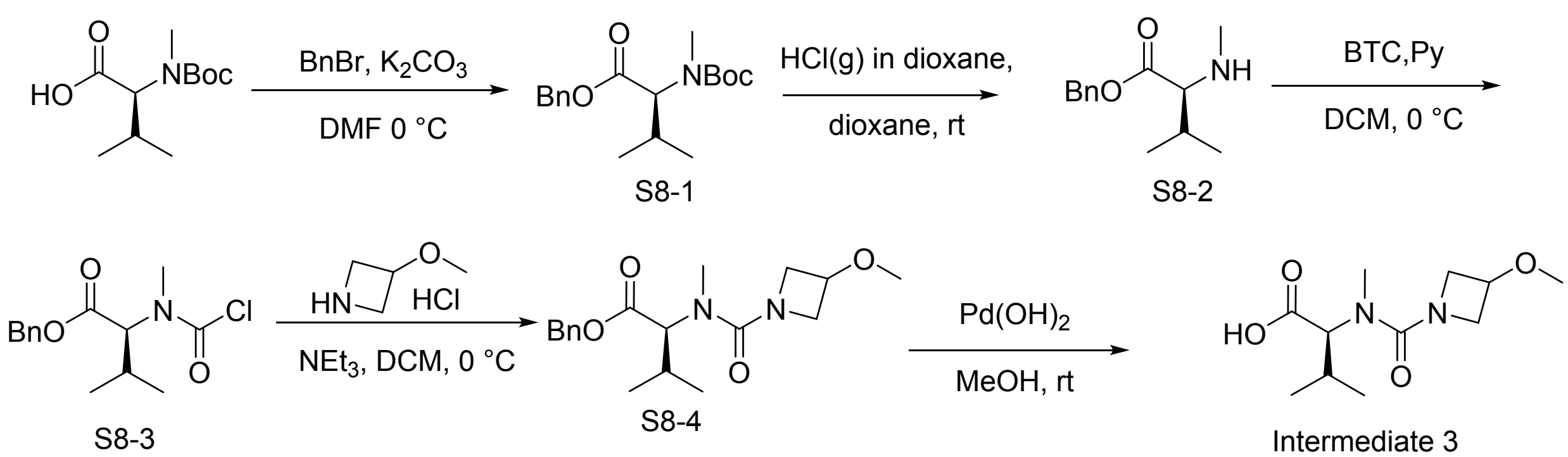

Benzyl (2S)-2-[(tert-butoxycarbonyl)(methyl)amino]-3-methylbutanoate (S8-1) A solution of (2*S*)-2-[(*tert*-butoxycarbonyl)(methyl)amino]-3-methylbutanoic acid (2 g, 8.65 mmol) in DMF (20 mL) was treated with K_2CO_3 (1.79 g, 12.97 mmol) at 0 °C for 2 min under a nitrogen atmosphere followed by the addition of BnBr (1.77 g, 10.38 mmol) in portions. After 2 min, the resulting solution was warmed to room temperature and then stirred for 2 h. The reaction was quenched by the addition of H_2O (10 mL) at 0 °C. The resulting mixture was extracted with EtOAc (3 x 20 mL). The combined organic layers were washed with brine (3 x 20 mL), dried over anhydrous Na_2SO_4. After filtration, the filtrate was concentrated under reduced pressure. The residue was purified by silica gel column chromatography, eluted with Pet. Ether / EtOAc (10:1) to afford benzyl (2*S*)-2-[(*tert*-butoxycarbonyl)(methyl)amino]-3-methylbutanoate (2.4 g, 86% yield) as an oil. ^{1}H NMR (400 MHz, DMSO-d_6) δ 7.41 – 7.28 (m, 5H), 5.22 – 5.07 (m, 2H), 4.18

(dd, *J* = 109.3, 10.3 Hz, 1H), 2.74 (d, *J* = 11.5 Hz, 3H), 2.26 – 2.02 (m, 1H), 1.36 (d, *J* = 24.9 Hz, 9H), 0.97 – 0.76 (m, 6H). ^{13}C NMR (101 MHz, DMSO) δ 170.92, 155.80, 155.06, 136.39, 128.85, 128.51, 128.33, 128.26, 79.76, 66.25, 66.17, 31.12, 28.37, 28.32, 27.54, 20.37, 20.03. HRMS (ESI$^+$, m/z): m/z [M+H] calc'd for $C_{18}H_{28}NO_4$ 322.20128; found 322.20059.

Benzyl methyl-L-valinate (S8-2) Into a 3-L round-bottom flask, was placed benzyl *N*-(*tert*-butoxycarbonyl)-*N*-methyl-*L*-valinate (920.00 g, 2862.33 mmol), dioxane (500.00 mL).
To the above solution was added HCl(gas) in 1,4-dioxane (1.00 L, 17517.48 mmol) dropwise at 0 °C. The resulting solution was stirred for 8 h at room temperature. The crude product was purified by re-crystallization from Pet. ether. The solids were collected by filtration. This resulted in 446 g (crude) of benzyl methyl-*L*-valinate as a solid. ^{1}H NMR (400 MHz, DMSO-d_6) δ 7.44 – 7.24 (m,5H), 5.15 (d, *J* = 1.8 Hz,2H), 2.85 (d, *J* = 6.5 Hz, 1H), 2.22 (s, 3H), 1.87 –1.78 (m, 2H), 0.86 (dd, *J* = 10.0, 6.8 Hz, 6H). ^{13}C NMR (101 MHz, DMSO) δ 174.75, 136.66, 128.86, 128.61, 128.61, 128.50, 69.31, 65.85, 35.11, 31.38, 19.63, 19.14. HRMS (ESI$^+$, m/z): m/z [M+H] calc'd for $C_{13}H_{19}NO_2$ 222.14886; found 222.14891.

Benzyl N-(chlorocarbonyl)-N-methyl-L-valinate (S8-3) Into a 500-mL round-bottom flask, was placed benzyl methyl-*L*-valinate (22.60 g, 102.12 mmol), pyridine (16.44 mL, 207.84 mmol), DCM (200.00 mL). To the above mixture was added triphosgene (10.00 g, 33.701 mmol) in DCM dropwise at 0 °C. The resulting solution was stirred for 1 h at room temperature. The mixture was concentrated and precipitated by the addition of EtOAc. The filtrate was collected and concentrated under reduced pressure. This resulted in benzyl *N*-(chlorocarbonyl)-*N*-methyl-*L*-valinate (25 g, crude) as a solid. The crude product was used in the next step directly without further purification. ^{1}H NMR (400 MHz, DMSO-d_6) δ 7.48 – 7.36 (m, 5H), 5.33 – 5.19 (m, 2H), 3.85 (d, *J* = 9.5 Hz, 1H), 2.90 (s, 3H), 2.18 –2.09 (m, 1H), 1.07 – 0.85 (m, 6H). ^{13}C NMR (101 MHz, DMSO) δ 171.71, 135.55, 129.27, 129.18, 129.06, 129.02, 128.99, 128.92, 72.18, 67.64, 34.69, 29.18, 19.69, 19.26. LCMS (ESI): m/z [M+H] calc'd for $C_{14}H_{18}ClNO_3$ 283.0; found 284.0.

Benzyl N-(3-methoxyazetidine-1-carbonyl)-N-methyl-L-valinate (S8-4) Into a 1-L 3-necked round-bottom flask purged and maintained with a nitrogen atmosphere, was placed 3-methoxyazetidine (15.35 g, 100.00 mmol), NEt_3 (122.46 mL, 881.06 mmol), DCM (250.00 mL).To the above mixture was added benzyl *N*-(chlorocarbonyl)-*N*-methyl-*L*-valinate (25.00 g, 88.106 mmol) in DCM dropwise at 0 °C. The resulting solution was stirred for 1 h at room temperature. The resulting mixture was washed with H_2O (3 x 200mL). The aqueous layer was extracted with DCM (3 x 150
mL). The residue was purified by silica gel column chromatography, eluted with Pet. ether/EtOAc, which resulted in 20.8 g (71%, yield) of benzyl *N*-(3-methoxyazetidine-1-carbonyl)-*N*-methyl-*L*-valinate as an

oil. ^{1}H NMR (400 MHz, DMSO-d_6) δ 7.45 – 7.27 (m, 5H), 5.18 – 5.07 (m, 2H), 4.23 (d, J = 10.4 Hz, 1H), 4.14 – 3.95 (m, 3H), 3.74 – 3.62 (m, 2H), 3.17 (s, 3H), 2.73 (s, 3H), 2.20 – 2.07 (m, 1H), 0.86 (dd, J = 30.1, 6.6 Hz, 6H). ^{13}C NMR (101 MHz, DMSO) δ 171.44, 162.87, 136.42, 128.89, 128.54, 128.46, 69.06, 66.16, 64.03, 58.11, 58.03, 55.66, 31.70, 27.55, 20.01, 19.48. HRMS (ESI$^+$, m/z): m/z [M+H] calc'd for $C_{18}H_{27}N_2O_4$ 335.19653; found 335.19623.

N-(3-Methoxyazetidine-1-carbonyl)-N-methyl-L-valine (Intermediate 3) Into a 250-mL round-bottom flask, was placed benzyl *N*-(3-methoxyazetidine-1-carbonyl)-*N*-methyl-*L*-valinate (20.00 g), MeOH (150.00 mL), $Pd(OH)_2/C$ (20.00 g). The resulting solution was stirred under an H_2 atmosphere for 5 h at room temperature. The solids were filtered out. The resulting mixture was concentrated under reduced pressure, which resulted in *N*-(3-methoxyazetidine-1-carbonyl)-*N*-methyl-*L*-valine (15 g, crude) as an oil. ^{1}H NMR (400 MHz, DMSO-d_6) δ 4.15 – 3.89 (m, 4H), 3.76 – 3.70 (m, 1H), 3.67 – 3.60 (m, 1H), 3.18 (d, J = 4.5 Hz, 3H), 2.73 (s, 3H), 2.10 – 1.95 (m, 1H), 0.92 (d, J = 6.5 Hz, 3H), 0.78 (d, J = 6.8 Hz, 3H). ^{13}C NMR (101 MHz, DMSO) δ 173.84, 163.18, 69.10, 65.33, 58.18, 57.98, 55.63, 31.29, 27.60, 20.47, 19.66. HRMS (ESI$^+$, m/z): m/z [M+H] calc'd for $C_{11}H_{20}N_2O_4$ 245.14958; found 245.14964.

Synthesis of Compound 6

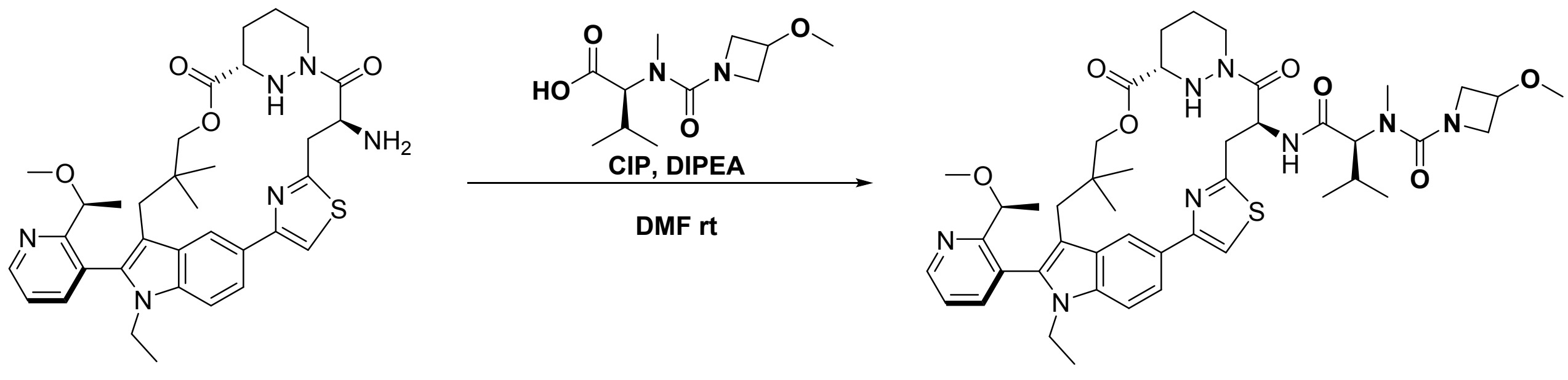

N-((2S)-1-(((6³S,4S,Z)-1¹-Ethyl-1²-(2-((S)-1-methoxyethyl)pyridin-3-yl)-10,10-dimethyl-5,7-dioxo-6¹,6²,6³,6⁴,6⁵,6⁶-hexahydro-11H-8-oxa-2(4,2)-thiazola-1(5,3)-indola-6(1,3)-pyridazinacycloundecaphane-4-yl)amino)-3-methyl-1-oxobutan-2-yl)-3-methoxy-N-methylazetidine-1-carboxamide To a solution of (6³*S*,4*S*,*Z*)-4-amino-1¹-ethyl-1²-(2-((*S*)-1-methoxyethyl)pyridin-3-yl)-10,10-dimethyl-6¹,6²,6³,6⁴,6⁵,6⁶-hexahydro-11*H*-8-oxa-2(4,2)-thiazola-1(5,3)-indola-6(1,3)-pyridazinacycloundecaphane-5,7-dione (12 g, 19.023 mmol), *N*-(3-methoxyazetidine-1-carbonyl)-*N*-methyl-*L*-valine (13.94 g, 57.063 mmol) and DIPEA (165.67 mL, 1281.884 mmol) in DMF (500 mL) was added CIP (15.87 g, 57.086 mmol) at room temperature . The resulting solution was stirred overnight. The reaction was quenched with H_2O (100 mL). The resulting mixture was extracted with EtOAc (3 x 200 mL). The organic layers were combined,

dried over anhydrous Na_2SO_4, filtered, concentrated and purified by reverse-phase chromatography with the following conditions: column, Cat No: SO230330-2, C18, 330 g,20-35µm, 100 Å, Lot: BQ0003Q08; mobile phase,MeCN:H_2O (0.05% TFA) = 5% increasing to MeCN:H_2O (0.05% TFA) = 100 % within 30 minutes, hold CH_3CN:H_2O (0.05 % TFA) = 50 % within 30 minutes; Detector,UV 254 nm. This resulted in N-((2S)-1-(((6^3S,4S,Z)-1^1-ethyl-1^2-(2-((S)-1-methoxyethyl)pyridin-3-yl)-10,10-dimethyl-5,7-dioxo-6^1,6^2,6^3,6^4,6^5,6^6-hexahydro-11H-8-oxa-2(4,2)-thiazola-1(5,3)-indola-6(1,3)-pyridazinacycloundecaphane-4-yl)amino)-3-methyl-1-oxobutan-2-yl)-3-methoxy-N-methylazetidine-1-carboxamide (10 g, 61% yield) as a yellow solid. ^{1}H NMR (400 MHz, DMSO-d_6) δ 8.76 (dd, J = 4.8, 1.7 Hz, 1H), 8.49 (d, J = 1.6 Hz, 1H), 8.36 (d, J = 8.5 Hz, 1H), 7.85 – 7.71 (m, 3H), 7.62 – 7.48 (m, 2H), 5.54 – 5.45 (m, 1H), 5.11 (d, J = 12.2 Hz, 1H), 4.40 – 4.27 (m, 2H), 4.26 – 4.07 (m, 7H), 3.88 – 3.82 (m, 1H), 3.81 – 3.74 (m, 1H), 3.61 – 3.52 (m, 2H), 3.30 – 3.20 (m, 8H), 2.97 (d, J = 14.4 Hz, 1H), 2.76 (s, 4H), 2.40 (d, J = 14.3 Hz, 1H), 2.17 – 2.03 (m, 2H), 1.79 (s, 2H), 1.60 – 1.45 (m, 1H), 1.37 (d, J = 6.0 Hz, 3H), 0.91 (d, J = 5.6 Hz, 6H), 0.87 – 0.76 (m, 6H), 0.32 (s, 3H). ^{13}C NMR (101 MHz, DMSO) δ 172.34, 172.27, 170.88, 166.78, 163.44, 159.25, 154.64, 150.11, 140.58, 136.96, 135.63, 130.13, 127.16, 126.37, 123.65, 119.74, 117.40, 111.88, 111.55, 111.03, 76.17, 70.63, 69.17, 63.71, 60.22, 58.22, 58.10, 57.74, 56.06, 55.75, 49.63, 38.08, 37.58, 35.69, 32.81, 31.20, 28.91, 26.81, 26.16, 24.81, 23.45, 20.14, 19.30, 16.47, 15.10. HRMS (ESI$^+$, m/z): m/z [M+H] calc'd for $C_{45}H_{61}N_8O_7S$ 857.43784; found 857.43652.

Synthesis of Compound 7

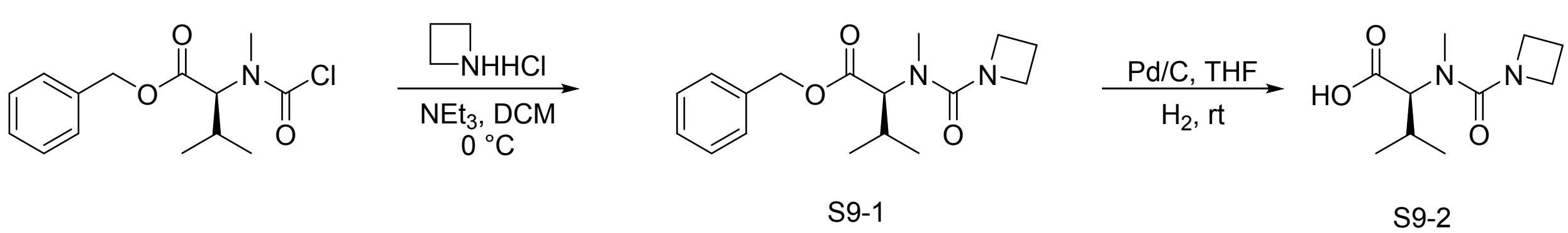

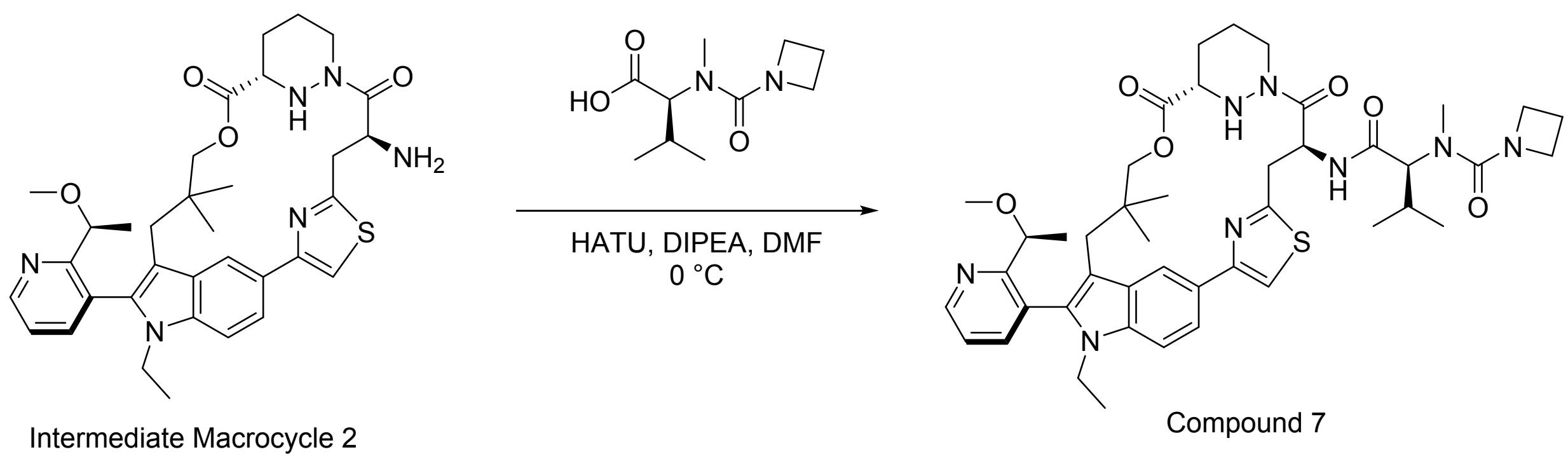

benzyl N-(Azetidine-1-carbonyl)-N-methyl-L-valinate (S9-1) To a stirred solution of azetidine hydrochloride (98.91 mg, 1.06 mmol) and NEt_3 (427.94 mg, 4.23 mmol) in DCM (2.00 mL) was added

benzyl *N*-(chlorocarbonyl)-*N*-methyl-*L*-valinate (300.00 mg, 1.06 mmol) dropwise at 0 °C under a nitrogen atmosphere. The resulting mixture was stirred for 4 h at room temperature under nitrogen atmosphere. The reaction solution was purified by Prep-TLC (Pet. ether/EtOAc 1:1) to afford benzyl *N*-(azetidine-1-carbonyl)-*N*-methyl-*L*-valinate (200 mg, 62% yield) as an oil. LCMS (ESI): m/z [M+H] calc'd for $C_{17}H_{24}N_2O_3$ 305.18; found 305.2.

N-(Azetidine-1-carbonyl)-N-methyl-L-valine (S9-2) To a solution of benzyl *N*-(azetidine-1-carbonyl)-*N*-methyl-*L*-valinate (200.00 mg, 0.66 mmol) in THF (10.00 mL) was added Pd/C (699.23 mg, 0.007 mmol) under a nitrogen atmosphere in a 50 mL round-bottom flask. The mixture was hydrogenated at room temperature overnight using a hydrogen balloon, filtered through a Celite pad and concentrated under reduced pressure to afford *N*-(azetidine-1-carbonyl)-*N*-methyl-*L*-valine (120 mg, 85% yield) as an oil. The crude product was used in the next step directly without further purification. LCMS (ESI): m/z [M+H] calc'd for $C_{10}H_{18}N_2O_3$ 215.13; found 215.2.

N-((2S)-1-(((6^{3}S,4S,Z)-1^1-Ethyl-1^2-(2-((S)-1-methoxyethyl)pyridin-3-yl)-10,10-dimethyl-5,7-dioxo-6^1,6^2,6^3,6^4,6^5,6^6-hexahydro-1^{1}H-8-oxa-2(4,2)-thiazola-1(5,3)-indola-6(1,3)-pyridazinacycloundecaphane-4-yl)amino)-3-methyl-1-oxobutan-2-yl)-N-methylazetidine-1-carboxamide (Compound 7) Into a 8 mL vial were added (6^3*S*,4*S*,*Z*)-4-amino-1^1-ethyl-1^2-(2-((*S*)-1-methoxyethyl)pyridin-3-yl)-10,10-dimethyl-6^1,6^2,6^3,6^4,6^5,6^6-hexahydro-1^1*H*-8-oxa-2(4,2)-thiazola-1(5,3)-indola-6(1,3)-pyridazinacycloundecaphane-5,7-dione (200.00 mg, 0.32 mmol), *N*-(azetidine-1-carbonyl)-*N*-methyl-*L*-valine (81.52 mg, 0.38 mmol), HATU (180.83 mg, 0.48 mmol), DIPEA (204.88 mg, 1.59 mmol) and DMF (2.00 mL) at 0 °C. The resulting mixture was stirred for 2 h at room temperature under a nitrogen atmosphere. The reaction was purified by Prep-HPLC with the following conditions (Column: Welch Ultimate XB-C18, 50 x 250mm 10um; Mobile Phase A:Water, Mobile Phase B:ACN; Flow rate:100 mL/min; Gradient:35 B to 70 B in 30 min; 254/220 nm; RT1:27) to afford *N*-((2*S*)-1-(((6^3*S*,4*S*,*Z*)-1^1-ethyl-1^2-(2-((*S*)-1-methoxyethyl)pyridin-3-yl)-10,10-dimethyl-5,7-dioxo-6^1,6^2,6^3,6^4,6^5,6^6-hexahydro-1^1*H*-8-oxa-2(4,2)-thiazola-1(5,3)-indola-6(1,3)-pyridazinacycloundecaphane-4-yl)amino)-3-methyl-1-oxobutan-2-yl)-*N*-methylazetidine-1-carboxamide (82.5 mg, 31% yield) as a solid. ^{1}H NMR (400 MHz, DMSO-d_6) δ 8.79 – 8.73 (dd, J = 4.7, 1.7 Hz, 1H), 8.52 – 8.45 (d, J = 1.7 Hz, 1H), 8.32 – 8.24 (d, J = 8.5 Hz, 1H), 7.89 – 7.82 (s, 1H), 7.82 – 7.70 (ddd, J = 16.9, 8.2, 1.7 Hz, 2H), 7.61 – 7.55 (d, J = 8.6 Hz, 1H), 7.55 – 7.49 (dd, J = 7.7, 4.8 Hz, 1H), 5.54 – 5.45 (t, J = 8.6, 8.6 Hz, 1H), 5.16 – 5.05 (d, J = 12.2 Hz, 1H), 4.43 – 4.26 (dq, J = 12.4, 6.7, 6.7, 6.2 Hz, 2H), 4.26 – 4.04 (m, 4H), 4.04 – 3.86 (dq, J = 21.4, 7.9, 7.8, 7.8 Hz, 4H), 3.63 – 3.48 (m, 2H), 3.31 – 3.24 (d, J = 13.0 Hz, 4H), 3.01 – 2.90 (d, J = 14.4 Hz, 1H), 2.83 – 2.70 (s, 4H), 2.46 – 2.35 (d, J = 14.3 Hz, 1H), 2.25 – 2.14 (p, J = 7.7, 7.7, 7.7, 7.7 Hz, 2H), 2.14 – 2.03 (m, 2H), 1.87 – 1.70 (s, 2H), 1.62 – 1.43 (m,

1H), 1.42 – 1.32 (d, J = 6.0 Hz, 3H), 1.02 – 0.68 (m, 12H), 0.43 – 0.22 (s, 3H). LCMS (ESI): m/z [M+H] calc'd for $C_{44}H_{58}N_8O_6S$ 827.1; found 827.5.

Synthesis of Compound 8

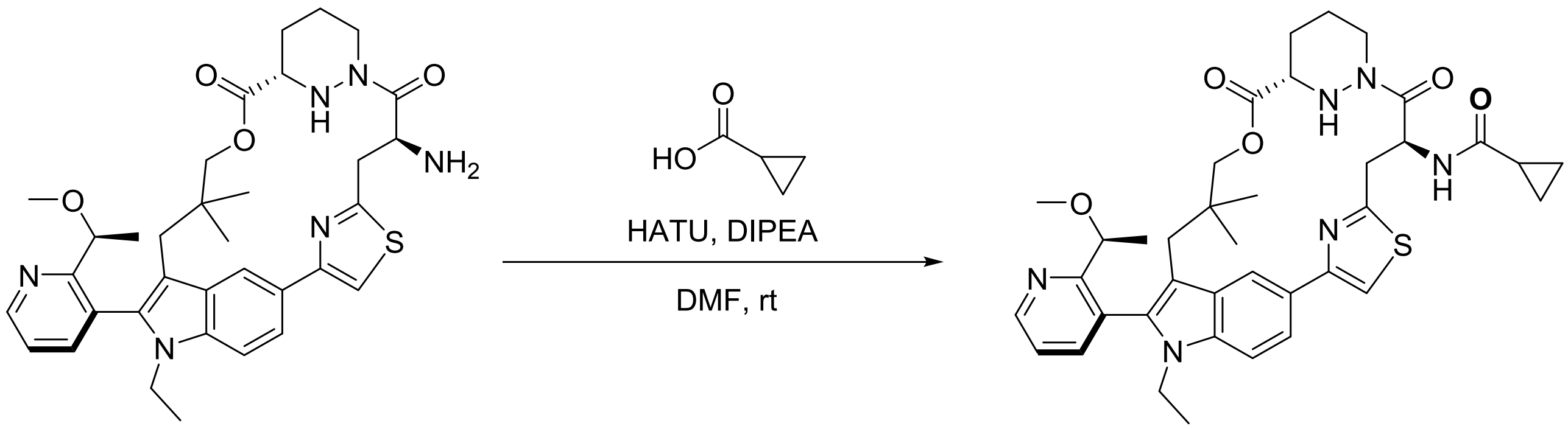

Intermediate Macrocycle 2

Compound 8

N-((6³S,4S,Z)-11-Ethyl-12-(2-((S)-1-methoxyethyl)36yridine-3-yl)-10,10-dimethyl-5,7-dioxo-6¹,6²,6³,6⁴,6⁵,6⁶-hexahydro-1¹H-8-oxa-2(4,2)-thiazola-1(5,3)-indola-6(1,3)-pyridazinacycloundecaphane-4-yl)cyclopropanecarboxamide (Compound 8) To a stirred solution of ((6³*S*,4*S*,*Z*)-4-amino-1¹-ethyl-12-(2-((*S*)-1-methoxyethyl)pyridin-3-yl)-10,10-dimethyl-6¹,6²,6³,6⁴,6⁵,6⁶-hexahydro-1¹*H*-8-oxa-2(4,2)-thiazola-1(5,3)-indola-6(1,3)-pyridazinacycloundecaphane-5,7-dione (200 mg, 0.32 mmol) and DIPEA (552.27 uL, 3.17 mmol) in DMF (5 mL) were added cyclopropanecarboxylic acid (35.48 mg, 0.41 mmol) and HATU (180.83 mg, 0.48 mmol) in portions at 0 °C . The resulting mixture was stirred for 1 h at room temperature. The reaction was quenched by the addition of H_2O (5 mL) at 0 °C. The resulting mixture was extracted with EtOAc (3 x 20 mL). The combined organic layers were washed with brine (3 x 30 mL), dried over anhydrous Na_2SO_4. After filtration, the filtrate was concentrated under reduced pressure. The crude product was purified by reverse phase flash with the following conditions (Mobile Phase A: H_2O (10mmol/L $NH_3.H_2O$), Mobile Phase B: MeCN; Flow rate: 60 mL/min; Gradient: 0 % B to 100 % B in 30 min, 55 % B ; Wave Length: 254nm/220nm nm) to afford *N*-((6³*S*,4*S*,*Z*)-11-ethyl-12-(2-((*S*)-1-methoxyethyl)pyridin-3-yl)-10,10-dimethyl-5,7-dioxo-6¹,6²,6³,6⁴,6⁵,6⁶-hexahydro-1¹*H*-8-oxa-2(4,2)-thiazola-1(5,3)-indola-6(1,3)-pyridazinacycloundecaphane-4-yl)cyclopropanecarboxamide (180 mg, 79% yield) as a solid. ^{1}H NMR (400 MHz, DMSO-d_6) δ 8.79 – 8.71 (m, 1H), 8.59 (d, J = 9.0 Hz, 1H), 8.51 (d, J = 1.6 Hz, 1H), 7.84 – 7.69 (m, 3H), 7.61 – 7.46 (m, 2H), 5.65 – 5.55 (m, 1H), 5.09 (d, J = 12.1 Hz, 1H), 4.46 – 4.01 (m, 5H), 3.58 (s, 3H), 3.38 – 3.03 (m, 3H), 3.21 – 3.12 (m, 1H), 2.96 (d, J = 14.4 Hz, 1H), 2.82 – 2.70 (m, 1H), 2.41 (d, J = 14.3 Hz, 1H), 2.14 – 2.01 (m, 1H), 1.88 – 1.68 (m, 3H), 1.60 – 1.44 (m, 1H), 1.37 (d, J = 6.0 Hz, 3H), 0.96 – 0.79 (m, 6H), 0.75 – 0.55 (m, 4H), 0.33 (s, 3H). ^{13}C NMR (101 MHz, DMSO-d_6) δ 173.17, 172.71, 172.31, 166.73, 159.28, 154.60, 150.11, 140.58, 136.94, 135.63, 130.13, 127.19, 126.40, 123.63, 119.73, 117.47, 111.89, 111.49, 111.02, 76.18, 70.64, 57.86,

56.07, 49.39, 40.67, 38.26, 38.10, 35.71, 32.83, 28.91, 26.13, 24.87, 23.42, 16.51, 15.12, 14.05, 6.97, 6.87. HRMS (ESI$^+$, m/z): m/z [M+H] calc'd for $C_{38}H_{46}N_6O_5S$ 699.33232; found 699.33118.

Synthesis of Compound 9

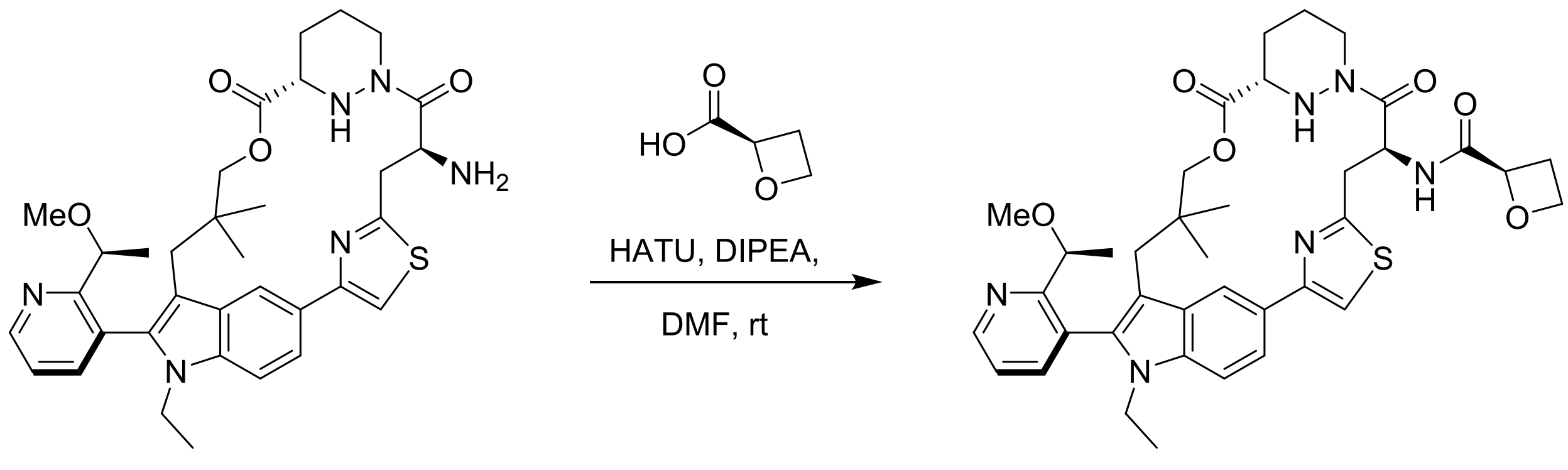

Intermediate macrocyle 2

Compound 9

(2R)-N-((6^{3}S,4S,Z)-1^1-Ethyl-1^2-(2-((S)-1-methoxyethyl)pyridin-3-yl)-10,10-dimethyl-5,7-dioxo-6^1,6^2,6^3,6^4,6^5,6^6-hexahydro-1^{1}H-8-oxa-2(4,2)-thiazola-1(5,3)-indola-6(1,3)-pyridazinacycloundecaphane-4-yl)oxetane-2-carboxamide (Compound 9) To a stirred solution of (6^3*S*,4*S*,*Z*)-4-amino-1^1-ethyl-1^2-(2-((*S*)-1-methoxyethyl)pyridin-3-yl)-10,10-dimethyl-6^1,6^2,6^3,6^4,6^5,6^6-hexahydro-1^1*H*-8-oxa-2(4,2)-thiazola-1(5,3)-indola-6(1,3)-pyridazinacycloundecaphane-5,7-dione (200 mg, 0.32 mmol) and DIPEA (122.93 mg, 0.95 mmol) in DMF (5 mL) were added (2*R*)-oxetane-2-carboxylic acid (48.55 mg, 0.48 mmol) and HATU (180.83 mg, 0.48 mmol) in portions at 0 °C under an air atmosphere. The resulting mixture was stirred for 1 h at room temperature. The reaction was quenched by the addition of H_2O (5 mL) at 0 °C. The resulting mixture was extracted with EtOAc (2 x 5 mL). The combined organic layers were washed with brine (3 x 20 mL), dried over anhydrous Na_2SO_4. After filtration, the filtrate was concentrated under reduced pressure. The residue was purified by reverse phase flash chromatography with the following conditions (Column:C18 Column; Mobile Phase A: Water (0.05%NH3.H2O), Mobile Phase B: ACN; Flow rate: 50 mL/min; Gradient: 0% B to 100% B in 25 min, 75% B; Wave Length: 254/220 nm; RT1(min): 5; Number of Runs: 0) to afford (2*R*)-N-((6^3*S*,4*S*,*Z*)-1^1-ethyl-1^2-(2-((*S*)-1-methoxyethyl)pyridin-3-yl)-10,10-dimethyl-5,7-dioxo-6^1,6^2,6^3,6^4,6^5,6^6-hexahydro-1^1*H*-8-oxa-2(4,2)-thiazola-1(5,3)-indola-6(1,3)-pyridazinacycloundecaphane-4-yl)oxetane-2-carboxamide (177 mg, 78% yield) as a solid. ^{1}H NMR (400 MHz, DMSO-d_6) δ 8.76 (dd, J = 4.8, 1.7 Hz, 1H), 8.50 (d, J = 1.6 Hz, 1H), 8.32 (d, J = 9.3 Hz, 1H), 7.82 – 7.77 (m, 2H), 7.75 (dd, J = 8.6, 1.6 Hz, 1H), 7.58 (d, J = 8.6 Hz, 1H), 7.52 (dd, J = 7.7, 4.7 Hz, 1H), 5.59 (t, J = 9.0 Hz, 1H), 5.17 (d, J = 12.2 Hz, 1H), 5.03 (dd, J = 8.9, 6.5 Hz, 1H), 4.62 (ddt, J = 20.6, 8.8, 6.0 Hz, 2H), 4.39 – 4.19 (m, 4H), 4.18 – 4.02 (m, 1H), 3.63 – 3.52

(m, 2H), 3.46 – 3.34 (m, 1H), 3.26 (s, 3H), 2.94 (ddt, *J* = 11.0, 8.8, 1.9 Hz, 2H), 2.82 – 2.73 (m, 1H), 2.47 – 2.32 (m, 2H), 2.15 – 2.04 (m, 1H), 1.81 (s, 2H), 1.55 – 1.46 (m, 1H), 1.37 (d, *J* = 6.0 Hz, 3H), 0.93 – 0.79 (m, 6H), 0.33 (s, 3H). HRMS (ESI$^+$, m/z): m/z [M+H] calc'd for $C_{38}H_{47}N_6O_6S$ 715.3272; found 715.3251.

Synthesis of Compound 10

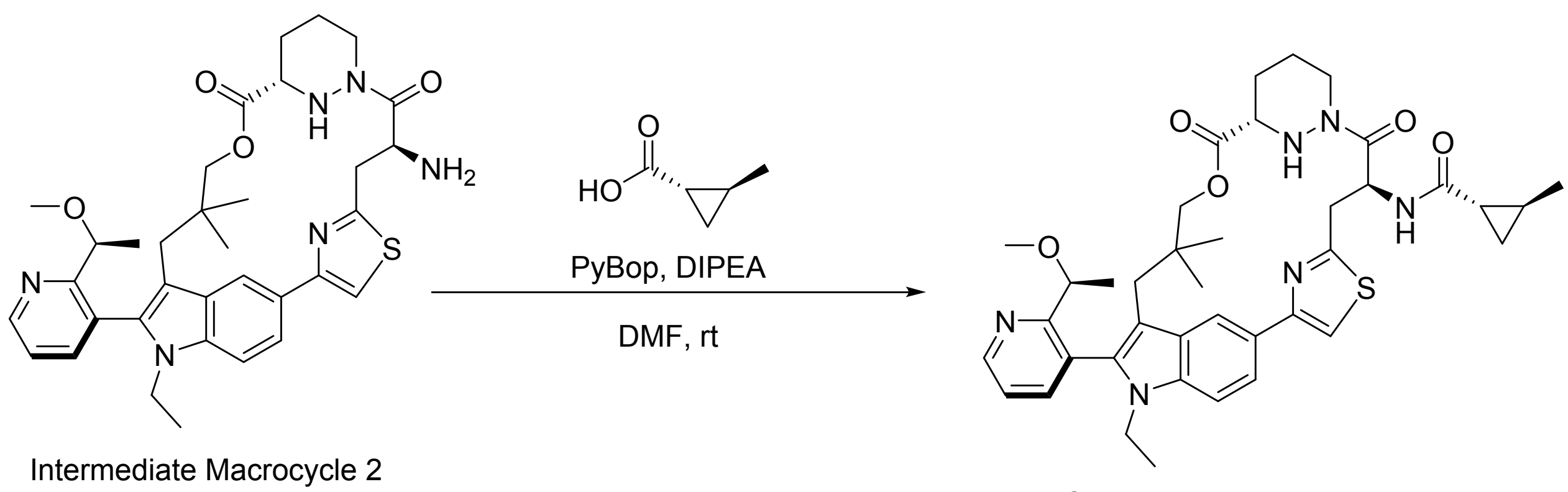

Intermediate Macrocycle 2

Compound 10

(1S,2S)-N-((6^{3}S,4S,Z)-1^1-Ethyl-1^2-(2-((S)-1-methoxyethyl)pyridin-3-yl)-10,10-dimethyl-5,7-dioxo-6^1,6^2,6^3,6^4,6^5,6^6-hexahydro-1^{1}H-8-oxa-2(4,2)-thiazola-1(5,3)-indola-6(1,3)-pyridazinacycloundecaphane-4-yl)-2-methylcyclopropane-1-carboxamide (Compound 10) To a stirred solution of (6^3*S*,4*S*,*Z*)-4-amino-1^1-ethyl-1^2-(2-((*S*)-1-methoxyethyl)pyridin-3-yl)-10,10-dimethyl-6^1,6^2,6^3,6^4,6^5,6^6-hexahydro-1^{1}H-8-oxa-2(4,2)-thiazola-1(5,3)-indola-6(1,3)-pyridazinacycloundecaphane-5,7-dione (200 mg, 0.32 mmol) and DIPEA (409.78 mg, 3.12 mmol) in DMF (5 mL) were added (1*S*,2*S*)-2-methylcyclopropane-1-carboxylic acid (41.27 mg, 0.41 mmol) and PyBop (214.49 mg, 0.41 mmol) in portions at 0 °C. The resulting mixture was stirred for 1 h at room temperature. The reaction was quenched by the addition of H_2O (5 mL) at 0 °C. The resulting mixture was extracted with EtOAc (3 x 20 mL). The combined organic layers were washed with brine (3 x 30 mL), dried over anhydrous Na_2SO_4. After filtration, the filtrate was concentrated under reduced pressure. The residue was purified by reversed-phase flash chromatography with the following conditions (Mobile Phase A: Water (0.05 % $NH_3 \cdot H_2O$), Mobile Phase B: ACN; Flow rate: 60 mL/min; Gradient: 0 % B to 100 % B in 30 min, 65 % B; Wave Length: 254/220 nm) to afford (1S,2S)-N-((6^3*S*,4*S*,*Z*)-1^1-ethyl-1^2-(2-((*S*)-1-methoxyethyl)pyridin-3-yl)-10,10-dimethyl-5,7-dioxo-6^1,6^2,6^3,6^4,6^5,6^6-hexahydro-1^{1}H-8-oxa-2(4,2)-thiazola-1(5,3)-indola-6(1,3)-pyridazinacycloundecaphane-4-yl)-2-methylcyclopropane-1-carboxamide (188 mg, 77.35%) as a solid. ^{1}H NMR (400 MHz, DMSO-d_6) δ 8.79 – 8.70 (m, 1H), 8.58 – 8.45 (m, 2H), 7.86 – 7.69 (m, 3H), 7.64 – 7.45 (m, 2H), 5.57 (t, *J* = 9.0 Hz, 1H), 5.09 (d, *J* = 12.1 Hz, 1H), 4.40 – 4.05 (m, 5H), 3.58 (s, 2H), 3.26 (s, 3H), 3.20 – 3.10 (m, 1H), 2.96 (d, *J* = 14.3 Hz, 1H), 2.82 –2.70 (m, 1H), 2.41 (d, *J* = 14.4 Hz, 1H), 2.12 – 2.04 (m, 1H), 1.83 – 1.71 (m, 2H), 1.59 – 1.44 (m, 2H), 1.37 (d, *J* = 6.0 Hz, 3H), 1.32 – 1.21 (m, 1H), 1.09 – 1.01 (m, 4H), 0.93 – 0.80

(m, 7H), 0.59 – 0.51 (m, 1H), 0.32 (s, 3H). ^{13}C NMR (101 MHz, DMSO-d_6) δ 172.75, 172.31, 166.72, 159.27, 154.60, 150.11, 140.57, 136.93, 135.63, 130.13, 127.19, 126.41, 123.63, 119.73, 117.47, 111.89, 111.48, 111.01, 76.17, 70.63, 57.88, 56.07, 49.34, 38.25, 38.10, 35.71, 32.83, 28.90, 26.12, 24.87, 23.42, 22.54, 18.06, 16.51, 15.12, 15.09, 15.00. HRMS (ESI$^+$, m/z): m/z [M+H] calc'd for $C_{39}H_{49}N_6O_5S$ 713.34797; found 713.34686.

Synthesis of Compound 11

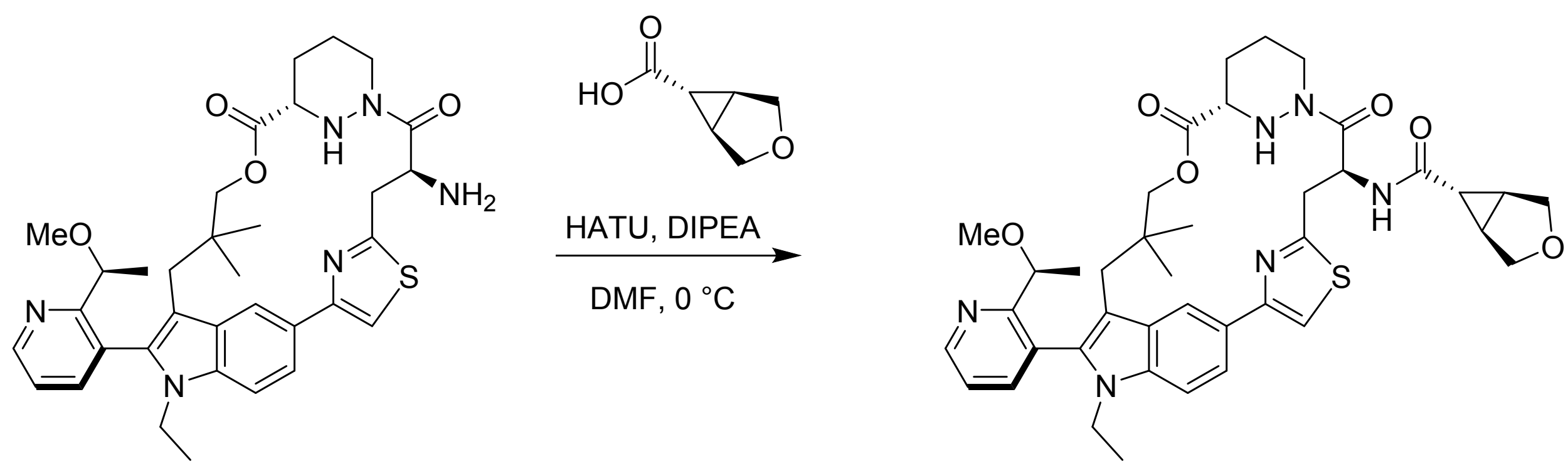

Intermediate Macrocycle 2

Compound 11

(1R,5S,6r)-N-((6^{3}S,4S,Z)-1^1-Ethyl-1^2-(2-((S)-1-methoxyethyl)pyridin-3-yl)-10,10-dimethyl-5,7-dioxo-6^1,6^2,6^3,6^4,6^5,6^6-hexahydro-1^{1}H-8-oxa-2(4,2)-thiazola-1(5,3)-indola-6(1,3)-pyridazinacycloundecaphane-4-yl)-3-oxabicyclo[3.1.0]hexane-6-carboxamide (Compound 11) To a stirred solution of (6^3*S*,4*S*,*Z*)-4-amino-1^1-ethyl-1^2-(2-((*S*)-1-methoxyethyl)pyridin-3-yl)-10,10-dimethyl-6^1,6^2,6^3,6^4,6^5,6^6-hexahydro-1^1*H*-8-oxa-2(4,2)-thiazola-1(5,3)-indola-6(1,3)-pyridazinacycloundecaphane-5,7-dione (200 mg, 0.32 mmol) and DIPEA (122.93 mg, 0.95 mmol) in DMF (5 mL) were added (1*R*,5*S*,6*R*)-3-oxabicyclo[3.1.0]hexane-6-carboxylic acid (60.93 mg, 0.48 mmol) and HATU (180.83 mg, 0.48 mmol) in portions at 0 °C under an air atmosphere. The resulting mixture was stirred for 1 h at room temperature. The reaction was quenched by the addition of H_2O (8 mL) at 0 °C. The resulting mixture was extracted with EtOAc (2 x 5 mL). The combined organic layers were washed with brine (3 x 20 mL), dried over anhydrous Na_2SO_4. After filtration, the filtrate was concentrated under reduced pressure. The residue was purified by reverse phase flash chromatography with the following conditions (Column:C18 Column; Mobile Phase A: Water (0.05% $NH_3{\cdot}H_2O$), Mobile Phase B: ACN; Flow rate: 60 mL/min; Gradient: 0% B to 100% B in 30 min, 61% B; Wave Length: 254/220 nm; RT1(min): 5; Number of Runs: 0) to afford (1R,5S,6r)-N-((6^{3}S,4S,Z)-1^1-ethyl-1^2-(2-((S)-1-methoxyethyl)pyridin-3-yl)-10,10-dimethyl-5,7-dioxo-6^1,6^2,6^3,6^4,6^5,6^6-hexahydro-1^{1}H-8-oxa-2(4,2)-thiazola-1(5,3)-indola-6(1,3)-pyridazinacycloundecaphane-4-yl)-3-oxabicyclo[3.1.0]hexane-6-carboxamide (215 mg, 92% yield) as a solid. ^{1}H NMR (400 MHz, Chloroform-*d*) δ 8.81 (dd, *J* = 4.8, 1.7 Hz, 1H), 8.58 (d, *J* = 1.6 Hz, 1H), 7.66 (dd, *J* = 7.6, 1.7 Hz, 1H), 7.63 – 7.56 (m, 1H), 7.44 – 7.32 (m, 2H), 7.29 (s, 1H), 6.53 (d, *J* = 9.5 Hz, 1H), 6.00 – 5.84 (m, 1H), 4.58

(d, *J* = 12.9 Hz, 1H), 4.39 (q, *J* = 6.0 Hz, 1H), 4.31 – 4.11 (m, 3H), 4.09 – 3.86 (m, 4H), 3.81 – 3.69 (m, 4H), 3.40 (s, 4H), 3.24 – 3.05 (m, 2H), 2.68 (td, *J* = 12.9, 3.0 Hz, 1H), 2.48 (d, *J* = 14.4 Hz, 1H), 2.26 – 2.17 (m, 2H), 2.12 (dt, *J* = 7.3, 3.0 Hz, 1H), 1.95 (dt, *J* = 13.8, 3.2 Hz, 2H), 1.61 – 1.40 (m, 6H), 1.00 – 0.83 (m, 6H), 0.43 (s, 3H). ^{13}C NMR (101 MHz, $CDCl_3$) δ 173.35, 171.94, 171.73, 171.12, 169.60, 165.48, 163.84, 162.57, 160.74, 159.83, 157.80, 155.21, 150.52, 150.14, 140.30, 140.14, 136.17, 135.69, 135.52, 134.78, 130.21, 129.65, 127.10, 126.78, 126.42, 122.76, 121.82, 120.84, 119.57, 119.42, 118.17, 112.34, 112.25, 111.44, 110.50, 109.93, 109.56, 77.48, 77.16, 76.85, 76.03, 75.59, 73.13, 70.98, 69.42, 69.35, 58.52, 57.71, 56.52, 56.42, 49.31, 45.21, 41.72, 39.58, 38.80, 38.21, 37.26, 35.73, 35.37, 32.66, 32.18, 29.69, 29.02, 26.26, 26.18, 26.13, 25.95, 25.08, 24.32, 23.78, 23.73, 23.52, 23.29, 18.50, 16.59, 15.25, 14.79, 0.03. LCMS (ESI): m/z [M+H] calc'd for $C_{40}H_{48}N_6O_6S$ 740; found 741.0.

Synthesis of Common Intermediate Macrocycle 3

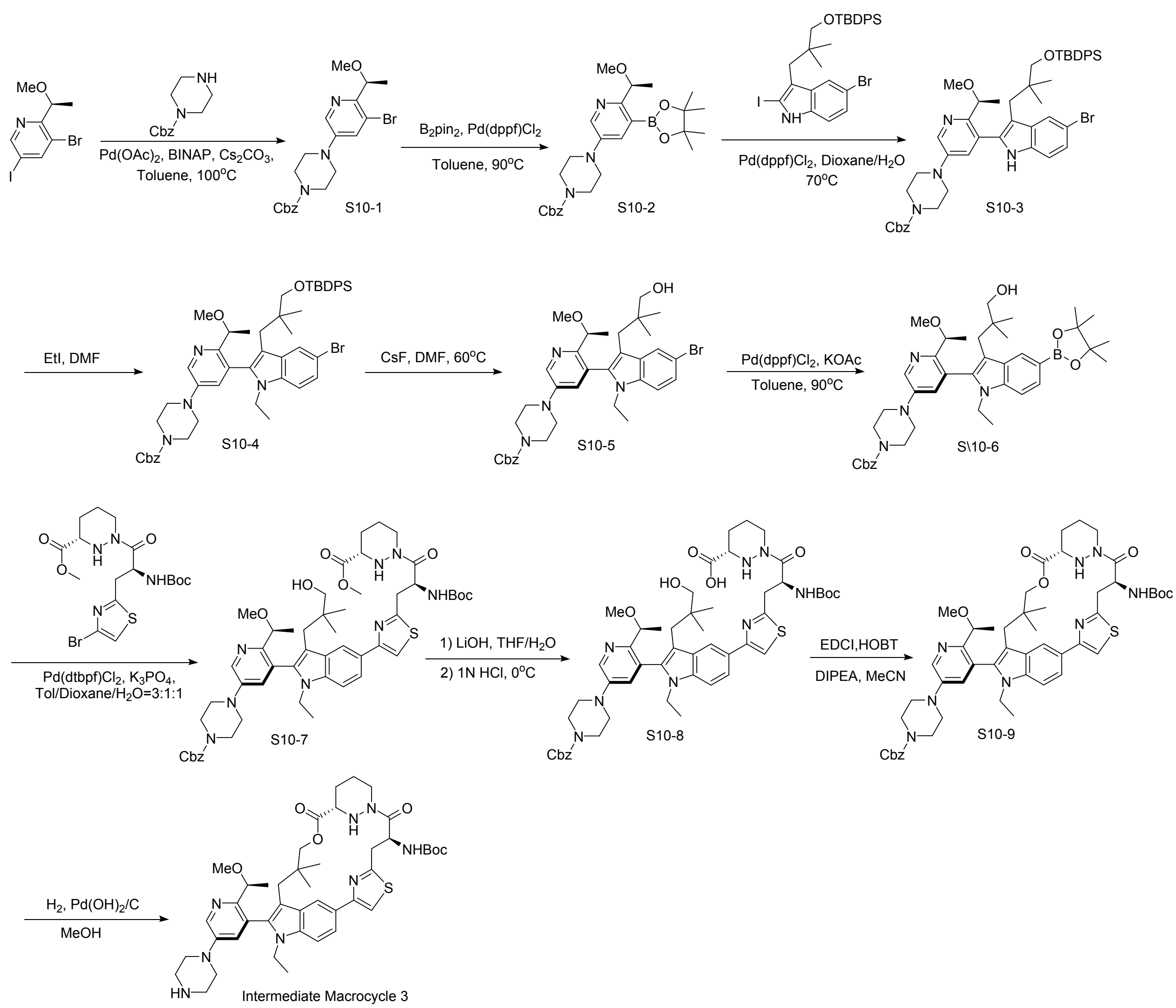

(S)-4-(5-Bromo-6-(1-methoxyethyl)pyridin-3-yl)piperazine-1-carboxylate (S10-1) Into a 3L 3-necked round-bottom flask purged and maintained with an inert atmosphere of argon, was placed 3-bromo-5-iodo-2-[(1*S*)-1-methoxyethyl]pyridine (147 g, 429.8 mmol) benzyl piperazine-1-carboxylate (94.69 g, 429.8 mmol), $Pd(OAc)_2$ (4.83 g, 21.4 mmol), BINAP (5.35 g, 8.6 mmol), Cs_2CO_3 (350.14 g, 1074.6 mmol), toluene (1 L). The resulting solution was stirred overnight at 100 °C in an oil bath. The reaction mixture was cooled to room temperature after completion. The resulting mixture was concentrated under reduced pressure. The residue was purified by silica gel column chromatography with EtOAc/hexane (1:1). Removal of solvent under reduced pressure gave benzyl (*S*)-4-(5-bromo-6-(1-methoxyethyl)pyridin-3-yl)piperazine-1-carboxylate (135 g, 65% yield) as a solid. ^{1}H NMR (400 MHz, DMSO-d_6) δ 8.33 (d, *J* = 2.5 Hz, 1H), 7.60 (d, *J* = 2.5 Hz, 1H), 7.42 – 7.30 (m, 5H), 5.12 (s, 2H), 4.75 (q, *J* = 6.4 Hz, 1H), 3.55 (t, *J* = 4.8 Hz, 4H), 3.29 (t, *J* = 5.2 Hz, 4H), 1.37 (d, *J* = 6.4 Hz, 3H). ^{13}C NMR (101 MHz, Chloroform-*d*) δ 155.03, 147.07, 146.23, 136.34, 133.11, 128.66, 128.55, 128.18, 127.91, 120.82, 76.30, 67.43, 57.02, 47.27, 43.03, 20.05. LCMS (ESI): m/z [M+H] calc'd for $C_{20}H_{24}BrN_3O_3$ 433.1; found 434.1.

(S)-4-(6-(1-Methoxyethyl)-5-(4,4,5,5-tetramethyl-1,3,2-dioxaborolan-2-yl)pyridin-3-yl)piperazine-1-carboxylate (S10-2) Into a 3-L 3-necked round-bottom flask purged and maintained with an inert atmosphere of argon, was placed benzyl 4-[5-bromo-6-[(1*S*)-1-methoxyethyl]pyridin-3-yl]piperazine-1-carboxylate (135 g, 310.8 mmol), bis(pinacolato)diboron (86.82 g, 341.9 mmol), Pd(dppf)Cl_2 (22.74 g, 31.0 mmol), KOAc (76.26 g, 777.5 mmol), toluene (1 L). The resulting solution was stirred for 2 days at 90 °C in an oil bath. The reaction mixture was cooled to room temperature. The resulting mixture was concentrated under reduced pressure. The residue was applied onto a purified by alumina column with EtOAc/hexane (1:3). Removal of solvent under reduced pressure gave benzyl (*S*)-4-(6-(1-methoxyethyl)-5-(4,4,5,5-tetramethyl-1,3,2-dioxaborolan-2-yl)pyridin-3-yl)piperazine-1-carboxylate (167 g, crude) as a solid. ^{1}H NMR (400 MHz, DMSO-d_6) δ 8.27 (d, *J* = 3.0 Hz, 1H), 7.98 (s, 1H), 7.43 – 7.29 (m, 6H), 5.12 (s, 2H), 4.56 (dq, *J* = 31.3, 6.5 Hz, 1H), 3.55 (q, *J* = 10.2, 7.5 Hz, 4H), 3.17 (q, *J* = 5.2, 3.9 Hz, 5H), 3.09 (s, 2H), 1.38 (dd, *J* = 12.4, 6.5 Hz, 3H), 1.31 (s, 6H). ^{13}C NMR (101 MHz, DMSO-d_6) δ 154.88, 145.10, 138.94, 137.31, 128.88, 128.86, 128.32, 128.07, 128.03, 84.11, 80.26, 79.15, 66.81, 56.31, 56.25, 48.49, 48.30, 25.21, 24.96, 21.34, 20.73. LCMS (ESI): m/z [M+H] calc'd for $C_{26}H_{36}BN_3O_5$ 481.3; found 482.1.

Benzyl (S)-4-(5-(5-bromo-3-(3-((tert-butyldiphenylsilyl)oxy)-2,2-dimethylpropyl)-1H-indol-2-yl)-6-(1-methoxyethyl)pyridin-3-yl)piperazine-1-carboxylate (S10-3) Into a 3-L 3-necked round-bottom flask purged and maintained with an inert atmosphere of argon, was placed (*S*)-4-(6-(1-methoxyethyl)-5-(4,4,5,5-tetramethyl-1,3,2-dioxaborolan-2-yl)pyridin-3-yl)piperazine-1-carboxylate (167 g, 346.9 mmol), 5-bromo-3-[3-[(*tert*-butyldiphenylsilyl)oxy]-2,2-dimethylpropyl]-2-iodo-1H-indole (224.27 g, 346.9

mmol), Pd(dppf)Cl_2 (25.38 g, 34.6 mmol), dioxane (600 mL), H_2O (200 mL), K_3PO_4 (184.09 g, 867.2 mmol), toluene (200 mL). The resulting solution was stirred overnight at 70 °C in an oil bath. The reaction mixture was cooled to room temperature and concentrated under reduced pressure. The residue was purified by flash chromatography with EtOAc/hexane (1:1). Removal of solvent under reduced pressure gave benzyl (*S*)-4-(5-(5-bromo-3-(3-((*tert*-butyldiphenylsilyl)oxy)-2,2-dimethylpropyl)-1*H*-indol-2-yl)-6-(1-methoxyethyl)pyridin-3-yl)piperazine-1-carboxylate (146 g, 48% yield) as a solid. ^{1}H NMR (400 MHz, DMSO-d_6) δ 11.32 (s, 1H), 8.43 (d, *J* = 2.8 Hz, 1H), 7.77 (d, *J* = 1.9 Hz, 1H), 7.57 – 7.47 (m, 4H), 7.46 – 7.29 (m, 12H), 7.25 (d, *J* = 2.9 Hz, 1H), 7.21 (dd, *J* = 8.6, 1.9 Hz, 1H), 5.12 (s, 2H), 4.10 (q, *J* = 6.2 Hz, 1H), 3.52 (t, *J* = 5.2 Hz, 4H), 3.18 (d, *J* = 5.7 Hz, 4H), 2.87 (s, 3H), 2.51 (m, 2H), 1.44 – 1.20 (m, 4H), 0.96 (s, 9H), 0.65 (d, *J* = 7.5 Hz, 6H). ^{13}C NMR (101 MHz, Chloroform-*d*) δ 149.20, 137.38, 136.44, 135.73, 135.71, 134.19, 133.83, 131.64, 129.64, 129.10, 128.63, 128.28, 128.11, 127.67, 125.82, 125.03, 122.67, 112.43, 111.48, 73.22, 67.49, 55.32, 48.28, 39.09, 32.57, 27.12, 24.45, 24.32, 19.51. LCMS (ESI): m/z [M+H] calc'd for $C_{49}H_{57}BrN_4O_4Si$ 872.3; found 873.3.

Benzyl (S)-4-(5-(5-bromo-3-(3-((tert-butyldiphenylsilyl)oxy)-2,2-dimethylpropyl)-1-ethyl-1H-indol-2-yl)-6-(1-methoxyethyl)pyridin-3-yl)piperazine-1-carboxylate (S10-4) To a stirred mixture of benzyl (*S*)-4-(5-(5-bromo-3-(3-((*tert*-butyldiphenylsilyl)oxy)-2,2-dimethylpropyl)-1*H*-indol-2-yl)-6-(1-methoxyethyl)pyridin-3-yl)piperazine-1-carboxylate (146 g, 167.0 mmol) and Cs_2CO_3 (163.28 g, 501.1 mmol) in DMF (1200 mL) was added C_2H_5I (52.11 g, 334.0 mmol) in portions at 0 °C under a nitrogen atmosphere. The final reaction mixture was stirred at room temperature for 12 h. The resulting mixture was diluted with EtOAc (1 L) and washed with brine (3 x 1.5 L). The organic layers were dried over anhydrous Na_2SO_4. After filtration, the filtrate was concentrated under reduced pressure to give benzyl (*S*)-4-(5-(5-bromo-3-(3-((*tert*-butyldiphenylsilyl)oxy)-2,2-dimethylpropyl)-1-ethyl-1*H*-indol-2-yl)-6-(1-methoxyethyl)pyridin-3-yl)piperazine-1-carboxylate (143 g, crude) as a solid that was used directly for next step without further purification. ^{1}H NMR (400 MHz, DMSO-d_6) δ 8.48 (dd, *J* = 4.4, 2.8 Hz, 1H), 7.81 (dd, *J* = 4.2, 1.9 Hz, 1H), 7.59 – 7.46 (m, 5H), 7.45 – 7.34 (m, 10H), 7.33 – 7.22 (m, 3H), 5.13 (s, 2H), 4.12 – 3.63 (m, 3H), 3.52 (s, 4H), 3.42 (t, *J* = 9.2 Hz, 1H), 3.37 (s, 3H), 3.28 – 3.08 (m, 5H), 2.98 (s, 1H), 2.81 (s, 2H), 1.34 (d, *J* = 6.2 Hz, 2H), 1.21 (d, *J* = 6.3 Hz, 1H), 1.14 (t, *J* = 7.1 Hz, 2H), 1.07 (t, *J* = 7.1 Hz, 1H), 0.95 (d, *J* = 9.0 Hz, 9H), 0.77 (s, 1H), 0.69 (d, *J* = 3.9 Hz, 3H), 0.63 (s, 2H). ^{13}C NMR (101 MHz, DMSO-d_6) δ 154.81, 149.77, 145.24, 138.60, 137.24, 137.16, 136.69, 135.58, 135.52, 134.64, 133.48, 130.88, 130.12, 128.81, 128.29, 128.15, 128.08, 128.06, 126.01, 124.13, 122.48, 112.25, 112.12, 110.55, 75.32, 66.83, 56.04, 55.75, 47.87, 27.15, 27.13, 25.17, 25.03, 24.75, 19.37, 19.35, 19.03, 17.91, 15.63, 15.36. LCMS (ESI): m/z [M+H] calc'd for $C_{51}H_{61}BrN_4O_4Si$ 900.4; found 901.4.

Benzyl (S)-4-(5-(5-bromo-1-ethyl-3-(3-hydroxy-2,2-dimethylpropyl)-1H-indol-2-yl)-6-(1-methoxyethyl)pyridin-3-yl)piperazine-1-carboxylate (S10-5) To a stirred mixture of benzyl benzyl (*S*)-4-(5-(5-bromo-3-(3-((*tert*-butyldiphenylsilyl)oxy)-2,2-dimethylpropyl)-1-ethyl-1*H*-indol-2-yl)-6-(1-methoxyethyl)pyridin-3-yl)piperazine-1-carboxylate (143 g, 158.5 mmol) in DMF (1250 mL) was added CsF (72.24 g, 475.5 mmol). Then the reaction mixture was stirred at 60 °C for 2 days under a nitrogen atmosphere. The resulting mixture was diluted with EtOAc (1 L) and washed with brine (3 x 1 L). Then the organic phase was concentrated under reduced pressure. The residue was purified by silica gel column chromatography, eluted with Pet. ether/EtOAc (1:3) to afford two atropisomers of benzyl (*S*)-4-(5-(5-bromo-1-ethyl-3-(3-hydroxy-2,2-dimethylpropyl)-1*H*-indol-2-yl)-6-(1-methoxyethyl)pyridin-3-yl)piperazine-1-carboxylate **A** (38 g, 36% yield, RT = 1.677 min in 3 min LCMS(0.1% FA)) and **B** (34 g, 34% yield, RT = 1.578 min in 3 min LCMS(0.1% FA)) both as solids. ^{1}H NMR (400 MHz, DMSO-d6) δ 8.47 (d, J = 2.8 Hz, 1H), 7.90 (d, J = 1.9 Hz, 1H), 7.55 – 7.42 (m, 2H), 7.41 – 7.24 (m, 6H), 5.12 (s, 2H), 4.11 – 4.01 (m, 1H), 3.86 (m, 1H), 3.56 (d, J = 6.2 Hz, 4H), 3.33 (q, J = 5.0 Hz, 4H), 3.06 (d, J = 10.3 Hz, 1H), 2.98 (d, J = 10.3 Hz, 1H), 2.89 (s, 3H), 2.66 (d, J = 14.0 Hz, 1H), 2.51 (m, 1H), 2.15 (d, J = 14.1 Hz, 1H), 1.37 (d, J = 6.2 Hz, 3H), 1.16 (m, 4H), 0.63 (d, J = 8.3 Hz, 6H). ^{13}C NMR (101 MHz, DMSO-d6) δ 154.89, 147.86, 145.90, 137.25, 136.12, 135.68, 134.69, 130.97, 128.84, 128.29, 128.11, 128.04, 124.36, 122.81, 112.28, 112.17, 111.73, 74.76, 70.20, 66.86, 56.06, 47.66, 38.20, 25.37, 25.18, 18.39, 15.35. LCMS (ESI): m/z [M+H] calc'd for $C_{35}H_{43}BrN_4O_4$ 663.2; found 662.2.

(S)-4-(5-(1-Ethyl-3-(3-hydroxy-2,2-dimethylpropyl)-5-(4,4,5,5-tetramethyl-1,3,2-dioxaborolan-2-yl)-1H-indol-2-yl)-6-(1-methoxyethyl)pyridin-3-yl)piperazine-1-carboxylate (S10-6) Into a 500-mL 3-necked round-bottom flask purged and maintained with an inert atmosphere of nitrogen, was placed benzyl (*S*)-4-(5-(5-bromo-1-ethyl-3-(3-hydroxy-2,2-dimethylpropyl)-1*H*-indol-2-yl)-6-(1-methoxyethyl)pyridin-3-yl)piperazine-1-carboxylate **A** (14 g, 21.1 mmol), bis(pinacolato)diboron (5.89 g, 23.21 mmol), Pd(dppf)Cl_2 (1.54 g, 2.1 mmol), KOAc (5.18 g, 52.7 mmol), toluene (150 mL). The resulting solution was stirred for 5 h at 90 °C in an oil bath. The reaction mixture was cooled to room temperature. The resulting mixture was concentrated under reduced pressure. The residue was purified by silica gel column chromatography, eluted with Pet. ether/EtOAc (1:3) to give benzyl (*S*)-4-(5-(1-ethyl-3-(3-hydroxy-2,2-dimethylpropyl)-5-(4,4,5,5-tetramethyl-1,3,2-dioxaborolan-2-yl)-1*H*-indol-2-yl)-6-(1-methoxyethyl)pyridin-3-yl)piperazine-1-carboxylate (12 g, 76% yield) as a solid. ^{1}H NMR (400 MHz, DMSO-d_6) δ 8.48 (d, *J* = 2.6 Hz, 1H), 8.09 (s, 1H), 7.56 (d, *J* = 8.2 Hz, 1H), 7.49 – 7.21 (m, 7H), 5.10 (s, 2H), 4.48 (m, 1H), 4.08 – 4.03 (m, 1H), 3.87 (m, 1H), 3.55 (d, *J* = 16.8 Hz, 10H), 3.34 – 3.19 (m, 4H), 3.07 (m, 2H), 2.82 (d, *J* = 13.8 Hz, 3H), 2.72 (d, *J* = 13.4 Hz, 1H), 2.26 (t, *J* = 15.0 Hz, 1H), 1.95 (s, 1H), 1.52 – 1.33 (m, 3H), 1.33 – 1.19 (m, 8H), 1.09 (s, 1H), 0.66 (dd, *J* = 30.5, 13.4 Hz, 6H). ^{13}C NMR (101

MHz, DMSO-d_6) δ 154.90, 149.97, 145.37, 138.41, 137.96, 137.24, 135.68, 128.96, 128.84, 128.29, 128.03, 111.81, 83.52, 75.30, 71.24, 66.84, 60.21, 55.74, 48.06, 38.33, 25.36, 25.23, 25.14, 25.07, 21.11, 18.02, 15.37, 15.29, 14.47. LCMS (ESI): m/z [M+H] calc'd for $C_{41}H_{55}BN_4O_6$ 710.4; found 711.3.

(S)-1-((S)-3-(4-(2-(5-(4-((Benzyloxy)carbonyl)piperazin-1-yl)-2-((S)-1-methoxyethyl)pyridin-3-yl)-1-ethyl-3-(3-hydroxy-2,2-dimethylpropyl)-1H-indol-5-yl)thiazol-2-yl)-2-((tert-butoxycarbonyl)amino)propanoyl)hexahydropyridazine-3-carboxylate (S10-7) Into a 250-mL round-bottom flask purged and maintained with an inert atmosphere of argon, was placed benzyl (*S*)-4-(5-(1-ethyl-3-(3-hydroxy-2,2-dimethylpropyl)-5-(4,4,5,5-tetramethyl-1,3,2-dioxaborolan-2-yl)-1*H*-indol-2-yl)-6-(1-methoxyethyl)pyridin-3-yl)piperazine-1-carboxylate (10.8 g, 15.2 mmol), methyl (3*S*)-1-[(2*S*)-3-(4-bromo-1,3-thiazol-2-yl)-2-[(*tert*-butoxycarbonyl)amino]propanoyl]-1,2-diazinane-3-carboxylate (7.98 g, 16.7 mmol), Pd(dtbpf)Cl_2 (0.99 g, 1.52 mmol), K_3PO_4 (8.06 g, 37.9 mmol), toluene (60 mL), dioxane (20 mL), H_2O (20 mL). The resulting solution was stirred for 3 h at 70 °C in an oil bath. The reaction mixture was cooled to room temperature. The resulting solution was extracted with EtOAc (2 x 50 mL) and concentrated under reduced pressure. The residue was applied onto a silica gel column with EtOAc/hexane (10:1). Removal of solvent to give methyl (*S*)-1-((*S*)-3-(4-(2-(5-(4-((benzyloxy)carbonyl)piperazin-1-yl)-2-((*S*)-1-methoxyethyl)pyridin-3-yl)-1-ethyl-3-(3-hydroxy-2,2-dimethylpropyl)-1*H*-indol-5-yl)thiazol-2-yl)-2-((*tert*-butoxycarbonyl)amino)propanoyl)hexahydropyridazine-3-carboxylate (8 g, 51% yield) as a solid. ^1H NMR (400 MHz, DMSO-d_6) δ 8.48 (d, *J* = 2.8 Hz, 1H), 8.22 (d, *J* = 1.6 Hz, 1H), 7.84 – 7.67 (m, 2H), 7.52 (d, *J* = 8.6 Hz, 1H), 7.42 – 7.27 (m, 6H), 6.62 (d, *J* = 8.7 Hz, 1H), 5.46 – 5.24 (m, 2H), 5.12 (s, 2H), 4.45 (t, *J* = 5.1 Hz, 1H), 4.16 – 3.98 (m, 2H), 3.91 (m, 2H), 3.61 (d, *J* = 25.6 Hz, 8H), 3.29 (m, 5H), 3.11 (dd, *J* = 10.2, 5.3 Hz, 1H), 3.02 (dd, *J* = 10.2, 5.2 Hz, 1H), 2.86 (s, 3H), 2.70 (d, *J* = 14.0 Hz, 1H), 2.51 (p, *J* = 1.8 Hz, 1H), 2.26 (d, *J* = 14.0 Hz, 1H), 1.95 – 1.80 (m, 1H), 1.77 – 1.46 (m, 3H), 1.38 (d, *J* = 5.1 Hz, 12H), 1.16 (dd, *J* = 8.4, 5.9 Hz, 4H), 0.66 (d, *J* = 22.3 Hz, 6H). ^{13}C NMR (101 MHz, DMSO-d_6) δ 171.94, 171.33, 166.01, 154.90, 149.90, 145.40, 138.39, 137.28, 136.25, 135.82, 129.48, 128.87, 128.32, 128.07, 127.40, 126.50, 126.30, 120.70, 111.81, 110.23, 78.62, 75.32, 70.98, 66.85, 58.34, 55.76, 52.17, 48.04, 41.31, 38.36, 28.65, 25.27, 25.21, 18.03, 15.45. LCMS (ESI): m/z [M+H] calc'd for $C_{52}H_{68}N_8O_9S$ 980.5; found 980.9.

(S)-1-((S)-3-(4-(2-(5-(4-((Benzyloxy)carbonyl)piperazin-1-yl)-2-((S)-1-methoxyethyl)pyridin-3-yl)-1-ethyl-3-(3-hydroxy-2,2-dimethylpropyl)-1H-indol-5-yl)thiazol-2-yl)-2-((tert-butoxycarbonyl)amino)propanoyl)hexahydropyridazine-3-carboxylic acid (S10-8) To a stirred mixture of methyl (*S*)-1-((*S*)-3-(4-(2-(5-(4-((benzyloxy)carbonyl)piperazin-1-yl)-2-((S)-1-methoxyethyl)pyridin-3-yl)-1-ethyl-3-(3-hydroxy-2,2-dimethylpropyl)-1*H*-indol-5-yl)thiazol-2-yl)-2-((*tert*-

butoxycarbonyl)amino)propanoyl)hexahydropyridazine-3-carboxylate (12 g, 12.23 mmol) in THF (100 mL)/H_2O (100 mL) was added LiOH (2.45 g, 61.1 mmol) under a nitrogen atmosphere and the resulting mixture was stirred for 2 h at room temperature. THF was concentrated under reduced pressure. The pH of aqueous phase was acidified to 5 with HCL (1N) at 0 °C. The aqueous layer was extracted with DCM (3 x 100ml). The organic phase was concentrated under reduced pressure to give (*S*)-1-((*S*)-3-(4-(2-(5-(4-((benzyloxy)carbonyl)piperazin-1-yl)-2-((*S*)-1-methoxyethyl)pyridin-3-yl)-1-ethyl-3-(3-hydroxy-2,2-dimethylpropyl)-1*H*-indol-5-yl)thiazol-2-yl)-2-((*tert*-butoxycarbonyl)amino)propanoyl)hexahydropyridazine-3-carboxylic acid (10 g, 85% yield) as a solid. ^{1}H NMR (400 MHz, DMSO-d_6) δ 8.47 (d, J = 2.7 Hz, 1H), 8.29 (s, 1H), 7.75 (d, J = 8.7 Hz, 2H), 7.59 – 7.18 (m, 8H), 5.23 (s, 1H), 5.11 (s, 2H), 4.63 (d, J = 11.4 Hz, 1H), 4.20 (d, J = 11.4 Hz, 1H), 4.06 (m, 3H), 3.89 (m, 2H), 3.62 – 3.44 (m, 12H), 3.12 (d, J = 10.1 Hz, 1H), 3.02 (d, J = 10.3 Hz, 2H), 2.86 (d, J = 6.7 Hz, 3H), 2.70 (d, J = 16.6 Hz, 2H), 2.25 (d, J = 13.8 Hz, 1H), 1.96 (s, 1H), 1.71 (s, 2H), 1.28 – 0.96 (m, 11H), 0.65 (d, J = 21.1 Hz, 9H). ^{13}C NMR (101 MHz, DMSO-d_6) δ 165.66, 155.92, 155.24, 154.92, 149.87, 145.37, 137.23, 136.13, 135.75, 128.89, 128.35, 128.05, 111.91, 78.80, 75.28, 70.86, 66.84, 55.79, 47.99, 28.63, 25.31, 25.22, 18.09, 15.45. LCMS (ESI): m/z [M+H] calc'd for $C_{51}H_{66}N_8O_9S$ 966.5; found 967.0.

Benzyl 4-(5-((6^{3}S,4S,Z)-4-((tert-butoxycarbonyl)amino)-1^1-ethyl-10,10-dimethyl-5,7-dioxo-6^1,6^2,6^3,6^4,6^5,6^6-hexahydro-1^{1}H-8-oxa-2(4,2)-thiazola-1(5,3)-indola-6(1,3)-pyridazinacycloundecaphane-1^2-yl)-6-((S)-1-methoxyethyl)pyridin-3-yl)piperazine-1-carboxylate (S10-9) Into a 3-L round-bottom flask purged and maintained with an inert atmosphere of nitrogen, was placed (*S*)-1-((*S*)-3-(4-(2-(5-(4-((benzyloxy)carbonyl)piperazin-1-yl)-2-((*S*)-1-methoxyethyl)pyridin-3-yl)-1-ethyl-3-(3-hydroxy-2,2-dimethylpropyl)-1*H*-indol-5-yl)thiazol-2-yl)-2-((*tert*-butoxycarbonyl)amino)propanoyl)hexahydropyridazine-3-carboxylic acid (18 g, 18.61 mmol), MeCN (1.8 L), DIPEA (96.21 g, 744.4 mmol), EDCI (107.03 g, 558.3 mmol), HOBT (25.15 g, 186.1 mmol). The resulting solution was stirred overnight at 25 °C. The resulting mixture was concentrated under reduced pressure after the reaction was complete. The resulting solution was diluted with DCM (1 L). The resulting mixture was washed with HCl (3 x 1 L, 1N). The resulting mixture was washed with H_2O (3 x 1 L). Then the organic layer was concentrated, the residue was applied onto a silica gel column with EtOAc/hexane (1:1). Removal of solvent under reduced pressure gave benzyl 4-(5-((6^3*S*,4*S*,*Z*)-4-((*tert*-butoxycarbonyl)amino)-1^1-ethyl-10,10-dimethyl-5,7-dioxo-6^1,6^2,6^3,6^4,6^5,6^6-hexahydro-1^1*H*-8-oxa-2(4,2)-thiazola-1(5,3)-indola-6(1,3)-pyridazinacycloundecaphane-1^2-yl)-6-((*S*)-1-methoxyethyl)pyridin-3-yl)piperazine-1-carboxylate (10.4 g, 55% yield) as a solid. ^{1}H NMR (400 MHz, DMSO-d_6) δ 8.50 (d, J = 1.7 Hz, 1H), 8.47 (d, J = 2.8 Hz, 1H), 7.80 (s, 1H), 7.74 (dd, J = 8.6, 1.6 Hz, 1H), 7.55 (d, J = 8.7 Hz, 1H), 7.41 – 7.35 (m, 4H), 7.35 – 7.22 (m, 3H), 5.24 (t, J = 8.7 Hz, 1H), 5.11 (s, 2H), 5.06 (d, J = 12.2 Hz,

1H), 4.22 (m, 5H), 3.56 (t, *J* = 7.9 Hz, 6H), 3.29 (t, *J* = 5.3 Hz, 4H), 3.22 (s, 3H), 2.95 (d, *J* = 14.3 Hz, 1H), 2.77 (m, 1H), 2.50 (m, 1H), 2.44 (d, *J* = 14.4 Hz, 1H), 2.08 (t, *J* = 9.2 Hz, 1H), 1.80 (d, *J* = 9.0 Hz, 2H), 1.40 (s, 9H), 1.34 (d, *J* = 6.2 Hz, 4H), 1.08 (s, 1H), 0.95 – 0.84 (m, 6H), 0.35 (s, 3H). ^{13}C NMR (101 MHz, DMSO-d_6) δ 172.32, 167.04, 155.84, 154.90, 154.62, 149.67, 145.94, 137.44, 137.27, 135.52, 130.20, 128.86, 128.31, 128.06, 127.22, 126.38, 111.60, 78.50, 66.84, 55.82, 47.93, 35.69, 28.70, 26.25, 24.89, 16.49, 15.33. LCMS (ESI): m/z [M+H] calc'd for $C_{51}H_{64}N_8O_8S$ 948.5; found 949.3.

tert-Butyl ((6^{3}S,4S,Z)-11-ethyl-1^2-(2-((S)-1-methoxyethyl)-5-(piperazin-1-yl)pyridin-3-yl)-10,10-dimethyl-5,7-dioxo-6^1,6^2,6^3,6^4,6^5,6^6-hexahydro-1^{1}H-8-oxa-2(4,2)-thiazola-1(5,3)-indola-6(1,3)-pyridazinacycloundecaphane-4-yl)carbamate (Intermediate Macrocycle 4) Into a 250-mL round-bottom flask purged and maintained with an inert atmosphere of nitrogen, was placed benzyl 4-(5-((6^3*S*,4*S*,*Z*)-4-((*tert*-butoxycarbonyl)amino)-1^1-ethyl-10,10-dimethyl-5,7-dioxo-6^1,6^2,6^3,6^4,6^5,6^6-hexahydro-1^1*H*-8-oxa-2(4,2)-thiazola-1(5,3)-indola-6(1,3)-pyridazinacycloundecaphane-1^2-yl)-6-((*S*)-1-methoxyethyl)pyridin-3-yl)piperazine-1-carboxylate (10.40 g, 10.9 mmol), $Pd(OH)_2$/C (5 g, 46.9 mmol), MeOH (100 mL). The resulting solution was stirred for 3 h at room temperature under a 2 atm H_2 atmosphere. The solids were filtered out and the filter cake was washed with MeOH (3 x 100 mL). Then combined organic phase was concentrated under reduced pressure to give *tert*-butyl ((6^3*S*,4*S*,*Z*)-1*1*-ethyl-1^2-(2-((*S*)-1-methoxyethyl)-5-(piperazin-1-yl)pyridin-3-yl)-10,10-dimethyl-5,7-dioxo-6^1,6^2,6^3,6^4,6^5,6^6-hexahydro-1^1*H*-8-oxa-2(4,2)-thiazola-1(5,3)-indola-6(1,3)-pyridazinacycloundecaphane-4-yl)carbamate (8.5 g, 90% yield) as a solid. ^{1}H NMR (400 MHz, DMSO-d_6) δ 8.54 (d, *J* = 1.7 Hz, 1H), 8.49 (dd, *J* = 8.8, 2.9 Hz, 1H), 7.84 (s, 1H), 7.77 (dd, *J* = 8.6, 1.6 Hz, 1H), 7.59 (d, *J* = 8.7 Hz, 1H), 7.33 (d, *J* = 9.2 Hz, 1H), 7.21 (d, *J* = 2.9 Hz, 1H), 5.79 (s, 1H), 5.28 (t, *J* = 9.0 Hz, 1H), 5.10 (d, *J* = 12.2 Hz, 1H), 4.44 – 4.05 (m, 6H), 3.63 (t, *J* = 7.7 Hz, 3H), 3.49 – 3.07 (m, 13H), 3.06 – 2.94 (m, 2H), 2.89 (dd, *J* = 6.3, 4.0 Hz, 3H), 2.81 (t, *J* = 13.7 Hz, 1H), 2.66 (s, 1H), 2.54 (m, 1H), 2.49 (d, *J* = 14.4 Hz, 1H), 2.13 (d, *J* = 14.4 Hz, 1H), 1.83 (s, 2H), 1.66 – 1.50 (m, 1H), 1.44 (s, 8H), 1.38 (d, *J* = 6.1 Hz, 4H), 1.12 (s, 1H), 0.95 (d, *J* = 7.1 Hz, 6H), 0.40 (s, 3H). ^{13}C NMR (101 MHz, DMSO-d_6) δ 173.07, 172.31, 167.02, 155.84, 154.63, 146.59, 137.62, 135.50, 130.22, 127.12, 124.89, 119.57, 111.51, 111.27, 78.47, 55.78, 55.33, 48.82, 45.53, 35.68, 28.72, 26.27, 24.90, 16.50, 15.34. LCMS (ESI): m/z [M+H] calc'd for $C_{43}H_{58}N_8O_6S$ 814.4; found 815.3.

Synthesis of Compound 12

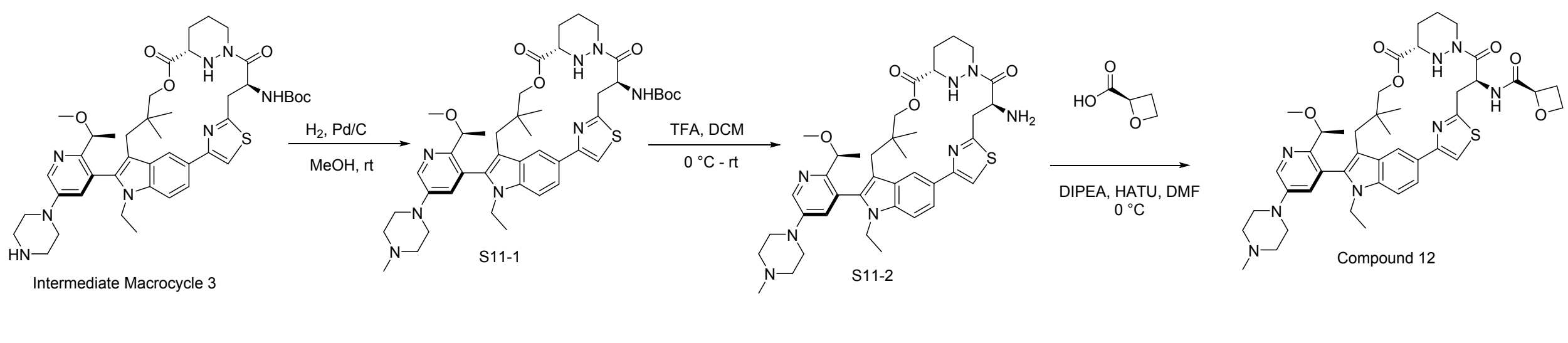

tert-Butyl ((6^{3}S,4S,Z)-1^1-ethyl-1^2-(2-((S)-1-methoxyethyl)-5-(4-methylpiperazin-1-yl)pyridin-3-yl)-10,10-dimethyl-5,7-dioxo-6^1,6^2,6^3,6^4,6^5,6^6-hexahydro-1^{1}H-8-oxa-2(4,2)-thiazola-1(5,3)-indola-6(1,3)-pyridazinacycloundecaphane-4-yl)carbamate (S11-1) A solution of *tert*-butyl ((6^3*S*,4*S*,*Z*)-1*1*-ethyl-1^2-(2-((*S*)-1-methoxyethyl)-5-(piperazin-1-yl)pyridin-3-yl)-10,10-dimethyl-5,7-dioxo-6^1,6^2,6^3,6^4,6^5,6^6-hexahydro-1^1*H*-8-oxa-2(4,2)-thiazola-1(5,3)-indola-6(1,3)-pyridazinacycloundecaphane-4-yl)carbamate (1.5 g, 1.58 mmol), $Pd(OH)_2/C$ (1.5 g) and Paraformaldehyde (208.85 mg, 4.74 mmol) in MeOH (15 mL) was stirred for 2 h at room temperature under a hydrogen atmosphere. The resulting mixture was filtered, the filter cake was washed with MeOH (3 x 50 mL). The filtrate was concentrated under reduced pressure. The residue was purified by reversed-phase flash chromatography with the following conditions: column, C18 silica gel; mobile phase, MeCN in water (10 mmol/L NH_4HCO_3), 10 % to 70 % gradient in 30 min; detector, UV 254 nm. This resulted in tert-butyl ((6^{3}S,4S,Z)-1^1-ethyl-1^2-(2-((S)-1-methoxyethyl)-5-(4-methylpiperazin-1-yl)pyridin-3-yl)-10,10-dimethyl-5,7-dioxo-6^1,6^2,6^3,6^4,6^5,6^6-hexahydro-1^{1}H-8-oxa-2(4,2)-thiazola-1(5,3)-indola-6(1,3)-pyridazinacycloundecaphane-4-yl)carbamate (800 mg, 58% yield) as a yellow solid. ^{1}H NMR (400 MHz, DMSO-d_6) δ 8.37 – 8.15 (m, 2H), 7.61 – 7.43 (m, 2H), 7.31 (d, *J* = 8.6 Hz, 1H), 7.12 – 6.90 (m, 2H), 5.17 – 4.96 (m, 1H), 4.85 (d, *J* = 11.8 Hz, 1H), 4.22 – 3.86 (m, 5H), 3.39 (q, *J* = 11.7 Hz, 3H), 3.16 – 2.89 (m, 9H), 2.77 (d, *J* = 14.1 Hz, 1H), 2.57 (d, *J* = 17.4 Hz, 1H), 2.36 – 2.09 (m, 5H), 1.98 (s, 3H), 1.91 – 1.81 (m, 1H), 1.66 – 1.44 (m, 2H), 1.17 (d, *J* = 17.7 Hz, 12H), 0.70 (d, *J* = 8.5 Hz, 6H), 0.16 (s, 3H). ^{13}C NMR (101 MHz, DMSO-d_6) δ 173.04, 172.29, 167.00, 155.81, 154.63, 149.06, 146.09, 137.59, 135.50, 130.21, 127.12, 126.35, 111.52, 78.45, 60.19, 55.76, 54.64, 47.87, 46.24, 35.68, 28.71, 26.26, 24.89, 16.49, 15.34, 14.53. LCMS (ESI): m/z [M+H] calc'd for $C_{44}H_{60}N_8O_6S$ 828.4; found 829.3.

(6^{3}S,4S,Z)-4-Amino-1^1-ethyl-1^2-(2-((S)-1-methoxyethyl)-5-(4-methylpiperazin-1-yl)pyridin-3-yl)-10,10-dimethyl-6^1,6^2,6^3,6^4,6^5,6^6-hexahydro-1^{1}H-8-oxa-2(4,2)-thiazola-1(5,3)-indola-6(1,3)-pyridazinacycloundecaphane-5,7-dione (S11-2) A solution of *tert*-butyl ((6^3*S*,4*S*,*Z*)-1^1-ethyl-1^2-(2-((*S*)-1-methoxyethyl)-5-(4-methylpiperazin-1-yl)pyridin-3-yl)-10,10-dimethyl-5,7-dioxo-6^1,6^2,6^3,6^4,6^5,6^6-hexahydro-1^1*H*-8-oxa-2(4,2)-thiazola-1(5,3)-indola-6(1,3)-pyridazinacycloundecaphane-4-yl)carbamate (800 mg, 0.97 mmol) in DCM (10 mL) was treated with TFA (2 mL) at 0 °C. After 3 min, the resulting solution was warmed to room temperature and then stirred for 2 h. The mixture was then basified to pH 7 with saturated $NaHCO_3$ (aq.). The resulting mixture was extracted with DCM (3 x 50 mL), dried over anhydrous Na_2SO_4. After filtration, the filtrate was concentrated under reduced pressure to afford (6^3*S*,4*S*,*Z*)-4-amino-1^1-ethyl-1^2-(2-((*S*)-1-methoxyethyl)-5-(4-methylpiperazin-1-yl)pyridin-3-yl)-10,10-dimethyl-6^1,6^2,6^3,6^4,6^5,6^6-hexahydro-1^1*H*-8-oxa-2(4,2)-thiazola-1(5,3)-indola-6(1,3)-pyridazinacycloundecaphane-5,7-dione (680 mg, crude) as a solid. ^{1}H NMR (400 MHz, DMSO-d_6) δ 8.48

(dd, *J* = 18.2, 2.2 Hz, 2H), 7.81 – 7.65 (m, 2H), 7.54 (d, *J* = 8.6 Hz, 1H), 7.23 (d, *J* = 2.9 Hz, 1H), 4.93 (d, *J* = 12.1 Hz, 1H), 4.38 (d, *J* = 8.5 Hz, 1H), 4.29 (p, *J* = 7.3, 5.9 Hz, 2H), 4.21 – 4.09 (m, 3H), 4.03 (q, *J* = 7.1 Hz, 1H), 3.60 (q, *J* = 10.9 Hz, 2H), 3.21 (s, 3H), 2.97 (d, *J* = 14.5 Hz, 1H), 2.75 (m, 2H), 2.54 – 2.37 (m, 6H), 2.21 (s, 3H), 2.08 (d, *J* = 12.1 Hz, 1H), 1.99 (s, 2H), 1.88 – 1.64 (m, 2H), 1.51 (m, 1H), 1.35 (d, *J* = 6.1 Hz, 3H), 1.23 (s, 1H), 1.18 (t, *J* = 7.1 Hz, 2H), 0.97 – 0.85 (m, 6H), 0.36 (s, 3H). ^{13}C NMR (101 MHz, DMSO-d_6) δ 176.35, 172.38, 168.21, 154.46, 148.98, 146.08, 137.48, 135.44, 130.17, 127.17, 126.50, 125.00, 119.62, 117.54, 111.54, 110.90, 110.75, 75.73, 70.80, 57.85, 55.73, 54.62, 51.37, 47.83, 46.23, 35.73, 26.15, 24.91, 16.50, 15.35. LCMS (ESI): m/z [M+H] calc'd for $C_{39}H_{52}N_8O_4S$ 728; found 729.

(2R)-N-((6^{3}S,4S,Z)-1^1-Ethyl-1^2-(2-((S)-1-methoxyethyl)-5-(4-methylpiperazin-1-yl)pyridin-3-yl)-10,10-dimethyl-5,7-dioxo-6^1,6^2,6^3,6^4,6^5,6^6-hexahydro-11H-8-oxa-2(4,2)-thiazola-1(5,3)-indola-6(1,3)-pyridazinacycloundecaphane-4-yl)oxetane-2-carboxamide (Compound 12) A solution of (6^3*S*,4*S*,*Z*)-4-amino-1^1-ethyl-1^2-(2-((*S*)-1-methoxyethyl)-5-(4-methylpiperazin-1-yl)pyridin-3-yl)-10,10-dimethyl-6^1,6^2,6^3,6^4,6^5,6^6-hexahydro-1^1*H*-8-oxa-2(4,2)-thiazola-1(5,3)-indola-6(1,3)-pyridazinacycloundecaphane-5,7-dione (680 mg, 1.00 mmol) in DMF (8 mL) was treated with DIPEA (1294.31 mg, 10.01 mmol) 2*R*)-oxetane-2-carboxylic acid (204.47 mg, 2.00 mmol) and HATU (761.55 mg, 2.00 mmol) for 1 h at 0 °C. The reaction was quenched by the addition of H_2O (10 mL) at 0 °C. The resulting mixture was extracted with EtOAc (3 x 20 mL). The combined organic layers were washed with brine (3 x 10 mL), dried over anhydrous Na_2SO_4. After filtration, the filtrate was concentrated under reduced pressure. The residue was purified by reversed-phase flash chromatography with the following conditions: column, C18 silica gel; mobile phase, MeCN in water (10 mmol/L NH_4HCO_3), 10 % to 80 % gradient in 30 min; detector, UV 254 nm to afford (2R)-N-((6^{3}S,4S,Z)-1^1-ethyl-1^2-(2-((S)-1-methoxyethyl)-5-(4-methylpiperazin-1-yl)pyridin-3-yl)-10,10-dimethyl-5,7-dioxo-6^1,6^2,6^3,6^4,6^5,6^6-hexahydro-11H-8-oxa-2(4,2)-thiazola-1(5,3)-indola-6(1,3)-pyridazinacycloundecaphane-4-yl)oxetane-2-carboxamide (250 mg, 29% yield) as a yellow solid. ^{1}H NMR (400 MHz, DMSO-d_6) δ 8.49 (d, *J* = 1.6 Hz, 1H), 8.45 (d, *J* = 2.9 Hz, 1H), 8.31 (d, *J* = 9.3 Hz, 1H), 7.79 (s, 1H), 7.73 (dd, *J* = 8.6, 1.6 Hz, 1H), 7.55 (d, *J* = 8.6 Hz, 1H), 7.22 (d, *J* = 2.9 Hz, 1H), 5.59 (t, *J* = 9.0 Hz, 1H), 5.17 (d, *J* = 12.1 Hz, 1H), 5.03 (dd, *J* = 8.9, 6.5 Hz, 1H), 4.62 (ddt, *J* = 20.5, 8.9, 6.0 Hz, 2H), 4.34 – 4.09 (m, 5H), 3.63 – 3.52 (m, 2H), 3.46 – 3.35 (m, 2H), 3.26 (d, *J* = 5.2 Hz, 4H), 3.21 (s, 3H), 2.98 – 2.88 (m, 2H), 2.86 – 2.72 (m, 1H), 2.45 (dd, *J* = 10.2, 5.5 Hz, 6H), 2.22 (s, 3H), 2.10 (d, *J* = 12.1 Hz, 1H), 1.82 (s, 2H), 1.53 (q, *J* = 10.1, 8.0 Hz, 1H), 1.33 (d, *J* = 6.1 Hz, 4H), 1.24 (s, 1H), 0.95 – 0.83 (m, 6H). ^{13}C NMR (101 MHz, $CDCl_3$) δ 172.82, 172.75, 171.96, 171.11, 169.56, 165.20, 163.27, 157.94, 155.13, 150.25, 145.87, 145.17, 139.35, 138.54, 136.79, 135.66, 135.38, 130.18, 126.96, 126.30, 125.92, 125.37, 120.74, 119.36, 118.00, 111.93, 111.82, 110.48, 109.88, 109.46, 79.39, 79.12, 77.44,

77.13, 76.81, 75.53, 75.06, 73.17, 71.05, 70.03, 69.75, 60.37, 58.62, 57.47, 56.30, 56.24, 54.59, 53.47, 48.80, 48.56, 48.30, 46.11, 45.21, 41.81, 39.96, 38.85, 38.21, 37.25, 35.72, 32.76, 31.91, 29.68, 29.10, 26.48, 26.06, 25.39, 24.97, 24.46, 23.65, 23.28, 22.68, 21.04, 18.52, 16.45, 15.36, 15.01, 14.20, 14.13, 0.01. HRMS (ESI$^+$, m/z): [M+H] calc'd for $C_{43}H_{57}N_8O_6S$ 813.4116; found 813.4093.

Synthesis of Compound 13 (RMC-6236)

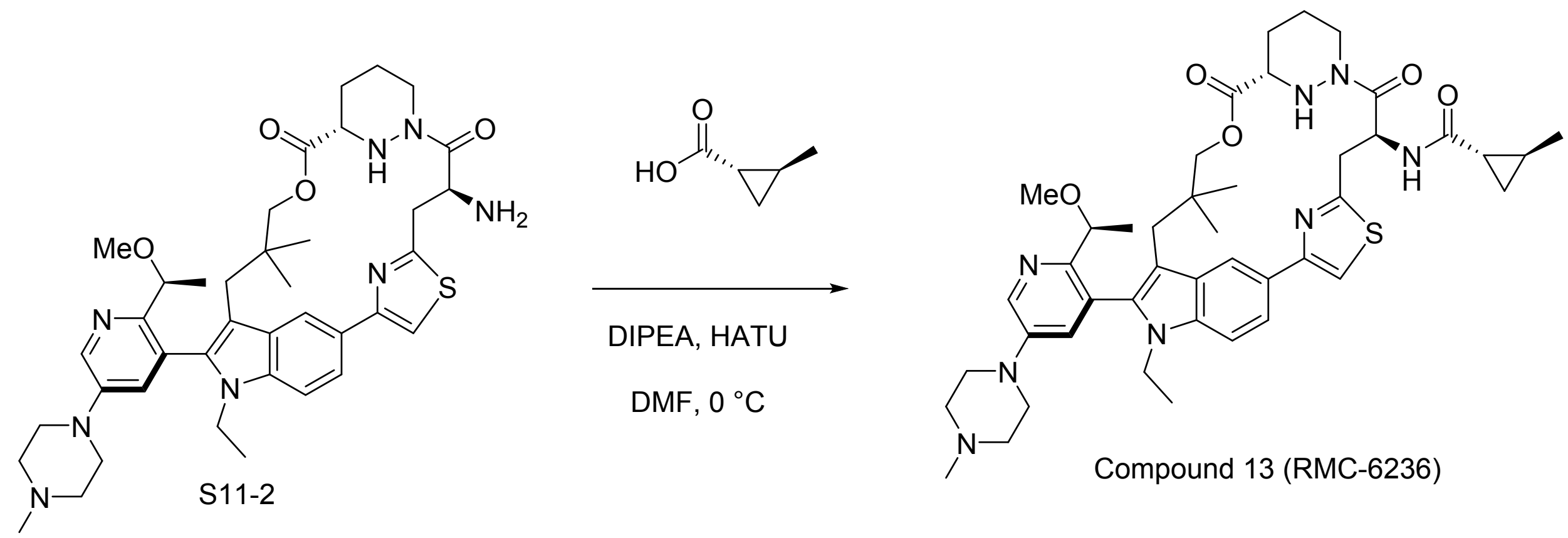

(1S,2S)-N-((6^{3}S,4S,Z)-1^1-Ethyl-1^2-(2-((S)-1-methoxyethyl)-5-(4-methylpiperazin-1-yl)pyridin-3-yl)-10,10-dimethyl-5,7-dioxo-6^1,6^2,6^3,6^4,6^5,6^6-hexahydro-1^{1}H-8-oxa-2(4,2)-thiazola-1(5,3)-indola-6(1,3)-pyridazinacycloundecaphane-4-yl)-2-methylcyclopropane-1-carboxamide (Compound 13 ***RMC-6236****)* To a stirred solution of (6^3*S*,4*S*,*Z*)-4-amino-1^1-ethyl-1^2-(2-((*S*)-1-methoxyethyl)-5-(4-methylpiperazin-1-yl)pyridin-3-yl)-10,10-dimethyl-6^1,6^2,6^3,6^4,6^5,6^6-hexahydro-1^1*H*-8-oxa-2(4,2)-thiazola-1(5,3)-indola-6(1,3)-pyridazinacycloundecaphane-5,7-dione (400 mg, 0.55 mmol) and (1S,2S)-2-methylcyclopropane-1-carboxylic acid (65.92 mg, 0.66 mmol) in DMF (4 mL) was added DIPEA (283.7 mg, 2.2 mmol) and HATU (229.51 mg, 0.60 mmol) in portions at 0 °C under a nitrogen atmosphere. The resulting mixture was stirred for 40 min at 0 °C then the reaction was quenched by the addition of sat. NH_4Cl (aq.) (30 mL). The resulting mixture was extracted with EtOAc (3 x 10 mL). The combined organic layers were washed with brine (2 x 50 mL) and dried over anhydrous Na_2SO_4. After filtration, the filtrate was concentrated under reduced pressure. The resulting crude product was purified by Prep-HPLC with the following conditions (Column: C18 spherical, 20-35 um; 100 A, 80 g; Mobile Phase A:Water(10 MMOL/L NH_4HCO_3), Mobile Phase B:ACN; Flow rate:60 mL/min; Gradient:30 B to 70 B in 30 min, 254 nm; RT1:15.2) to afford (1*S*,2*S*)-*N*-((6^3*S*,4*S*,*Z*)-1^1-ethyl-1^2-(2-((*S*)-1-methoxyethyl)-5-(4-methylpiperazin-1-yl)pyridin-3-yl)-10,10-dimethyl-5,7-dioxo-6^1,6^2,6^3,6^4,6^5,6^6-hexahydro-1^1*H*-8-oxa-2(4,2)-thiazola-1(5,3)-indola-6(1,3)-pyridazinacycloundecaphane-4-yl)-2-methylcyclopropane-1-carboxamide (102 mg, 96.4%

purity, 22% yield) as a white solid. ^{1}H NMR (400 MHz, DMSO-d6) δ 8.26 (d, J = 22.0 Hz, 3H), 7.50 (d, J = 18.5 Hz, 2H), 7.29 (d, J = 8.5 Hz, 1H), 6.98 (s, 1H), 5.37 (t, J = 9.2 Hz, 1H), 4.84 (d, J = 11.7 Hz, 1H), 4.16 – 3.84 (m, 5H), 3.15 (s, 2H), 3.01 (d, J = 18.9 Hz, 9H), 2.74 (d, J = 14.0 Hz, 1H), 2.23 (d, J = 20.8 Hz, 5H), 1.96 (s, 3H), 1.86 (s, 1H), 1.55 (s, 2H), 1.29 (s, 2H), 1.21 – 1.01 (m, 3H), 0.84 (s, 4H), 0.68 (s, 8H), 0.31 (s, 1H), 0.14 (s, 3H). 13C NMR (100 MHz, DMSO) δ 172.78, 172.32, 166.74, 154.72, 149.04, 146.09, 137.56, 135.49, 130.18, 127.12, 126.32, 125.00, 119.59, 117.45, 111.51, 111.36, 110.86, 75.72, 70.73, 57.89, 55.81, 54.65, 49.42, 47.87, 46.28, 38.24, 35.69, 33.00, 28.94, 26.23, 24.92, 23.47, 22.58, 21.16, 18.08, 16.54, 15.37, 15.09. HRMS (ESI): m/z [M+H] calc'd for $C_{44}H_{58}N_8O_5S$ 811.43073.; found 811.43236.

Synthesis of Compound 14

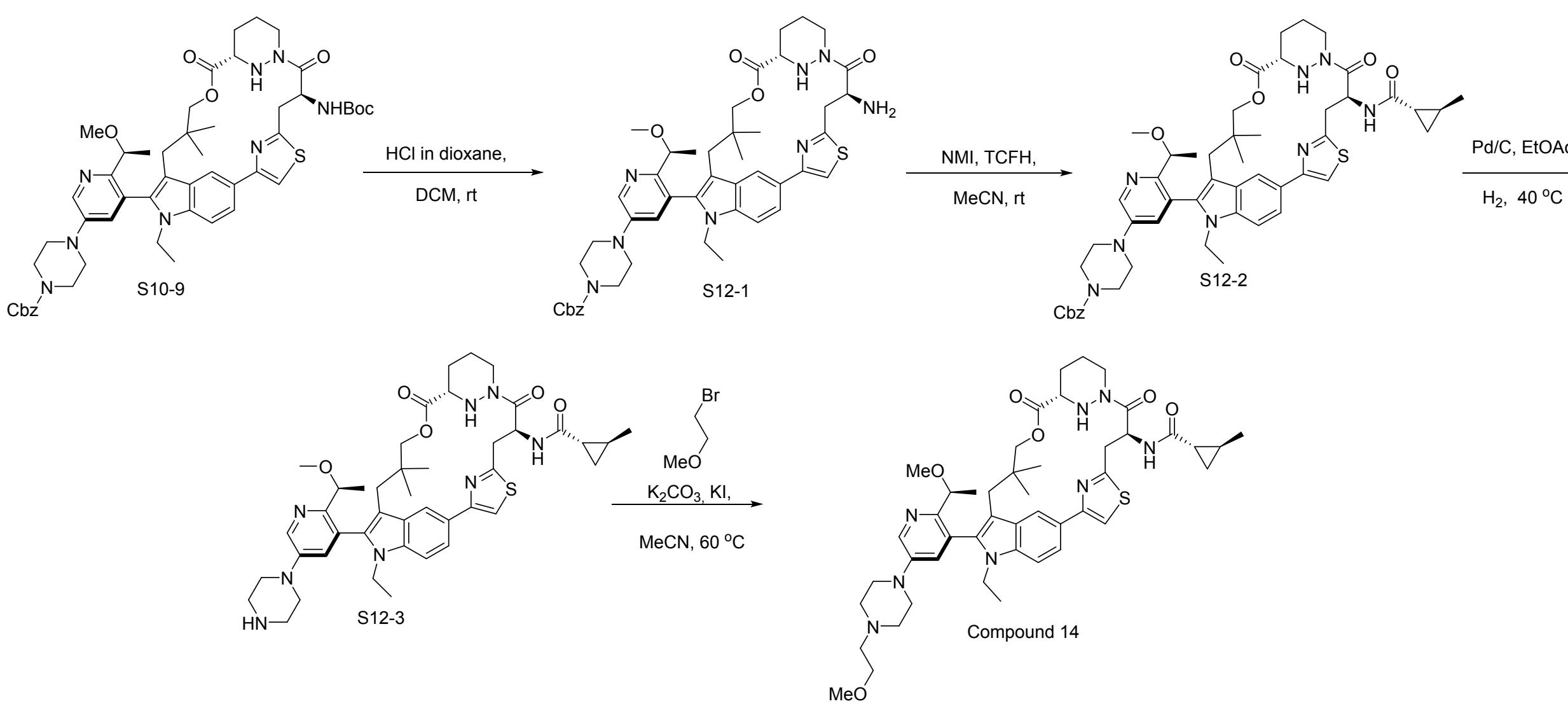

Benzyl 4-(5-((6^{3}S,4S,Z)-4-amino-1^1-ethyl-10,10-dimethyl-5,7-dioxo-6^1,6^2,6^3,6^4,6^5,6^6-hexahydro-1^{1}H-8-oxa-2(4,2)-thiazola-1(5,3)-indola-6(1,3)-pyridazinacycloundecaphane-12-yl)-6-((S)-1-methoxyethyl)pyridin-3-yl)piperazine-1-carboxylate (S12-1) To a stirred solution of benzyl 4-(5-((6^3*S*,4*S*,*Z*)-4-((*tert*-butoxycarbonyl)amino)-1^1-ethyl-10,10-dimethyl-5,7-dioxo-6^1,6^2,6^3,6^4,6^5,6^6-hexahydro-1^1*H*-8-oxa-2(4,2)-thiazola-1(5,3)-indola-6(1,3)-pyridazinacycloundecaphane-12-yl)-6-((*S*)-1-methoxyethyl)pyridin-3-yl)piperazine-1-carboxylate (500 mg, 0.53 mmol) in DCM (3 mL) were added hydrochloric titrant (1 mL) dropwise at 0 °C under a nitrogen atmosphere. The resulting mixture was stirred at room temperature for 1h. The resulting mixture was concentrated under reduced pressure. This resulted in benzyl 4-(5-((6^3*S*,4*S*,*Z*)-4-amino-1^1-ethyl-10,10-dimethyl-5,7-dioxo-6^1,6^2,6^3,6^4,6^5,6^6-hexahydro-1^1*H*-8-oxa-2(4,2)-thiazola-1(5,3)-indola-6(1,3)-pyridazinacycloundecaphane-12-yl)-6-((*S*)-1-methoxyethyl)pyridin-3-yl)piperazine-1-carboxylate (500 mg crude) as a solid. The crude product was used in the next step directly without further purification. ^{1}H NMR (400 MHz, DMSO-d_6) δ 8.47 (dd, *J* =

6.6, 2.2 Hz, 2H), 7.77 (s, 1H), 7.73 (dd, *J* = 8.6, 1.6 Hz, 1H), 7.55 (d, *J* = 8.7 Hz, 1H), 7.38 (d, *J* = 4.4 Hz, 4H), 7.33 (dt, *J* = 8.6, 4.0 Hz, 1H), 7.27 (d, *J* = 2.9 Hz, 1H), 5.11 (s, 2H), 4.94 (d, *J* = 12.2 Hz, 1H), 4.36 (d, *J* = 8.6 Hz, 1H), 4.34 – 4.23 (m, 2H), 4.20 – 4.05 (m, 3H), 3.62 – 3.53 (m, 6H), 3.31 – 3.25 (m, 6H), 3.21 (s, 4H), 2.95 (d, *J* = 14.4 Hz, 1H), 2.79 – 2.68 (m, 2H), 2.43 (d, *J* = 14.3 Hz, 1H), 2.10 – 2.05 (m, 1H), 1.79 (s, 2H), 1.57 – 1.45 (m, 1H), 1.34 (d, *J* = 6.1 Hz, 3H), 0.96 – 0.84 (m, 6H), 0.35 (s, 3H). ^{13}C NMR (101 MHz, DMSO-d_6) δ 176.29, 172.44, 168.25, 154.89, 154.36, 149.50, 145.93, 137.95, 137.31, 137.28, 135.42, 130.11, 128.91, 128.36, 128.07, 127.22, 126.45, 125.65, 119.59, 117.42, 111.55, 111.03, 110.85, 75.70, 70.78, 66.81, 57.81, 55.83, 55.37, 51.32, 47.86, 42.17, 38.17, 35.73, 32.92, 28.98, 26.13, 24.89, 23.48, 16.53, 15.37. HRMS (ESI$^+$, m/z): [M+H] calc'd for $C_{46}H_{57}N_8O_6S$ 849.41163; found 849.41077.

Benzyl 4-(5-((6^{3}S,4S,Z)-1^1-ethyl-10,10-dimethyl-4-((1S,2S)-2-methylcyclopropane-1-carboxamido)-5,7-dioxo-61,6^2,6^3,6^4,6^5,6^6-hexahydro-1^{1}H-8-oxa-2(4,2)-thiazola-1(5,3)-indola-6(1,3)-pyridazinacycloundecaphane-12-yl)-6-((S)-1-methoxyethyl)pyridin-3-yl)piperazine-1-carboxylate (S12-2)

To a stirred solution of benzyl 4-(5-((6^3*S*,4*S*,*Z*)-4-amino-1^1-ethyl-10,10-dimethyl-5,7-dioxo-6^1,6^2,6^3,6^4,6^5,6^6-hexahydro-1^1*H*-8-oxa-2(4,2)-thiazola-1(5,3)-indola-6(1,3)-pyridazinacycloundecaphane-12-yl)-6-((*S*)-1-methoxyethyl)pyridin-3-yl)piperazine-1-carboxylate (500 mg, 0.59 mmol) and 1-methyl-1*H*-imidazole (241.76 mg, 2.95 mmol) in MeCN (5 mL) were added (1*S*,2*S*)-2-methylcyclopropane-1-carboxylic acid (88.44 mg, 0.88 mmol) and TCFH (330.46 mg, 1.18 mmol) in portions at room temperature under a nitrogen atmosphere. The resulting mixture was stirred for 1 h. The resulting mixture was extracted with EtOAc (1 x 50mL). The combined organic layers were washed with brine (3 x 30 mL), dried over anhydrous Na_2SO_4. After filtration, the filtrate was concentrated under reduced pressure. The residue was purified by silica gel column chromatography, eluted with Pet. ether / EtOAc (1:1) to afford benzyl 4-(5-((6^3*S*,4*S*,*Z*)-1^1-ethyl-10,10-dimethyl-4-((1*S*,2*S*)-2-methylcyclopropane-1-carboxamido)-5,7-dioxo-61,6^2,6^3,6^4,6^5,6^6-hexahydro-1^1*H*-8-oxa-2(4,2)-thiazola-1(5,3)-indola-6(1,3)-pyridazinacycloundecaphane-12-yl)-6-((*S*)-1-methoxyethyl)pyridin-3-yl)piperazine-1-carboxylate (410 mg, 75% yield) as a solid. ^{1}H NMR (400 MHz, DMSO-d_6) δ 8.70 – 8.44 (m, 3H), 7.79 (s, 1H), 7.73 (dd, *J* = 8.8, 1.7 Hz, 1H), 7.55 (d, *J* = 8.6 Hz, 1H), 7.37 (d, *J* = 4.5 Hz, 4H), 7.33 (dt, *J* = 8.9, 4.4 Hz, 1H), 7.27 (d, *J* = 2.8 Hz, 1H), 5.57 (t, *J* = 9.2 Hz, 1H), 5.11 (s, 2H), 4.36 – 4.10 (m, 5H), 4.03 (q, *J* = 7.1 Hz, 2H), 3.57 (s, 6H), 3.35 (d, *J* = 4.2 Hz, 2H), 3.22 (s, 2H), 3.19 – 3.08 (m, 1H), 2.95 (d, *J* = 14.3 Hz, 1H), 2.76 (s, 1H), 2.69 (d, *J* = 1.2 Hz, 1H), 2.44 (d, *J* = 14.3 Hz, 1H), 2.08 (d, *J* = 11.5 Hz, 1H), 1.99 (s, 2H), 1.79 (s, 2H), 1.59 – 1.46 (m, 2H), 1.34 (d, *J* = 6.1 Hz, 3H), 1.17 (dd, *J* = 7.7, 6.5 Hz, 2H), 1.07 (s, 3H), 0.94 – 0.86 (m, 6H), 0.65 – 0.50 (m, 1H), 0.35 (s, 3H). ^{13}C NMR (101 MHz, DMSO-d_6) δ 172.74, 172.35, 170.80, 166.73, 154.87, 154.66, 149.58, 145.93, 137.99, 137.40, 137.29, 135.47, 130.13, 127.18, 128.90, 128.35, 128.07, 126.29, 125.64, 119.58, 117.38, 111.54, 111.40, 110.91, 75.70, 70.70, 66.81, 60.23,

57.84, 55.87, 55.38, 49.34, 47.88, 38.71, 35.68, 32.96, 28.90, 26.19, 24.89, 23.39, 22.53, 21.23, 18.05, 16.52, 15.37, 14.55. HRMS (ESI$^+$, m/z): [M+H] calc'd for $C_{51}H_{63}N_8O_7S$ 931.45349; found 931.45233.

1S,2S)-N-((6^{3}S,4S,Z)-1^1-Ethyl-12-(2-((S)-1-methoxyethyl)-5-(piperazin-1-yl)pyridin-3-yl)-10,10-dimethyl-5,7-dioxo-6^1,6^2,6^3,6^4,6^5,6^6-hexahydro-1^{1}H-8-oxa-2(4,2)-thiazola-1(5,3)-indola-6(1,3)-pyridazinacycloundecaphane-4-yl)-2-methylcyclopropane-1-carboxamide (S12-3) To a stirred solution of benzyl 4-(5-((6^3*S*,4*S*,*Z*)-1^1-ethyl-10,10-dimethyl-4-((1*S*,2*S*)-2-methylcyclopropane-1-carboxamido)-5,7-dioxo-61,6^2,6^3,6^4,6^5,6^6-hexahydro-1^1*H*-8-oxa-2(4,2)-thiazola-1(5,3)-indola-6(1,3)-pyridazinacycloundecaphane-12-yl)-6-((*S*)-1-methoxyethyl)pyridin-3-yl)piperazine-1-carboxylate (410 mg, 0.44 mmol) in EtOAc (5 mL) were added Pd/C (410 mg, 3.85 mmol) in portions at room temperature under a nitrogen atmosphere. The resulting mixture was stirred at 40 °C for 16 h under a hydrogen atmosphere. The resulting mixture was filtered, the filter cake was washed with EtOAc (3 x 50 mL). The filtrate was concentrated under reduced pressure. The crude product was used in the next step directly without further purification. This resulted in (1*S*,2*S*)-*N*-((6^3*S*,4*S*,*Z*)-1^1-ethyl-12-(2-((*S*)-1-methoxyethyl)-5-(piperazin-1-yl)pyridin-3-yl)-10,10-dimethyl-5,7-dioxo-6^1,6^2,6^3,6^4,6^5,6^6-hexahydro-1^1*H*-8-oxa-2(4,2)-thiazola-1(5,3)-indola-6(1,3)-pyridazinacycloundecaphane-4-yl)-2-methylcyclopropane-1-carboxamide (400 mg crude) as a solid. ^{1}H NMR (300 MHz, DMSO-d_6) δ 8.58 – 8.40 (m, 3H), 7.79 (s, 1H), 7.72 (dd, *J* = 8.7, 1.6 Hz, 1H), 7.55 (d, *J* = 8.6 Hz, 1H), 7.18 (d, *J* = 2.9 Hz, 1H), 5.56 (t, *J* = 9.0 Hz, 1H), 5.16 – 4.99 (m, 1H), 4.38 – 4.09 (m, 5H), 4.03 (q, *J* = 7.1 Hz, 2H), 3.57 (s, 3H), 3.44 (d, *J* = 7.0 Hz, 4H), 2.98 – 2.83 (m, 4H), 2.75 (d, *J* = 8.4 Hz, 1H), 2.44 (d, *J* = 14.1 Hz, 1H), 2.08 (d, *J* = 12.2 Hz, 1H), 1.99 (s, 2H), 1.79 (s, 2H), 1.49 (d, *J* = 9.0 Hz, 2H), 1.33 (d, *J* = 6.1 Hz, 3H), 1.17 (t, *J* = 7.1 Hz, 3H), 1.07 (d, *J* = 1.9 Hz, 4H), 0.90 (q, *J* = 7.2, 5.6 Hz, 6H), 0.62 – 0.48 (m, 1H), 0.35 (s, 3H). ^{13}C NMR (101 MHz, DMSO) δ 172.73, 172.35, 170.81, 166.72, 154.66, 148.91, 146.62, 137.58, 135.46, 130.13, 128.07, 127.07, 126.26, 124.84, 119.53, 117.37, 111.46, 111.39, 110.90, 75.72, 70.70, 60.23, 55.84, 49.34, 48.87, 45.57, 38.19, 38.14, 35.67, 32.96, 28.89, 26.21, 24.88, 23.39, 22.52, 21.24, 18.05, 16.55, 15.37, 15.08, 14.56. HRMS (ESI$^+$, m/z): [M+H] calc'd for $C_{43}H_{57}N_8O_5S$ 797.41671; found 797.41455.

(1S,2S)-N-((63S,4S,Z)-1^1-Ethyl-12-(2-((S)-1-methoxyethyl)-5-(4-(2-methoxyethyl)piperazin-1-yl)pyridin-3-yl)-10,10-dimethyl-5,7-dioxo-6^1,6^2,6^3,6^4,6^5,6^6-hexahydro-1^{1}H-8-oxa-2(4,2)-thiazola-1(5,3)-indola-6(1,3)-pyridazinacycloundecaphane-4-yl)-2-methylcyclopropane-1-carboxamide (Compound 14) To a stirred solution of (1*S*,2*S*)-*N*-((6^3*S*,4*S*,*Z*)-1^1-ethyl-12-(2-((*S*)-1-methoxyethyl)-5-(piperazin-1-yl)pyridin-3-yl)-10,10-dimethyl-5,7-dioxo-6^1,6^2,6^3,6^4,6^5,6^6-hexahydro-1^1*H*-8-oxa-2(4,2)-thiazola-1(5,3)-indola-6(1,3)-pyridazinacycloundecaphane-4-yl)-2-methylcyclopropane-1-carboxamide (100 mg, 0.125 mmol) and 2-bromoethyl methyl ether (22.67 mg, 0.163 mmol) in MeCN (1 mL) were added KI (20.83 mg, 0.125 mmol) and K_2CO_3 (17.34 mg, 0.125 mmol) in portions at room temperature under a nitrogen atmosphere.

The resulting mixture was stirred at 60 °C for 4 h. The resulting mixture was extracted with EtOAc (1 x 50mL). The combined organic layers were washed with brine (3 x 30 mL), dried over anhydrous Na_2SO_4. After filtration, the filtrate was concentrated under reduced pressure. The residue was purified by silica gel column chromatography, eluted with Pet. ether / EtOAc (1:2) to afford (1*S*,2*S*)-*N*-((6^3*S*,4*S*,*Z*)-1^1-ethyl-1^2-(2-((*S*)-1-methoxyethyl)-5-(4-(2-methoxyethyl)piperazin-1-yl)pyridin-3-yl)-10,10-dimethyl-5,7-dioxo-6^1,6^2,6^3,6^4,6^5,6^6-hexahydro-1^1*H*-8-oxa-2(4,2)-thiazola-1(5,3)-indola-6(1,3)-pyridazinacycloundecaphane-4-yl)-2-methylcyclopropane-1-carboxamide (50 mg, 47%yield) as a white solid. ^{1}H NMR (400 MHz, DMSO-d_6) δ 8.60 – 8.45 (m, 3H), 7.80 (s, 1H), 7.74 (dd, J = 8.7, 1.7 Hz, 1H), 7.57 (d, J = 8.7 Hz, 1H), 7.44 (d, J = 2.9 Hz, 1H), 5.56 (t, J = 9.1 Hz, 1H), 5.09 (d, J = 12.2 Hz, 1H), 4.35 – 4.11 (m, 6H), 4.01 (d, J = 12.5 Hz, 2H), 3.74 – 3.67 (m, 2H), 3.64 – 3.51 (m, 4H), 3.45 – 3.36 (m, 2H), 3.34 (s, 3H), 3.32 – 3.06 (m, 9H), 2.94 (d, J = 14.2 Hz, 1H), 2.81 – 2.72 (m, 1H), 2.46 – 2.40 (m, 1H), 2.07 (d, J = 12.0 Hz, 1H), 1.77 (d, J = 14.5 Hz, 2H), 1.59 – 1.45 (m, 2H), 1.34 (d, J = 6.2 Hz, 3H), 1.14 – 1.00 (m, 4H), 0.95 – 0.84 (m, 6H), 0.59 – 0.50 (m, 1H), 0.35 (s, 3H). ^{13}C NMR (101 MHz, DMSO) δ 172.78, 172.34, 170.82, 166.71, 154.66, 149.00, 146.14, 137.60, 137.54, 135.46, 130.13, 127.07, 126.25, 124.98, 119.54, 117.38, 111.49, 111.38, 110.89, 70.70, 70.43, 60.23, 58.47, 57.49, 55.83, 55.35, 53.11, 49.06, 47.99, 35.66, 31.76, 29.49, 28.88, 26.18, 24.86, 22.52, 21.21, 18.03, 16.52, 15.34, 15.08, 14.98, 14.53. HRMS (ESI$^+$, m/z): [M+H] calc'd for $C_{46}H_{63}N_8O_6S$ 855.45858; found 855.45575.

Synthesis of Compound 15

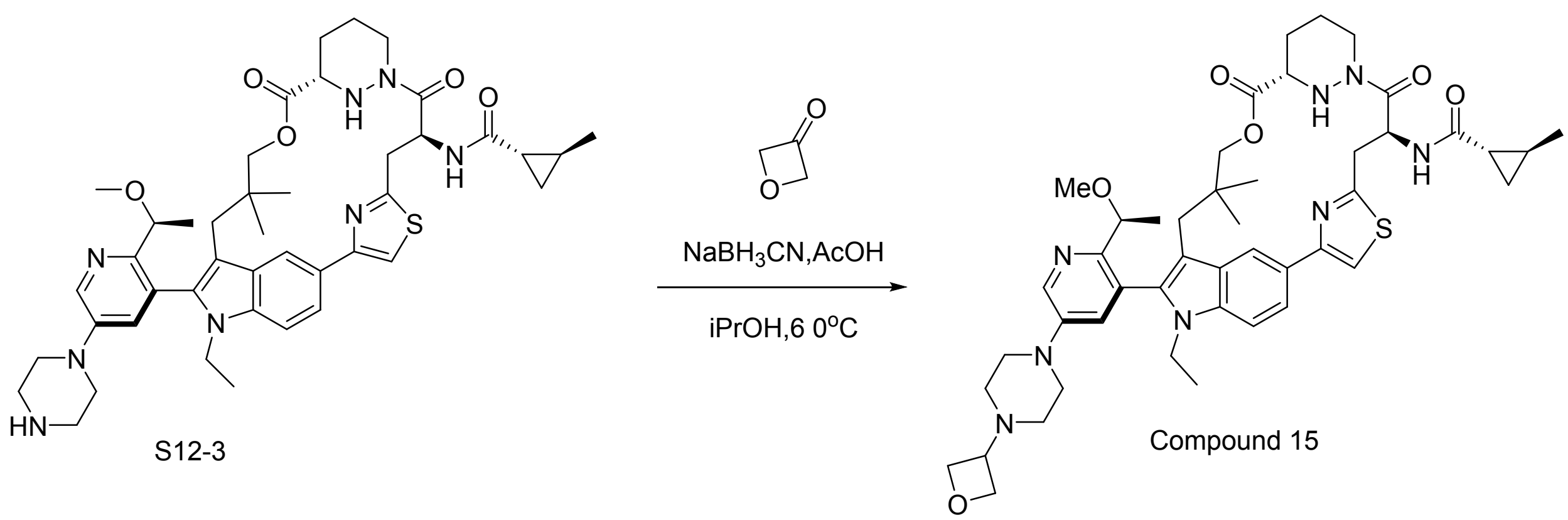

(1S,2S)-N-((6^{3}S,4S,Z)-1^1-Ethyl-1^2-(2-((S)-1-methoxyethyl)-5-(4-(oxetan-3-yl)piperazin-1-yl)pyridin-3-yl)-10,10-dimethyl-5,7-dioxo-6^1,6^2,6^3,6^4,6^5,6^6-hexahydro-1^{1}H-8-oxa-2(4,2)-thiazola-1(5,3)-indola-6(1,3)-pyridazinacycloundecaphane-4-yl)-2-methylcyclopropane-1-carboxamide (Compound 15) To a stirred solution of (1*S*,2*S*)-*N*-((6^3*S*,4*S*,*Z*)-1^1-ethyl-12-(2-((*S*)-1-methoxyethyl)-5-(piperazin-1-yl)pyridin-3-yl)-10,10-dimethyl-5,7-dioxo-6^1,6^2,6^3,6^4,6^5,6^6-hexahydro-1^1*H*-8-oxa-2(4,2)-thiazola-1(5,3)-indola-6(1,3)-pyridazinacycloundecaphane-4-yl)-2-methylcyclopropane-1-carboxamide (150 mg, 0.19 mmol) and 3-oxetanone (271.24 mg, 3.76 mmol) in iPrOH (1.5 mL) were added AcOH (56.51 mg, 0.94 mmol) and

sodium cyanoborohydride (35.48 mg, 0.56 mmol) in portions at 0 °C under a nitrogen atmosphere. The resulting mixture was stirred at 60°C for 1 h. The reaction was quenched with sat. NH_4Cl (aq.) at 0 °C. The resulting mixture was extracted with EtOAc (1 x 50mL). The combined organic layers were washed with brine (3 x 30 mL), dried over anhydrous Na_2SO_4. After filtration, the filtrate was concentrated under reduced pressure. The residue was purified by silica gel column chromatography, eluted with Pet. ether / EtOAc (1:2) to afford (1*S*,2*S*)-*N*-((6^3*S*,4*S*,*Z*)-1^1-ethyl-1^2-(2-((*S*)-1-methoxyethyl)-5-(4-(oxetan-3-yl)piperazin-1-yl)pyridin-3-yl)-10,10-dimethyl-5,7-dioxo-6^1,6^2,6^3,6^4,6^5,6^6-hexahydro-1^1*H*-8-oxa-2(4,2)-thiazola-1(5,3)-indola-6(1,3)-pyridazinacycloundecaphane-4-yl)-2-methylcyclopropane-1-carboxamide (60 mg, 38%yield) as a solid. ^{1}H NMR (400 MHz, DMSO-d_6) δ 8.58 – 8.42 (m, 3H), 7.79 (s, 1H), 7.73 (dd, *J* = 8.7, 1.6 Hz, 1H), 7.55 (d, *J* = 8.6 Hz, 1H), 7.24 (d, *J* = 2.9 Hz, 1H), 5.57 (t, *J* = 9.1 Hz, 1H), 5.16 – 5.03 (m, 1H), 4.57 (t, *J* = 6.5 Hz, 2H), 4.46 (t, *J* = 6.1 Hz, 2H), 4.35 – 4.24 (m, 2H), 4.23 – 4.09 (m, 3H), 4.03 (q, *J* = 7.1 Hz, 1H), 3.58 (s, 2H), 3.50 – 3.40 (m, 1H), 3.21 (s, 3H), 3.19 – 3.11 (m, 1H), 2.94 (d, *J* = 14.3 Hz, 1H), 2.83 – 2.71 (m, 1H), 2.54 – 2.48 (m, 2H), 2.46 – 2.37 (m, 4H), 2.13 – 2.05 (m, 2H), 1.99 (s, 2H), 1.87 – 1.71 (m, 2H), 1.57 – 1.46 (m, 2H), 1.33 (d, *J* = 6.0 Hz, 3H), 1.17 (t, *J* = 7.1 Hz, 2H), 1.07 (s, 3H), 0.95 – 0.86 (m, 6H), 0.60 – 0.51 (m, 1H), 0.35 (s, 3H). ^{13}C NMR (101 MHz, DMSO-d_6) δ 170.61, 170.23, 168.69, 164.59, 152.54, 147.02, 143.96, 135.40, 133.34, 128.01, 126.78, 124.98, 124.14, 123.02, 117.43, 115.24, 109.38, 109.28, 108.79, 72.68, 68.58, 58.11, 56.77, 55.73, 53.74, 47.22, 46.95, 45.59, 36.02, 33.55, 30.84, 26.77, 24.08, 22.76, 21.27, 20.40, 19.11, 15.93, 14.42, 13.25, 12.96, 12.44. HRMS (ESI$^+$, m/z): [M+H] calc'd for $C_{46}H_{61}N_8O_6S$ 853.44293; found 853.44055.

Synthesis of Compound 16

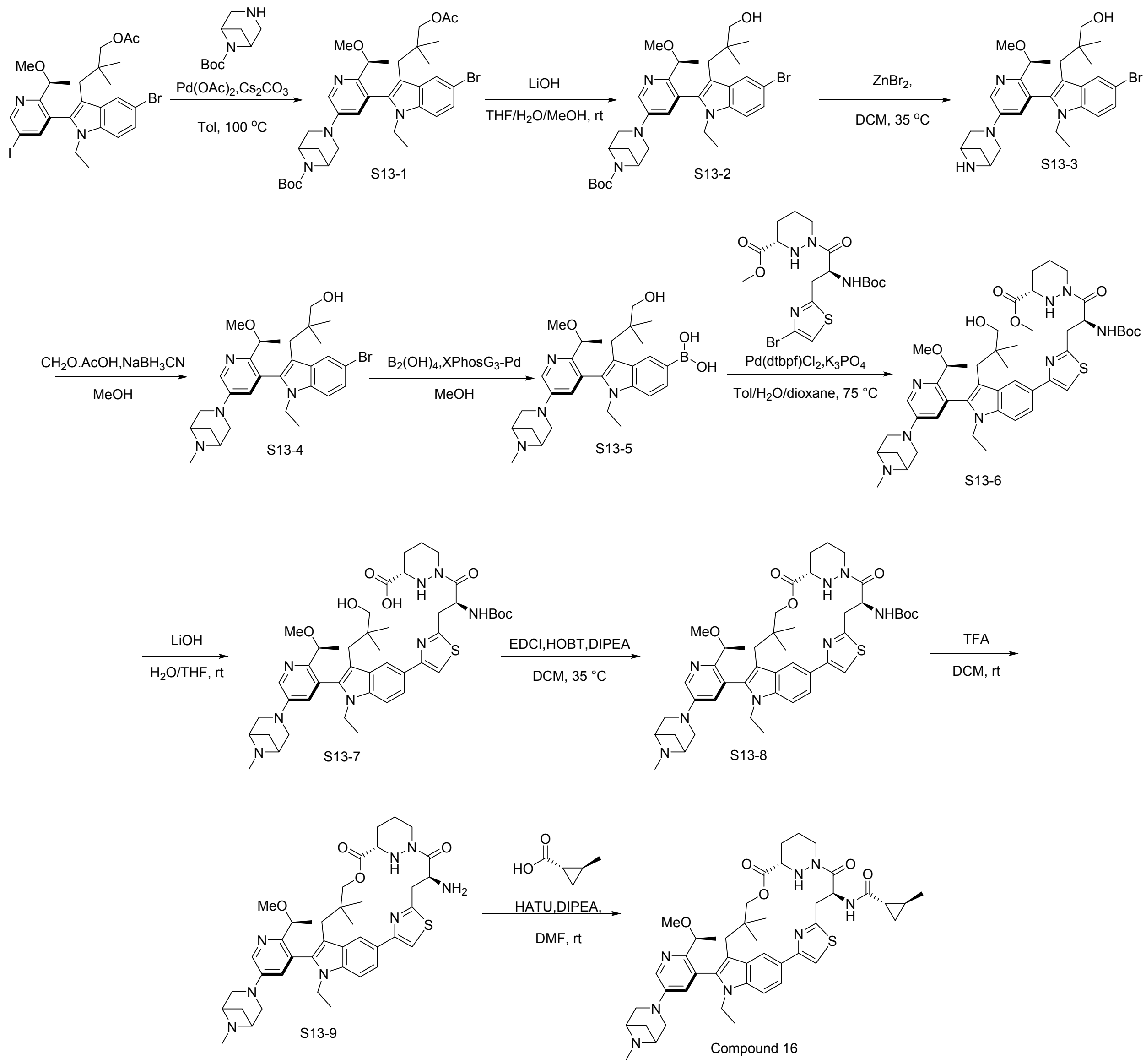

tert-Butyl 3-(5-(3-(3-acetoxy-2,2-dimethylpropyl)-5-bromo-1-ethyl-1H-indol-2-yl)-6-((S)-1-methoxyethyl)pyridin-3-yl)-3,6-diazabicyclo[3.1.1]heptane-6-carboxylate (S13-1) To a stirred solution of (*S*)-3-(5-bromo-1-ethyl-2-(5-iodo-2-(1-methoxyethyl)pyridin-3-yl)-1*H*-indol-3-yl)-2,2-dimethylpropyl acetate (3 g, 4.89 mmol) and *tert*-butyl 3,6-diazabicyclo[3.1.1]heptane-6-carboxylate (1.16 g, 5.87 mmol) in toluene (30 mL) was added $Pd(OAc)_2$ (54.91 mg, 0.25 mmol) and BINAP (60.92 mg, 0.10 mmol) and Cs_2CO_3 (3.98 g, 12.23 mmol) in portions at 100 °C under a nitrogen atmosphere. The resulting mixture was filtered, the filter cake was washed with EtOAc (3 x 20 mL). The filtrate was concentrated under reduced pressure. The residue was purified by silica gel column chromatography, eluted with Pet. ether / EtOAc (1:1) to afford *tert*-butyl 3-(5-(3-(3-acetoxy-2,2-dimethylpropyl)-5-bromo-1-ethyl-1*H*-indol-2-yl)-

6-((*S*)-1-methoxyethyl)pyridin-3-yl)-3,6-diazabicyclo[3.1.1]heptane-6-carboxylate (2.5 g) ^{1}H NMR (400 MHz, DMSO-d6) δ 8.30 (d, J = 2.9 Hz, 1H), 7.79 (d, J = 1.9 Hz, 1H), 7.50 (d, J = 8.7 Hz, 1H), 7.28 (dd, J = 8.7, 1.9 Hz, 1H), 7.01 (d, J = 2.9 Hz, 1H), 4.21 (d, J = 6.2 Hz, 2H), 4.09 – 3.97 (m, 2H), 3.81 (d, J = 6.3 Hz, 3H), 3.65 – 3.57 (m, 2H), 3.32 (s, 1H), 2.78 (s, 3H), 2.70 (d, J = 14.2 Hz, 1H), 2.60 – 2.53 (m, 1H), 2.29 (d, J = 14.2 Hz, 1H), 1.87 (s, 3H), 1.52 (d, J = 8.7 Hz, 1H), 1.37 (d, J = 6.2 Hz, 3H), 1.27 (s, 9H), 1.21 – 1.15 (m, 3H), 0.76 (s, 3H), 0.68 (s, 3H). ^{13}C NMR (101 MHz, DMSO) δ 170.52, 156.61, 146.66, 143.34, 137.68, 134.60, 133.32, 130.84, 126.69, 124.07, 122.24, 119.97, 112.41, 112.11, 109.73, 79.79, 75.25, 71.52, 58.86, 55.91, 55.58, 38.86, 36.47, 33.11, 29.49, 28.98, 28.31, 25.50, 25.03, 20.99, 18.17, 15.43. HRMS (ESI$^+$, m/z): [M+H] calc'd for $C_{35}H_{47}BrN_4O_5$ 683.2730; found 683.2806.

tert-Butyl 3-(5-(5-bromo-1-ethyl-3-(3-hydroxy-2,2-dimethylpropyl)-1H-indol-2-yl)-6-((S)-1-methoxyethyl)pyridin-3-yl)-3,6-diazabicyclo[3.1.1]heptane-6-carboxylate (S13-2) To a stirred solution of *tert*-butyl 3-(5-(3-(3-acetoxy-2,2-dimethylpropyl)-5-bromo-1-ethyl-1*H*-indol-2-yl)-6-((*S*)-1-methoxyethyl)pyridin-3-yl)-3,6-diazabicyclo[3.1.1]heptane-6-carboxylate (2.5 g, 3.66 mmol) in THF (10 mL) and H_2O (10 mL) and methanol (10 mL) was added LiOH.H_2O (613.73 mg, 14.63 mmol) in portions at room temperature under an air atmosphere. The aqueous layer was extracted with EtOAc (3 x 30 mL). The resulting mixture was concentrated under reduced pressure. This resulted in *tert*-butyl 3-(5-(5-bromo-1-ethyl-3-(3-hydroxy-2,2-dimethylpropyl)-1*H*-indol-2-yl)-6-((*S*)-1-methoxyethyl)pyridin-3-yl)-3,6-diazabicyclo[3.1.1]heptane-6-carboxylate (3 g) as a solid. ^{1}H NMR (400 MHz, DMSO-d6) δ 8.30 (d, J = 2.9 Hz, 1H), 7.95 – 7.88 (m, 1H), 7.47 (dd, J = 8.7, 5.9 Hz, 1H), 7.26 (dd, J = 8.7, 1.9 Hz, 1H), 7.04 – 6.96 (m, 1H), 4.60 – 4.52 (m, 1H), 4.21 (d, J = 6.2 Hz, 2H), 4.07 – 4.01 (m, 2H), 3.98 – 3.94 (m, 1H), 3.86 (s, 2H), 3.32 (s, 1H), 3.12 – 3.05 (m, 1H), 2.99 (d, J = 6.4 Hz, 1H), 2.82 (s, 3H), 2.65 (d, J = 14.1 Hz, 1H), 2.59 – 2.53 (m, 1H), 2.15 (d, J = 13.8 Hz, 1H), 1.99 (s, 1H), 1.53 (d, J = 8.7 Hz, 1H), 1.35 (d, J = 6.2 Hz, 3H), 1.27 (s, 9H), 1.19 – 1.09 (m, 4H), 0.60 (d, J = 6.6 Hz, 5H). ^{13}C NMR (101 MHz, DMSO) δ 170.81, 156.64, 146.86, 143.30, 137.46, 136.99, 134.53, 133.21, 131.01, 126.92, 123.96, 122.55, 120.14, 112.20, 111.96, 110.86, 79.82, 75.31, 70.26, 60.23, 56.49, 56.05, 55.58, 46.94, 38.76, 38.08, 37.85, 33.21, 31.75, 28.98, 28.31, 25.52, 25.03, 21.21, 19.00, 18.09, 15.67, 15.44, 14.53. HRMS (ESI$^+$, m/z): [M+H] calc'd for $C_{33}H_{45}BrN_4O_4$ 641.2624; found 641.2702.

3-(2-(5-(3,6-Diazabicyclo[3.1.1]heptan-3-yl)-2-((S)-1-methoxyethyl)pyridin-3-yl)-5-bromo-1-ethyl-1H-indol-3-yl)-2,2-dimethylpropan-1-ol (S13-3) To a stirred solution of *tert*-butyl 3-(5-(5-bromo-1-ethyl-3-(3-hydroxy-2,2-dimethylpropyl)-1*H*-indol-2-yl)-6-((*S*)-1-methoxyethyl)pyridin-3-yl)-3,6-diazabicyclo[3.1.1]heptane-6-carboxylate (3 g, 4.68 mmol) in DCM (30 mL) was added Zinc bromide (21.06 g, 93.50 mmol) in portions at 35 °C under an air atmosphere. The reaction was quenched with $NaHCO_3$(aq.) at 0 °C. The resulting mixture was filtered, the filter cake was washed with DCM (3 x 30

mL). The aqueous layer was extracted with DCM (3 x 50 mL). The resulting mixture was concentrated under reduced pressure. This resulted in 3-(2-(5-(3,6-diazabicyclo[3.1.1]heptan-3-yl)-2-((*S*)-1-methoxyethyl)pyridin-3-yl)-5-bromo-1-ethyl-1*H*-indol-3-yl)-2,2-dimethylpropan-1-ol (2 g) as a solid. ^{1}H NMR (400 MHz, DMSO-d6) δ 8.36 – 8.29 (m, 1H), 7.88 (s, 1H), 7.48 (d, J = 8.7 Hz, 1H), 7.25 (d, J = 8.7 Hz, 1H), 7.07 – 6.99 (m, 1H), 4.56 – 5.48 (m, 3H), 4.09 – 4.05 (m, J = 14.5, 7.2, 6.8 Hz, 1H), 3.98 – 3.92 (m, 2H), 3.84 (s, 3H), 3.75 (d, J = 11.8 Hz, 1H), 3.00 – 2.87 (m, 4H), 2.83 (s, 3H), 2.64 (d, J = 14.0 Hz, 1H), 2.17 (d, J = 14.1 Hz, 1H), 1.90 (d, J = 9.9 Hz, 1H), 1.34 (d, J = 6.3 Hz, 3H), 1.16 – 1.08 (m, 4H), 0.62 (d, J = 4.9 Hz, 6H). ^{13}C NMR (101 MHz, DMSO) δ 147.67, 142.71, 137.22, 134.55, 133.71, 130.97, 126.97, 124.02, 122.59, 120.93, 112.32, 111.97, 110.98, 75.29, 70.22, 58.08, 55.71, 55.49, 48.10, 46.01, 38.83, 38.12, 33.27, 29.33, 25.47, 25.20, 18.15, 15.48, 9.00. HRMS (ESI$^+$, m/z): [M+H] calc'd for $C_{28}H_{37}BrN_4O_2$ 541.2100; found 541.2168.

3-(5-Bromo-1-ethyl-2-(2-((S)-1-methoxyethyl)-5-(6-methyl-3,6-diazabicyclo[3.1.1]heptan-3-yl)pyridin-3-yl)-1H-indol-3-yl)-2,2-dimethylpropan-1-ol (S13-4) To a stirred solution of 3-(2-(5-(3,6-diazabicyclo[3.1.1]heptan-3-yl)-2-((*S*)-1-methoxyethyl)pyridin-3-yl)-5-bromo-1-ethyl-1*H*-indol-3-yl)-2,2-dimethylpropan-1-ol (2 g, 3.69 mmol) and formaldehyde solution (665.35 mg, 22.16 mmol) in MeOH (20 mL) was added AcOH (665.35 mg, 11.08 mmol) and sodium cyanoborahydride (1.16 g, 18.47 mmol) in portions at room temperature under an air atmosphere. The reaction was quenched with $NaHCO_3$(aq.) at room temperature. The aqueous layer was extracted with EtOAc (3 x 50 mL). The resulting mixture was concentrated under reduced pressure. The residue was purified by silica gel column chromatography, eluted with Pet. ether / EtOAc (1:3) to afford 3-(5-bromo-1-ethyl-2-(2-((*S*)-1-methoxyethyl)-5-(6-methyl-3,6-diazabicyclo[3.1.1]heptan-3-yl)pyridin-3-yl)-1*H*-indol-3-yl)-2,2-dimethylpropan-1-ol (1.2 g) as a solid. ^{1}H NMR (400 MHz, DMSO-d6) δ 8.33 (d, J = 3.1 Hz, 1H), 7.91 (d, J = 2.0 Hz, 1H), 7.48 (d, J = 8.6 Hz, 1H), 7.27 (dd, J = 8.6, 1.9 Hz, 1H), 7.00 (d, J = 3.0 Hz, 1H), 5.77 (s, 1H), 4.70 – 4.46 (m, 1H), 4.06 – 3.96 (m, 1H), 3.93 – 3.85 (m, 1H), 3.80 – 3.72 (m, 1H), 3.65 – 3.51 (m, 3H), 3.46 (d, J = 11.3 Hz, 1H), 3.38 – 3.34 (m, 2H), 3.10 (d, J = 10.3 Hz, 1H), 3.06 – 2.97 (m, 1H), 2.87 (s, 3H), 2.70 – 2.61 (m, 5H), 2.18 (d, J = 14.0 Hz, 1H), 1.58 (d, J = 8.3 Hz, 1H), 1.37 (d, J = 6.2 Hz, 3H), 1.23 – 1.11 (m, 4H), 0.64 (d, J = 4.2 Hz, 6H). ^{13}C NMR (101 MHz, DMSO) δ 146.60, 142.88, 137.57, 134.51, 133.20, 131.04, 126.84, 123.91, 122.56, 120.01, 112.19, 111.94, 110.83, 75.33, 70.80, 70.38, 58.59, 56.05, 55.62, 55.58, 55.38, 46.05, 38.85, 38.30, 38.17, 33.76, 33.25, 30.10, 25.42, 25.16, 25.07, 23.08, 19.21, 18.40, 18.21, 15.46, 15.22, 11.10, 7.68. HRMS (ESI$^+$, m/z): [M+H] calc'd for $C_{29}H_{39}BrN_4O_2$ 555.2256; found 555.2318.

(1-Ethyl-3-(3-hydroxy-2,2-dimethylpropyl)-2-(2-((S)-1-methoxyethyl)-5-(6-methyl-3,6-diazabicyclo[3.1.1]heptan-3-yl)pyridin-3-yl)-1H-indol-5-yl)boronic acid (S13-5) To a stirred solution of

3-(5-bromo-1-ethyl-2-(2-((*S*)-1-methoxyethyl)-5-(6-methyl-3,6-diazabicyclo[3.1.1]heptan-3-yl)pyridin-3-yl)-1*H*-indol-3-yl)-2,2-dimethylpropan-1-ol (1.1 g, 1.98 mmol) and (dihydroxyboranyl)boronic acid (532.52 mg, 5.94 mmol) in methanol (5 mL) and 2-methyltetrahydrofuran (10 mL) was added potassium 2,2-dimethylpropanoate (971.73 mg, 6.93 mmol) and XPhos Pd G3 (167.60 mg, 0.20 mmol) in portions at 40 °C under a nitrogen atmosphere. The resulting mixture was filtered, the filter cake was washed with EtOAc (3 x 30 mL). The resulting mixture was washed with brine (1 x 50 mL). The aqueous layer was extracted with EtOAc (2 x 50 mL). The resulting mixture was concentrated under reduced pressure. This resulted in (1-ethyl-3-(3-hydroxy-2,2-dimethylpropyl)-2-(2-((*S*)-1-methoxyethyl)-5-(6-methyl-3,6-diazabicyclo[3.1.1]heptan-3-yl)pyridin-3-yl)-1*H*-indol-5-yl)boronic acid (1.4 g) as a solid. ^{1}H NMR (400 MHz, DMSO-d6) δ 8.33 (d, J = 7.5 Hz, 1H), 8.22 (d, J = 8.0 Hz, 1H), 7.87 (s, 1H), 7.71 (dd, J = 31.6, 8.3 Hz, 1H), 7.46 (dd, J = 25.4, 8.2 Hz, 2H), 7.04 (d, J = 14.8 Hz, 1H), 4.49 (s, 1H), 4.26 – 4.06 (m, 3H), 3.90 (s, 1H), 3.74 (d, J = 9.4 Hz, 1H), 3.60 (d, J = 6.0 Hz, 3H), 3.42 – 3.32(m, 3H), 3.14 (d, J = 10.2 Hz, 1H), 3.09 – 3.00 (m, 1H), 2.87 (s, 3H), 2.71 (d, J = 13.9 Hz, 2H), 2.28 (d, J = 13.9 Hz, 1H), 2.03 (s, 3H), 1.58 (d, J = 8.2 Hz, 1H), 1.45 – 1.33 (m, 4H), 1.31 – 1.25 (m, 2H), 1.20 – 1.12 (m, 3H), 0.97 – 0.88 (m, 1H), 0.73 (s, 3H), 0.65 (s, 3H). ^{13}C NMR (101 MHz, DMSO) δ 146.81, 142.86, 137.43, 137.34, 135.98, 135.60, 133.16, 133.00, 128.92, 127.63, 127.52, 127.41, 127.29, 123.63, 120.51, 120.19, 111.51, 111.10, 109.02, 75.26, 75.11, 71.39, 58.52, 56.08, 55.67, 55.61, 55.34, 38.83, 38.57, 38.38, 38.09, 33.85, 32.94, 31.81, 30.14, 25.72, 25.42, 25.29, 25.14, 25.10, 24.29, 19.36, 18.71, 18.42, 15.77, 15.58, 15.52. HRMS (ESI^{+}, m/z): [M+H] calc'd for $C_{29}H_{41}BN_4O_4$ 521.3221; found 521.3289.

Methyl (3S)-1-((2S)-2-((tert-butoxycarbonyl)amino)-3-(4-(1-ethyl-3-(3-hydroxy-2,2-dimethylpropyl)-2-(2-((S)-1-methoxyethyl)-5-(6-methyl-3,6-diazabicyclo[3.1.1]heptan-3-yl)pyridin-3-yl)-1H-indol-5-yl)thiazol-2-yl)propanoyl)hexahydropyridazine-3-carboxylate (S13-6) To a stirred solution of (1-ethyl-3-(3-hydroxy-2,2-dimethylpropyl)-2-(2-((*S*)-1-methoxyethyl)-5-(6-methyl-3,6-diazabicyclo[3.1.1]heptan-3-yl)pyridin-3-yl)-1*H*-indol-5-yl)boronic acid (1.23 g, 2.36 mmol) and methyl (3*S*)-1-[(2*S*)-3-(4-bromo-1,3-thiazol-2-yl)-2-[(*tert*-butoxycarbonyl)amino]propanoyl]-1,2-diazinane-3-carboxylate (18.34 mg, 0.04 mmol) in toluene (12 mL) and 1,4-dioxane (4 mL) and H_2O (4 mL) was added K_3PO_4 (1.25 g, 5.91 mmol) and Pd(dtbpf)CL_2 (154.02 mg, 0.24 mmol) in portions at 75 °C under a nitrogen atmosphere. The mixture was allowed to cool down to room temperature. The aqueous layer was extracted with EtOAc (3 x 30 mL). The resulting mixture was concentrated under reduced pressure. The residue was purified by silica gel column chromatography, eluted with Pet. ether / EtOAc (1:3) to afford methyl (3*S*)-1-((2*S*)-2-((*tert*-butoxycarbonyl)amino)-3-(4-(1-ethyl-3-(3-hydroxy-2,2-dimethylpropyl)-2-(2-((*S*)-1-methoxyethyl)-5-(6-methyl-3,6-diazabicyclo[3.1.1]heptan-3-yl)pyridin-3-yl)-1*H*-indol-5-yl)thiazol-2-yl)propanoyl)hexahydropyridazine-3-carboxylate (1.4 g) as a solid. ^{1}H NMR (400 MHz, DMSO-d6) δ

8.31 (d, J = 2.9 Hz, 1H), 8.23 (s, 1H), 7.77 (d, J = 5.8 Hz, 2H), 7.52 (d, J = 8.5 Hz, 1H), 7.02 (d, J = 2.9 Hz, 1H), 5.42 – 5.26 (m, 2H), 4.46 (s, 1H), 4.19 – 4.01 (m, 2H), 3.92 – 3.84 (m, 2H), 3.61 (s, 3H), 3.57 (dd, J = 20.7, 8.5 Hz, 3H), 3.47 (d, J = 11.3 Hz, 3H), 3.41 – 3.35 (m, 3H), 3.13 (d, J = 10.2 Hz, 1H), 3.07 – 3.00 (m, 1H), 2.87 (s, 3H), 2.73 (d, J = 13.8 Hz, 1H), 2.47 (d, J = 7.0 Hz, 1H), 2.27 (d, J = 14.0 Hz, 1H), 2.03 (s, 3H), 1.94 – 1.84 (m, 1H), 1.73 – 1.65 (m, 2H), 1.62 – 1.52 (m, 2H), 1.38 (d, J = 5.1 Hz, 12H), 1.27 – 1.15 (m, 5H), 0.68 (d, J = 19.8 Hz, 6H). ^{13}C NMR (101 MHz, DMSO) δ 171.95, 171.33, 166.08, 155.95, 155.24, 146.75, 142.91, 136.74, 135.73, 133.05, 129.44, 129.17, 127.23, 126.19, 120.54, 120.15, 118.12, 111.88, 111.60, 110.23, 78.58, 75.33, 71.01, 58.57, 58.30, 55.63, 55.37, 52.19, 51.04, 41.23, 38.73, 38.33, 38.11, 36.00, 33.72, 31.88, 30.15, 29.49, 28.66, 27.50, 25.27, 25.19, 22.71, 18.28, 15.85, 15.58. HRMS (ESI$^+$, m/z): [M+H] calc'd for $C_{46}H_{64}N_8O_7S$ 873.4619; found 873.4680.

(3S)-1-((2S)-2-((tert-Butoxycarbonyl)amino)-3-(4-(1-ethyl-3-(3-hydroxy-2,2-dimethylpropyl)-2-(2-((S)-1-methoxyethyl)-5-(6-methyl-3,6-diazabicyclo[3.1.1]heptan-3-yl)pyridin-3-yl)-1H-indol-5-yl)thiazol-2-yl)propanoyl)hexahydropyridazine-3-carboxylic acid (S13-7) To a stirred solution of methyl (3*S*)-1-((2*S*)-2-((*tert*-butoxycarbonyl)amino)-3-(4-(1-ethyl-3-(3-hydroxy-2,2-dimethylpropyl)-2-(2-((*S*)-1-methoxyethyl)-5-(6-methyl-3,6-diazabicyclo[3.1.1]heptan-3-yl)pyridin-3-yl)-1*H*-indol-5-yl)thiazol-2-yl)propanoyl)hexahydropyridazine-3-carboxylate (1.3 g, 1.49 mmol) in THF (10 mL) and H_2O (10 mL) was added LiOH.H_2O (249.90 mg, 5.96 mmol) in portions at room temperature under an air atmosphere. The mixture was cooled to 0 °C. The mixture was acidified to pH 5 with 1N HCl(aq.). The aqueous layer was extracted with DCM (3 x 40 mL). The resulting mixture was concentrated under reduced pressure. This resulted in (3*S*)-1-((2*S*)-2-((*tert*-butoxycarbonyl)amino)-3-(4-(1-ethyl-3-(3-hydroxy-2,2-dimethylpropyl)-2-(2-((*S*)-1-methoxyethyl)-5-(6-methyl-3,6-diazabicyclo[3.1.1]heptan-3-yl)pyridin-3-yl)-1*H*-indol-5-yl)thiazol-2-yl)propanoyl)hexahydropyridazine-3-carboxylic acid (1.2 g) as a solid. ^{1}H NMR (400 MHz, DMSO-d6) δ 8.36 – 8.22 (m, 2H), 7.76 (s, 2H), 7.51 (d, J = 8.5 Hz, 1H), 7.04 (d, J = 3.0 Hz, 1H), 6.54 (d, J = 8.5 Hz, 1H), 5.25 (s, 1H), 4.94 (d, J = 11.2 Hz, 1H), 4.21 – 4.07 (m, 3H), 3.91 (dd, J = 14.7, 7.5 Hz, 1H), 3.79 (s, 2H), 3.61 (d, J = 11.1 Hz, 1H), 3.50 (d, J = 17.5 Hz, 3H), 3.36 (d, J = 5.3 Hz, 2H), 3.19 – 2.97 (m, 3H), 2.87 (s, 3H), 2.72 (d, J = 14.1 Hz, 1H), 2.61 (s, 1H), 2.32 – 2.23 (m, 1H), 2.16 (s, 2H), 1.99 – 1.89 (m, 1H), 1.78 – 1.62 (m, 2H), 1.60 – 1.48 (m, 2H), 1.38 (d, J = 5.6 Hz, 12H), 1.24 (d, J = 6.1 Hz, 1H), 1.21 – 1.15 (m, 3H), 1.11 (s, 2H), 0.78 – 0.62 (m, 6H). ^{13}C NMR (101 MHz, DMSO) δ 179.91, 174.08, 170.87, 165.88, 155.91, 155.20, 147.05, 142.73, 136.63, 135.72, 133.20, 129.44, 127.26, 126.21, 120.54, 120.40, 118.22, 111.89, 111.70, 110.25, 78.63, 75.31, 70.92, 59.57, 55.65, 51.02, 41.39, 38.72, 38.31, 38.19, 35.76, 33.68, 28.66, 27.51, 25.32, 25.23, 23.47, 18.28, 15.59. HRMS (ESI$^+$, m/z): [M+H] calc'd for $C_{46}H_{64}N_8O_7S$ 859.4462; found 859.4523.

tert-Butyl ((6^3S,4S,Z)-1^1-ethyl-1^2-(2-((S)-1-methoxyethyl)-5-(6-methyl-3,6-diazabicyclo[3.1.1]heptan-3-yl)pyridin-3-yl)-10,10-dimethyl-5,7-dioxo-6^1,6^2,6^3,6^4,6^5,6^6-hexahydro-1^1H-8-oxa-2(4,2)-thiazola-1(5,3)-indola-6(1,3)-pyridazinacycloundecaphane-4-yl)carbamate (S13-8) To a stirred solution of (3*S*)-1-((2*S*)-2-((*tert*-butoxycarbonyl)amino)-3-(4-(1-ethyl-3-(3-hydroxy-2,2-dimethylpropyl)-2-(2-((*S*)-1-methoxyethyl)-5-(6-methyl-3,6-diazabicyclo[3.1.1]heptan-3-yl)pyridin-3-yl)-1*H*-indol-5-yl)thiazol-2-yl)propanoyl)hexahydropyridazine-3-carboxylic acid (1.06 g, 1.21 mmol) and HOBT (1.63 g, 12.06 mmol) in DCM (330 mL) was added DIPEA (5.45 g, 42.21 mmol) and EDCI (6.55 g, 42.21 mmol) in portions at 35 °C under an air atmosphere. The resulting mixture was washed with 1N HCl(aq.)(1 x 100 mL), $NaHCO_3$(aq.)(1 x 100 mL), then H_2O (1 x 100 mL). The organic layer was concentrated under reduced pressure. The residue was purified by silica gel column chromatography, eluted with Pet. ether / EtOAc (1:3) to afford *tert*-butyl ((6^3*S*,4*S*,*Z*)-1^1-ethyl-1^2-(2-((*S*)-1-methoxyethyl)-5-(6-methyl-3,6-diazabicyclo[3.1.1]heptan-3-yl)pyridin-3-yl)-10,10-dimethyl-5,7-dioxo-6^1,6^2,6^3,6^4,6^5,6^6-hexahydro-1^1*H*-8-oxa-2(4,2)-thiazola-1(5,3)-indola-6(1,3)-pyridazinacycloundecaphane-4-yl)carbamate (420 mg) as a solid. ^{1}H NMR (400 MHz, DMSO-d6) δ 8.50 (s, 1H), 8.31 (d, J = 2.9 Hz, 1H), 7.81 (s, 1H), 7.76 – 7.71 (m, 1H), 7.55 (d, J = 8.6 Hz, 1H), 7.31 (d, J = 9.1 Hz, 1H), 6.93 (d, J = 2.9 Hz, 1H), 5.76 (s, 1H), 5.24 (s, 1H), 5.07 (d, J = 12.0 Hz, 1H), 4.35 – 4.13 (m, 5H), 3.62 – 3.54 (m, 4H), 3.50 (dd, J = 11.2, 5.1 Hz, 2H), 3.39 – 3.33 (m, 3H), 3.21 (d, J = 25.4 Hz, 5H), 2.97 (d, J = 14.3 Hz, 1H), 2.81 – 2.73 (m, 1H), 2.46 (d, J = 6.5 Hz, 1H), 2.08 (s, 2H), 2.03 (s, 3H), 1.84 – 1.75 (m, 2H), 1.55 (dd, J = 25.0, 9.2 Hz, 3H), 1.41 (s, 9H), 1.35 (d, J = 6.1 Hz, 3H), 0.96 – 0.90 (m, 6H), 0.38 (s, 3H). ^{13}C NMR (101 MHz, DMSO) δ 170.87, 170.23, 164.90, 153.68, 152.47, 144.49, 141.34, 135.85, 133.31, 130.70, 128.06, 124.97, 124.15, 117.35, 117.11, 115.25, 109.19, 108.77, 76.34, 73.69, 68.60, 56.42, 55.60, 54.10, 53.62, 53.26, 49.02, 41.88, 38.52, 38.31, 38.10, 37.89, 37.68, 37.47, 37.26, 36.07, 35.52, 33.54, 30.86, 29.85, 28.03, 26.82, 26.58, 24.14, 22.76, 21.28, 14.49, 13.81, 13.34, -0.50. HRMS (ESI$^+$, m/z): [M+H] calc'd for $C_{45}H_{60}N_8O_6S$ 841.4357; found 841.4420.

(6^3S,4S,Z)-4-Amino-1^1-ethyl-1^2-(2-((S)-1-methoxyethyl)-5-(6-methyl-3,6-diazabicyclo[3.1.1]heptan-3-yl)pyridin-3-yl)-10,10-dimethyl-6^1,6^2,6^3,6^4,6^5,6^6-hexahydro-1^1H-8-oxa-2(4,2)-thiazola-1(5,3)-indola-6(1,3)-pyridazinacycloundecaphane-5,7-dione (S13-9) To a stirred solution of *tert*-butyl ((6^3*S*,4*S*,*Z*)-1^1-ethyl-1^2-(2-((*S*)-1-methoxyethyl)-5-(6-methyl-3,6-diazabicyclo[3.1.1]heptan-3-yl)pyridin-3-yl)-10,10-dimethyl-5,7-dioxo-6^1,6^2,6^3,6^4,6^5,6^6-hexahydro-1^1*H*-8-oxa-2(4,2)-thiazola-1(5,3)-indola-6(1,3)-pyridazinacycloundecaphane-4-yl)carbamate (400 mg, 0.476 mmol) in DCM (4 mL) was added TFA (2 mL) dropwise at 0 °C under an air atmosphere and stirred for 2 h at room temperature. The mixture was cooled to 0 °C. The mixture was neutralized to pH 9 with $NaHCO_3$(aq.). The aqueous layer was extracted with DCM (3 x 20 mL). The resulting mixture was concentrated under reduced pressure. This

resulted in (6^3S,4S,Z)-4-amino-1^1-ethyl-1^2-(2-((S)-1-methoxyethyl)-5-(6-methyl-3,6-diazabicyclo[3.1.1]heptan-3-yl)pyridin-3-yl)-10,10-dimethyl-6^1,6^2,6^3,6^4,6^5,6^6-hexahydro-1^1H-8-oxa-2(4,2)-thiazola-1(5,3)-indola-6(1,3)-pyridazinacycloundecaphane-5,7-dione (330 mg) as a solid. ^1H NMR (400 MHz, DMSO-d6) δ 8.48 (d, J = 1.6 Hz, 1H), 8.30 (d, J = 2.9 Hz, 1H), 7.78 – 7.68 (m, 2H), 7.54 (d, J = 8.7 Hz, 1H), 6.93 (d, J = 2.9 Hz, 1H), 4.93 (d, J = 12.1 Hz, 1H), 4.37 (d, J = 8.4 Hz, 1H), 4.33 – 4.25 (m, 2H), 4.20 – 4.08 (m, 3H), 3.84 – 3.78 (m, 1H), 3.65 – 3.54 (m, 4H), 3.49 (dd, J = 11.3, 4.6 Hz, 2H), 3.37 (d, J = 9.7 Hz, 4H), 3.20 (s, 3H), 3.08 – 2.90 (m, 2H), 2.78 – 2.69 (m, 2H), 2.55 – 2.42 (m, 3H), 2.11 – 1.98 (m, 4H), 1.85 – 1.68 (m, 2H), 1.58 (d, J = 8.2 Hz, 1H), 1.54 – 1.45 (m, 1H), 1.34 (d, J = 6.2 Hz, 3H), 1.23 (d, J = 7.1 Hz, 2H), 1.19 – 1.04 (m, 2H), 0.91 (dd, J = 14.7, 7.3 Hz, 5H), 0.37 (s, 2H). ^{13}C NMR (101 MHz, DMSO) δ 175.96, 172.40, 168.05, 154.48, 146.61, 143.41, 137.85, 135.40, 132.84, 130.14, 129.38, 127.15, 126.42, 119.52, 119.33, 117.48, 111.33, 111.00, 110.80, 75.80, 70.83, 58.77, 57.84, 56.20, 55.69, 55.38, 51.29, 41.92, 40.91, 38.25, 35.72, 32.95, 30.01, 28.96, 26.47, 26.14, 24.93, 23.46, 18.72, 16.64, 15.92, 15.46, 12.67. HRMS (ESI$^+$, m/z): [M+H] calc'd for $C_{40}H_{52}N_8O_4S$ 741.3832; found 741.3897.

(1S,2S)-N-((6^3S,4S,Z)-1^1-Ethyl-1^2-(2-((S)-1-methoxyethyl)-5-(6-methyl-3,6-diazabicyclo[3.1.1]heptan-3-yl)pyridin-3-yl)-10,10-dimethyl-5,7-dioxo-6^1,6^2,6^3,6^4,6^5,6^6-hexahydro-1^1H-8-oxa-2(4,2)-thiazola-1(5,3)-indola-6(1,3)-pyridazinacycloundecaphane-4-yl)-2-methylcyclopropane-1-carboxamide (Compound 16)

To a stirred solution of (6^3S,4S,Z)-4-amino-1^1-ethyl-1^2-(2-((S)-1-methoxyethyl)-5-(6-methyl-3,6-diazabicyclo[3.1.1]heptan-3-yl)pyridin-3-yl)-10,10-dimethyl-6^1,6^2,6^3,6^4,6^5,6^6-hexahydro-1^1H-8-oxa-2(4,2)-thiazola-1(5,3)-indola-6(1,3)-pyridazinacycloundecaphane-5,7-dione (300 mg, 0.41 mmol) and (1S,2S)-2-methylcyclopropane-1-carboxylic acid (48.64 mg, 0.49 mmol) in DMF (3 mL) was added DIPEA (261.64 mg, 2.03 mmol) and HATU (230.92 mg, 0.61 mmol) in portions at room temperature under an air atmosphere. The residue was purified by reversed-phase flash chromatography with the following conditions: column, C18 silica gel; mobile phase, MeCN in Water (10mmol/L NH_4HCO_3), 10% to 50% gradient in 10 min; detector, UV 254 nm. This resulted in (1S,2S)-N-((6^3S,4S,Z)-1^1-ethyl-1^2-(2-((S)-1-methoxyethyl)-5-(6-methyl-3,6-diazabicyclo[3.1.1]heptan-3-yl)pyridin-3-yl)-10,10-dimethyl-5,7-dioxo-6^1,6^2,6^3,6^4,6^5,6^6-hexahydro-1^1H-8-oxa-2(4,2)-thiazola-1(5,3)-indola-6(1,3)-pyridazinacycloundecaphane-4-yl)-2-methylcyclopropane-1-carboxamide (225 mg) as a solid. ^1H NMR (400 MHz, DMSO-d6) δ 8.45 (d, J = 13.5 Hz, 2H), 8.24 (s, 1H), 7.71 (s, 1H), 7.65 (d, J = 8.6 Hz, 1H), 7.47 (d, J = 8.6 Hz, 1H), 6.86 (s, 1H), 5.68 (s, 1H), 5.51 (s, 1H), 5.01 (d, J = 12.1 Hz, 1H), 4.40 – 4.15 (m, 5H), 3.46 – 3.39 (m, 2H), 3.35 – 3.22 (m, 3H), 3.18 – 3.05 (m, 4H), 2.89 (d, J = 14.4 Hz, 1H), 2.73 – 2.65 (m, 1H), 2.39 (d, J = 7.0 Hz, 1H), 2.01 – 1.93 (m, 5H), 1.72 (d, J = 8.5 Hz, 2H), 1.54 – 1.39 (m, 3H), 1.28 (d, J = 6.1 Hz, 3H), 1.2 0 – 1.08 (m, 4H), 1.00 (s, 5H), 0.88 – 0.82 (m, 7H), 0.71 (s, 2H), 0.51 – 0.41 (m,

1H), 0.31 (s, 3H). ^{13}C NMR (101 MHz, DMSO) δ 170.63, 170.22, 164.61, 152.59, 144.52, 141.32, 135.83, 133.33, 130.72, 128.04, 124.98, 124.14, 117.38, 117.13, 116.39, 115.29, 109.21, 108.76, 73.70, 68.61, 58.10, 56.50, 55.75, 54.09, 53.63, 53.27, 47.26, 38.52, 38.31, 38.10, 37.89, 37.68, 37.47, 37.26, 36.09, 33.55, 30.87, 29.86, 28.00, 27.71, 27.37, 26.78, 24.35, 24.10, 23.48, 22.80, 21.29, 20.42, 19.10, 16.58, 15.94, 14.51, 13.81, 13.34, 12.96, 12.88, 12.44, 11.90, -0.50. HRMS (ESI$^+$, m/z): [M+H] calc'd for $C_{45}H_{58}N_8O_5S$ 823.4251; found 823.4318.

Synthesis of Compound 17

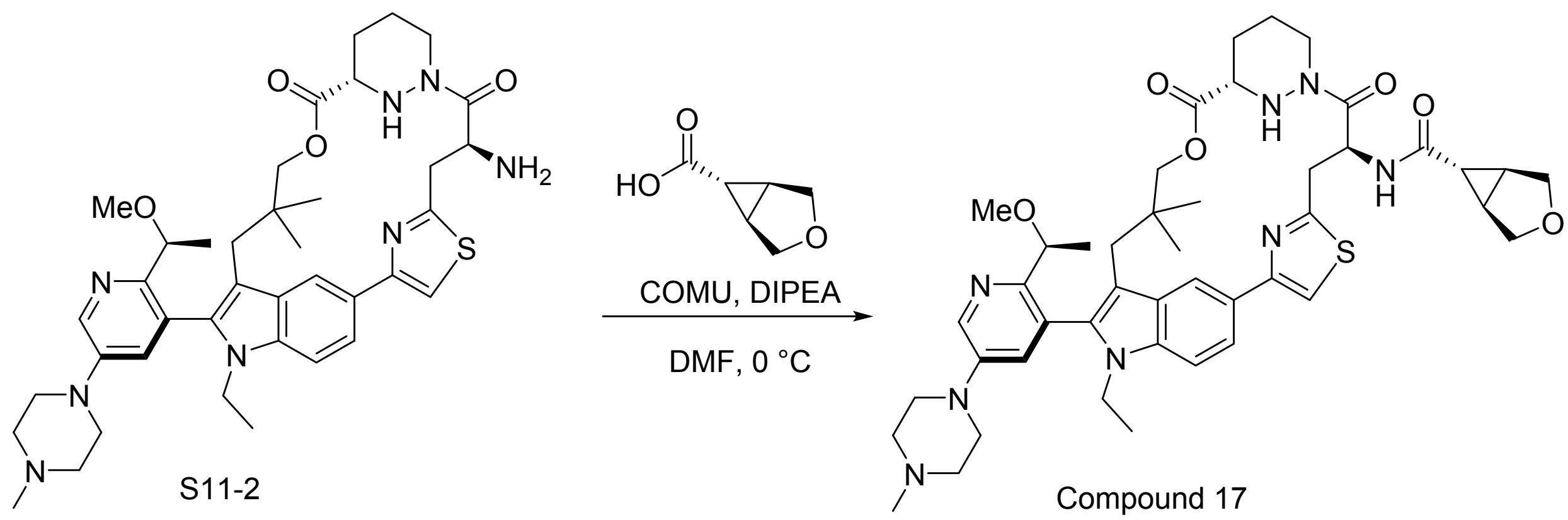

(1R,5S,6r)-N-((6^{3}S,4S,Z)-1^1-Ethyl-1^2-(2-((S)-1-methoxyethyl)-5-(4-methylpiperazin-1-yl)pyridin-3-yl)-10,10-dimethyl-5,7-dioxo-6^1,6^2,6^3,6^4,6^5,6^6-hexahydro-1^{1}H-8-oxa-2(4,2)-thiazola-1(5,3)-indola-6(1,3)-pyridazinacycloundecaphane-4-yl)-3-oxabicyclo[3.1.0]hexane-6-carboxamide (Compound 17) To a stirred mixture of (6^3*S*,4*S*,*Z*)-4-amino-1^1-ethyl-1^2-(2-((*S*)-1-methoxyethyl)-5-(4-methylpiperazin-1-yl)pyridin-3-yl)-10,10-dimethyl-6^1,6^2,6^3,6^4,6^5,6^6-hexahydro-1^1*H*-8-oxa-2(4,2)-thiazola-1(5,3)-indola-6(1,3)-pyridazinacycloundecaphane-5,7-dione (100.00 mg, 0.14 mmol) and (1*R*,5*S*,6*R*)-3-oxabicyclo[3.1.0]hexane-6-carboxylic acid (26.37 mg, 0.21 mmol) in DMF (2 mL) were added DIPEA (177.30 mg, 1.37 mmol) and (*Z*)-(ethyl cyano({[(dimethyliminiumyl)(morpholin-4-yl)methoxy]imino})formate) (117.50 mg, 0.27 mmol) in portions at 0 °C under a nitrogen atmosphere. The resulting mixture was stirred for 1 h at 0 °C. The resulting mixture was diluted with H_2O (10 mL) then extracted with EtOAc (3 x 10 mL). The combined organic layers were washed with brine (3 x 10 mL), dried over anhydrous Na_2SO_4. After filtration, the filtrate was concentrated under reduced pressure. The crude product was purified by Prep-HPLC with the following conditions (Column: XBridge Prep C18 OBD Column, 19*150 mm, 5μm; Mobile Phase A: Water(10MMOL/L NH_4HCO_3), Mobile Phase B: ACN; Flow rate: 25 mL/min; Gradient: 29% B to 53% B in 8 min; Wave Length: 254/220 nm; RT1(min): 7.5) to afford (1*R*,5*S*,6*r*)-*N*-((6^3*S*,4*S*,*Z*)-1^1-ethyl-1^2-(2-((*S*)-1-methoxyethyl)-5-(4-methylpiperazin-1-yl)pyridin-3-yl)-10,10-dimethyl-5,7-dioxo-6^1,6^2,6^3,6^4,6^5,6^6-hexahydro-1^1*H*-8-oxa-2(4,2)-thiazola-1(5,3)-

indola-6(1,3)-pyridazinacycloundecaphane-4-yl)-3-oxabicyclo[3.1.0]hexane-6-carboxamide (50 mg) as a solid. ^{1}H NMR (400 MHz, DMSO-d_6) δ 8.56 (dd, *J* = 48.3, 15.7 Hz, 3H), 7.74 (d, *J* = 18.2 Hz, 2H), 7.53 (d, *J* = 8.5 Hz, 1H), 7.22 (s, 1H), 5.61 (t, *J* = 8.6 Hz, 1H), 5.07 (d, *J* = 11.4 Hz, 1H), 4.49 – 4.02 (m, 5H), 3.85 (t, *J* = 8.0 Hz, 2H), 3.58 (d, *J* = 38.1 Hz, 11H), 2.98 (d, *J* = 13.8 Hz, 1H), 2.79 (d, *J* = 16.0 Hz, 1H), 2.48 (d, *J* = 23.7 Hz, 5H), 2.15 (d, *J* = 38.8 Hz, 4H), 2.03 – 1.88 (m, 2H), 1.74 (d, *J* = 31.5 Hz, 3H), 1.58 – 1.11 (m, 6H), 1.07 – 0.65 (m, 7H), 0.39 (s, 3H). ^{13}C NMR (101 MHz, DMSO-d_6) δ 172.64, 172.29, 171.29, 166.70, 154.74, 149.03, 146.06, 137.53, 135.50, 130.16, 127.12, 126.30, 125.01, 119.61, 117.50, 111.52, 111.37, 75.71, 69.07, 57.90, 55.79, 54.60, 49.56, 47.83, 46.22, 35.67, 26.20, 25.66, 25.51, 24.93, 23.18, 16.55, 15.34. HRMS (ESI$^+$, m/z): [M+H] calc'd for $C_{45}H_{59}N_8O_6S$ 839.42728; found 839.42657.

Synthesis of Compound 18

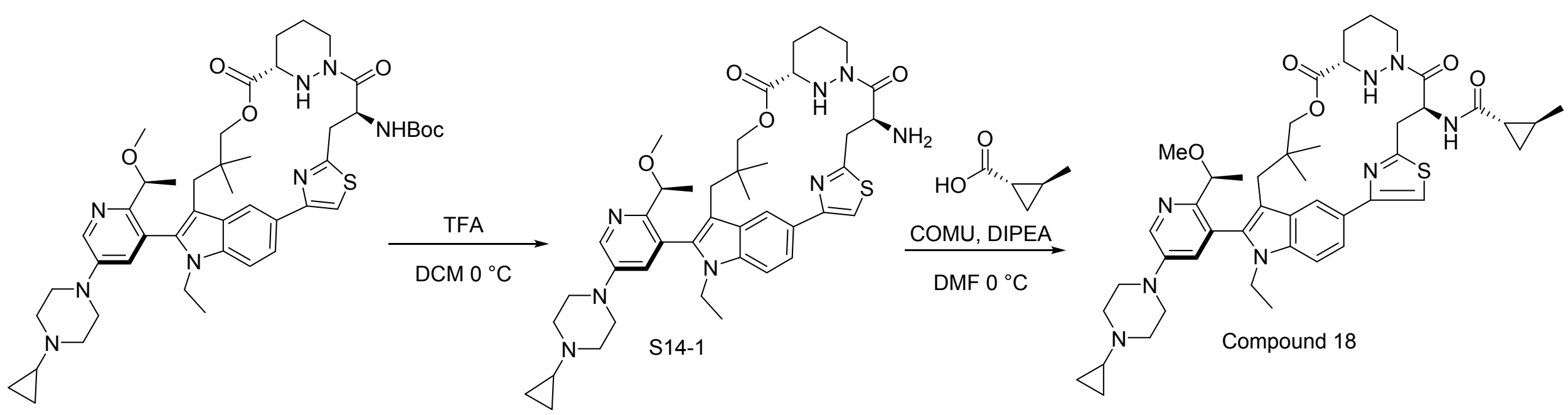

(6^{3}S,4S,Z)-4-Amino-1^{2}-(5-(4-cyclopropylpiperazin-1-yl)-2-((S)-1-methoxyethyl)pyridin-3-yl)-1^{1}-ethyl-10,10-dimethyl-6^{1},6^{2},6^{3},6^{4},6^{5},6^{6}-hexahydro-1^{1}H-8-oxa-2(4,2)-thiazola-1(5,3)-indola-6(1,3)-pyridazinacycloundecaphane-5,7-dione (S14-1) Into a 40 mL vial were added *tert*-butyl ((6^{3}*S*,4*S*,*Z*)-1^{2}-(5-(4-cyclopropylpiperazin-1-yl)-2-((*S*)-1-methoxyethyl)pyridin-3-yl)-1^{1}-ethyl-10,10-dimethyl-5,7-dioxo-6^{1},6^{2},6^{3},6^{4},6^{5},6^{6}-hexahydro-1^{1}*H*-8-oxa-2(4,2)-thiazola-1(5,3)-indola-6(1,3)-pyridazinacycloundecaphane-4-yl)carbamate (200 mg, 0.23 mmol) and TFA (3 mL) at 0 °C. The resulting mixture was stirred at 0 °C for 3h under an air atmosphere. The resulting mixture was concentrated under reduced pressure. The residue was purified by reversed-phase flash chromatography with the following conditions: column, C18 silica gel; mobile phase, MeCN in Water (10mmol/L NH4HCO3), 10% to 50% gradient in 10 min; detector, UV 254 nm. to afford (6^{3}*S*,4*S*,*Z*)-4-amino-1^{2}-(5-(4-cyclopropylpiperazin-1-yl)-2-((*S*)-1-methoxyethyl)pyridin-3-yl)-1^{1}-ethyl-10,10-dimethyl-6^{1},6^{2},6^{3},6^{4},6^{5},6^{6}-hexahydro-1^{1}*H*-8-oxa-2(4,2)-thiazola-1(5,3)-indola-6(1,3)-pyridazinacycloundecaphane-5,7-dione (160 mg) as a solid. ^{1}H NMR (400 MHz, DMSO-d_6) δ 8.46 (dd, *J* = 14.4, 2.2 Hz, 2H), 7.77 (s, 1H), 7.73 (dd, *J* = 8.6, 1.6 Hz, 1H), 7.55 (d, *J* = 8.6 Hz, 1H), 7.22 (d, *J* = 2.9 Hz, 1H), 4.93 (d, *J* = 12.1 Hz, 1H), 4.35 (d, *J* = 8.5 Hz, 1H), 4.28 (p, *J* = 7.4, 6.7 Hz, 2H), 4.21 – 4.08 (m, 3H), 3.57 (q, *J* = 10.9 Hz, 2H), 3.22 (d, *J* = 7.1 Hz, 8H), 2.95 (d, *J* =

14.3 Hz, 1H), 2.80 – 2.64 (m, 6H), 2.43 (d, *J* = 14.3 Hz, 1H), 2.08 (s, 2H), 2.11 – 2.03 (m, 1H), 1.86 (s, 2H), 1.77 (q, *J* = 11.9, 10.2 Hz, 2H), 1.66 (td, *J* = 6.6, 3.3 Hz, 1H), 1.50 (qd, *J* = 12.2, 4.4 Hz, 1H), 1.33 (d, *J* = 6.1 Hz, 3H), 0.90 (q, *J* = 5.7, 4.3 Hz, 6H), 0.44 (dt, *J* = 6.6, 3.6 Hz, 2H), 0.34 (s, 5H). ^{13}C NMR (101 MHz, DMSO-d_6) δ 174.20, 170.31, 166.13, 152.24, 146.82, 144.05, 135.53, 135.36, 133.28, 128.01, 125.01, 124.33, 122.96, 117.43, 116.40, 115.31, 109.37, 108.85, 108.69, 73.60, 68.65, 55.67, 53.68, 50.61, 49.22, 45.89, 40.15, 38.76, 36.38, 36.04, 33.60, 30.81, 26.87, 24.02, 22.76, 21.38, 14.39, 13.24, 4.00. HRMS (ESI$^+$, m/z): [M+H] calc'd for $C_{41}H_{54}N_8O_4S$ 754.39887; found 755.40320.

(1S,2S)-N-((6^{3}S,4S,Z)-1^2-(5-(4-Cyclopropylpiperazin-1-yl)-2-((S)-1-methoxyethyl)pyridin-3-yl)-1^1-ethyl-10,10-dimethyl-5,7-dioxo-6^1,6^2,6^3,6^4,6^5,6^6-hexahydro-1^{1}H-8-oxa-2(4,2)-thiazola-1(5,3)-indola-6(1,3)-pyridazinacycloundecaphane-4-yl)-2-methylcyclopropane-1-carboxamide (Compound 18) Into a 100 mL round-bottom flask were added (6^3*S*,4*S*,*Z*)-4-amino-1^2-(5-(4-cyclopropylpiperazin-1-yl)-2-((*S*)-1-methoxyethyl)pyridin-3-yl)-1^1-ethyl-10,10-dimethyl-6^1,6^2,6^3,6^4,6^5,6^6-hexahydro-1^1*H*-8-oxa-2(4,2)-thiazola-1(5,3)-indola-6(1,3)-pyridazinacycloundecaphane-5,7-dione (150 mg, 0.20 mmol), DMF (3 mL), DIPEA (0.35 mL, 1.99 mmol), (1*S*,2*S*)-2-methylcyclopropane-1-carboxylic acid (29.84 mg, 0.30 mmol) and COMU (127.63 mg, 0.30 mmol) at 0 °C. The resulting mixture was stirred at 0 °C for 1 h under an air atmosphere. The reaction was quenched by the addition of H_2O (20mL) at room temperature. The resulting mixture was extracted with EtOAc (3 x 20 mL). The combined organic layers were washed with brine (1 x 20 mL), dried over anhydrous Na_2SO_4. After filtration, the filtrate was concentrated under reduced pressure. The residue was purified by Prep-TLC (EtOAc), The crude product (150 mg) was purified by Prep-HPLC with the following conditions (Column: XBridge Prep OBD C18 Column, 30*150 mm, 5μm; Mobile Phase A: Water(10mmol/L NH4HCO3), Mobile Phase B: ACN; Flow rate: 60 mL/min mL/min; Gradient: 57%Bto58%Bin10min; Wave Length: 254nm/220nm nm; RT1(min): 8.37) to afford (1*S*,2*S*)-*N*-((6^3*S*,4*S*,*Z*)-1^2-(5-(4-cyclopropylpiperazin-1-yl)-2-((*S*)-1-methoxyethyl)pyridin-3-yl)-1^1-ethyl-10,10-dimethyl-5,7-dioxo-6^1,6^2,6^3,6^4,6^5,6^6-hexahydro-1^1*H*-8-oxa-2(4,2)-thiazola-1(5,3)-indola-6(1,3)-pyridazinacycloundecaphane-4-yl)-2-methylcyclopropane-1-carboxamide (50 mg, 27% yield) as a solid. ^{1}H NMR (400 MHz, DMSO-d_6) δ 8.52 (d, *J* = 8.9 Hz, 1H), 8.46 (dd, *J* = 13.6, 2.2 Hz, 2H), 7.80 (s, 1H), 7.72 (dd, *J* = 8.6, 1.6 Hz, 1H), 7.55 (d, *J* = 8.6 Hz, 1H), 7.21 (d, *J* = 2.9 Hz, 1H), 5.56 (t, *J* = 9.1 Hz, 1H), 5.09 (d, *J* = 12.1 Hz, 1H), 4.29 (dd, *J* = 15.0, 7.6 Hz, 1H), 4.23 (s, 1H), 4.24 – 4.09 (m, 3H), 3.56 (s, 2H), 3.35 (s, 1H), 3.22 (d, *J* = 5.1 Hz, 6H), 3.14 (dd, *J* = 14.7, 9.2 Hz, 1H), 2.93 (d, *J* = 14.2 Hz, 1H), 2.76 (s, 1H), 2.68 (t, *J* = 5.3 Hz, 4H), 2.44 (d, *J* = 14.3 Hz, 1H), 2.11 – 2.04 (m, 1H), 1.79 (s, 2H), 1.65 (tt, *J* = 6.6, 3.6 Hz, 1H), 1.55 – 1.45 (m, 2H), 1.32 (d, *J* = 6.0 Hz, 3H), 1.07 (s, 4H), 0.93 – 0.83 (m, 7H), 0.55 (dd, *J* = 8.0, 5.2 Hz, 1H), 0.43 (dt, *J* = 6.1, 2.8 Hz, 2H), 0.34 (q, *J* = 3.2 Hz, 5H). ^{13}C NMR (101 MHz, DMSO-d_6) δ 172.75, 172.74, 172.34, 166.71, 154.66, 149.02, 146.17, 137.69, 137.54, 135.46, 130.13,

127.08, 126.26, 125.07, 119.55, 117.38, 111.49, 111.39, 110.90, 75.71, 70.70, 57.85, 55.85, 52.73, 49.35, 48.03, 38.51, 38.21, 38.14, 35.67, 32.95, 28.89, 26.20, 24.89, 23.40, 22.53, 18.06, 16.54, 15.36, 15.08, 14.99, 6.12. HRMS (ESI$^+$, m/z): [M+H] calc'd for $C_{46}H_{60}N_8O_5S$ 836.44074; found 837.44489.

Synthesis of Compound 19

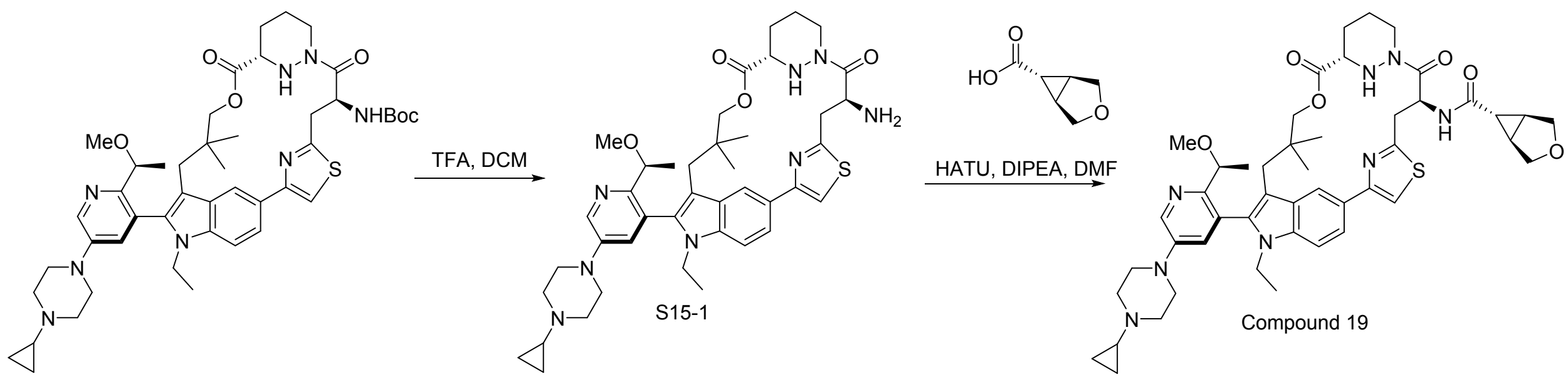

(6^3S,4S,Z)-4-Amino-1^2-(5-(4-cyclopropylpiperazin-1-yl)-2-((S)-1-methoxyethyl)pyridin-3-yl)-1^1-ethyl-10,10-dimethyl-6^1,6^2,6^3,6^4,6^5,6^6-hexahydro-1^1H-8-oxa-2(4,2)-thiazola-1(5,3)-indola-6(1,3)-pyridazinacycloundecaphane-5,7-dione (S15-1) To a stirred solution of tert-butyl ((6^3*S*,4*S*,*Z*)-1^2-(5-(4-cyclopropylpiperazin-1-yl)-2-((*S*)-1-methoxyethyl)pyridin-3-yl)-1^1-ethyl-10,10-dimethyl-5,7-dioxo-6^1,6^2,6^3,6^4,6^5,6^6-hexahydro-1^1*H*-8-oxa-2(4,2)-thiazola-1(5,3)-indola-6(1,3)-pyridazinacycloundecaphane-4-yl)carbamate (550 mg, 0.64 mmol) in DCM (3 mL) was added TFA (1 mL) dropwise at 0 °C. The resulting mixture was stirred for 2 h at room temperature then the reaction was basified to pH 9 with saturated $NaHCO_3$ (aq.). The resulting mixture was extracted with EtOAc (3 x 20 mL). The combined organic layers were dried over anhydrous Na_2SO_4. After filtration, the filtrate was concentrated under reduced pressure. This resulted in (6^3*S*,4*S*,*Z*)-4-amino-1^2-(5-(4-cyclopropylpiperazin-1-yl)-2-((*S*)-1-methoxyethyl)pyridin-3-yl)-1^1-ethyl-10,10-dimethyl-6^1,6^2,6^3,6^4,6^5,6^6-hexahydro-1^1*H*-8-oxa-2(4,2)-thiazola-1(5,3)-indola-6(1,3)-pyridazinacycloundecaphane-5,7-dione (230 mg, crude) as a solid. The crude product was used in the next step directly without further purification. ^{1}H NMR (400 MHz, DMSO-d_6) δ 8.46 (dd, *J* = 12.9, 2.3 Hz, 2H), 7.77 (s, 1H), 7.72 (dd, *J* = 8.6, 1.6 Hz, 1H), 7.54 (d, *J* = 8.7 Hz, 1H), 7.22 (d, *J* = 2.9 Hz, 1H), 4.94 (d, *J* = 12.1 Hz, 1H), 4.38 (d, *J* = 8.4 Hz, 1H), 4.31 – 4.20 (m, 2H), 4.18 – 4.06 (m, 3H), 3.57 (q, *J* = 10.9 Hz, 2H), 3.21 (d, *J* = 10.8 Hz, 9H), 2.93 (d, *J* = 14.4 Hz, 1H), 2.78 – 2.64 (m, 7H), 2.44 (d, *J* = 14.4 Hz, 2H), 2.06 (d, *J* = 7.8 Hz, 1H), 1.79 (s, 1H), 1.72 (d, *J* = 12.7 Hz, 1H), 1.66 (dq, *J* = 6.5, 3.3 Hz, 1H), 1.51 (dt, *J* = 12.4, 6.4 Hz, 1H), 1.33 (d, *J* = 6.1 Hz, 4H), 1.25 – 1.11 (m, 1H), 0.94 – 0.86 (m, 6H), 0.43 (dt, *J* = 6.1, 2.9 Hz, 2H), 0.38 – 0.29 (m, 5H). ^{13}C NMR (101 MHz, $CDCl_3$) δ 176.87, 172.41, 167.36, 155.02, 150.05, 146.09, 138.68, 136.86, 135.36, 130.23, 128.92, 128.76, 126.96, 126.42, 125.30, 125.23, 120.29, 120.17, 119.34, 117.99, 111.83, 109.93, 109.88, 109.40, 109.34, 104.95, 77.47, 77.15, 76.84, 75.54, 71.11, 57.54, 56.20, 52.79, 51.39, 48.41, 42.40, 41.95, 38.42, 38.24, 35.71,

32.72, 29.68, 29.17, 26.00, 24.98, 23.36, 16.46, 15.00, 5.89, 5.87, 1.03, 0.03. LCMS (ESI): m/z [M+H] calc'd for $C_{41}H_{54}N_8O_4S$ 754; found 755.

(3R,4R,5S)-N-((6^{3}S,4S,Z)-1^2-(5-(4-Cyclopropylpiperazin-1-yl)-2-((S)-1-methoxyethyl)pyridin-3-yl)-1^1-ethyl-10,10-dimethyl-5,7-dioxo-6^1,6^2,6^3,6^4,6^5,6^6-hexahydro-1^{1}H-8-oxa-2(4,2)-thiazola-1(5,3)-indola-6(1,3)-pyridazinacycloundecaphane-4-yl)tetrahydro-2H-pyran-4-carboxamide (Compound 19)
To a stirred solution of (6^3*S*,4*S*,*Z*)-4-amino-1^2-(5-(4-cyclopropylpiperazin-1-yl)-2-((*S*)-1-methoxyethyl)pyridin-3-yl)-1^1-ethyl-10,10-dimethyl-6^1,6^2,6^3,6^4,6^5,6^6-hexahydro-1^1*H*-8-oxa-2(4,2)-thiazola-1(5,3)-indola-6(1,3)-pyridazinacycloundecaphane-5,7-dione (230 mg, 0.31 mmol) and DIPEA (118.12 mg, 0.92 mmol) in DMF (3 mL) was added (1*R*,5*S*,6*R*)-3-oxabicyclo[3.1.0]hexane-6-carboxylic acid (58.55 mg, 0.46 mmol) and HATU (173.75 mg, 0.46 mmol) in portions at 0 °C. The resulting reaction was stirred for 1 h at room temperature then quenched by the addition of H_2O (5 mL). The resulting mixture was extracted with EtOAc (3 x 5 mL). The combined organic layers were washed with brine (3 x 10 mL), dried over anhydrous Na_2SO_4. After filtration, the filtrate was concentrated under reduced pressure. The residue was purified by reverse phase flash with the following conditions (Column:C18 Column; Mobile Phase A: Water (0.05% $NH_3 \cdot H_2O$), Mobile Phase B: ACN; Flow rate: 50 mL/min; Gradient: 0% B to 100% B in 25 min, 66% B; Wave Length: 254/220 nm; RT1(min): 5; Number of Runs: 0) to afford (3*R*,4*R*,5*S*)-N-((6^3*S*,4*S*,*Z*)-1^2-(5-(4-cyclopropylpiperazin-1-yl)-2-((*S*)-1-methoxyethyl)pyridin-3-yl)-1^1-ethyl-10,10-dimethyl-5,7-dioxo-6^1,6^2,6^3,6^4,6^5,6^6-hexahydro-1^{1}H-8-oxa-2(4,2)-thiazola-1(5,3)-indola-6(1,3)-pyridazinacycloundecaphane-4-yl)tetrahydro-2*H*-pyran-4-carboxamide (172 mg, 65% yield) as a solid. ^{1}H NMR (400 MHz, DMSO-d_6) δ 8.58 (d, *J* = 8.9 Hz, 1H), 8.46 (dd, *J* = 13.5, 2.2 Hz, 2H), 7.80 (s, 1H), 7.73 (dd, *J* = 8.6, 1.6 Hz, 1H), 7.55 (d, *J* = 8.7 Hz, 1H), 7.21 (d, *J* = 2.8 Hz, 1H), 5.55 (t, *J* = 9.1 Hz, 1H), 5.09 (d, *J* = 12.2 Hz, 1H), 4.32 – 4.10 (m, 5H), 3.83 (t, *J* = 8.0 Hz, 2H), 3.68 – 3.60 (m, 2H), 3.57 (s, 2H), 3.21 (d, *J* = 6.2 Hz, 7H), 3.12 (dd, *J* = 14.7, 9.2 Hz, 1H), 2.93 (d, *J* = 14.4 Hz, 1H), 2.75 (s, 1H), 2.68 (d, *J* = 5.4 Hz, 5H), 2.44 (d, *J* = 14.4 Hz, 1H), 2.12 – 2.03 (m, 1H), 1.92 (ddd, *J* = 18.6, 7.2, 4.0 Hz, 2H), 1.78 (s, 2H), 1.65 (p, *J* = 3.2 Hz, 2H), 1.57 – 1.44 (m, 1H), 1.33 (d, *J* = 6.0 Hz, 3H), 1.23 (s, 1H), 0.96 – 0.82 (m, 7H), 0.43 (dt, *J* = 6.2, 2.9 Hz, 2H), 0.34 (d, *J* = 6.1 Hz, 5H). ^{13}C NMR (101 MHz, $CDCl_3$) δ 173.26, 171.90, 171.60, 171.02, 169.54, 165.40, 157.86, 155.20, 150.68, 150.12, 146.03, 145.30, 139.39, 138.71, 136.76, 135.63, 135.34, 130.15, 129.66, 126.93, 126.80, 126.29, 125.79, 125.30, 120.65, 119.36, 118.04, 111.88, 111.83, 111.32, 110.39, 109.83, 109.48, 77.43, 77.11, 76.79, 75.51, 75.05, 73.13, 70.97, 69.39, 69.33, 58.52, 57.62, 56.25, 52.78, 49.27, 48.36, 45.17, 41.72, 39.78, 38.83, 38.43, 38.19, 37.22, 35.70, 32.73, 31.89, 29.66, 29.33, 29.01, 26.18, 26.13, 26.00, 25.42, 24.98, 24.32, 23.77, 23.51, 23.26, 22.67, 18.53, 16.48, 15.37, 14.99, 14.12, 5.84, 0.00. HRMS (ESI$^+$, m/z): [M+H] calc'd for $C_{47}H_{61}N_8O_6S$ 865.4429; found 865.4401.

Methods

Tri-complex crystallization: CypA and RAS proteins were expressed and purified as previously described[32]. Purified human CypA and RAS (untagged WT and mutants) bound to GMPPNP were combined in a 2:1 CypA:RAS molar ratio in a buffer solution consisting of 12.5 mM HEPES-NaOH pH 7.3, 75 mM NaCl, 5 mM $MgCl_2$. Tri-complex inhibitor was added from a 10 mM DMSO stock to give solutions of 100 µM RAS, 200 µM CypA, and 300 µM tri-complex inhibitor in 1 mL total volume. These mixtures were incubated for 5 minutes on ice and the tri-complexes were purified via gel filtration using a Superdex 75 10/300 GL column pre-equilibrated with a buffer consisting of 12.5 mM HEPES-NaOH pH 7.3, 75 mM NaCl, 5 mM $MgCl_2$. Fractions containing the tri-complex were pooled and concentrated to 15 mg/mL using an Amicon Ultra-4 30K centrifugal filter (Millipore Sigma). Crystallization conditions can be found in Supplementary Table 1). 0.3 µL of well solution was mixed with 0.3 µL of the concentrated tri-complex in a sitting drop and the plate was incubated at 18 °C. Crystals grew overnight and were vitrified following cryoprotection via mother liquor supplemented with 12.5% glycerol.

Crystallography data collection and refinement

X-ray diffraction datasets were collected at the Stanford Synchrotron Radiation Lightsource (SSRL BL9-2, BL12-2, or BL14-1, Menlo Park, California, USA) and the Canadian Light Source (CLSI-08ID-1, Saskatoon, Saskatchewan, Canada) at 100K. Data were processed with XDS (*31*), and initial structures were determined via Phaser (*32*) using previously solved KRAS and CypA as molecular replacement search models. Ligand restraints were generated using AceDRG (*33*). The final structures were determined through iterative rounds of model building using Coot (*34*) and refinement using REFMAC5 from the CCP4 suite (*35*) and phenix.refine (*36*). Final processing and refinement statistics can be found in **Supplementary Table 5**.

Supplementary Table 5.

Tri-complex inhibitor	Compound 2	Compound 3	Compound 6	Compound 10	RMC-6236

RAS isoform and mutant	$KRAS^{G12V}$	$KRAS^{G12V}$	$KRAS^{G12V}$	$KRAS^{G12V}$	$KRAS^{WT}$
Crystallization conditions	28% PEG 3350, 0.1 M Bis Tris, pH 5.5, 150 mM NaCl	28% PEG 3350, 0.1 M Bis Tris, pH 5.5, 150 mM NaCl	28% PEG 3350, 0.1 M Bis Tris, pH 5.5, 150 mM NaCl	28% PEG 3350, 0.1 M Bis Tris, pH 5.5, 150 mM NaCl	25% PEG 6000, 0.1 M imidazole pH 7
**PDB ID**	9BG4	9BG1	9BG7	9BG2	9BG9
**Crystal parameters**					
Wavelength	0.954	1.195	1.195	0.953	0.979
Space group	$P\ 1\ 2_1\ 1$	$P\ 1\ 2_1\ 1$	$P\ 1\ 2_1\ 1$	$P\ 2_1\ 2_1\ 2_1$	$P\ 2_1\ 2_1\ 2_1$
Unit cell length, a,b,c (Å)	48.1, 101.6, 66.2	48.0, 104.7, 66.3	47.7, 101.9, 66.7	66.4, 85.6, 127.3	66.1, 84.2, 128.0
Unit cell angle, α, β, γ (°)	90, 90.9, 90	90, 90.9, 90	90, 90.8, 90	90, 90, 90	90, 90, 90
**Diffraction data**					
Resolution range (Å)	40.3-1.14 (1.18-1.14)	52.4-1.51 (1.56-1.51)	39.1-1.60 (1.66-1.60)	58.9-1.64 (1.70-1.64)	52.0-1.58 (1.64-1.58)
Total reflections	1489590 (87059)	341422 (25012)	253401 (4401)	969968 (103650)	654136 (65832)
Unique reflections	220417 (17280)	100049 (7353)	76408 (2034)	88855 (8775)	97878 (9666)
Multiplicity	6.8 (5.0)	3.4 (3.4)	3.3 (2.2)	10.9 (11.8)	6.7 (6.8)
Completeness	95.41 (74.91)	97.8 (97.2)	91.1 (53.9)	99.29 (99.35)	99.40 (99.27)
Mean I/σ(I)	9.63 (0.96)	6.7 (1.3)	10.8 (1.4)	11.23 (0.95)	15.29 (0.82)
Wilson B-factor	12.6	16.1	17.0	26.9	26.4
R-pim	0.035 (0.676)	0.055 (0.575)	0.045 (0.615)	0.034 (0.861)	0.025 (1.018)
CC1/2	0.999 (0.363)	0.988 (0.591)	0.992 (0.472)	0.999 (0.361)	0.999 (0.369)
**Refinement**					

Number of non-hydrogen atoms	6735	6451	6326	6032	6065
Macromolecules	5378	5308	5295	5226	5287
Ligands	296	178	188	168	182
Solvent	1061	965	843	638	596
Protein residues	671	667	668	661	665
Reflections used in refinement	220312 (17262)	99866 (9830)	76197 (4502)	88804 (8774)	97824 (9619)
R_{work}	0.141 (0.269)	0.187 (0.312)	0.165 (0.236)	0.200 (0.337)	0.204 (0.421)
R_{free}	0.177 (0.302)	0.226 (0.325)	0.203 (0.278)	0.229 (0.369)	0.232 (0.412)
RMS bonds (Å)	0.010	0.012	0.006	0.011	0.006
RMS angles (°)	1.16	1.21	0.79	1.13	0.79
Ramachandran favored (%)	97.29	97.42	97.12	97.40	96.96
Ramachandran outliers (%)	0	0	0	0	0
Clash score	1.63	2.7	1.8	2.6	2.6

Tri-complex inhibitor	RMC-6236	RMC-6236	RMC-6236	RMC-6236	RMC-6236
RAS isoform and mutant	$KRAS^{G12C}$	$KRAS^{G12R}$	$KRAS^{G12V}$	$KRAS^{G13D}$	$KRAS^{Q61H}$
Crystallization conditions	28% PEG 4000, 0.1 M Tris pH 8	22% PEG 2000 MME, 0.1 M Tris pH 8	24% PEG 4000, 0.1 M Imidazole pH 7	24% PEG 4000, 0.1 M Imidazole pH 7	29% PEG 4000, 0.1 M Tris pH 8
**PDB ID**	9BGA	9BGC	9BG6	9BG5	9BGB
**Crystal parameters**					
Wavelength	0.953	0.953	1.000	0.953	0.953
Space group	$P\ 2_1\ 2_1\ 2_1$	$P\ 2_1\ 2_1\ 2_1$	$P\ 2_1\ 2_1\ 2_1$	$P\ 2_1\ 2_1\ 2_1$	$P\ 2_1\ 2_1\ 2_1$
Unit cell length, a,b,c (Å)	65.6, 81.6, 127.3	66.4, 85.9, 128.3	66.4, 84.2, 127.8	66.3, 84.5, 128.3	65.2, 80.6, 127.3

Unit cell angle, α, β, γ (°)	90, 90, 90	90, 90, 90	90 90 90	90, 90, 90	90, 90, 90
**Diffraction data**					
Resolution range (Å)	58.3-1.41 (1.46-1.41)	51.4-1.87 (1.94-1.87)	63.9-1.66 (1.72-1.66)	40.5-1.67 (1.73-1.67)	39.6-1.68 (1.74-1.68)
Total reflections	1754558 (174016)	448176 (41339)	560865 (57160)	624997 (59115)	580914 (60097)
Unique reflections	131773 (13050)	60989 (5840)	85218 (8403)	84194 (8308)	76785 (7556)
Multiplicity	13.3 (13.3)	7.3 (7.1)	6.6 (6.8)	7.4 (7.1)	7.6 (8.0)
Completeness	99.95 (99.87)	98.52 (95.02)	99.91 (99.89)	99.88 (99.64)	99.62 (99.39)
Mean I/σ(I)	13.6 (0.89)	9.77 (1.15)	13.03 (0.86)	10.53 (0.86)	10.46 (0.81)
Wilson B-factor	21.7	24.75	28.93	28.88	26.14
R-pim	0.024 (0.85)	0.04687 (0.6803)	0.02833 (0.9224)	0.0378 (0.9926)	0.04336 (1.057)
CC1/2	0.999 (0.339)	0.998 (0.694)	0.999 (0.372)	0.998 (0.342)	0.999 (0.33)
**Refinement**					
Number of non-hydrogen atoms	6166	5823	5939	5980	5797
Macromolecules	5281	5223	5236	5250	5210
Ligands	182	182	182	182	182
Solvent	703	418	521	548	405
Protein residues	666	665	665	666	663
Reflections used in refinement	131761 (13046)	60467 (5730)	85205 (8399)	84174 (8295)	76774 (7553)
R_{work}	0.1865 (0.2980)	0.2298 (0.5900)	0.2070 (0.4207)	0.2234 (0.4076)	0.1981 (0.3862)
R_{free}	0.2281 (0.3456)	0.2618 (0.6055)	0.2400 (0.4334)	0.2666 (0.4380)	0.2304 (0.4205)
RMS bonds (Å)	0.005	0.008	0.006	0.006	0.007
RMS angles (°)	0.86	0.91	0.79	0.8	0.83

Ramachandran favored (%)	97.57	96.5	97.56	96.5	97.71
Ramachandran outliers (%)	0	0	0	0	0
Clash score	1.59	1.8	1.51	2.1	1.23

Tri-complex inhibitor	RMC-6236	RMC-6236	RMC-6236	RMC-6236
RAS isoform and mutant	$NRAS^{WT}$	$NRAS^{Q61K}$	$NRAS^{Q61L}$	$NRAS^{Q61R}$
Crystallization conditions	29% PEG 4000, 0.1 M Tris pH 8	25.5% PEG 4000, 0.1 M Tris pH 8	21% PEG 3350, 0.1M Bis-Tris pH 5.5, 133mM NaCl	25% PEG 3350, 0.1M Bis Tris, pH 5.5, 150 mM NaCl
**PDB ID**	9BG0	9BG3	9BGD	9BG8
**Crystal parameters**				
Wavelength	0.953	0.953	0.953	0.953
Space group	$P\ 2_1\ 2_1\ 2_1$	$P\ 2_1\ 2_1\ 2_1$	$P\ 2_1\ 2_1\ 2_1$	$P\ 2_1\ 2_1\ 2_1$
Unit cell length, a,b,c (Å)	65.5, 81.7, 127.9	65.9, 84.1, 128.8	66.3, 85.5, 128.4	66.0, 84.1, 127.4
Unit cell angle, α, β, γ (°)	90, 90, 90	90, 90, 90	90, 90, 90	90, 90, 90
**Diffraction data**				
Resolution range (Å)	34.7-1.64 (1.70-1.64)	46.1-1.33 (1.38-1.33)	58.9-1.76 (1.82-1.76)	40.3-1.2 (1.24-1.20)
Total reflections	1146476 (117868)	1219514 (123090)	545179 (55002)	1625115 (150655)
Unique reflections	84668 (8332)	164077 (16205)	72994 (7197)	220757 (21857)
Multiplicity	13.5 (14.1)	7.4 (7.6)	7.5 (7.6)	7.4 (6.9)
Completeness	99.96 (99.95)	99.82 (99.50)	99.94 (99.97)	99.88 (99.81)
Mean I/σ(I)	12.28 (0.98)	9.20 (0.83)	11.62 (1.00)	11.40 (1.05)

Wilson B-factor	25.62	18.09	32.38	15.25
R-pim	0.04115 (0.82)	0.03608 (0.9382)	0.03442 (0.7979)	0.02848 (0.8667)
CC1/2	0.999 (0.354)	0.998 (0.346)	0.999 (0.408)	0.999 (0.399)
**Refinement**				
Number of non-hydrogen atoms	6019	6474	5935	6504
macromolecules	5213	5334	5249	5319
Ligands	182	183	182	210
Solvent	624	957	504	975
Protein residues	665	667	667	666
Reflections used in refinement	84653 (8330)	164052 (16199)	72987 (7195)	220735 (21853)
R_{work}	0.2067 (0.3552)	0.1699 (0.3373)	0.2000 (0.3767)	0.1526 (0.2793)
R_{free}	0.2361 (0.3695)	0.1981 (0.3821)	0.2374 (0.4044)	0.1859 (0.2912)
RMS bonds (Å)	0.007	0.006	0.007	0.005
RMS angles (°)	0.84	0.86	0.84	0.83
Ramachandran favored (%)	97.56	97.57	97.12	97.26
Ramachandran outliers (%)	0	0	0	0
Clash score	1.7	2.12	1.32	1.47

Values in parentheses are for highest-resolution shell

CypA binding affinity: The binding affinity of compounds for CypA was assessed by surface plasmon resonance (SPR) on Biacore 8K instrument. AviTag-CypA was immobilized on a streptavidin sensor chip by diluting CypA stock solutions to a final concentration of 1uM or 0.2uM in assay buffer (10 mM HEPES-NaOH pH 7.4, 150 mM NaCl, 0.005% v/v Surfactant P20, 2% v/v DMSO) and then using the built in Biacore 8K protocol at a flow rate of 30uL/minute for 10 seconds.Varying compound concentrations, diluted in the assay buffer without added DMSO and injected from low to high, were

flowed over the chip in assay buffer for 20 to 30 seconds followed by a dissociation wash with assay buffer only for 180 seconds. The SPR sensorgrams were fit using either a steady state affinity model or a 1:1 binding (kinetic) model to assess the K_D1 for CypA binding.

RAS-BRAF Disruption by TR-FRET: Disruption of the interactions between wild-type RAS or mutant RAS and the RAS-binding domain of BRAF were assessed by TR-FRET in reactions consisting of 12.5 nM His6- RAS [1-169], 50 nM GST-BRAF[155-229], and 25 µM CypA in reaction buffer (25 mM HEPES-NaOH pH 7.3, 0.002% Tween20, 0.1% bovine serum albumin, 100 mM NaCl, 5 mM MgCl2). Compounds or DMSO control (1% v/v) were added and incubated for 3 hours, followed by the addition of 10 nM LANCE Eu-W1024 anti-6xHis antibody (Perkin Elmer AD0111) with 50 nM Allophycocyanin-anti-GST antibody (Perkin Elmer AD0059G) in reaction buffer and an additional 1.5 hours incubation.TR-FRET was measured on a Perkin Elmer Envision plate reader (excitation at 320 nm, 20 µsec delay, 100 µsec window, 2000 µsec time between flashes; Emission at 665 nm and 615 nm in separate channels). The FRET ratio (665/615 nM emission) was used to calculate % Inhibition as: (1 – (FRET Ratio of Sample – Average FRET Ratio of Positive Controls)/(Average FRET Ratio of DMSO Control – Average FRET Ratio of Positive Controls)) x 100%.

RAS binding affinity: The binding affinity of compound-bound CypA for the mutant oncogenic or wild-type RAS proteins (K_D2) mentioned was assessed by SPR on a Biacore 8 K instrument. AviTag-RAS [residues 1–169] was immobilized on a streptavidin sensor chip by diluting RAS stock solutions to a final concentration of 10nM in assay buffer (10 mM HEPES-NaOH pH 7.4, 150 mM NaCl, 0.005% v/v Surfactant P20, 0.5mM EDTA pH 8.0, 10uM GMPPNP, and 2% v/v DMSO) and then using the built in Biacore 8K protocol at a flow rate of 10uL/minute for 20 seconds targeting <= 40RU. Varying compound concentrations, from low to high, were flowed over the chip in assay buffer, without DMSO and 25 µM CypA added, at a flow rate of 80uL/min for 20 seconds of association followed by 180 seconds of dissociation by flowing over assay buffer with 25uM of CypA at the same flow rate. The SPR sensorgrams were fitted using either a steady state affinity model or a 1:1 binding (kinetic) model to assess the dissociation constant (K_D2) for RAS binding.

Cellular ERK phosphorylation assays: Cells were plated in tissue culture-treated 384-well plates and incubated overnight. The following day, serial dilutions of compound were prepared in growth medium. Cells were treated with compound or DMSO control (0.1%) for 4 hours. Following the incubation period, cells were lysed and lysates were transferred to a 384-well plate (Perkin Elmer OptiPlate-384). Level of ERK phosphorylation was determined using the AlphaLISA® SureFire Ultra p-ERK1/2 Assay Kit (Perkin Elmer ALSU-PERK-A50K), following the manufacturer's instructions. The

assay was read in a Perkin Elmer Envision reader. A low signal control (LC) of 1 µM trametinib was included on each plate. The data were normalized to the DMSO control (HC) and LC using the Percent-of-control method, then fit to a sigmoidal curve for EC_{50} calculation in GeneData Screener.

2D cell proliferation: For 2D cellular proliferation assays, cells were plated in tissue culture-treated 96-well plates and incubated overnight. The following day, 10 serial dilutions of compound were prepared in growth medium. Cells were treated with compound or DMSO (0.1% v/v) for 120 hours. 50 µL of CellTiter-Glo 2.0 reagent (G9243, Promega) was added to each well. Plates were shaken at 450 rpm for 2 minutes at room temperature and then incubated for an additional 10 minutes without shaking at room temperature and protected from light. luminescence was detected using the SpectraMax M5 Plate Reader (Molecular Devices). Luminescence signal from the CellTiter-Glo® 2.0 reagent was normalized to vehicle treated wells (% vehicle = $(lum_{treated}/mean(lum_{vehicle}))*100$). Data were plotted as a function of log molar [inhibitor] and a 4-parameter sigmoidal concentration response model was fitted to the data to calculate the EC_{50}.

In Vitro Permeability: Compounds were prepared at 5 µM in HBSS (10 mM HEPES, pH 7.4) and dosed to the apical or basolateral side of a Caco-2 monolayer system; blank HBSS (10 mM HEPES, pH 7.4) containing 4% BSA was added to the receiver side. Cells were incubated for 2 hours at 37 °C with 5% CO_2 and 95% relative humidity. Samples from both donor and acceptor compartments were analyzed for test article using LC-MS/MS methods to calculate apparent permeability and efflux ratio.

ePSA Method.

Chemicals and Reagents. The mobile phase modifier consists of ammonium formate at 20 mM in HPLC grade methanol. The liquid CO2 is obtained from (beverage grade) 50 lb tanks featuring eductor tubes and used directly.

Standards and Sample Preparation. One mg of each standard is dissolved in 1 mL of DMSO and diluted 1/10 with methanol. Standards include chlorpromazine, desipramine, pindolol, diclofenac sodium salt, nitrobenzoic acid, bumetanide, and furosemide. Methanol (100%) is used as a blank. Each sample is obtained as a 10 mM stock solution and 10 mL diluted 1/10 with methanol. The samples are sonicated if the solution appears cloudy or crystals are observed to be present.

Instrumentation. The SFC/MS instrument used in this experiment is an Agilent supercritical fluid chromatograph system with Aurora CO_2 pump. The mass spectrometer used is a single quadrupole MSD detector with an ESI source. The effluent of the SFC is split to the MSD.

Analysis Conditions. Analysis is performed using a 4.6 mm × 50 mm Chirex 3014 (S)-column with 5 µm particle and 100 Å pore size. The column temperature is set to 35 °C. The flow rate is 3 mL/min with the outlet backpressure set to 140 bar. The injection volume is 2 µL. The mobile phase composition is varied from 5% to 65% modifier at 5%/min in a linear gradient after a 1 min hold, holding at 65% for 2 min and reverting to the original 5% until the end of the 14 min run. Total analysis time is 15 min. Detection consists of UV at 254 nm and MSD in total ion current (TIC) mode. The regression of the retention time and the PSA value for the standards is used to calculate the compound's EPSA value with its retention time.

Kinetic Solubility Method – PBS at pH 7.4 and Sodium Citrate at pH 5

Chemicals and Reagents. Sterile phosphate-buffered saline (PBS 1x, purchased from Corning) without calcium and magnesium is used. Separately sodium citrate dihydrate is prepared at 10 mM and pH adjusted to 5 with citric acid. Diclofenac is used as a control and dissolved in DMSO to make a 25 mM stock solution. A 0.1% Formic acid mobile phase is prepared in water (aqueous) and separately in acetonitrile (organic).

Standards and Sample Preparation. A 25 mM DMSO stock solution of each sample is prepared from powder. The stock solutions are sonicated if the solution appears cloudy or crystals are observed to be present. The diclofenac control as well as standards are prepared from each of these 25 mM stock solutions and diluted with 50% acetonitrile / water to final levels: 100, 50, 10, 5, 1 mM. In a deep well polypropylene 96-well plate (Millipore MultiScreen PCF), solubility samples are prepared in triplicate by diluting the 25mM DMSO stock solution with appropriate buffer (PBS x1 and separately sodium citrate) to a nominal concentration of 500 mM. The 96-well plates are then sealed and placed on an orbital shaker at 300 rpm for 5 hours. An aliquot of 150 mL is then vacuum filtered through the Multiscreen plate, the filtrate is diluted 10x with acetonitrile / water (1:1) and analyzed by HPLC.

Instrumentation. The instrument used in for the Ksol analysis is an Agilent 1290 , with quaternary pumping system and an enhanced sensitivity G7117B PDA UV detector.

Analysis Conditions. Quantitative analysis is performed using a 3.0 mm × 30 mm Agilent HPH C18 with 1.9 µm particle and 120 Å pore size. The column temperature is set to 30 °C. The flow rate is 1.1 mL/min. The injection volume is 4 µL. The mobile phase composition is varied from 15% to 98% acetonitrile with 0.1% formic acid in a 1.8 min linear gradient after a 0.1 min hold, holding at 98% acetonitrile (with 0.1% formic acid) for 0.4 min. Total analysis time per analysis is 2.5 min. UV detection

is carried out at 254 nm. Linear regression of the response factor for the standard is used to calculate each compound's filtrate concentration and reported as the Ksol value in the respective buffer at 5 hours.

LogD Method.

Chemicals and Reagents. The mobile phases consists of 10 mM sodium phosphate (pH 7.4) for the aqueous solvent and methanol for the organic solvent. The sample diluent is methanol.

Standards and Sample Preparation. One mg of each standard is dissolved in 1 mL of DMSO and diluted 1/10 with methanol. Standards include benzophenone, diphenyl ether, phenanthrene, triphenylamine, and dichloro-diphenyl-trichloroethane. Methanol (100%) is used as a blank. Each sample is obtained as a 25 mM stock solution and 10 uL diluted 1/10 with methanol.

Instrumentation. The instrument used for the LogD analysis is an Agilent 1290 II uPLC system.

Analysis Conditions. Analysis is performed using a 3.0 mm × 30 mm Agilent Poroshell 120 EC-C18 with a 1.9 µm 120Å pore size. The flow rate is 0.9 mL/min. The injection volume is 3 µL. The mobile phase composition varied from 3% to 93.25% methanol at 9.5%/min in a linear gradient, holding at 93.25% for 2 min and reverting to the original 3% until the end of the 13.5 min run. Data is acquired for 12 minutes, including rinsing and equilibration time, which results in an injection-to-injection time of 14 minutes. Each sample is analyzed in duplicate, resulting in a sample-to-sample turnaround time of 28 minutes. The column temperature is set to 30 °C. The MSD is used in total ion current (TIC) mode. The detection wavelength for UV is 254 nm. The regression of the retention time and the logD value for the standards are used to calculate the compound's logD value with its retention time.

Microsomal Stability: Compounds were incubated at 2 µM with 0.5 mg/mL mouse liver microsomes in the presence of 1 mM NADPH in phosphate bufferat 37 °C. The reactions were quenched at 0, 15, 30, 45, and 60 min with the addition of 3 volumes of cold acetonitrile containing internal standards (200 nM labetalol, 100 nM tolbutamide, 100 nM ketoprofen), and the samples were centrifuged at 3220 *g* for 45 min. The disappearance of test article was monitored by LC-MS/MS methods to calculate intrinsic clearance and $t_{1/2}$.

Blood-Plasma Partitioning: Compounds were incubated at 01, 0.5, 1, 5, or 10 µM in mouse whole blood or control plasma for 60 min at 37 °C. After incubation, whole blood samples were processed to plasma by centrifugation (10,000 g for 10 min at 37 °C). All plasma samples were quenched with the addition of 4 volumes of acetonitrile containing internal standards (200 nM labetalol and 200 nM imipramine), and the samples were centrifuged at 16,000 *g* for 15 min. The supernatants were analyzed for test article concentration using LC-MS/MS methods to determine the ratio between control plasma

and test samples. Test article stability after a 60 min incubation was also assessed by quenching an aliquot of whole blood with the addition of 7 volumes of acetonitrile containing internal standards followed by centrifugation at 3220 *g* for 30 min.

Blood Stability: Compounds were incubated at 1 μM in whole blood at 37 °C. Aliquots of whole blood samples were quenched at 0, 15, 30, 45, 60, and 120 min with the addition of 7 volumes of acetonitrile containing internal standards (100 nM alprazolam, 200 nM labetalol, 200 nM imipramine, 2000 nM ketoprofen) followed by centrifugation at 3220 *g* for 30 min.. The disappearance of test article was monitored by LC-MS/MS methods to calculate $t_{1/2}$.

Hepatocyte Stability: Compounds were incubated at 1 μM in hepatocytes (0.5 million cells/mL) suspended in medium (Williams E Medium and GlutaMAX) at 37 °C with 5% CO_2. The reactions were stopped at 0. 15, 30, 60, and 120 min with the addition of 6 volumes of cold acetonitrile containing internal standards (100 nM alprazolam and 200 nM labetalol), and the samples were centrifuged at 3220 *g* for 25 min. The disappearance of test article was monitored by LC-MS/MS methods to calculate $t_{1/2}$.

Mouse PK Studies: Female Balb/c mice were non-fasted on the day of dosing. PK was evaluated following a single dose via IV (1 mg/kg) or PO (10 mg/kg). A formulation containing NMP/EtOH/PEG400/Solutol/water (10/10/10/10/60; %v/v/v/v/v) was used for IV administration with a dose volume of 5 mL/kg. A formulation containing DMSO/PEG400/Solutol/HPMC (10/20/10/2; %v/v/v/v) in 50 mM sodium citrate buffer or DMSO/PEG400/Solutol/water (10/20/10/60; %v/v/v/v) was used for PO administration with a dose volume of 10 mL/kg. Serial blood samples were collected at 0.033 (IV only), 0.083 (IV only), 0.25, 0.5, 1, 2, 4, 8, 24 h post-dose and stored on wet ice. For plasma PK, blood samples were processed by centrifugation (4600 rpm for 5 min at 4 °C) within 30 min of collection. Plasma and/or blood samples were stored at -80 °C until analysis.

Rat, Dog, and Monkey PK Studies: Male Sprague-Dawley rats were non-fasted on the day of dosing, while non-naïve male beagle dogs or non-naïve male cynomolgus monkeys were fasted overnight and fed 4 hours post-dose. PK was evaluated following a single dose via IV (1 mg/kg) or PO (2.5, 5, or 60 mg/kg). A formulation containing NMP/EtOH/PEG400/Solutol/water (10/10/10/10/60; %v/v/v/v/v) was used for IV administration to rats with a dose volume of 2 mL/kg. A formulation containing NMP/EtOH/PEG400/water (10/10/10/70; %v/v/v/v) was used for IV administration to dogs and monkeys with a dose volume of 1 mL/kg. A formulation containing DMSO/PEG400/Solutol/water (10/20/10/60; %v/v/v/v) was used for PO administration to rats with a dose volume of 10 mL/kg. A formulation containing DMSO/PEG400/Solutol/water (10/30/2/58; %v/v/v/v) was used for PO administration to dogs and monkeys with a dose volume of 5 mL/kg. Serial blood samples were collected at 0.0167 (dog and

monkey IV only), 0.033 (rat IV only), 0.083 (IV only), 0.25, 0.5, 1, 2, 4, 8, 24 h post-dose and stored on wet ice. For plasma PK, blood samples were processed by centrifugation (3000 rpm for 10 min at 4 °C) within 30 min of collection. Plasma and/or blood samples were stored at -80 °C until analysis.

Bioanalysis of PK Samples: Quantification of analytes was determined using LC-MS/MS methods. Whole blood or plasma samples were extracted with a 20X volume of acetonitrile or acetonitrile/methanol (1:1; %v/v) containing internal standard (terfenadine, tolbutamide, propranolol, or diclofenac). After mixing and centrifugation, the supernatant was diluted as needed with methanol/water (1:1; %v/v, with 0.1% formic acid) and analyzed on a Sciex 4000, Sciex 5500, or Sciex 6500+ triple quadrupole mass spectrometer coupled with a Shimadzu UPLC system. An ACE Excel 5 C4, Kinetex C18, or Waters X-Bridge BEH C18 column was used with gradient elution for compound separation. Analytes of interest were detected by positive electrospray ionization using multiple reaction monitoring.

Xenograft Studies

Cell Culture: Studies were conducted at Wuxi AppTec (Suzhou, China). NCI-H441 tumor cells (ATCC HTB-174) were maintained *in vitro* as a monolayer culture in RPMI-1640 medium supplemented with 10% heat inactivated fetal bovine serum, 100 U/mL penicillin and 100 µg/mL streptomycin at 37°C in an atmosphere of 5% CO_2 in air. MIA PaCa-2 tumor cells were maintained *in vitro* as a monolayer culture in DMEM/F12 medium supplemented with 10% heat inactivated fetal bovine serum at 37°C in an atmosphere of 5% CO_2 in air. HPAF II tumor cells (ATCC, CRL-1997) were maintained *in vitro* as a monolayer culture in EMEM medium supplemented with 10% heat inactivated fetal bovine serum, 100 U/mL penicillin and 100 µg/mL streptomycin at 37 °C in an atmosphere of 5% CO_2 in air. NCI-H1373 tumor cells (ATCC, CRL-5866) were maintained *in vitro* as a monolayer culture in RPMI-1640 medium supplemented with 10% fetal bovine serum, 100 U/mL penicillin and 100 µg/mL streptomycin at 37 °C in an atmosphere of 5% CO_2 in air. Tumor cells were subcultured twice weekly. Cells growing in an exponential growth phase were harvested and counted for tumor inoculation. Female BALB/c nude mice 6-8 weeks old from Shanghai Sino-British Sippr/BK Laboratory Animal Co., LTD. were used for these studies. Each mouse was inoculated subcutaneously at the right flank with tumor cells (2-5 x 10^6) in 0.2 mL of PBS supplemented with Matrigel (1:1) for tumor development.

Efficacy study: Treatments started at the time of randomization when the average tumor volume of each group reached the range of 150-250 mm^3. Test article was administered daily at the indicated doses via oral gavage. Tumors were measured in two dimensions using a digital caliper, by an unblinded employee who was not involved in the design or analysis of study results. The tumor volume in mm^3 was calculated using the formula: Volume = $((\text{width})^2 \times \text{length})/2$, where width and length are measured in mm. Mice on studies were weighed and tumor measurements collected 2 times a week.

Single-dose pharmacokinetic/pharmacodynamics (PK/PD) study: Mice were randomized into treatment groups when mean tumor volume reached 400-600 mm^3. At the indicated time points after a single dose of test article at the indicated dose levels, plasma or whole blood and tumor samples were collected for PK and PD analysis, respectively. Test article was formulated in 10/20/10/60 (%v/v/v/v) (DMSO/PEG400/Solutol/water), and the vehicle formulation was used for control group.

In vivo pharmacodynamic analysis by DUSP6 qPCR: RNA was extracted from at least 20 mg of tumor tissue using RNeasy Mini Kit (Qiagen, 74104) using a High Throughput Tissue grinder and following manufacturer's protocol from the RNeasy Mini Kit. Reverse transcription was carried out using High-Capacity cDNA Reverse Transcription Kit (ABI, 4368814) according to manufacturer's protocol. The cDNA product was used for qPCR analysis using TaqMan Gene Expression Master Mix (ABI, 4369016). TaqMan primer probes specific to human DUSP6 and 18S (used as an internal control gene) were used to detect the levels from each sample in duplicates using a 10 µL final reaction volume in a 384-well clear optical reaction plate. For qPCR, Ct values of DUSP6 and 18S were obtained for analysis. DUSP6 Ct value was normalized to 18S, and then the mean relative mRNA expression levels of each group were normalized to the vehicle control group.

Incidence estimates of mutant RAS cancers: NGS data from Foundation Medicine Insights database (March 29th, 2022)[19] was analyzed for the following disease indications: non-small cell lung cancer (NSCLC), colorectal, pancreatic ductal adenocarcinoma (PDAC), endometrial renal, melanoma, bladder, esophageal, head and neck squamous cell carcinoma (HNSCC), ovarian, stomach, biliary, and carcinomas of unknown primary (CUP). The frequencies of mutations in genes *KRAS, NRAS,* and *HRAS* occurring at amino acid positions G12, G13 and Q61 were obtained. Only mutations identified at a prevalence of >=0.1% in a disease indication were included. Incidence estimates for each cancer type were obtained from ACS Cancer Facts and Figures 2023[20] and scaled by the mutational frequencies to obtain the incidence estimate of each RAS mutation for each disease indication (rounded to the nearest 100). For all disease indications, the "Estimated New Cases" values were used except for melanoma, bladder, and endometrial, where "Estimated Deaths" were used as a proxy for advanced cancers in those disease indications. For PDAC, NSCLC, and HNSCC the parent cancer types incidence estimates were further adjusted for the specific subtype (PDAC is ~90% of pancreatic cancers[21], NSCLC is ~85% of lung cancers[22], HNSCC is ~85% of head and neck cancers[19]).

HPLC Traces

Compound 6

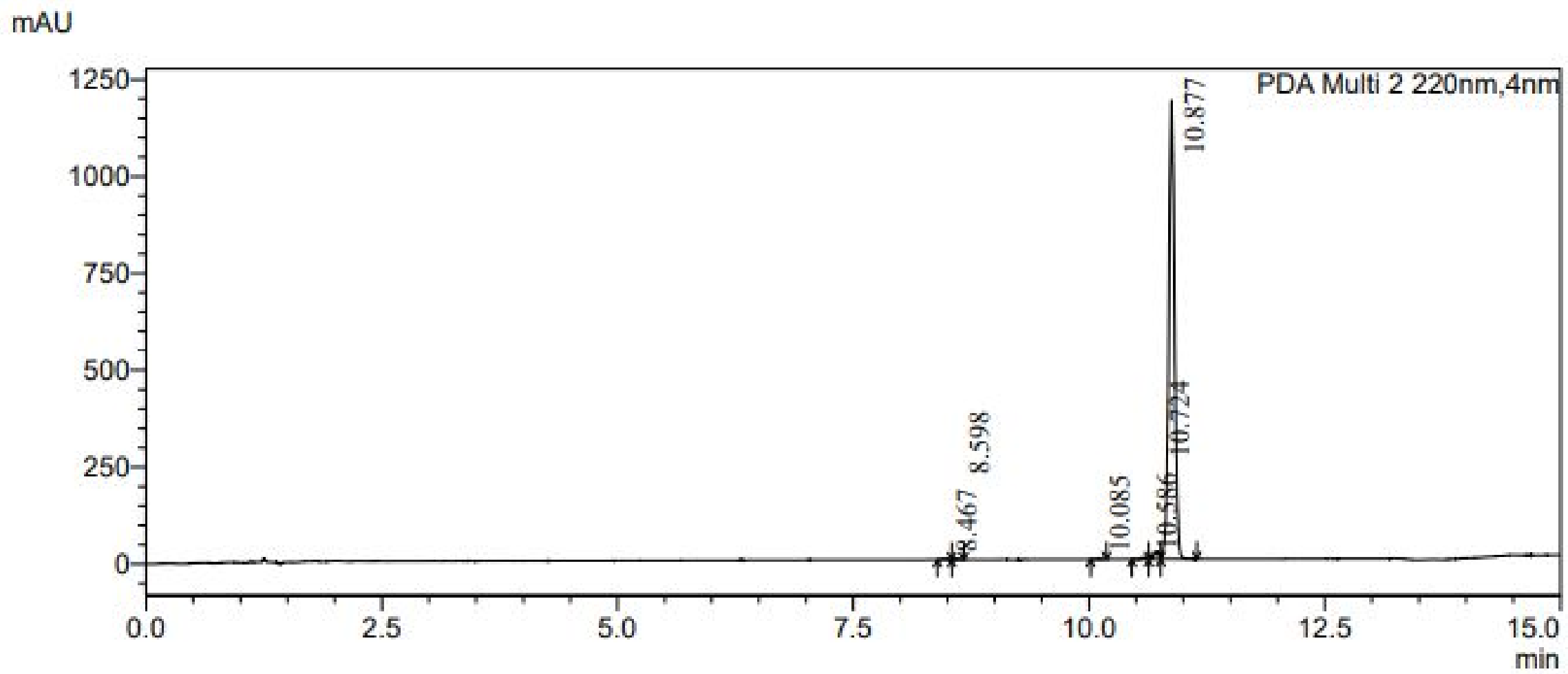

Peak Table

PDA Ch2 220nm

Peak#	Ret. Time	Height	Height%	Area	Area%
1	8.467	2289	0.190	9306	0.181
2	8.598	1734	0.144	5351	0.104
3	10.085	1229	0.102	3963	0.077
4	10.586	4204	0.348	15368	0.299
5	10.724	19708	1.632	83281	1.621
6	10.877	1178272	97.585	5020956	97.718
Total		1207436	100.000	5138225	100.000

Compound 13

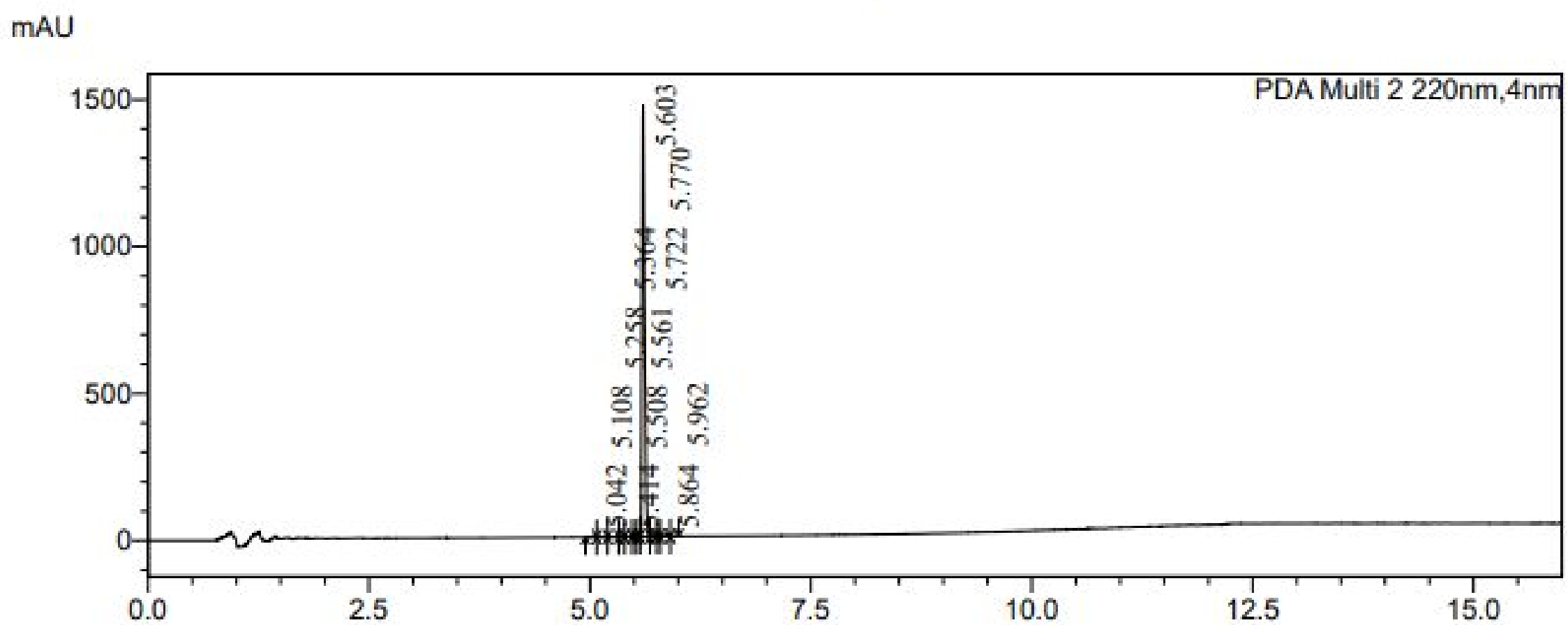

Peak Table

PDA Ch2 220nm

Peak#	Ret. Time	Height	Height%	Area	Area%
1	5.042	4424	0.304	10100	0.312
2	5.108	2422	0.166	5318	0.164
3	5.258	1060	0.073	3130	0.097
4	5.364	1912	0.131	3937	0.122
5	5.414	1354	0.093	4993	0.154
6	5.508	1137	0.078	2583	0.080
7	5.561	1072	0.074	2088	0.065
8	5.603	1425773	97.947	3144715	97.263
9	5.722	4267	0.293	17382	0.538
10	5.770	3103	0.213	7658	0.237
11	5.864	6863	0.471	22613	0.699
12	5.962	2273	0.156	8675	0.268
Total		1455660	100.000	3233194	100.000
